# Sensitization Pathways in NIR-Emitting Yb(III) Complexes Bearing 0, +1, +2, or +3 Charges

**DOI:** 10.1021/jacs.2c05813

**Published:** 2022-11-08

**Authors:** Emilie Mathieu, Salauat R. Kiraev, Daniel Kovacs, Jordann A. L. Wells, Monika Tomar, Julien Andres, K. Eszter Borbas

**Affiliations:** † Department of Chemistry, Ångström Laboratory, Uppsala University, Box 523, 75120 Uppsala, Sweden; ‡ Chemistry and Chemical Engineering Section, Ecole Polytechnique Fédérale de Lausanne (EPFL), BCH 3311, CH-1015 Lausanne, Switzerland

## Abstract

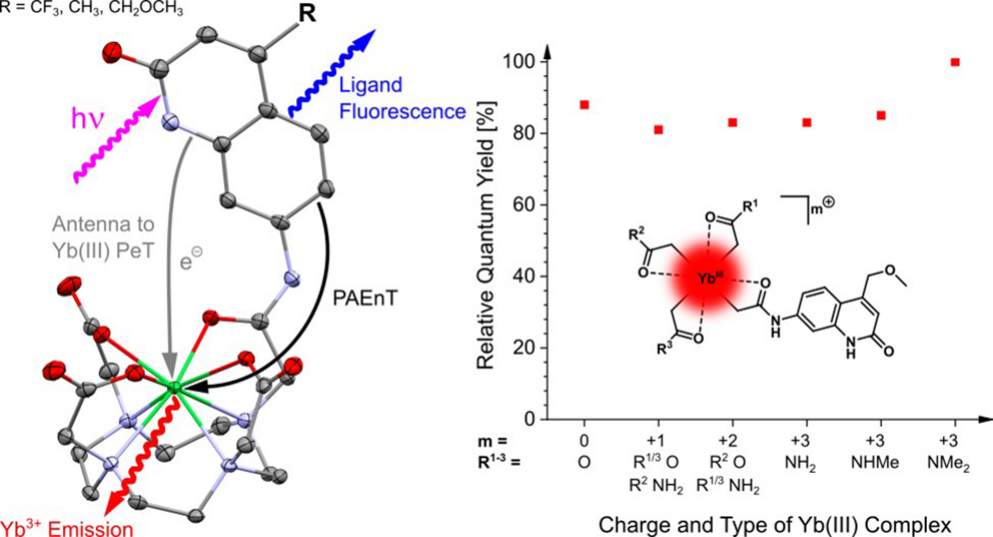

Yb(III) complexes of macrocyclic ligands based on 1,4,7,10-tetraazacyclododecane were synthesized. The ligands carried a carbostyril chromophore for Yb(III) sensitization, and carboxylate or carbamide donors for metal binding, forming complexes of 0, +1, +2, or +3 overall charge. The coordination geometry was little affected by the replacement of carboxylates with amides, as shown by paramagnetic ^1^H NMR spectroscopy. The Yb(III)/Yb(II) reduction potentials were dependent on the nature of the metal binding site, and the more positively charged complexes were easier to reduce. Carbostyril excitation resulted in Yb(III) luminescence in every complex. The residual carbostyril fluorescence quantum yields were smaller in complexes containing more reducible Yb(III) centers decreasing from 5.9% for uncharged complexes to 3.1–4.4% in +3 charged species, suggesting photoinduced electron transfer (PeT) from the antenna to the Yb(III). The relative Yb(III) luminescence quantum yields were identical within the experimental error, except for the +3 charged complex with fully methylated coordinating amides, which was the most intense Yb(III) emitter of the series in water. Quenching of the Yb(III) excited state by NH vibrations proved to limit Yb(III) emission. No clear improvement of the Yb(III) sensitization efficiency was shown upon faster PeT. This result can be explained by the concomitant sensitization of Yb(III) by phonon-assisted energy transfer (PAEnT) from the antenna triplet excited state, which was completely quenched in all of the Yb complexes. Depopulation of the triplet by PeT quenching of the donor singlet excited state would be compensated by the sensitizing nature of the PeT pathway, thus resulting in a constant overall sensitization efficiency across the series.

## Introduction

The autofluorescence of biomaterials creates a high background that sensitive luminescent probes must overcome for interference-free detection. Near-infrared (NIR) emitting probes are particularly effective at solving this problem: their signals are readily distinguished from blue-green autofluorescence, and the NIR region is a transparent window for biological matter, increasing the sensitivity of the detection, while adequate ligand design can improve the selectivity of bioimaging experiments.^1−6^ The majority of NIR emitters are based on organic fluorophores, nanoparticles, or transition-metal complexes.^7−11^

**Figure 1:**
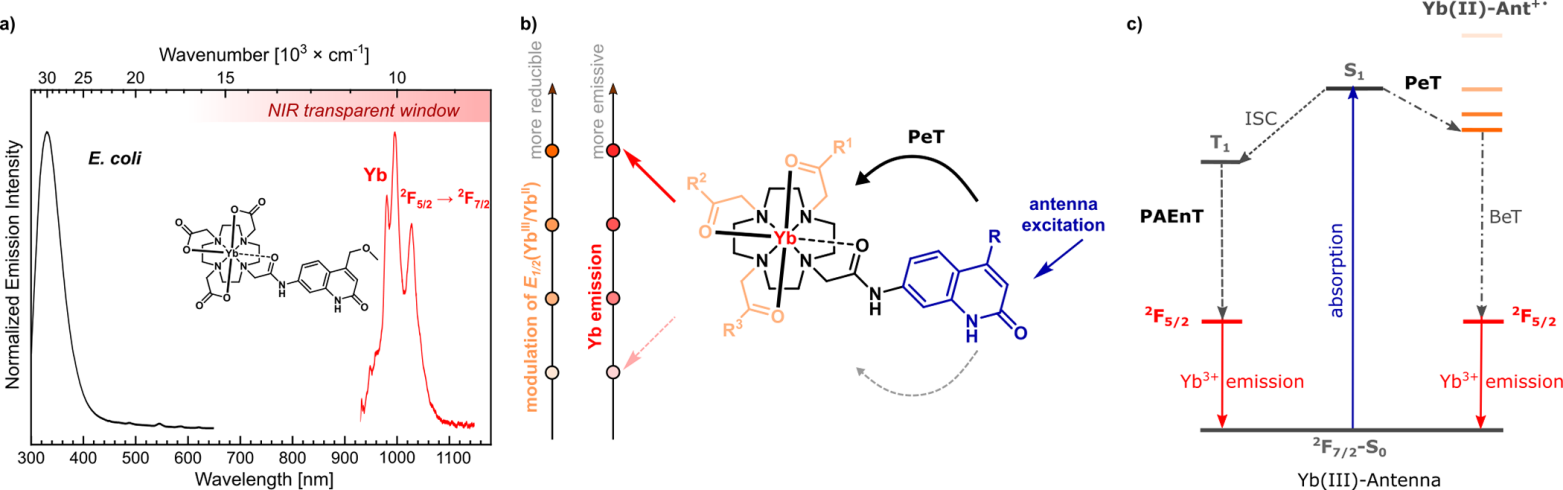
(a) NIR Ln(III) emission combined with autofluorescence from a bacterial lysate. (b) Strategy followed in this work for modulating Yb(III) luminescence. (c) Yb(III) sensitization through a photoinduced electron transfer (PeT)–back electron transfer (BeT) pathway, or through phonon-assisted energy transfer (PAEnT).

Trivalent lanthanides (Ln) emit immediately recognizable long-lived luminescence consisting of sharp peaks across the visible and NIR spectral regions (Figure 1a).^12,13^ Ln(III) excited states are usually populated via energy transfer (EnT) from the excited state of a proximal light-harvesting antenna, which overcomes the low efficiency of direct 4f–4f excitation. Although several Lns emit in the NIR, not all are equally well suited for biological applications.^1,14,15^ Sensitive, low-energy Er emission^16^ is only observed when a carefully crafted protective shell keeps the solvent molecules away from the Ln(III) center.^17^ Eu, Tm, Ho, Pr, and Sm have but a small proportion of their emissions in the NIR, and hence, low NIR-luminescence quantum yields.^18−20^ The emissions of Yb and Nd, however, are fully localized in the NIR. In addition, the gap between their emissive and receiving levels is larger than that of Er, which makes Yb and Nd less sensitive to quenching by protic solvents than Er.^16^ Finally, Yb and Nd emission lifetimes are orders of magnitude longer than autofluorescence, in the range of several microseconds rather than nanoseconds.

Yb(III) in particular is fascinating for several reasons. It has a high intrinsic quantum yield compared to Nd, Tm, and Er,^14^ mainly because of its only excited state ^2^F_5/2_ at 10 260 cm^–1^.^21^ The ^2^F_5/2_ ← ^2^F_7/2_ transition from the ground state poorly overlaps with the transitions from the excited states of the antennae that are typically located in the UV and in the high-energy visible part of the spectrum.^16^ Nevertheless, Yb(III) emission is often observed in Yb(III) complexes carrying such antennae.^22−26^

One possible mechanism populating the ^2^F_5/2_ level is a stepwise photoinduced electron transfer (PeT)–back electron transfer (BeT) process (Figure 1b,c).^22^ Despite the widespread acceptance of this sensitization route, efforts to experimentally study it have been limited. Beeby, Faulkner, and Ward showed that the sensitization mechanism in a phenanthridine-appended Yb(III) complex was pH-dependent.^24^ When protonated, the antenna became less reducing, making electron transfer (eT) from its first singlet excited state (S_1_) to Yb(III) thermodynamically unfavorable. This enabled an inefficient EnT from the first triplet excited state (T_1_) of the antenna to dominate sensitization via dissipation of the excess energy to the surroundings. This process, sometimes referred to as phonon-assisted energy transfer (PAEnT, Figure 1c), was first proposed by Crosby and Kasha^27^ to explain Yb(III) sensitization with UV-absorbing chromophores. The competition between these two pathways was also proposed to be responsible for ^1^O_2_ generation by a pyrene-sensitized Yb(III) chelate.^28^

An understanding of how to tune, boost, or deactivate one or several sensitization pathways, and how the modification of one sensitization pathway may affect the others as well as Yb(III) luminescence are crucial for designing bright emitters and responsive biological probes. Such probes often rely on the turning on or off of luminescence (or sensitization) by physicochemical interactions with biological materials.^3,29−36^ However, there are currently no systematic studies on the structural requirements that allow PeT or PAEnT to operate.

Yb(III) sensitization via PeT depends on the oxidation and reduction potentials of the antenna and the metal center, respectively. We hypothesized that modulating the coordination environment of the complex while keeping the antenna constant would change the Yb(III)/Yb(II) redox potential and provide control over the electron transfer without altering the electronic and photophysical properties of the antenna (Figure 1b,c). PeT from an excited carbostyril to Eu(III) could be controlled by tuning the Eu(III)/Eu(II) redox potential via the ligand.^37,38^

**Chart 1:**
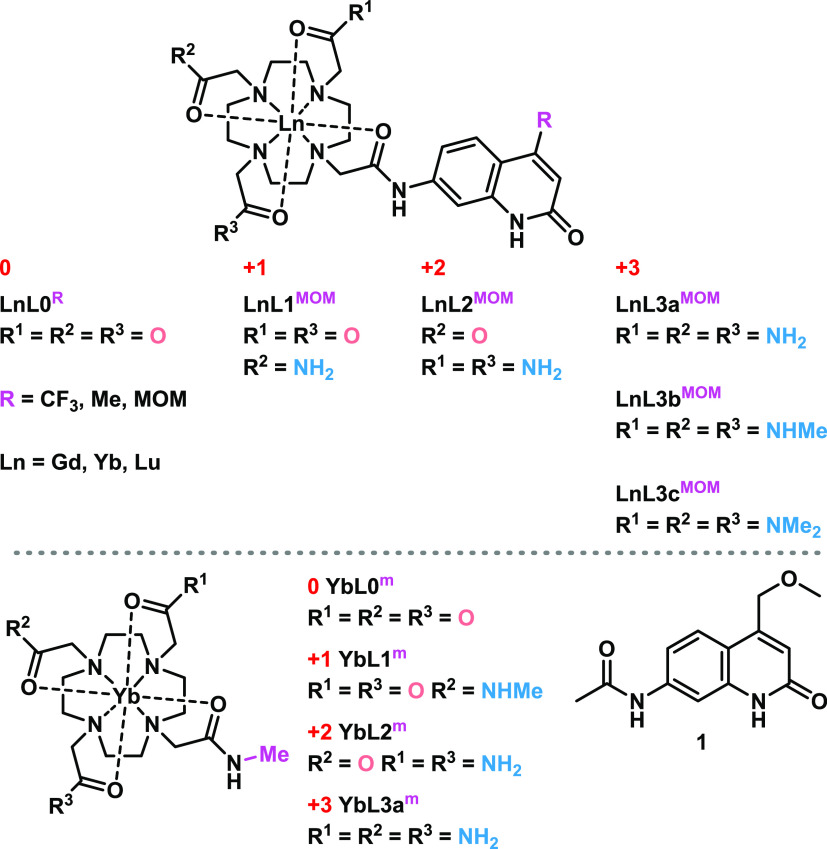
Gd(III), Yb(III), and Lu(III) Complexes (Top) and Model Compounds (Bottom) Studied Herea

Here, we investigated how the redox properties of Yb(III) impact the photophysical properties of the emitter. We report the synthesis and characterization of a series of gradually more reducible Yb(III) complexes (Chart 1) with 0, +1, +2, and +3 overall charge. Increasing amounts of coordinating amide donors were introduced in a series of 1,4,7,10-tetraazacyclododecane (cyclen)-based ligands by replacing one, two, or three carboxylate groups. The redox properties of two types of model compounds were also studied: Yb complexes without antenna (**YbL**^**m**^) and the acetylated 4-methoxymethyl (MOM)-substituted carbostyril (**1**). These models enabled the investigation of metal and antenna fragments separately without interference from other functional units.

a Complex names indicate overall charge (0, +1, +2, +3) and the antenna type (CF_3_ = trifluoromethyl, Me = methyl, MOM = methoxymethyl, m = model). The syntheses of **LnL0-3**^**MOM**^, **LnL0**^**CF3**^, **LnL0**^**Me**^, and **1** have previously been reported,^23,37,39^ and those of **YbL0-3**^**m**^ are detailed in the Supporting Information.

Three versions of the +3 charged triamide complex were prepared to allow the assessment of secondary effects caused by the increasing number of N–H oscillators.^40^ Three different carbostyril antennae bearing methyl (Me), CF_3_, or MOM auxochromes in the 4 position of the carbostyril were also tested on neutral tricarboxylate ligands to evaluate the effect that the sensitizer might have on the Yb(III) photophysical properties.

The structures of the complexes were studied by paramagnetic ^1^H NMR spectroscopy and X-ray crystallography to confirm that the increasing overall charge did not induce major structural changes. The Yb(III)/Yb(II) reduction potentials of the corresponding complexes were measured by cyclic voltammetry to quantify the change in metal center reducibility upon charge variation. The photophysical properties were determined using absorption and luminescence spectroscopy in the UV−vis and NIR. The effects of all of the structural variations on the antenna and Yb(III) emission were analyzed in light of the hypothetical PeT mechanism that is believed to be involved in the deactivation of the antenna and the Ln(III) sensitization. While PeT is often invoked as the major or even only sensitization pathway in Yb(III) emitters, in this set of complexes, we found that PeT contributed little to Yb(III) luminescence sensitization, even when the process was thermodynamically feasible. Furthermore, a larger PeT rate constant improved the Yb(III) luminescence quantum yield only marginally.

## Results and Discussion

### Solution and Solid-State Structures

Ligands were synthesized following literature procedures, and the Yb(III) and Lu(III) complexes were prepared as previously described for Gd(III), Eu(III), and Tb(III).^37,39^ To ascertain that the modification of the coordination site and resulting charge do not significantly change the structure of the complexes in solution and in solid state, the Yb(III) compounds were analyzed by paramagnetic ^1^H NMR spectroscopy and X-ray crystallography, respectively.

**Figure 2:**
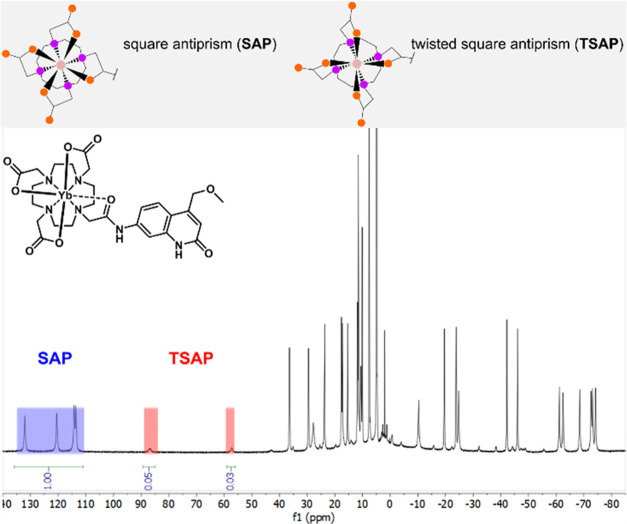
^1^H NMR spectrum (600 MHz) of **YbL0**^**MOM**^ in D_2_O at r.t. with the highlighted regions corresponding to TSAP (red) and SAP (blue) cyclen ring protons, respectively.

The ^1^H NMR spectra of Yb(III) complexes often enable the observation of individual well-resolved signals for each magnetically unique proton.^41^ We assigned the most deshielded chemical shifts in the ^1^H spectra (400 MHz, D_2_O, r.t.) of **YbL** to the axial protons of the cyclen ring in the square-antiprismatic (SAP) isomers (Figures 2 and S7–S21).^41−48^ A thorough characterization of **YbL0**^**MOM**^ (^1^H and ^13^C NMR spectra, COSY, EXSY, and HSQC experiments) can be found in the ESI (Figures S28–S41). The SAP-related ^1^H signals of the tricarboxylate complexes resonated at higher frequencies (111.63–133.79 ppm) than the ones of the corresponding amide-substituted species (92.07–116.55 ppm). **YbL0** were present as mixtures of SAP and twisted SAP (TSAP) conformers, and the protons of the latter resonated at 82.64–84.90 and 54.38–57.18 ppm (Figures 2 and S7–S10). The ratios of SAP:TSAP for **YbL0**^**CF3,Me,MOM**^ were 1:0.05, 1:0.09, and 1:0.06, respectively, while for the model **YbL0**^**m**^ complex, the proportion of the TSAP isomer was slightly larger (1:0.10). The latter might be explained by the increased flexibility of the amide pendant arm mimicking the absent antenna, as four signals were present in the TSAP region (69.45–81.59 ppm, Figure S17). Nevertheless, the contribution of the TSAP isomer is minor in all **YbL0** complexes and is missing in the positively charged antenna-appended Yb(III) compounds. The ^1^H spectrum of **YbL1**^**m**^ contained half as many signals as the other complexes due to the higher symmetry of this molecule (Figure S18).

Based on ^1^H NMR spectroscopy, all charged Yb(III) compounds with antennae were present as single species in solution, and neutral **YbL0** complexes had the same structure of their predominant isomers as the single conformers of amide analogues. Hence, the examined electrochemical and photophysical properties of Yb(III) complexes (vide infra) were accounted for from either only SAP isomers (charged species) or the weighted average of SAP (major) and TSAP (minor) conformers (neutral molecules).

**Figure 3:**
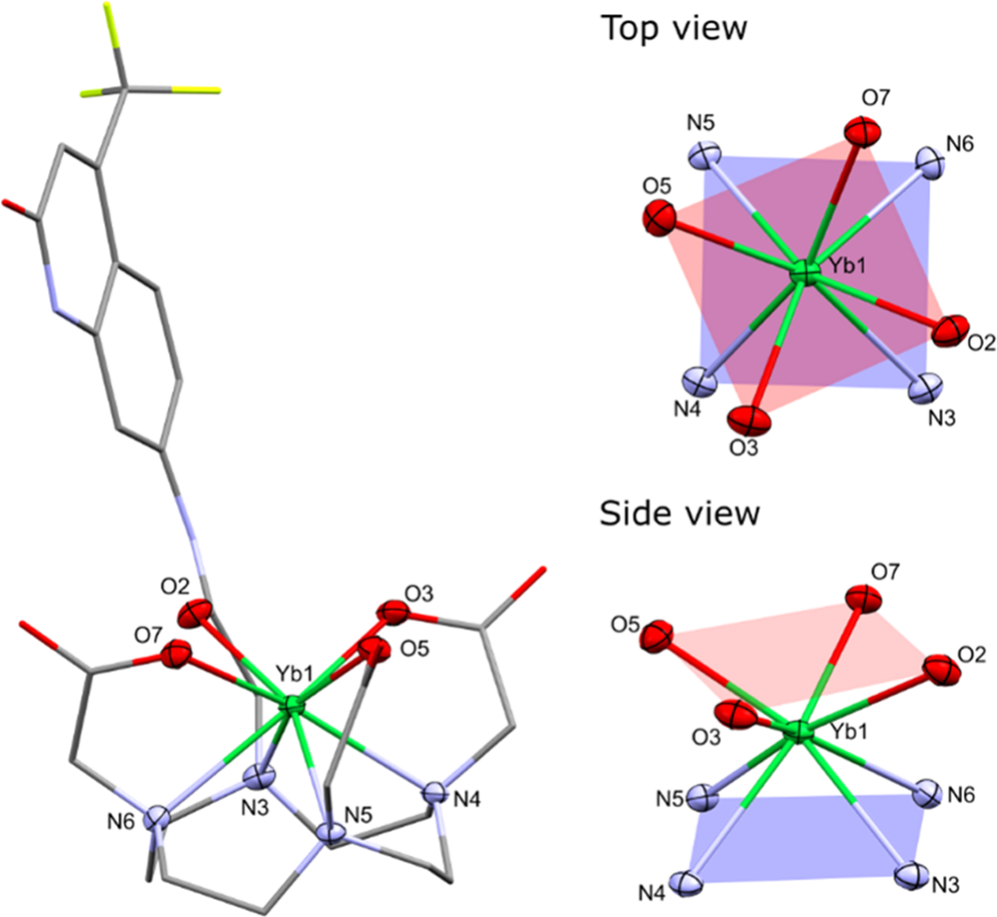
X-ray crystal structure (left) and the coordination environment of Yb (right, view from the top and view from the side of the polyhedron) in **YbL0**^**CF3**^. The H atoms, water, and dioxane molecules were omitted for clarity. Yb(III) coordination environment is depicted as ellipsoids displayed at 50% probability, and the remaining atoms are shown as capped sticks.

Crystals suitable for X-ray diffraction analysis were obtained by vapor diffusion of dioxane into a concentrated aqueous solution of **YbL0**^**CF3**^. In the solid state, the square-antiprismatic Yb(III) center in **YbL0**^**CF3**^ is not capped (Figure 3), unlike the Ln(III) in previously reported complexes (Ln = Eu, Gd, Tb) employing similar carbostyril-substituted cyclen ligands.^37^ The eight-coordinate Yb center sits between two planes formed by four cyclen N-atoms (4N_PL_) and three tethered carboxylate and one amide O-atoms (4O_PL_). The 4N_PL_ and 4O_PL_ planes are near-parallel, with a 4N_PL_-Yb-4O_PL_ angle of 175°, although slightly more distorted than in previously reported examples (range: 176–178°). The distance of the Yb(III) ion to the 4O_PL_ and 4N_PL_ (1.0701(7) and 1.4522(8) Å, respectively) differ significantly from those of the related Ln(III) complexes (Ln = Eu, Gd, Tb; range: Ln–4O_PL_, 0.640(2)–0.736(2) Å; Ln–4N_PL_, 1.595(2)–1.6982(9) Å), which is consistent with the decreased Yb(III) ionic radius (for coordination number (CN) of 8: Yb^3+^ 0.99 Å vs Eu^3+^ 1.07 Å, Gd^3+^ 1.05 Å, and Tb^3+^ 1.04 Å)^49^ allowing the metal ion to sit almost equidistant to the planes. Similar to the Yb(III) complexes, the bond metrics for **YbL0**^**CF3**^ are relatively shorter when considering other Ln(III) carbostyril cyclen complexes.^50^ Carboxylate Yb–O distances range 2.2344(14)–2.2800(14) Å, the longer antenna amide Yb–O distance is 2.3354(14) Å, and the Yb–N distances range 2.4929(16)–2.5456(17) Å (Table S2).

The complex is racemic in the solid state, with both Δ and Λ isomers present in the unit cell (Figure S42).^51^ These isomers correspond to the TSAP geometry due to identical signs of NCCN and NCCO torsional angles (±56.8° and ±14.2°, respectively) in two separate molecules.^52^ The pair of positive torsional angles makes up a Λ(λλλλ) structure, while the pair of negative ones yields Δ(δδδδ). It is a rare example of a crystallographically characterized Yb(III) complex with only TSAP isomers, as normally the SAP conformation is predominant in the solid state.^53^ This could be connected with the asymmetrical ligand structure or co-crystallization of dioxane molecules. However, the solution data suggest that once dissolved, all of the **YbL** complexes adopt the same square antiprism geometry. Therefore, we can confirm that changes in photophysical properties cannot be due to variations in CN, geometry, or conformation.

### Electrochemistry

The redox properties of the Yb(III) complexes and model compounds were determined by cyclic voltammetry. Redox events were assigned with the help of models for the chelated Yb(III) centers (**YbL**^**m**^, Chart 1) and the amide-linked MOM antenna (**1**). Analyses were performed at 0.1 V/s scan rate in DMF containing 0.1 M (*n*-Bu)_4_NClO_4_ as the electrolyte. DMF has a more suitable solvent window than water to study Yb(III) reduction (Figure S44). Voltammograms were recorded by scanning first toward more negative potential values (reduction). A glassy carbon electrode and Ag/Ag^+^ reference electrode (0.01 M AgNO_3_ in acetonitrile) were used, and a ferrocene internal reference was added at the end of the experiment. The anodic and cathodic peak potentials (*E*_pa_, *E*_pc_), apparent reduction potential (*E*_1/2_), and peak potential separation (Δ*E*_p_) values vs *F*_c_/*F*_c_^+^ and vs NHE are reported in Table S3. Unless mentioned otherwise, all *E* values are reported vs *F*_c_/*F*_c_^+^.

**Figure 4:**
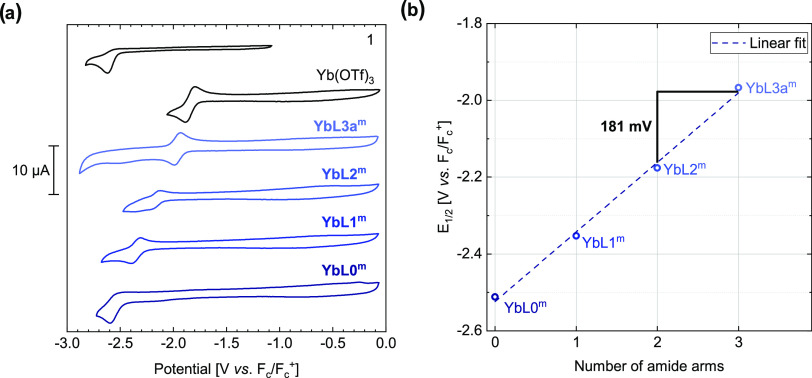
(a) Cyclic voltammograms of the reference compounds; (b) variation of apparent reduction potential vs number of amide arms. [**YbL**] = 0.5 mM in DMF (0.1 M (*n*-Bu)_4_NClO_4_) in a glovebox. Scan rate, 0.1 V/s; working electrode, glassy carbon (⌀ 3 mm); reference electrode, Ag/Ag^+^ (10 mM AgNO_3_ in acetonitrile); counter electrode, Pt wire. Data represent the average of three consecutive scans.

The cyclic voltammograms of Yb(OTf)_3_ and **YbL0–3a**^**m**^ display a single wave that can be attributed to the Yb(III)/Yb(II) redox couple (Figure 4a). Electrochemical studies of Yb(OTf)_3_ and the model compounds at different scan rates show that the peak potential values shift only slightly with increasing scan rate (Figures S45–S57, Tables S4–S9), suggesting that these are electrochemically reversible systems. The apparent Yb(III)/Yb(II) reduction potential (*E*_1/2_) of **YbL0–3a**^**m**^ increases linearly from −2.51 V for **YbL0**^**m**^ to −1.97 V vs for **YbL3a**^**m**^, which corresponds to an increase of ∼181 mV per carboxylate arm replaced by an amide arm (Figure 4b). The apparent reduction potential of Yb(OTf)_3_ measured under the same conditions is still larger (−1.84 V), which shows that the functionalized cyclen-based ligand better stabilizes the Yb(III) oxidation state. The less negative reduction potential of Yb(OTf)_3_ compared to **YbL3a**^**m**^ can be explained by the replacement of weakly coordinating triflate anions by neutral solvent molecules (CN = 8 in DMF).^54^ Irreversible reduction of the acetylated MOM-antenna (**1**) was observed at *E*_pc_ = −2.62 V (Figure 4a). The oxidation of **1** under these conditions ([**1**] = 0.5 mM in DMF with 0.1 M (*n*-Bu)_4_NClO_4_ as the electrolyte) could not be accomplished, as it takes place at a potential outside the solvent window (>0.5 V vs *F*_c_/*F*_c_^+^). In acetonitrile **1** could be oxidized at *E*_pa_ = 1.76 V vs NHE.^39^

The cyclic voltammograms of **YbL**^**MOM**^ display similar trends as **YbL**^**m**^ with some small differences. Analogously to the observations made for **YbL0–3a**^**m**^, an increasing overall positive charge of the complexes yields more positive *E*_pc_ values (Figure S45). **YbL0**^**MOM**^ and **YbL1**^**MOM**^ display a large reduction wave with no return oxidation wave, and the height of the wave is approximately twice the one observed for the other complexes (Figure S45) suggesting an irreversible 2 e^–^-reduction. The cyclic voltammograms of **YbL2**^**MOM**^ and **YbL3a–c**^**MOM**^ display a single reduction wave with a corresponding return oxidation wave. This redox event was assigned to the one-electron Yb(III) reduction–oxidation (Table S3). **YbL3a–c**^**MOM**^ were further studied at different scan rates. *E*_pa_ and *E*_pc_ were independent of scan rate, and Δ*E*_p_ was close to 60 mV, indicating that these systems are electrochemically reversible (Figures S58–S63, Tables S10–S12). Amide methylation leads to a slightly more negative apparent reduction potential for **YbL3c**^**MOM**^ (*E*_1/2_ = −1.95 V) than for **YbL3a**^**MOM**^ (*E*_1/2_ = −1.91 V), similarly to what was seen in the Eu complexes of the same ligands.^37^

The solvent effect was estimated from the behavior of the Eu(III)/Eu(II) couple, which has been studied extensively. In water/DMF mixtures, increasing the proportion of DMF shifts *E*_1/2_(Eu(III)/Eu(II)) to more negative potentials by ∼146 mV.^55^ In another study, in the more electron-donating DMF^56^ the formal potential of Eu(III/II) was ∼332 mV more negative than in water, and that of Yb(III/II) was similarly affected.^57^ Complexation by cryptates diminished the influence of the solvent 2- to 3-fold. Thus, it is reasonable to assume that *E*_1/2_(Yb(III)/Yb(II)) of **YbL** in water is less negative than the values obtained in DMF, making Yb(III) more reducible. Complexation by **L0**–**L3** is expected to diminish the influence of the solvent. The solvent effect may also impact the antenna. The electron-donating ability of the solvent is expected to (de)stabilize the (reduced) oxidized antenna.^58^ This is what we observed, as the reduction of **1** in acetonitrile happens at *E*_pc_ ≈ −1.88 V vs NHE,^39^ which is less negative than the value measured in DMF (*E*_pc_ = −2.22 V vs NHE, Table S3). Hence, we can assume that the oxidation potential of **1** in water may be more negative than the one obtained in acetonitrile (1.76 V vs NHE^39^).

**YbL3a–c**^**MOM**^ are electrochemically reversible systems, as shown by the scan rate-independent *E*_p_. Electron transfer is fast, Ln redox state change does not cause either substantial reorganization or demetallation. In addition, these ligands can accommodate, and have a good affinity for both Yb(II) and Yb(III). The trend in the redox potentials confirms our original hypothesis that increasing the overall positive charge on the complexed Yb(III) would make it more willing to accept an electron. Increasing the overall positive charge has a more pronounced effect on Yb(III) than on Eu(III), shifting *E*_1/2_ by 181 vs 95 mV per charge on average, respectively.^37^ The reason for the larger shift may be the smaller size and harder Lewis acidity of Yb(III), which results in larger stabilization differences of the Ln(II) and Ln(III) complexes with soft amide donors than in the case of the larger, softer Lewis acid Eu(III). An alternative explanation could be the change of geometry or CN from 8 in **YbL** to 9 in **EuL**.^37^

### Photophysical Properties

**Figure 5:**
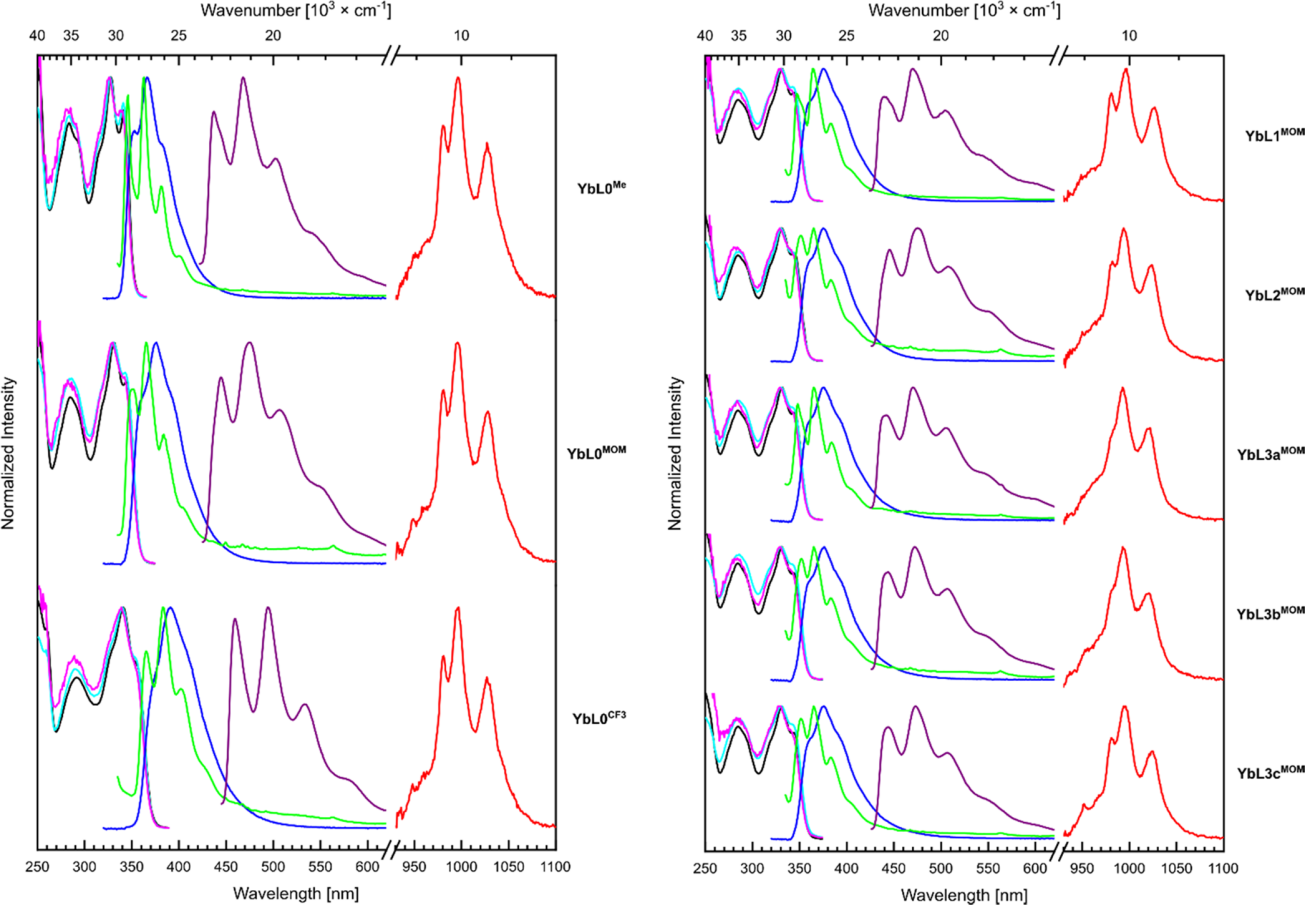
Normalized absorption (black) and normalized excitation spectra of the **YbL** complexes for the antennae (λ_em_ = 385/405/415 nm, cyan) and for the Yb(III) emission (λ_em_ = 997 nm, magenta). Steady-state emission spectra at r.t. of the antennae (λ_ex_ = 329 nm, blue) and of the Yb(III) (λ_ex_ = 323 nm, red). Steady-state emission spectra of **YbL** at 77 K (λ_ex_ = 329 nm, green) and phosphorescence spectra of **GdL** at 77 K (λ_ex_ = 344 nm, purple). Measured in 10 mM PIPES in H_2_O, pH 6.5, absorbance set at *A* = 0.1.

The photophysical properties of the complexes were measured in 10 mM PIPES-buffered aqueous solutions at pH = 6.5. These conditions were chosen to avoid the deprotonation of the CF_3_-substituted antenna.^23^ The characterization was done at room temperature by UV–vis absorption spectrophotometry and luminescence spectroscopy in the UV–vis and NIR ranges, where the antenna and Yb(III) emit, respectively (Figures S65–S80). UV–vis absorption was carried out first to set the solutions at the same absorbance. This ensured that all of the complexes absorbed the same amount of light at the excitation wavelength used for luminescence spectroscopy. Steady-state emission and excitation spectra were obtained both for the fluorescence of the antenna and the luminescence of the Yb(III) center (Figure 5). Low-temperature emission spectra were recorded with frozen solutions at 77 K to locate the antenna T_1_. The quantum yields of the residual antenna fluorescence were calculated from the emission spectra relative to the emission of a solution of quinine sulfate (Tables 1 and S13–S15). Only relative quantum yields could be calculated for the Yb emissions because no reference compound with a NIR emission and an excitation range in the same domain as our complexes could be found. The lifetimes of the antenna were measured in the nanosecond domain under picosecond pulsed UV excitation (Tables 2 and S18–S21). Yb(III) emission lifetimes were too short to be measured with the NIR detector and the nanosecond instrument was not sensitive enough in the NIR to measure Yb luminescence.

**Table 1:** Antenna Fluorescence Quantum Yields Φ_L_ and Relative Quantum Yields φ_L_ of **LnL** (Ln = Yb, Gd, Lu) in H_2_O^a^

YbL	Φ_L_ (φ_L_) [%]^b^^,^^c^	GdL	Φ_L_ (φ_L_) [%]^b^^,^^d^	LuL	Φ_L_ (φ_L_) [%]^b^
**L0**^**MOM**^	5.9 (100)	**L0**^**MOM**^	7.2 (100)	**L0**^**MOM**^	8.3 (100)
**L1**^**MOM**^	5.4 (93)	**L1**^**MOM**^	7.0 (97)	**L1**^**MOM**^	8.4 (101)
**L2**^**MOM**^	4.3 (74)	**L2**^**MOM**^	6.8 (95)	**L2**^**MOM**^	8.0 (96)
**L3a**^**MOM**^	3.1 (53)	**L3a**^**MOM**^	6.5 (91)	**L3a**^**MOM**^	7.7 (93)
**L3b**^**MOM**^	3.5 (59)	**L3b**^**MOM**^	6.4 (88)	**L3b**^**MOM**^	7.6 (92)
**L3c**^**MOM**^	4.4 (74)	**L3c**^**MOM**^	6.5 (90)	**L3c**^**MOM**^	7.2 (86)
**L0**^**CF3**^	4.8 (81)				
**L0**^**Me**^	4.2 (72)				

a Measurements were performed in 10 mM aqueous PIPES buffer solutions at pH 6.5, [**LnL**] = 10 μM.

b Φ_L_ determined relative to quinine sulfate (Φ = 0.59) in H_2_SO_4_ (0.05 M) in H_2_O;^62^ φ_L_ compared to **LnL0**^**MOM**^.

c Mean for two or three (**YbL0**^**MOM/Me**^**, LuL0–3b**^**MOM**^) independent measurements.

d From ref (37).

**Table 2:** Measured Lifetimes (τ_f,L_) of **LnL**^**MOM**^ (Ln = Yb, Lu) Antenna Fluorescence in H_2_O (λ_ex_ = 341.5 nm, λ_em_ = 377 nm, Monoexponential Reconvolution Fit), and Calculated τ_rad,L_, *k*_rad,L_, *k*_f,L_ (s^–1^), *k*_nr,L_, and *k*_PeT_^a^

compound	τ_f,L_ [ns]^b^	τ_rad,L_ [ns]	*k*_f,L_ [ns^–1^]	*k*_rad,L_ [ns^–1^]	*k*_nr,L_ [ns^–1^]	*k*_PeT_ [ns^–1^]
**LuL0**^**MOM**^	0.47	5.64	2.14	0.18	1.96	n/a
**LuL1**^**MOM**^	0.47	5.56	2.14	0.18	1.96	n/a
**LuL2**^**MOM**^	0.46	5.76	2.18	0.17	2.01	n/a
**LuL3a**^**MOM**^	0.45	5.81	2.24	0.17	2.07	n/a
**LuL3b**^**MOM**^	0.45	5.86	2.24	0.17	2.07	n/a
**LuL3c**^**MOM**^	0.43	6.01	2.33	0.17	2.16	n/a
**YbL0**^**MOM**^	0.34	5.84	2.92	0.17	2.74	0.8
**YbL1**^**MOM**^	0.34	6.26	2.94	0.16	2.78	0.8
**YbL2**^**MOM**^	0.25	5.87	3.95	0.17	3.78	1.8
**YbL3a**^**MOM**^	0.26	8.53	3.82	0.12	3.70	1.6
**YbL3b**^**MOM**^	0.23	6.53	4.42	0.15	4.27	2.2
**YbL3c**^**MOM**^	0.28	6.44	3.57	0.16	3.42	1.3

a Measurements were performed in 10 mM aqueous PIPES buffer solutions at pH 6.5, [**LnL**^**MOM**^] = 10 μM. *k*_f,L_, *τ*_rad,L_, *k*_rad,L_, *k*_nr,L_, and *k*_PeT_ were calculated as follows: *k*_f,L_ = 1/τ_f,L_, τ_rad,L_ = τ_f,L_/Φ_L_, *k*_rad,L_ = 1/τ_rad,L_, *k*_nr,L_ = *k*_f,L_ – *k*_rad,L_, *k*_PeT_ ≈ *k*_nr,L_(Yb) – *k*_nr,L_(Lu).

b Mean for two (**LuL0–3b**^**MOM**^, **YbL**^**MOM**^) independent measurements.

### Ligand-Centered Photophysics

Φ_L_ and φ_L_ are the absolute and relative antenna fluorescence quantum yields, respectively. The photophysical properties of the antenna in the Yb(III) complexes were compared to those of their Gd(III) and Lu(III) analogues. Gd(III) has only high-energy excited states (32 200 cm^–1^),^21^ and is the most difficult to reduce Ln(III) ions (*E*_1/2_ = −3.9 V).^59^ Lu(III) similarly lacks both photo- and redox activities.^59,60^ Lu(III) and Yb(III) have similar ionic radii (CN = 8, 0.98, vs 0.99 Å, respectively);^49^ therefore, Lu and Yb complexes are expected to have similar geometries and charge densities. The comparison of **YbL**^**MOM**^ with **GdL**^**MOM**^ and **LuL**^**MOM**^ thus enables studying the effect of the photoactive Yb(III) center on the photophysical properties of the antenna within a similar environment that reproduces the heavy atom effect of the Ln.

Every complex carrying the MOM-substituted antenna (**YbL0–3**^**MOM**^) had superimposable absorption spectra between 310 and 400 nm (Figure S81). This absorption band corresponds to the S_1_ ← S_0_ π–π* transition of the carbostyril chromophore. The local absorption maximum in this band is at λ_max_ = 330–331 nm between two shoulders located at 318–319 and 343–344 nm. The same transitions were observed for the analogous **LuL0–3**^**MOM**^ (Figure S82) and other previously studied **LnL**^**MOM**^,^37^ indicating that the nature of the Ln(III), the coordination site, and the overall charge have little to no effect on the antenna absorption.

When excited in the antenna absorption band, the Yb(III) complexes showed Yb(III) luminescence of different intensities (vide infra), along with residual antenna fluorescence (Figures 5 and S84–S91). As seen by inspecting the complexes with the CF_3_-, Me-, and MOM-substituted antenna, electron-withdrawing auxochromes (CF_3_) on the carbostyril red-shift the absorption and fluorescence spectra (Figures S83 and S86). The emission spectra of ligands carrying the same antenna but different Ln(III) centers or different coordination sites have the same shape (Figures S85, S87, and S88).^23^

When cooled to 77 K, the frozen solutions show, as expected, emission and excitation spectra with sharper bands and a more visible vibronic structure. The emission of the **GdL0–3**^**MOM**^ and **LuL0–3**^**MOM**^ complexes displayed fluorescence between 340 and 430 nm (S_1_ → S_0_ transition), as well as an extra band between 450 and 700 nm that is attributed to phosphorescence from the triplet state (T_1_ → S_0_ transition). This phosphorescent band is absent from the **YbL0–3**^**MOM**^ spectra, which suggests either that the triplet is not formed in these complexes, or that it is quickly quenched (Figure S92). Since the only difference between the complexes is the identity of the Ln(III), this means that Yb(III) rapidly quenches the T_1_ state. The same effect was observed with the other antennae, **LnL0**^**CF3**^ and **LnL0**^**Me**^, which show red- and blue-shifted fluorescence and phosphorescence, respectively, in the Gd complexes upon addition of electron-withdrawing and -donating auxochromes, but no phosphorescence within the corresponding Yb complexes (Figure S93).

The fluorescence quantum yields of the antenna (Φ_L_) in the **YbL**^**MOM**^ complexes, measured at room temperature, gradually dropped from 5.9(5)% for the charge-neutral **YbL0**^**MOM**^ to 3.1(3)% for the +3 charged **YbL3a**^**MOM**^ (Table 1), a decrease of 47%. In **LuL0–3a**^**MOM**^, **GdL0–3a**^**MOM**^, and **TbL0–3a**^**MOM**^,^37^ Φ_L_ decreased only by ∼3% with each extra positive charge, which is 7-fold smaller than what was seen for **YbL0–3a**^**MOM**^. The replacement of primary amide donors with secondary and tertiary ones increases Φ_L_ for **YbL3a–c**^**MOM**^ from 3.1(3) to 4.4(4)%. The measurement of the **Yb,LuL**^**MOM**^ quantum yields in PIPES-buffered D_2_O (pD = 6.9)^61^ did not show any significant variation of Φ_L_ that remained within 10% experimental error to the values measured in aqueous buffer solution (Table S14). This last result suggests that the antenna excited state is insensitive to quenching by the O–H vibrations of the solvent.

The fluorescence lifetimes (τ_f,L_) of the antennae in **YbL0–3**^**MOM**^ and **LuL0–3**^**MOM**^ were determined by measuring their fluorescence decays in the nanosecond range with an excitation wavelength at 341.5 nm. The decays were monoexponential and yielded τ_f,L_ from 0.23 to 0.34 ns for **YbL0–3**^**MOM**^ and from 0.43 and 0.47 ns for **LuL0–3**^**MOM**^ (Tables 2 and S18–S21). Using τ_f,L_ and Φ_L_, the radiative rate constants of fluorescence (*k*_rad,L_ = 1/τ_rad,L_) were calculated according to (eq 1). The rate of nonradiative antenna decay (*k*_nr,L_) was then estimated as the difference between the measured rate constant of fluorescence decay (*k*_f,L_ = 1/τ_f,L_) and the calculated *k*_rad,L_.
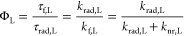
1Within the **LuL**^**MOM**^ series, *k*_rad,L_ and *k*_nr,L_ remain constant. Within the **YbL**^**MOM**^ series, the *k*_nr,L_ values are 1.4- to 2.1-fold larger than in the Lu(III) series. Furthermore, the nonradiative deactivation is not constant anymore and seems to increase with increasing positive charge (**YbL0–3a**^**MOM**^). When measured in PIPES-buffered D_2_O solutions, the resulting *k*_nr,L_’s are similar to the values in H_2_O but show a clearer increase in the range of 3.01–4.44 ns^–1^ upon going from **YbL0**^**MOM**^ to **YbL3a**^**MOM**^ (Table S15). These results are consistent with a faster nonradiative deactivation of the antenna S_1_ in the Yb(III) complexes that increases when increasing the overall charge of the complex.

The only major difference between the Yb(III) and Lu(III) complexes is the accessibility of the Yb(III) center to PeT or EnT. We may assume that (1) direct EnT from S_1_ to Yb(III) is unlikely due to the negligible spectral overlap between the S_1_ → S_0_ transition of the antenna and the ^2^F_5/2_ ← ^2^F_7/2_ transition of Yb(III), and (2) all other nonradiative deactivations are similar in the Yb(III) complexes and their Lu(III) analogues. The latter is supported by the similar lifetimes in D_2_O and by the 77 K spectra that show similar S_1_/T_1_ peak ratios upon variation of the coordination site (Table S16), which is consistent with comparable intersystem crossing (ISC). Therefore, the observed difference in nonradiative deactivation rate constant ought to come from PeT. The difference between *k*_nr,L_(Yb) and *k*_nr,L_(Lu) should thus give a good estimate of the rate constant of PeT (*k*_PeT_ in Table 2).

In charge-neutral **YbL0**^**MOM**^, *k*_PeT_ is over 3.5 times smaller than that of *k*_nr,L_ and *k*_f,L_. The largest PeT rate constant was seen in the +3 charged species **YbL3b**^**MOM**^, in which PeT represents 50% of the overall relaxation rate constant. The significant drop in *k*_PeT_ in dimethylated **YbL3c**^**MOM**^ compared to the monomethylated **YbL3b**^**MOM**^ can be explained by the more negative *E*_1/2_ of **YbL3c**^**MOM**^ (−1.95 V) than of **YbL3b**^**MOM**^ (−1.91 V).

To understand the effect of solvent on **LnL** photophysical properties, a limited study was carried out in DMF (Table S17, Figures S94–S100), the solvent used for cyclic voltammetry experiments. In DMF, the UV–vis absorption spectra were sensitive to the overall charge of the complex. Complexes had red-shifted shoulders in their absorption bands and smaller Φ_L_ compared to that in water. The **YbL** and **GdL** absorption spectra were nonsuperimposable despite carrying the same antenna. The coordination site dependence of the absorptions indicates that in DMF, the antenna photophysics are substantially affected by the metal binding site, which would complicate the analysis of the Yb(III) sensitization. Therefore, and as aqueous solutions are more relevant for biological applications, subsequent studies were done in water.

### Metal-Based Photophysics

Φ_Yb_^L^ and φ_Ln_ are absolute and relative Yb(III) luminescence quantum yields, respectively. Next, the Yb(III)-centered emission was studied. Upon excitation of the antenna at 323 nm, Yb(III) emission consisting of three peaks centered at 980, 996, and 1024 nm (Figure 5) was observed for every complex. These peaks all belong to the ^2^F_5/2_ → ^2^F_7/2_ transition of Yb(III). The shapes of the emission spectra were compared by normalizing them relative to the integral intensity of the emission bands.

The Yb(III) emission shape is antenna-independent, which suggests that the coordination environments of the various complexes are similar (Figure S90). Increasing the number of amides blue-shifts and intensifies the two low-energy transitions, whereas the highest-energy transition remains at the same energy but seems only to change in intensity (Figure S89).

The Yb(III) excitation spectra resemble the absorption spectra for the S_1_ ← S_0_ transition, proving that Yb(III) is sensitized by the excited antenna (Figure 5).

Relative Yb(III) luminescence quantum yields (φ_Ln_)^63−68^ were calculated from the integrated intensity of the Yb emission bands (*I*_Yb_) with **YbL3c**^**MOM**^ as the reference intensity (*I*_Yb,ref_) and (1 – *T*_ref_)/(1 – *T*) the absorptance correction factor (eq 2).
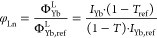
2**YbL3c**^**MOM**^ is the strongest emitter in the series (Table 3). The MOM-substituted antenna yields larger φ_Ln_ than the Me-substituted one, which in turn yields a larger φ_Ln_ than the CF_3_-substituted antenna. **YbL1–3b**^**MOM**^ on the other hand are all within 83 ± 2% of the quantum yield of **YbL3c**^**MOM**^ in buffered solution at r.t. The superior φ_Ln_ of **YbL3c**^**MOM**^ relative to the other complexes is significant, as it is 6 standard deviations larger than **YbL0**^**MOM**^, which is the second most luminescent complex in this series. **YbL3c**^**MOM**^ has improved φ_Ln_ compared to **YbL0**^**MOM**^. Notably, **YbL3c**^**MOM**^ was 1.14 times more emissive in H_2_O than the tricarboxylate analogue **YbL0**^**MOM**^ despite the fact that the number of X–H oscillators is identical for the two complexes.

**Table 3:** Measured Relative Yb(III) Luminescence Quantum Yields φ_Ln_ of **YbL**^b^

complex	φ_Ln_ [%], H_2_O^a^	φ_Ln_ [%], D_2_O^a^
**YbL0**^**MOM**^	88 ± 2	89 ± 6
**YbL1**^**MOM**^	81 ± 2	
**YbL2**^**MOM**^	83 ± 2	
**YbL3a**^**MOM**^	83 ± 2	100
**YbL3b**^**MOM**^	85 ± 2	
**YbL3c**^**MOM**^	100	
**YbL0**^**CF3**^	77 ± 1	
**YbL0**^**Me**^	82 ± 2	

a Measured in aqueous 10 mM PIPES, pH 6.5, upon excitation at 323 nm.

b Measured in 10 mM PIPES in D_2_O, pD 6.9, upon excitation at 331 nm.

In **YbL3b**^**MOM**^, even 1 NH per amide induces enough quenching so that φ_Ln_ is not significantly improved compared to the NH_2_ analogues. The improvement of φ_Ln_ upon NH oscillator removal (**YbL3c**^**MOM**^) is expected as only three NH vibrations (3000–3500 cm^–1^) are required to quench the Yb excited state (Figure 1c). In D_2_O solution, +3 charged **YbL3a**^**MOM**^ was 1.12 times more luminescent than charge-neutral **YbL0**^**MOM**^. Thus, in the absence of N–H quenching, the more reducible Yb(III) center was more emissive; however, this difference was not observable in H_2_O. Intriguingly, the uncertainty on the relative φ_Ln_ is larger in D_2_O than in H_2_O because the emission of **YbL3a**^**MOM**^ turned out to vary much more than **YbL0**^**MOM**^ in deuterated solution.

### Photoinduced Electron Transfer vs Phonon-Assisted Energy Transfer

Φ_Yb_^Yb^ and Φ_sens_ are Yb(III) intrinsic and sensitization quantum yields, respectively. In many sensitized Ln(III) emitters, the antenna T_1_ is considered the major feeding level.^12,69^ T_1_ in Me-, MOM-, and CF_3_-substituted carbostyrils are at 22 900, 22 500, and 21 800 cm^–1^, respectively.^23^ The spectral overlap between the antenna phosphorescence and the only Yb(III) excited spectroscopic level (10 260 cm^–1^) is negligible.^21^ Thus, EnT from S_1_ or T_1_ to Yb(III) is not happening directly by resonance EnT. In the following section, we discuss the contributions of the two potential sensitization mechanisms: PeT^22,24^ and PAEnT.^27,70^

The driving force for PeT from the excited carbostyril to the Yb(III) was calculated (eq S6 in the SI),^24,71^ and the values are listed in Tables 4 and S24.

**Table 4:** Yb(III)/Yb(II) Reduction Potential of **YbL**^**MOM**^ and Driving Force for PeT from the Excited Carbostyril (MOM)

complex	*E*_red_^YbL^ [V vs NHE]^a^	Δ*G*(PeT)[eV]^b^
**YbL0**^**MOM**^	–2.42	0.50
**YbL1**^**MOM**^	–1.92	0.00
**YbL2**^**MOM**^	–1.70	–0.23
**YbL3a**^**MOM**^	–1.55	–0.37
**YbL3b**^**MOM**^	–1.56	–0.36
**YbL3c**^**MOM**^	–1.60	–0.32

a Measured in DMF with 0.1 M NBu_4_ClO_4_ electrolyte; scan rate 0.1 V/s; GC electrode.

b Calculated using eq 3, with *E*_ox_ = 1.76 V vs NHE.^39^

Sensitization via T_1_ by PeT is endergonic (Δ*G*_PeT_ > +50 kJ/mol). PeT from S_1_ was found to be thermodynamically favored in most complexes, the most negative Δ*G*(eT) was calculated for the +3 charged ones. However, for **YbL0**^**MOM**^, PeT was clearly thermodynamically uphill (0.50 eV), while in **YbL1**^**MOM**^, Δ*G*(eT) was close to 0. Solvent effects on the Yb(III)/Yb(II) reduction potentials (obtained in DMF, vide supra) and the antenna oxidation potential (obtained in acetonitrile) mean that the complexes may be easier to reduce and the antenna easier to oxidize in water. However, unless the combined effect on the antenna and Yb(III) redox behavior is larger than +370 or −500 mV, at least one of the complexes in the series will have Δ*G* > 0 eV and one Δ*G* < 0 eV (Figure S128). The estimated PeT rate constants were at least twice as large for **YbL2–3c**^**MOM**^ as for **YbL0,1**^**MOM**^ (Table 2), suggesting that in **YbL0–1**^**MOM**^, PeT is slow and is not the major sensitizing pathway.

**Figure 6:**
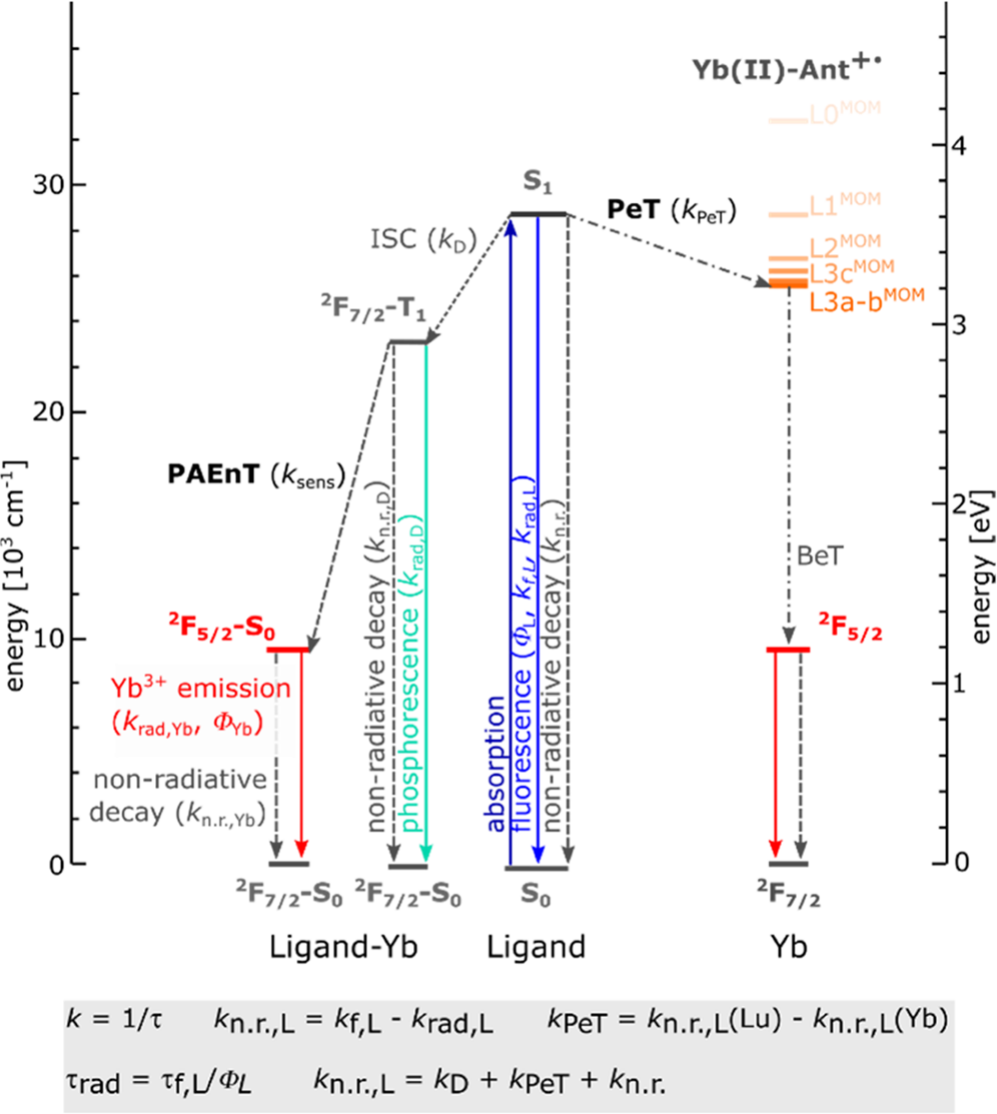
Proposed sensitization pathways for **YbL**.

PAEnT could explain that **YbL0–1**^**MOM**^ are nonetheless luminescent (Figure 6). In this model, the metal center and the ligand are not decoupled. PAEnT corresponds to the nonradiative relaxation of the (^2^F_7/2_–T_1_) state to the (^2^F_5/2_–S_0_) state with a rate that is proportional to the Franck–Condon factor, which was calculated using (eq 3)^70^
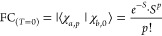
3where *S* is the Huang–Rhys factor (*S* = 2)^72,73^ and *p* is the reduced energy gap estimated in units of ℏω_Q_ between |*a*⟩ and |*b*⟩ υ = 0 levels.

The ratios of the calculated Franck–Condon factors for the nonradiative processes (^2^F_7/2_–T_1_) → (^2^F_5/2_–S_0_) and (^2^F_7/2_–T_1_) → (^2^F_7/2_–S_0_) suggest that in **YbL0–3**^**MOM**^ (^2^F_7/2_–T_1_) → (^2^F_5/2_–S_0_) are 8 orders of magnitude faster than the alternative nonradiative (^2^F_7/2_–T_1_) → (^2^F_7/2_–S_0_) relaxations (Table S23). Thus, energy transfer via (^2^F_7/2_–T_1_) → (^2^F_5/2_–S_0_) would dominate nonradiative relaxations. This agrees with the observation that in all **YbL**, T_1_ is quenched compared to **GdL** and **LuL** (Figures S92 and S93). If T_1_ is formed and then quenched by PAEnT, then T_1_ is quenched much faster than the radiative relaxation to S_0_. This is a reasonable assumption, as the phosphorescence radiative rate constant is typically very slow (∼ms^–1^–s^–1^).

The intermediacy of T_1_ in **YbL** is supported by the photostability data (Figures S126–S133). Under air, **YbL** are highly photostable and retain 95–96% of their antenna fluorescence after irradiation for 2 h, while under inert atmosphere (Ar or N_2_), up to 25% of the antenna fluorescence is lost. For **GdL**, the trends are similar, although the loss of antenna fluorescence is larger (95% under Ar). Decay is slower in D_2_O than in H_2_O (15 vs 22%, respectively, for **YbL3c**^**MOM**^), and in unbuffered solutions, the pH increases upon irradiation. These observations are consistent with the formation of T_1_ in both **GdL** and **YbL** and subsequent T_1_ oxidation by H^+^, which is thermodynamically feasible (eq S7). O_2_ rapidly quenches T_1_, which returns the ground-state antenna. In the absence of O_2_, T_1_ is long-lived and can degrade photochemically by reducing H^+^ (Figures S131, S133, S135, and S137). The slower antenna degradation in **YbL** than in **GdL** is consistent with excited state Yb(III) formation depleting T_1_.

The absolute quantum yield of Yb luminescence (Φ_Yb_^L^, eq 4) is a combination of the sensitization quantum yield (Φ_sens_) and intrinsic quantum yield of Yb emission (Φ_Yb_^Yb^).
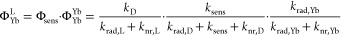
4The quenching of the Yb(III) excited state by NH vibrations is affecting Φ_Yb_^Yb^ by increasing the nonradiative relaxation rate constant (*k*_nr,Yb_) of the ^2^F_5/2_ spectroscopic level of Yb(III). Φ_sens_ is dependent on the rate constant of sensitization (*k*_sens_) from the donor level (D) relative to the other deactivations of the level, and the rate constant of formation of this level (*k*_D_) from the antenna S_1_ excited state. No significant differences in φ_Ln_ due to PeT are seen for these complexes in H_2_O due to N–H oscillators; in D_2_O, PeT made **YbL3a**^**MOM**^ 1.12 times as emissive as **YbL0**^**MOM**^ (Table 3, Figure S91). If PeT is not sensitizing, but only deactivates S_1_, any sensitizing lower-energy level (D) would also be depopulated by PeT, decreasing Φ_Yb_^L^. If PeT is sensitizing but another major process depending on a level less energetic than S_1_ dominates sensitization, increasing depopulation of the major sensitizing level by PeT would be compensated for by PeT sensitization. Therefore, the Yb(III) emission quantum yield would be left unaffected by PeT. This is consistent with mixed PeT + PAEnT sensitization limited mainly by Φ_Yb_^Yb^. PAEnT is certainly involved in Yb sensitization as Yb(III) emission is observed in complexes where PeT is not thermodynamically favorable (**YbL0**^**MOM**^ and **YbL1**^**MOM**^). The improvement of Yb emission suggests that PeT, despite being a minor sensitization pathway, might be more efficient than PAEnT. This makes sense because PeT is more direct than PAEnT, as the latter must go through ISC and then depends on the T_1_ decay rate constant.

## Conclusions

A series of Yb(III) complexes carrying carbostyril antennae and with structurally similar but electronically varied coordination environments were synthesized. The complex structures were found to be analogous in solution by paramagnetic ^1^H NMR spectroscopy, a finding also supported by the binding site-independent shapes of the antenna absorption as well as the shapes of the antenna fluorescence spectra; Yb(III) luminescence spectra varied only slightly. The overall more positively charged complexes contained more reducible Yb(III) centers, shifting *E*_red_ = −2.42 (V vs NHE) for uncharged **YbL0**^**MOM**^ to *E*_red_ = −1.55 (V vs NHE) for +3 charged **YbL3a**^**MOM**^, as determined by cyclic voltammetry. A single carboxylate-to-carboxamide substitution shifted the Yb(III)/Yb(II) reduction potential on average by 181 mV.

Fluorescence spectroscopy showed that **YbL** have smaller Φ_L_ than their **LuL** analogues, which supports that PeT is taking place in **YbL**. More positively charged species had increasing nonradiative deactivation of S_1_, consistent with a larger rate of PeT correlated to a less negative reduction potential of Yb(III).

Yb(III) luminescence was observed in all cases, even when the PeT process was not thermodynamically favored. Yb(III) emission quantum yields were particularly sensitive to the introduction of NH groups, as demonstrated by measurements in D_2_O. As such, the most efficient Yb(III) emitter of the series was the fully methylated amide complex, which benefits from the removal of NH quenching.

The analysis of a series of emitters can reveal trends that would be hidden when only a single emitter is studied. Here, sensitization pathways were studied for a systematically varied series of Yb complexes to help identify competing sensitization pathways and understand the structural features that promote them. The contribution of PeT to Yb(III) luminescence sensitization was small, while the alternative PAEnT sensitization pathway was calculated to dominate T_1_ nonradiative relaxation. The MOM-derivatized carbostyril antenna was better at sensitizing Yb(III) than the Me and CF_3_ analogues. PeT tuning by variation of the coordination site and overall charge is only marginally affecting the emission quantum yield of Yb(III). No drastic improvement of the Yb(III) luminescence quantum yield was observed when increasing the PeT rate constant, partly because it is probably not the only sensitizing process, and mainly because vibrational quenching is a more important parameter to control to give an opportunity for Yb to emit. Therefore, a direct correlation between an increase in PeT and an improved Yb(III) luminescence can only be expected when alternative sensitization pathways are insignificant. In these complexes, PeT was certainly observed to be *E*_red_-dependent, but its impact on the emission of Yb(III) is still unclear. A detailed study of the rise time and decay of the Yb(III) emission would be required to investigate the involvement of the T_1_ state.

## Supporting Information Available

Syntheses and characterization data of new compounds; crystallographic analysis of **YbL0**^**CF3**^; ^1^H, ^13^C, and ^19^F NMR spectra; cyclic voltammetry of **YbL**; and photophysical characterization of **LnL** (PDF)
