# Supplementary material for: Sensitization Pathways in NIR-Emitting Yb(III) Complexes Bearing 0, +1, +2, or +3 Charges
Source: J Am Chem Soc. 2022 Nov 8;144(46):21056–67. doi: 10.1021/jacs.2c05813 (PMC9706556; doi:10.1021/jacs.2c05813)
Supplement: Supplementary file 1 — ja2c05813_si_001.pdf [file ja2c05813_si_001.pdf]

Sensitization pathways in NIR-emitting Yb(III) complexes bearing 0, +1, +2, or +3 charges

Emilie Mathieu,[†,‡,a] Salauat R. Kiraev,[†,a] Daniel Kovacs,[†] Jordann A. L. Wells,[†] Monika Tomar,[†] Julien Andres,[*,§] K. Eszter Borbas[*,†]

[†] Department of Chemistry, Ångström Laboratory, Uppsala University, Box 523, 75120, Uppsala, Sweden, eszter.borbas@kemi.uu.se

[‡] Present address : Laboratoire de chimie de coordination du CNRS, Université de Toulouse, CNRS, 31077, Toulouse, France

[§] Chemistry and Chemical Engineering Section, Ecole polytechnique fédérale de Lausanne (EPFL), BCH 3311, CH-1015, Lausanne, Switzerland, julien.andres@epfl.ch

[a] E. M. and S. R. K. contributed equally

Materials and methods **S2**

General procedures S2

Chromatography S2

Spectroscopy S2

Near-infrared spectra S4

Paramagnetic ^{1}H NMR S5

Crystallography S5

Electrochemistry S5

Syntheses **S7**

Synthetic schemes S7

Synthetic procedures and characterization data S9

^{1}H NMR spectra of Lu(III) and Yb(III) complexes **S18**

^{1}H NMR spectra of Lu(III) complexes S18

^{1}H NMR spectra of Yb(III) complexes S21

Temperature dependent ^{1}H NMR spectra of Yb(III) complexes S29

NMR characterization of **YbL0^{MOM}** complex S32

X-ray crystallography **S40**

Electrochemical characterization **S43**

Photophysical characterization **S55**

Absorption, emission and excitation spectra S55

Fluorescence decay fits and residuals S80

Photostability experiments S94

Calculation of Franck-Condon factors **S100**

Driving force for photoinduced electron transfer **S101**

^{1}H, ^{13}C and ^{19}F NMR spectra **S102**

References **S120**

MATERIALS AND METHODS

General Procedures. ^{1}H NMR (400 MHz), ^{13}C NMR (101 MHz) and ^{19}F NMR (376 MHz) spectra were recorded on a JEOL 400 MHz instrument. Chemical shifts were referenced to residual solvent peaks and are given as follows: chemical shift (δ, ppm), multiplicity (s, singlet; br, broad; d, doublet, t, triplet; q, quartet; m, multiplet), coupling constant (Hz), integration. LC-MS analysis was carried out using an analytical Dionex UltiMate 3000 HPLC instrument coupled to a Thermo Finnigan LCQ DECA XP MAX mass spectrometer. HR-ESI-MS analyses were performed at the Organisch Chemisches Institut WWU Münster, Germany. All compounds displayed the expected isotope distribution pattern. Anhydrous CH_2Cl_2 was obtained by distillation from CaH_2 under an Ar atmosphere.

Compounds **1**,[1] **S1**,[2] **S3**,[3] **S4**,[4] **S5**,[4] **S6**,[3] **L0-3^{MOM}**,[4] **L0^{CF3}**,[4] and **YbL0^{Me}** (Ref 4) were synthesized following literature methods. All other chemicals were from commercial sources and used as received.

Chromatography. Preparative chromatography was carried out on silica gel [Normasil 60 chromatographic silica media (40–63 micron)] and Al oxide [activated, neutral, Brockmann Activity I, Sigma-Aldrich]. Thin layer chromatography was performed on silica-coated (60G F254) aluminum plates from Merck and Al oxide coated with 254 nm fluorescent indicator Al plates from Sigma-Aldrich. Samples were visualized by UV-light (254 and 365 nm) and permanganate stain.

HPLC-analysis was performed on a Dionex UltiMate 3000 system using a Phenomenex Gemini® C18 TMS end-capped 150 mm×4.6 mm HPLC column with water (0.05% formic acid): CH_3CN (0.05% formic acid) eluent system using the methods: (a) 0–10 min: 10%→90% CH_3CN, 0.5 mL/min; (b) 0–12 min: 10%→50% CH_3CN & 12–14 min: 50%→90%, 0.5 mL/min; (c) 0–8 min: 10→20% & 8–12 min: 20% iso CH_3CN, 0.5 mL/min; (d) 0–6 min: 10% iso CH_3CN & 6–12 min: 10%→50%, 0.25 mL/min. UV (UltiMate 3000 Photodiode Array Detector) and ESI-MS detections (LCQ DECA XP MAX) were used. Semi-preparative HPLC was performed on Dionex UltiMate 3000 system using a Phenomenex Gemini® C18 TMS end-capped 150 mm×30 mm HPLC column (10 µm particles size) with water (0.05% formic acid): MeOH (0.05% formic acid) eluent system with the same UV-detection. The method utilized for semi-preparative purification was the following: 10% iso MeOH for 30 min, 25 mL/min.

Spectroscopy. All measurements were performed in PIPES-buffered distilled water at pH 6.5 unless indicated otherwise. Complex concentration was nominally 10 µM, however, small quantities of Ln salts may diminish this. Quartz cells with 1 cm optical pathlengths were used for the r.t. measurements. The absorbance spectra were measured by a Varian Cary 100 Bio UV-Visible spectrophotometer. The UV-Vis emission and excitation spectra and antenna fluorescence quantum yields were measured on a Horiba FluoroMax-4P. All emissions were corrected by the wavelength sensitivity (correction function) of the spectrometer. All measurements were performed at r.t. unless stated otherwise.

Antenna fluorescence quantum yields were measured at r.t., using quinine sulfate (QS) in H_2SO_4 0.05 M (Φ_{ref} = 0.59) as reference,[5] in Equation (S1). Quantum yields were calculated according to (S1), with Φ_s the quantum yield of the sample, Φ_{ref} the quantum yield of the reference, I the integrated corrected emission intensity of the sample (s) and of the reference (ref), f_A the absorption factor of the sample (s) and of the reference (ref) at the excitation wavelength and n the refractive indexes of the sample (s) and of the reference (ref). The concentration of the complexes was adjusted to obtain an absorbance around the maxima of the antennae matching that of the QS fluorescence standard (A = 0.10). The excitation wavelength where the absorption factors of the samples and of the reference were

the same was chosen (i.e. where the absorptions are identical). The corrected emission spectra of the sample and reference standard were then measured under the same conditions over the 320–800 nm spectral range as well as blank samples containing only the solvent (PIPES buffered aqueous solutions). The appropriate blanks were subtracted from their respective spectra. The quantum yields were then calculated according to Equation (S1). The given relative error on the quantum yields ($\delta\Phi = \Delta\Phi/\Phi$, where $\Delta\Phi$ is the absolute error) take into account the accuracy of the spectrometer and of the integration procedure [$\delta(I_s/I_{ref}) < 2\%$], an error of 0.59 ±0.01 on the quantum yield of the reference QS [$\delta(\Phi_{ref})$ < 2%], an error on the ratio of the absorption factors [$\delta(f_{Aref}/f_{As}) < 5\%$, relative to the fixed absorption factor of the reference QS] and an error on the ratio of the squared refractive indexes [$\delta(n_s^2/n_{ref}^2) < 1\%$, < 0.25% around 1.333 on each individual refractive index], which sums to a total estimated relative error that should be $\delta\Phi_s < 10\%$. A limit value of 10% is thus chosen.

$$\Phi_S = \frac{I_S}{I_{ref}} \cdot \frac{f_{Aref}}{f_{As}} \cdot \frac{(n_S)^2}{(n_{ref})^2} \cdot \Phi_{ref} \qquad \text{(S1)}$$

Low temperature measurements were done in quartz capillaries (0.2 cm optical pathlength) at 77 K by immersion in a liquid N_2-filled quartz Dewar and with addition of glycerol (1 drop) to the solutions (9 drops) measured at r.t. Glycerol used for low temperature experiments was of 99.9+% purity.

The fluorescence lifetime decays in the nanosecond range were measured on Spectrofluorometer FS5 system from Edinburgh Instruments. The system was equipped with picosecond pulsed light emitting diode EPLED-340 with excitation wavelength at 341.5 nm. The data were acquired in the 50 ns time range with peak preset at 10^4 counts in 1024 channels. The repetition rate of the excitation source was 10 MHz, and the synchronization delay was 80 s. The scatter light profile (prompt signal, black in the decay figures) was recorded for each experiment individually in the same quartz cuvette using diluted Ludox solution in HPLC water at 341.5 nm emission wavelength with similar parameters as were used for the measured sample (red in the decay figures). All measurements were done at r.t. with such concentration of the sample that A = 0.10 at 341.5 nm, and with 10 nm slits width of the emission detector and maximum optical power of the excitation source. All Ln(III) complexes were measured at 377 nm emission wavelength, and the solvent was 10 mM PIPES buffered HPLC water (pH ~ 6.5). The control experiment was performed with Rose Bengal in MeOH at λ_{ex} = 577 nm as a lifetime standard.[4] The obtained data were fitted in the Fluoracle software (green trace in the decay figures) using single exponential reconvolution fit model in Equation (S2), where τ_1 is the sample lifetime, t is time represented in ns, B_1 is the population (100% in all cases) (Table S18–S21 and Figures S101–S125).

$$\boldsymbol{R(t) = B_1 * \exp(-t/\tau_1)} \qquad \text{(S2)}$$

Photostability experiments were performed with **YbL0,3a,c$^{\mathbf{MOM}}$** and **GdL3a$^{\mathbf{MOM}}$** on a Horiba FluoroMax-4P instrument at r.t. at λ_{ex} = 331 nm, A = 0.10 in 10 mM PIPES buffered H_2O or D_2O solutions at pH 6.5 and pD 6.9[6] as well as in unbuffered aqueous or D_2O solutions. Each sample was prepared in a 1 cm screw-capped quartz cuvette (3 mL), and was continuously irradiated for 2 h with emission spectrum recorded every 15 min. The front slit was 3 nm, the exit slit was 2.5 nm. Each sample evolution upon light irradiation was tested via absorbance spectroscopy and LC (with UV-Vis detection) before and after being irradiated. No major differences were noted either in the absorbance spectra or the LC traces for either of the **YbL** samples, for **GdL** the absorption decreased substantially. The signal of the solvent recorded in the same conditions was subtracted from the recorded emission spectra, which were then integrated (346–647 nm). The resulting integrated intensity values were divided by that at t_0 and multiplied by 100%. The photostabilities under Ar or N_2 were recorded with identical samples of complexes purged for 10 min with the corresponding gas.

Near-infrared spectra. UV-Vis measurements were carried out by a Perkin-Elmer lambda 750 and a Perkin Elmer lambda 365 spectrophotometer. Luminescence spectra were recorded on a Horiba Fluorolog FL-3-22 equipped with CW 450W Xenon source. Data were collected by using a water-cooled UV-Vis R2658P PMT from Hamamatsu (range 220-1010 nm), a thermoelectrically-cooled H10330-75 NIR-PMT from Hamamatsu (range 950-1700 nm), and a UV-enhanced photodiode for monitoring the intensity of the excitation light. The signals were corrected for instrument correction factors. The correction factor for the range between 930–950 nm on the NIR detector was calculated by measuring indocyanine green in DMSO ($2.8 \cdot 10^{-5}$ M) with both PMTs, normalizing the background subtracted corrected spectra at 960 nm where both detectors had a known correction function, combining the two spectra, rescaling the stitched spectrum to the initial NIR PMT CPS level and dividing the stitched corrected spectrum by the background subtracted uncorrected signal to obtain a multiplicative correction factors that can be used on background subtracted uncorrected spectra. Values below 930 nm were found to be unusable due to excessive noise generated by the strong correction (i.e. low sensitivity) of the NIR detector in this range. This procedure ensured that the Yb(III) signal could be fully measured with the NIR detector that has a much better sensitivity than the UV-Vis PMT. The Yb(III) signal could not be well-resolved on the UV-Vis PMT. Excitation spectra were calculated by dividing the dark offset subtracted intensities at the chosen emission wavelength as a function of excitation wavelength by the intensities of the excitation light at each excitation wavelength.

The measured excitation and emission spectra were compared by normalizing at the peak intensity of the antenna's lowest energy band and highest energy band respectively, namely 326 nm, 339 nm and 329 nm for the excitation spectra of complexes bearing the Me-, CF_3- and MOM-carbostyril antennae, and respectively 364 nm, 388 nm and 374 nm for the emission spectra. The Yb(III) excitation spectra were recorded at emission wavelength of 997 nm.

Relative quantum yields were determined via the Equation (S3) on the sample solution the absorbances of which were set at $A = 0.10$. The relative quantum yields were calculated by subtracting the average background signal between 1125–1150 nm (S_{bkg}) to the uncorrected raw spectra (S_{Yb}), applying the correction function (CF), dividing the background subtracted corrected emission spectra by the intensity of the excitation light at each scanned wavelengths as recorded by the photodiode detector (R_{Yb}), integrating the spectra between 932–1125 nm (I_{Yb}), dividing the integration value by the integration value of a reference compound ($I_{\mathrm{Yb,ref}}$) and multiplying by an absorption correction factor. The absorption correction factor was calculated by dividing the absorptance ($1-T_{\mathrm{ref}}$) of the reference compound at the excitation wavelength by the absorptance ($1-T$) of the complex at that same wavelength.

$$\varphi_{\mathrm{Yb}} = \frac{\Phi_{\mathrm{Yb}}}{\Phi_{\mathrm{Yb,ref}}} = \frac{I_{\mathrm{Yb}} \cdot (1-T_{\mathrm{ref}})}{I_{\mathrm{Yb,ref}} \cdot (1-T)} = \frac{\int_{\lambda_{min}}^{\lambda_{max}} \frac{[S_{\mathrm{Yb}}(\lambda) - S_{\mathrm{bkg}}(\lambda)] \cdot CF(\lambda)}{R_{\mathrm{Yb}}(\lambda)} \cdot d\lambda}{\int_{\lambda_{min}}^{\lambda_{max}} \frac{[S_{\mathrm{Yb,ref}}(\lambda) - S_{\mathrm{bkg,ref}}(\lambda)] \cdot CF(\lambda)}{R_{\mathrm{Yb,ref}}(\lambda)} \cdot d\lambda} \cdot \frac{(1-T_{\mathrm{ref}})}{(1-T)} \qquad \text{(S3)}$$

The spectra used in this procedure were recorded under identical conditions by exciting at 323 nm with 1 nm increments, an integration time of 1 s and slits open at 14.7 nm and 5 nm to ensure that the collected signal has a decent intensity and therefore that the signal to noise ratio is higher. The precision of the relative quantum yields was estimated by considering the relative errors on the absorbances and the relative error on the integrated spectra. The relative error on the integrated spectra was estimated by calculating the standard deviation of the background signal and multiplying it by the number of wavelengths over which the emission was integrated. The total relative error was then calculated by taking the square root of the sum of the squared relative errors.

Paramagnetic ^{1}H NMR. ^{1}H NMR spectra (400 MHz) of Yb complexes at r.t. were recorded using general parameters: 1 ms relaxation delay and 1024 number of scans. Specific parameters for **YbL**: number of points 65536 or 131072, range −100 to 160 ppm.

Temperature dependent ^{1}H NMR spectra of Yb complexes were recorded at 400 MHz using the following parameters: measurements performed at 10 °C, r.t. (~20 °C), and 40 °C; equilibration time: 15 min; relaxation delay: 1 s; number of scans: 64; range: −150 to 150 ppm.

1D ^{1}H and ^{13}C{^{1}H} NMR experiments and 2D experiments were recorded on a Bruker NEO 600 MHz spectrometer equipped with a 5-mm triple-resonance inverse Z-gradient probe (TBI 1H, 31P, BB). 1D and 2D NMR spectra were collected in pure D_2O.

Variable temperature ^{1}H spectra were recorded over 283−353 K in 10 K increments, for a total of 8 spectra using the following parameters: spectral width, 260 ppm; 30° nutation angle duration, 8.3 µs; recycling delay, 1.1 s (1 s acquisition time and 1 ms relaxation delay). The spectra were 2x zero-filled and subject to an exponential prior for Fourier transformation.

For EXSY experiment, spectra were acquired at 298 K using the pulse sequence noesyph (Bruker library) with a mixing time of 1 ms, 160 averages for each t1 value after 4 dummy scans, a datum set of 8k time domain data point in the t2 dimension with 64 t1 increments, and the States-TPPI method for quadrature detection in the t1 dimension.

COSY spectra were obtained using the non-gradient pulse sequence (Bruker library). The spectra resulted from 8192 (F2) × 256 (F1) data matrix size with 120 scans per t1 increment. A spectral width of 260 ppm was used in both F1 and F2 dimensions.

The 2D ^{1}H-^{13}C HSQC spectra were acquired using the pulse sequence 13C-XL-Alsofast-HMQC.[7] ALSOFAST experiments in this study have been performed with d1=500 ms using forward and reverse INEPT delays of 1.7 ms and 1.3 ms, respectively. 120 scans were collected for each of the 256 t1 values with 8192 complex points in the corresponding FID. Data were transformed with a shifted sine window function along both the F1 and F2 dimensions and with a zero-filling to 512 in F1.

Accumulation lasted ca. 9 h for the ^{13}C{1H} NMR experiments, 3 h for the ^{1}H-^{1}H COSY, and 4.5 h for the ^{1}H-^{13}C HSQC experiments.

Crystallography. Measurements were performed using graphite-monochromatized Mo K_α radiation at 170 K using a Bruker D8 APEX-II equipped with a CCD camera. The structures were solved by direct methods (SHELXS-2014) and refined by full-matrix least-squares techniques against F^2 (SHELXL-2018). The non-hydrogen atoms were refined with anisotropic displacement parameters. The H atoms of the CH_2 / CH groups were refined with common isotropic displacement parameters for the H atoms of the same group and idealized geometry. The H atoms of the methyl groups were refined with common isotropic displacement parameters for the H atoms of the same group and idealized staggered geometry; one methyl group is modelled as a disordered staggered configuration.

Specific for **YbL0^{CF3}**: Lattice H_2O protons were located on the difference map and placed with distance/angle constraints. One H_2O oxygen atom was disorder over two sites with occupancy being refined freely (0.57:0.43).

Electrochemistry. Cyclic voltammograms were measured in a glovebox (Ar) at r.t. (~20 °C) using an AUTOLAB PGSTAT100 potentiostat equipped with a 3 mm glassy carbon electrode, a platinum wire auxiliary electrode, and a silver reference electrode (Ag/$AgNO_3$ 10 mM in MeCN). Measurements

were performed in anhydrous DMF with NBu_4ClO_4 (0.1 M) as the supporting electrolyte. The solvent was degassed prior to bringing it into the glovebox, by doing three freeze-pump-thaw cycles. The electrolyte solution was prepared in the glovebox. The voltammograms were recorded by scanning first toward more negative potential values (reduction).

A solution of NBu_4ClO_4 (0.1 M) in DMF (5 mL) was added to the electrochemical cell. The working electrode was polished with 0.05 µm alumina on a polishing pad, washed with water and ethanol, and dried with air before bringing into the glovebox. This was repeated before each new sample. The three electrodes (GC working electrode, platinum wire auxiliary electrode, and silver reference electrode) were inserted into the cell setup and a background scan was recorded with a scan rate of 100 mV/s, and four sweeps. The ytterbium complex was added in the solution (0.5 mM). Scans were recorded at various scan rates (50 to 1000 mV/s) with four sweeps for each measurement. Ferrocene was used as an internal reference. At the end of the experiment, ferrocene was added to the solution, and cyclic voltammograms were recorded again at various scan rates (50 to 1000 mV/s) with one sweep for each measurement.

Spectroelectrochemistry experiments with **YbL3a^{MOM}** (1 mM) were performed using an AUTOLAB PGSTAT100 potentiostat and an Agilent Spectrophotometer at r.t. (~20 °C). The experiments were performed in a quartz cuvette with 1 mm optical pathlength and 1 mL overall volume of the solution. The cuvette was equipped with a Ag wire as the reference electrode (Ag/AgCl/ sat. KCl_{aq}), a Pt rod as the counter electrode, and a Pt net as the working electrode. An anhydrous DMF solution with 0.1 M NBu_4ClO_4 as the supporting electrolyte was used. The spectroelectrochemistry was studied at an applied potential of –2.15 V vs Fc^+/Fc with 30 min Ar purging before and a positive Ar flow above the solution of **YbL3a^{MOM}** after the start of experiment. The UV-Vis spectra were recorded every 15 s upon applied potential and every 10th spectrum (2.5 min) is reported (Figure S64).

SYNTHESES

Synthetic schemes

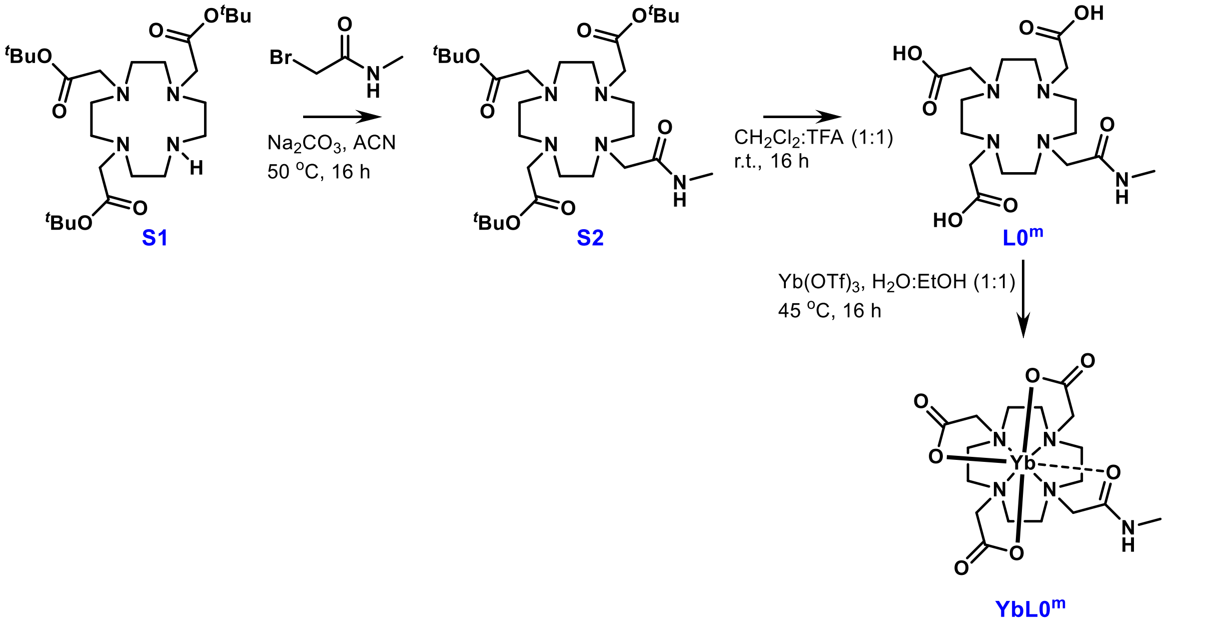

Scheme S1. Synthesis of **YbL0m**.

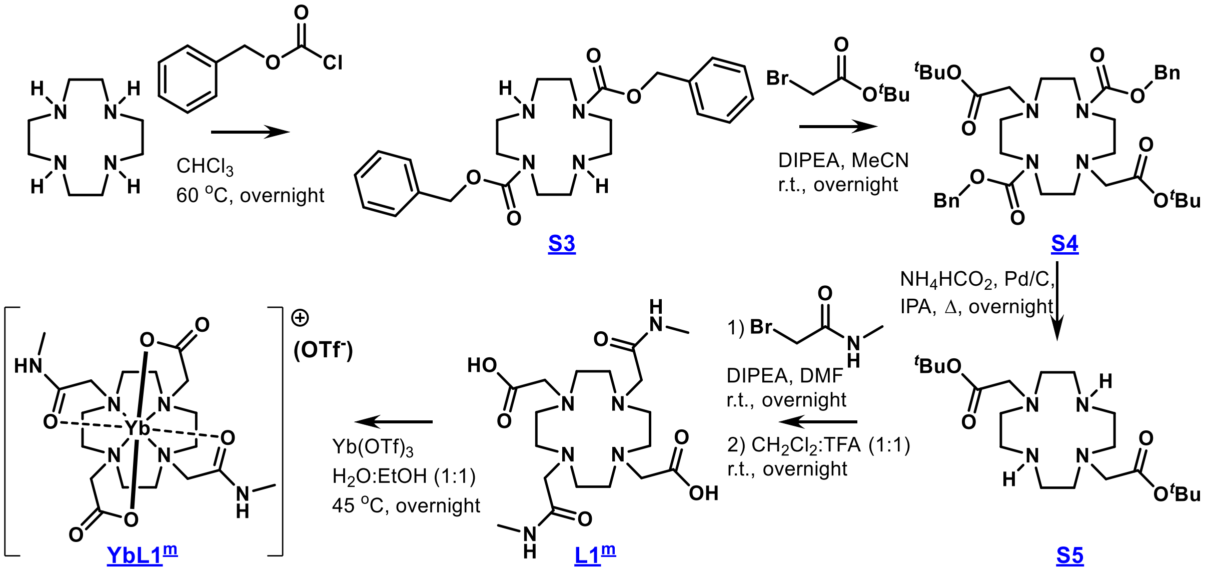

Scheme S2. Synthesis of **YbL1m**.

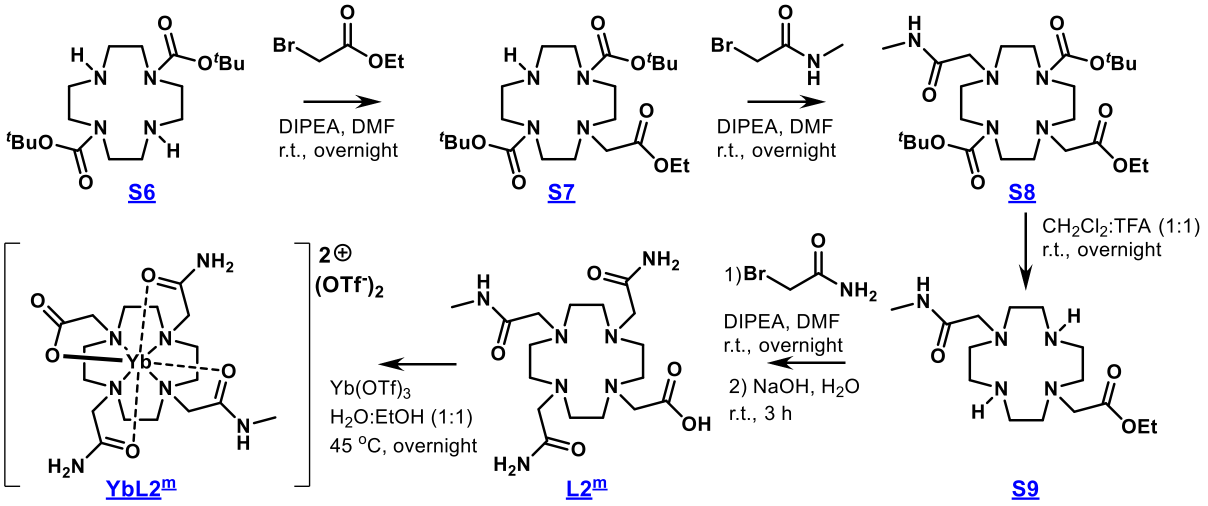

Scheme S3. Synthesis of **YbL2m**.

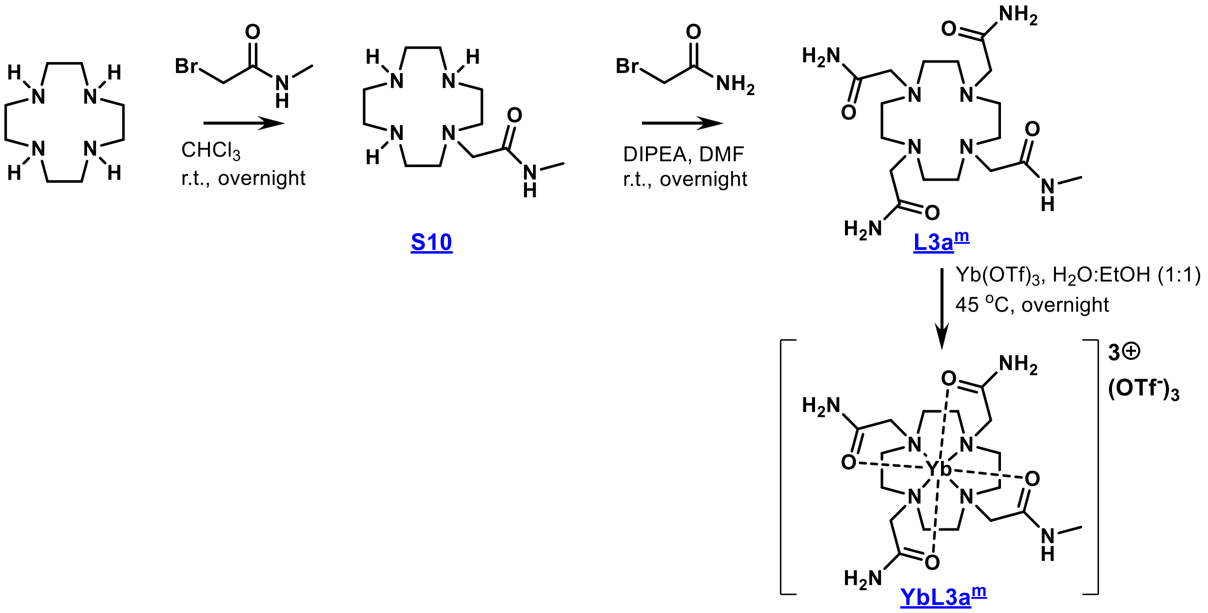

Scheme S4. Synthesis of **YbL3am**.

Synthetic procedures and characterization data

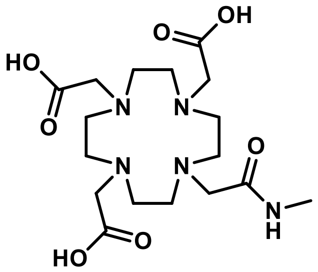

L0^{m}. Adapted procedure.[8] 2-bromo-*N*-methylacetamide (193 mg, 1.27 mmol) was added to a solution of **S1** (500 mg, 0.97 mmol) in MeCN (20 mL), followed by the addition of Na_2CO_3 (168 mg, 1.6 mmol). The reaction mixture was stirred at 50 °C for 16 h. Another equivalent of 2-bromo-*N*-methylacetamide (147 mg, 0.97 mmol) was added. The reaction mixture was filtered, the filtrate was concentrated under reduced pressure, and the crude product was purified by column chromatography [silica, gradient CH_2Cl_2:MeOH (98:2 to 95:5)]. The obtained compound (**S2**, 187 mg, 0.32 mmol) was diluted with CH_2Cl_2 (3.5 mL), and TFA (3.5 mL) was added dropwise to the solution. The reaction mixture was stirred for 16 h at r.t. The mixture was then concentrated under reduced pressure, and the residue was purified by semi-preparative HPLC (see general procedures) yielding an off-white solid (87 mg, 65%). ^{1}H NMR (400 MHz, D_2O) δ ppm 2.67 (s, 3H, CH_3), 2.90–3.18 (m, 8H, CH_2 cyclen), 3.32–3.49 (m, 8H, CH_2 cyclen), 3.49–3.60 (m, 4H, CH_2(CO)), 3.75–3.89 (m, 4H, CH_2(CO)), 8.25 (s, 1H, HCO_2^-); ^{13}C NMR (101 MHz, D_2O) δ ppm 26.1 (CH_3), 48.2 (CH_2 cyclen), 48.6 (CH_2 cyclen), 50.4 (CH_2 cyclen), 51.5 (CH_2 cyclen), 53.3 (CH_2(CO)), 56.1 (CH_2(CO)), 56.5 (CH_2(CO)), 166.7 (HCO_2^-), 170.0 (CO_2H), 172.3 (CO(NHMe)), 174.7 (CO_2H); RP-HPLC t_R = 2.97 min (method(d)); ESI-MS obsd 418.50, calcd 418.23 $(M + H)^+$; HR-ESI-MS obsd 440.2110, calcd 440.2116 $(M + Na)^+$, [M = $C_{17}H_{31}N_5O_7$].

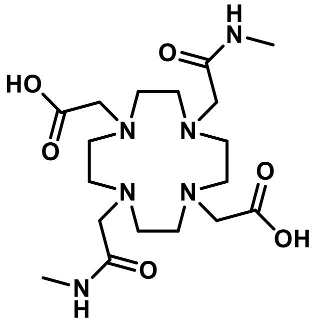

L1^{m}. 2-bromo-*N*-methylacetamide (1.04 g, 6.8 mmol) was added to a solution of **S5**[3] (1.23 g, 3.1 mmol) in DMF (40 mL), followed by DIPEA (2.7 mL, 15.5 mmol). The reaction mixture was stirred at r.t. for 16 h, DMF was co-evaporated with heptane, and the residue was purified by column chromatography [silica, gradient CH_2Cl_2:MeOH (10:0 to 6:4)]. The desired compound was obtained as an oil (3.10 g) that contained traces of DIPEA. Then, 83 mg of this compound (0.16 mmol) was diluted with 1.7 mL of CH_2Cl_2, and TFA (1.7 mL) was added dropwise to the solution. The reaction mixture was stirred at r.t. for 16 h, and was concentrated under reduced pressure. The residue was purified by semi-preparative HPLC (see general procedures) yielding a solid (22 mg, 33%). ^{1}H NMR (400 MHz, DMSO-*d$_6$*) δ ppm 2.68–2.63 (m, 6H, CH_3), 2.66 (s, 8H, CH_2 cyclen), 3.03 (s, 8H, CH_2 cyclen), 3.09 (s, 4H, CH_2(CONHMe)), 3.39 (s, 4H, $CH_2(CO_2H)$), 8.18 (s, 2H, NHMe); ^{13}C NMR (101 MHz, DMSO-*d$_6$*) δ ppm 25.4 (CH_3), 49.3 (CH_2 cyclen), 51.5 (CH_2 cyclen), 54.3 ($CH_2(CO_2H)$), 59.8 (CH_2(CONHMe)), 163.4 (HCO_2^-), 168.7 (CO_2H), 170.4 (CONHMe); RP-HPLC t_R = 2.95 min (method(d)); HR-ESI-MS obsd 429.2457, calcd 429.2467 $(M + H)^-$, [M = $C_{18}H_{32}N_6O_6^{2-}$].

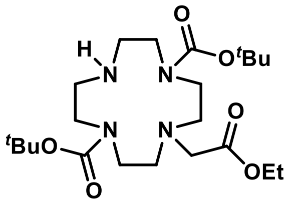

S7. Ethyl bromoacetate (0.15 mL, 1.3 mmol) was added to a solution of **S6**[9] (1.01 g, 2.7 mmol) in DMF (36 mL), followed by the addition of DIPEA (0.94 mL, 5.4 mmol). The reaction mixture was stirred for 16 h at r.t., and the crude product was purified by column chromatography by loading the reaction mixture directly on top of a neutral alumina column packed in acetonitrile [gradient MeCN:MeOH (10:0 to 8:2)]. The desired compound was obtained as an oil (248 mg). NMR analysis showed traces of DIPEA. The product was used without further purification in the next step. ^{1}H NMR (400 MHz, DMSO-*d*$_6$) δ ppm 1.20 (t, *J* = 7.0 Hz, 3H, $CH_2C\underline{H_3}$), 1.24–1.32 (m, 6H, DIPEA, 6H), 1.40 (s, 18H, $C(CH_3)_3$) 1.75 (s, 1H, impurities), 2.73 (s, DMF), 2.89 (s, DMF), 2.94–3.28 (m, 11H, CH_2, DIPEA), 3.33 (s, H_2O), 3.43–4.02 (m, 7H, CH_2, DIPEA), 4.02–4.19 (m, 2H, $C\underline{H_2}CO_2$), 7.95 (s, DMF); ^{13}C NMR (101 MHz, DMSO-*d*$_6$) δ ppm 12.8 ($CH_2\underline{C}H_3$), 14.6, 17.2 (DIPEA), 18.5 (DIPEA), 23.0, 28.4 ($C(\underline{C}H_3)_3$), 31.3 (DMF), 36.3 (DMF), 42.1 (DMF), 45.0 (CH_2), 53.9 (DIPEA), 60.8 ($\underline{C}H_2CH_3$), 80.2 ($CH_2(CO)$), 80.6 ($\underline{C}(CH_3)_3$), 155.5 (CO_2R), 162.8 (DMF); RP-HPLC t_R = 4.70 min (method(d)); ESI-MS obsd 459.53, calcd 459.32 $(M + H)^+$; HR-ESI-MS obsd 459.3182, calcd 459.3177 $(M + H)^+$, [M = $C_{22}H_{42}N_4O_6$].

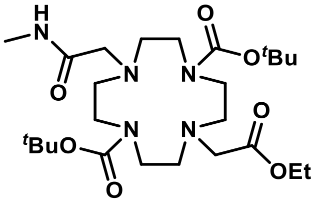

S8. 2-bromo-*N*-methylacetamide (80 mg, 0.52 mmol) was added to a solution of **S7** (180 mg, 0.44 mmol) in DMF (6 mL), followed by the addition of DIPEA (0.15 mL, 0.87 mmol). The reaction mixture was stirred for 16 h at r.t., after which H_2O (100 mL) was added, and extraction was performed with ethyl acetate (3 x 100 mL). The combined organic layers were dried over anhydrous Na_2SO_4 and concentrated under reduced pressure. The crude product was purified by column chromatography [silica, gradient CH_2Cl_2:MeOH (95:5 to 80:20)]. The desired compound was obtained as an oil (160 mg, 77%). ^{1}H NMR (400 MHz, DMSO-*d*$_6$) δ ppm 0.83 (q, *J* = 7.0 Hz, 1H, impurities), 1.19 (t, *J* = 7.0 Hz, 3H, $CH_2C\underline{H_3}$), 1.36 (s, 9H, $C(CH_3)_3$), 1.39 (s, 9H, $C(CH_3)_3$), 2.54–2.71 (m, 8H, CH_2 cyclen), 2.73 (s, DMF), 2.80 (s, 3H, CH_3), 2.89 (s, DMF), 3.04 (s, 1.5H, $\underline{CH_3}OH$), 3.18–3.34 (m, 8H, CH_2 cyclen), 3.38 (s, H_2O), 3.49–3.60 (m, 4H, $CH_2(CO)$), 4.03 (s, 0.5H, $CH_3\underline{OH}$), 4.08 (q, *J* = 7.0 Hz, 2H, $C\underline{H_2}CH_3$), 4.13 (m, 1H, impurities), 7.95 (s, DMF); ^{13}C NMR (101 MHz, DMSO-*d*$_6$) δ ppm 14.2 ($CH_2\underline{C}H_3$), 25.4, 28.1 ($C(\underline{C}H_3)_3$), 30.9 (DMF), 35.9 (DMF), 46.5 (CH_2), 53.7 (CH_2), 59.8 ($\underline{C}H_2CH_3$), 78.8 ($\underline{C}(CH_3)_3$), 162.4 (DMF), 170.6 (CO); RP-HPLC t_R = 9.30 min (method(b)); ESI-MS obsd 530.77, calcd 530.35 $(M + H)^+$; HR-ESI-MS obsd 530.3569, 552.3391, calcd 530.3548 $(M + H)^+$, 552.3368 $(M + Na)^+$, [M = $C_{25}H_{47}N_5O_7$].

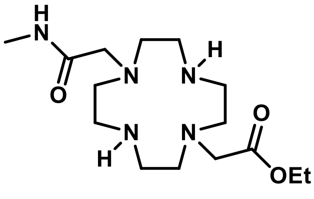

S9. **S8** (117 mg, 0.22 mmol) was dissolved in 2.5 mL of CH_2Cl_2, and trifluoroacetic acid (2.5 mL) was added dropwise to the solution. The reaction mixture was stirred for 16 h at r.t., concentrated under

reduced pressure, and purified by semi-preparative HPLC (see general procedures). The desired compound was obtained as an oil (34 mg, 47%). ^{1}H NMR (400 MHz, DMSO-*d*$_6$) δ ppm 1.20 (t, *J* = 7.0 Hz, 3H, $CH_2C\underline{H_3}$), 2.61 (s, 3H, $NHC\underline{H_3}$), 2.72–2.84 (m, 15H, CH_2 cyclen), 2.88–2.94 (m, 1H, impurities), 2.97–3.07 (m, 2H, NH), 3.23 (s, 2H, CH_2(CONHMe)), 3.52 (s, 2H, $CH_2(CO_2Et)$), 4.10 (q, *J* = 7.0 Hz, 3H, $C\underline{H_2}CH_3$), 7.87 (m, 1H, N$\underline{H}$Me), 8.27 (s, 2H, HCO_2^-); ^{13}C NMR (101 MHz, DMSO-*d*$_6$) δ ppm 14.2 ($CH_2\underline{C}H_3$), 25.4 ($NHC\underline{H_3}$), 42.7 (impurities), 43.7 (impurities), 45.1 (CH_2 cyclen), 45.2 (CH_2 cyclen), 50.2 (CH_2 cyclen), 50.9 (CH_2 cyclen), 55.1 ($CH_2(CO_2Et)$), 57.5 (CH_2(CONHMe)), 60.1 ($C\underline{H_2}CH_3$), 164.5 (HCO_2^-), 171.3 (CO), 171.4 (CO); RP-HPLC t_R = 2.32 min (method(d)); ESI-MS obsd 331.06, calcd 330.25 $(M + H)^+$; HR-ESI-MS obsd 165.6292, 330.2506, calcd 165.6286 $(M + 2H)^{2+}$, 330.2500 $(M + H)^+$, [M = $C_{15}H_{31}N_5O_3$].

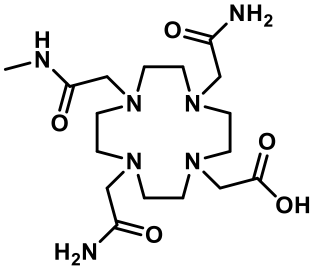

L2^m. 2-bromoacetamide (32.7 mg, 0.24 mmol) was added to a solution of **S9** (39 mg, 0.12 mmol) in DMF (1.7 mL), followed by the addition of DIPEA (0.62 mL, 0.36 mmol). The reaction mixture was stirred for 16 h at r.t., at which point H_2O (2 mL) was added. The crude product was purified by semi-preparative HPLC (see general procedures). The isolated product (22 mg, 0.05 mmol) was dissolved in water (600 µL) and NaOH (1 M, 124 µL) was added to the solution. The reaction mixture was sonicated for 3 h, and the desired compound was obtained after purification by semi-preparative HPLC (see general procedures) as a solid (35 mg, quantitative). ^{1}H NMR (400 MHz, DMSO-*d*$_6$) δ ppm 1.75 (s, 1H, impurities), 2.15–2.34 (m, 8H, CH_2), 2.55–2.67 (m, 8H, CH_2), 2.69–3.13 (m, 11H, CH_2CO, CH_3) 7.04 (s, 2H, NH_2), 7.58 (s, 2H, NH_2), 8.03 (m, 1H, NHMe), 8.44 (s, 5H, HCO_2^-); ^{13}C NMR (101 MHz, DMSO-*d*$_6$) δ ppm 22.6 (impurities), 25.5 (CH_3), 50.4 (br, CH_2), 56.6 (CH_2), 57.0 (CH_2), 58.9 (CH_2), 166.7 (HCO_2^-), 171.4 (CO(NHMe)), 173.1 (CO), 174.2 (CO); RP-HPLC t_R = 2.42 min (method(d)); ESI-MS obsd 416.76, 438.73, calcd 416.26 $(M + H)^+$, 438.24 $(M + Na)^+$; HR-ESI-MS obsd 416.2607, 438.2410, 454.2076, calcd 416.2616 $(M + H)^+$, 438.2435 $(M + Na)^+$, 454.2175 $(M + K)^+$, [M = $C_{17}H_{33}N_7O_5$].

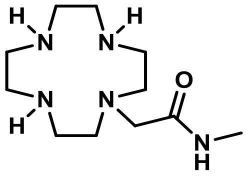

S10. Known compound, new procedure.[10] 2-bromo-*N*-methylacetamide (500 mg, 3.29 mmol) was added to a solution of cyclen (1.70 g, 9.87 mmol) in $CHCl_3$ (9 mL), and stirred at r.t. for 16 h. The reaction mixture was concentrated under reduced pressure, and the crude product was purified by column chromatography [silica, isocratic gradient CH_2Cl_2:MeOH:NH_3 (aq) (50:45:5)]. The desired compound was obtained as an oil (223 mg, 28%). ^{1}H NMR (400 MHz, DMSO-*d*$_6$) δ ppm 2.45–2.48 (m, 2H, CH_2 cyclen), 2.52–2.57 (m, 8H, CH_2 cyclen), 2.57–2.64 (m, 9H, CH_2 cyclen, and CH_3), 3.04 (s, 2H, CH_2(CO)), 3.40 (s, H_2O), 8.10 (s, 1H, NHMe); ^{13}C NMR (101 MHz, DMSO-*d*$_6$) δ ppm 25.3 (CH_3), 45.1 (CH_2 cyclen), 46.1 (CH_2 cyclen), 46.8 (CH_2 cyclen), 52.1 (CH_2 cyclen), 58.0 (CH_2(CO)), 171.1 (C=O); RP-HPLC t_R = 1.13 min (method(c)); ESI-MS obsd 244.64, calcd 244.21 $(M + H)^+$; HR-ESI-MS obsd 244.2149, calcd 244.2132 $(M + H)^+$, [M = $C_{11}H_{25}N_5O$].

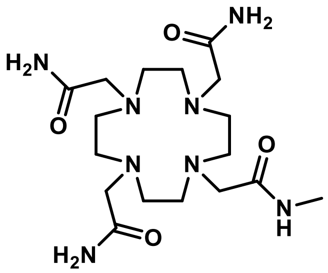

L3a^{m}. 2-bromoacetamide (177 mg, 1.28 mmol) was added to a solution of **S10** (104 mg, 0.43 mmol) in DMF (6 mL), followed by the addition of DIPEA (350 µL, 2.01 mmol). The reaction mixture was stirred at r.t. for 16 h. Purification was performed by semi-preparative HPLC (see general procedures). The desired compound was obtained as a solid (44 mg, 25%). ^{1}H NMR (400 MHz, DMSO-d_6) δ ppm 1.05 (t, J = 7.0 Hz, 6H, EtOH), 2.57–2.76 (m, 20H, CH_2 cyclen, and CH_3), 2.97–3.13 (m, 8H, CH_2(CO)), 3.44 (q, J = 7.0 Hz, 4H, EtOH), 6.96–7.11 (m, 3H, NH_2), 7.48–7.61 (m, 3H, NH_2), 7.88 (q, J = 4.5 Hz, 1H, NHMe), 8.28 (s, 1H, HCO_2^-); ^{13}C NMR (101 MHz, DMSO-d_6) δ ppm 18.6 (EtOH), 25.5 (CH_3), 52.3 (CH_2 cyclen), 52.4 (CH_2 cyclen), 56.1 (EtOH), 57.0 (CH_2(CO)), 57.2 (CH_2(CO)), 57.8 (CH_2(CO)), 164.4 (HCO_2^-), 170.5 (CO(NHMe)), 172.0 ($CONH_2$), 172.2 ($CONH_2$); RP-HPLC t_R = 2.25 min (method(d)); ESI-MS obsd 208.56, 416.14, calcd 208.26 $(M + 2H)^{2+}$, 415.51 $(M + H)^+$; HR-ESI-MS obsd 208.14239, 415.2771 calcd 208.14239 $(M + 2H)^{2+}$, 415.2776 $(M + H)^+$, [M = $C_{17}H_{34}N_8O_4$].

General procedure for the synthesis of YbL^{MOM} and YbL0^{CF3} complexes. Samples of the appropriate ligands (**L^{MOM}** or **L0^{CF3}**, 1 equiv.) and anhydrous $YbCl_3$ (2.4 equiv.) were dissolved in a H_2O:EtOH mixture (1:1, 0.05 M). The reaction mixtures were stirred at 45 °C for 16 h. The following day the completion of the complexation was confirmed via LC-MS and TLC analysis. The excess lanthanide(III) salt was removed via column chromatography on neutral alumina (Isopropanol:H_2O, 9:1→6:4; Isopropanol:EtOH:H_2O, 4:2:4→2:4:4) yielding the colorless complexes. The isolated compounds contained traces of $YbCl_3$. For CV measurements $Yb(OTf)_3$ was used instead of the chloride for the synthesis of the analogous complexes with different counterions.

General procedure for the synthesis of LuL^{MOM} and YbL^{m} complexes. Samples of **L^{MOM}** (1 equiv.) or **L^{m}** (1 equiv.) and $LuCl_3$ (1.05 eq.) or $Yb(OTf)_3$ (0.95 equiv.) were placed into a 1 mL-vial. The solids were dissolved in a mixture of H_2O and EtOH (1:1, 0.05 M). The reaction mixtures were heated at 45 °C for 16 h. When needed, the pH of the solutions was adjusted to pH 4–5 by the addition of NaOH (1 M). The completion of the complexation was confirmed by LC-MS. Then the solvent was removed by evaporation, and the products were used without further purification.

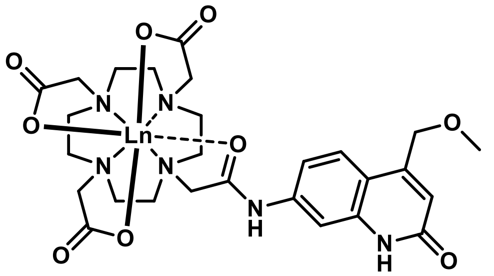

YbL0^{MOM}. 35 mg (88%). ^{1}H NMR (600 MHz, D_2O) δ ppm 132.02, 120.52, 114.27, 113.62, 86.56, 57.30, 42.82, 36.39, 35.12, 29.55, 27.80, 25.40, 24.18, 23.67, 19.65, 17.59, 17.24, 15.36, 14.07, 11.78, 11.46, 10.58, 10.12, 7.60, 7.30, 4.97, 1.93, −0.67, −3.98, −10.27, −15.68, −19.62, −22.34, −23.85, −24.75, −32.09, −38.24, −42.21, −44.20, −46.06, −47.30, −47.78, −48.88, −55.46, −61.11, −62.47, −68.53, −72.58, −73.12, −74.23; ^{13}C NMR (151 MHz, D_2O) δ ppm 171.88, 152.75, 145.19, 135.81,

128.62, 121.63, 120.51, 117.47, 73.30, 60.08, 15.74, 7.81; RP-HPLC t_R = 9.08 min (method(d)); ESI-MS obsd 762.18, calcd 762.20 $(M + H)^+$; HR-ESI-MS obsd 784.17559, calcd 784.17508 $[(M + Na)^+$, M = $C_{27}H_{35}N_6O_9Yb]$; λ_{em} = 376 nm (λ_{ex} = 329 nm), 981, 996, 1028 nm (λ_{ex} = 323 nm).

LuL0^{MOM}. Known compound, new procedure.[11] 12 mg (93% of expected mass). ^1H NMR (400 MHz, D_2O) δ ppm 2.44–4.09 (m, 27H + 3H from MeOH), 3.49 (s, 3H), 4.75 (s, 2H), 6.62 (s, 1H), 7.31 (d, *J* = 9.0 Hz, 1H), 7.74 (d, *J* = 9.0 Hz, 1H), 7.90 (s, 1H); RP-HPLC t_R = 3.10 min (method(d)); ESI-MS obsd 383.30, 763.65, calcd 382.10 $(M + 2H)^{2+}$, 763.19 $(M + H)^+$; HR-ESI-MS obsd 401.0737, 763.1941, calcd 401.0789 $(M + H + K)^{2+}$, 763.1946 $(M + H)^+$, [M = $C_{27}H_{35}N_6O_9Lu$]; λ_{em} = 375 nm (λ_{ex} = 308 nm).

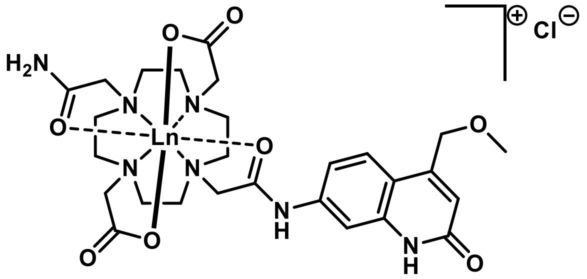

YbL1^{MOM}. 39 mg (94%). ^1H NMR (400 MHz, D_2O) δ ppm –73.11, –71.58, –65.00, –62.31, –59.75, –48.69, –47.79, –8.90, –0.41, 2.44, 2.66, 6.52, 6.70, 6.82, 6.82, 6.94, 9.08, 9.54, 10.71, 12.23, 22.41, 26.41, 28.06, 30.16, 35.38, 100.86, 101.41, 107.64, 114.47; RP-HPLC t_R = 1.40 min (method(c)); ESI-MS obsd 761.59, calcd 761.21 $(M)^+$; HR-ESI-MS obsd 761.2089, calcd 761.2091 $[(M)^+$, M = $C_{27}H_{37}N_7O_8Yb]$; λ_{em} = 375 nm (λ_{ex} = 329 nm), 981, 997, 1027 nm (λ_{ex} = 323 nm).

LuL1^{MOM}. 13 mg (90% of expected mass). ^1H NMR (400 MHz, D_2O) δ ppm 2.49–2.99 (m, 13H), 3.25–4.14 (m, 14H + 6H from 2 MeOH), 3.50 (s, 3H), 4.83 (s, 2H), 6.65 (s, 1H), 7.32 (d, *J* = 8.5 Hz, 1H), 7.77 (d, *J* = 8.5 Hz, 1H), 7.89 (s, 1H); RP-HPLC t_R = 2.57 min (method(d)); ESI-MS obsd 381.60, 762.21, calcd 381.61 $(M)^{2+}$, 762.21 $(M)^+$; HR-ESI-MS obsd 381.6085, 762.2096, calcd 381.6089 $(M)^{2+}$, 762.2106 $[(M)^+$, M = $C_{27}H_{37}N_7O_8Lu]$; λ_{em} = 376 nm (λ_{ex} = 308 nm).

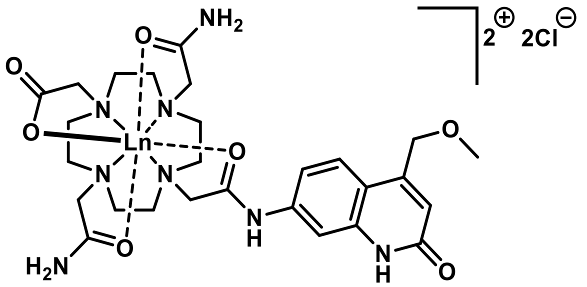

YbL2^{MOM}. 55 mg (95%). ^1H NMR (400 MHz, D_2O) δ ppm –63.37, –62.40, –61.09, –50.32, –35.33, –29.85, –26.17, –25.93, –23.43, –21.64, 2.94, 3.89, 5.13, 7.43, 7.74, 7.90, 9.50, 12.43, 15.33, 19.77, 20.92, 21.15, 23.81, 25.15, 94.71, 102.65, 108.85, 113.90; RP-HPLC t_R = 1.22 min (method(c)); ESI-MS obsd 380.53, calcd 380.62 $(M)^{2+}$; HR-ESI-MS obsd 380.6160, calcd 380.6162 $[(M)^{2+}$, M = $C_{27}H_{39}N_8O_7Yb]$; λ_{em} = 375 nm (λ_{ex} = 329 nm), 982, 994, 1024 nm (λ_{ex} = 323 nm).

LuL2^{MOM}. 14 mg (99% of expected mass). ^1H NMR (400 MHz, D_2O) δ ppm 2.52–3.05 (m, 13H), 3.30–4.08 (m, 14H + 3H from MeOH), 3.51 (s, 3H), 4.81 (s, 2H), 6.71 (s, 1H), 7.39 (d, *J* = 8.5 Hz, 1H), 7.84 (d, *J* = 8.5 Hz, 1H), 7.91 (s, 1H); RP-HPLC t_R = 2.25 min (method(d)); ESI-MS obsd 381.79, calcd

381.12 $(M)^{2+}$; HR-ESI-MS obsd 381.1166, calcd 381.1169 $[(M)^{2+}$, M = $C_{27}H_{39}N_8O_7Lu]$; λ_{em} = 375 nm (λ_{ex} = 308 nm).

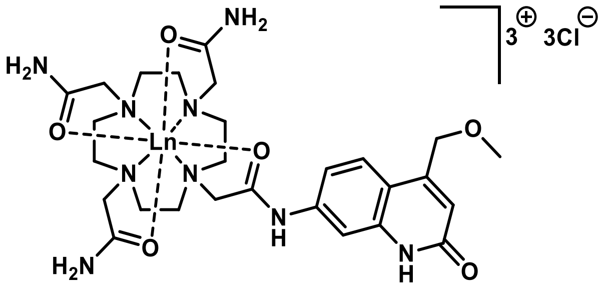

YbL3a[MOM]. 45 mg (70%). ^{1}H NMR (400 MHz, D_2O) δ ppm –57.24, –55.88, –55.36, –40.32, –36.24, –29.85, –29.50, –25.29, –22.55, –21.76, –20.07, 3.92, 5.31, 6.28, 7.75, 8.21, 11.42, 14.42, 15.24, 15.46, 15.68, 17.85, 18.33, 23.33, 94.66, 96.01, 101.34; ^{13}C NMR (125 MHz, D_2O) δ ppm 60.34, 73.64, 118.02, 120.92, 121.99, 129.08, 136.42, 145.61, 153.15, 172.38; RP-HPLC t_R = 1.12 min (method(c)); ESI-MS obsd 254.08, calcd 253.75 $(M)^{3+}$; HR-ESI-MS obsd 253.7517, calcd 253.7519 $[(M)^{3+}$, M = $C_{27}H_{41}N_9O_6Yb^{3+}]$; λ_{em} = 376 nm (λ_{ex} = 329 nm), 993, 1022 nm (λ_{ex} = 323 nm).

LuL3a[MOM]. 14 mg (95% of expected mass). ^{1}H NMR (400 MHz, D_2O) δ ppm 2.62–3.00 (m, 13H), 3.30–4.08 (m, 14H + 4.5H from 1.5 MeOH), 3.51 (s, 3H), 4.82 (s, 2H), 6.72 (s, 1H), 7.40 (dd, J_1 = 9.0 Hz, J_2 = 2.0 Hz 1H), 7.86 (d, J = 9.0 Hz, 1H), 7.92 (d, J = 2.0 Hz, 1H); RP-HPLC t_R = 2.22 min (method(d); ESI-MS obsd 380.73, calcd 380.62 $(M – H)^{2+}$; HR-ESI-MS obsd 380.6249, calcd 380.6249 $[(M – H)^{2+}$, M = $C_{27}H_{41}N_9O_6Lu]$; λ_{em} = 376 nm (λ_{ex} = 308 nm).

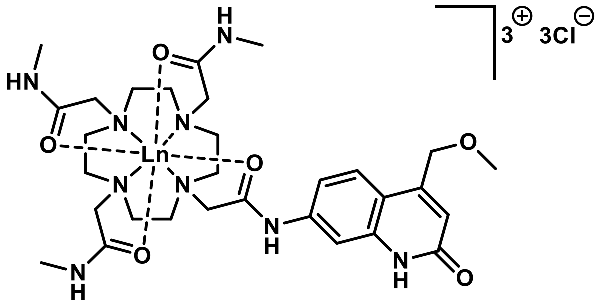

YbL3b[MOM]. 22 mg (69%). ^{1}H NMR (400 MHz, D_2O) δ ppm –56.66, –55.54, –42.42, –37.28, –30.18, –29.58, –24.06, –18.16, –16.81, –9.64, –8.28, –3.40, 5.55, 7.74, 8.09, 8.97, 10.14, 14.11, 14.32, 14.53, 15.66, 17.48, 19.87, 25.46, 92.07, 93.39, 102.11; RP-HPLC t_R = 2.22 min (method(d)); ESI-MS obsd 267.67, calcd 267.77 $(M)^{3+}$; HR-ESI-MS obsd 267.7674, calcd 267.7675 $[(M)^{3+}$, M = $C_{30}H_{47}N_9O_6Yb]$; λ_{em} = 376 nm (λ_{ex} = 329 nm), 993, 1021 nm (λ_{ex} = 323 nm).

LuL3b[MOM]. 14 mg (95% of expected mass). ^{1}H NMR (400 MHz, D_2O) δ ppm 2.59–2.98 (m, 23H), 2.89 (s, 9H) 3.28–4.23 (m, 13H + 5H from 1.67 MeOH), 3.51 (s, 3H), 4.83 (s, 2H), 6.73 (s, 1H), 7.49 (dd, J_1 = 9.0 Hz, J_2 = 2.0 Hz 1H), 7.78 (d, J = 2.0 Hz, 1H), 7.87 (d, J = 9.0 Hz, 1H); RP–HPLC t_R = 2.25 min (method(d)); ESI-MS obsd 268.19, calcd 268.10 $(M)^{3+}$; HR-ESI-MS obsd 268.1015, 401.6484 calcd 268.1014 $(M)^{3+}$, 401.6484 $(M – H)^{2+}$ $[M = C_{30}H_{47}N_9O_6Lu]$; λ_{em} = 376 nm (λ_{ex} = 308 nm).

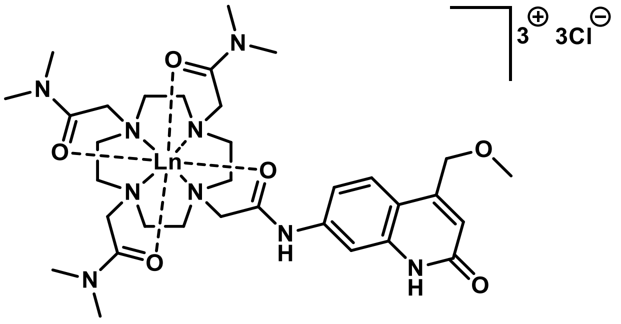

YbL3c^{MOM}. 33 mg (quant.). ^{1}H NMR (400 MHz, D_2O) δ ppm –63.92, –59.48, –58.34, –48.36, –47.90, –36.73, –35.04, –26.01, –21.86, –21.02, –17.37, –15.19, –14.86, –8.33, –7.66, –6.50, –6.37, –6.22, 2.04, 3.50, 6.61, 7.07, 7.85, 12.46, 13.70, 14.29, 16.63, 17.87, 20.38, 25.07, 106.04, 108.41, 110.14, 112.94; RP-HPLC t_R = 2.27 min (method(d)); ESI-MS obsd 422.86, calcd 422.68 $(M)^{2+}$; HR-ESI-MS obsd 281.7831, calcd 281.7832 [$(M)^{3+}$, M = $C_{33}H_{53}N_9O_6Yb^{3+}$]; λ_{em} = 376 nm (λ_{ex} = 329 nm), 981, 996, 1025 nm (λ_{ex} = 323 nm).

LuL3c^{MOM}. The crude complex was purified via a similar procedure as Yb(III) complexes. 8.5 mg (quant.). ^{1}H NMR (400 MHz, D_2O) δ ppm 2.60–3.20 (m, 30H) 3.43–3.67 (m, 8H), 3.54 (s, 3H), 3.82–4.19 (m, 7H), 4.87 (s, 2H), 6.76 (s, 1H), 7.55 (dd, J_1 = 8.9 Hz, J_2 = 2.1 Hz, 1H), 7.75 (s, 1H), 7.90 (d, J = 8.9 Hz, 1H); RP-HPLC t_R = 2.27 min (method(d)); ESI-MS obsd 422.86, calcd 422.67 $(M)^{2+}$; HR-ESI-MS obsd 282.1180, 422.6731 calcd 282.1170 $(M)^{3+}$, 422.6719 $(M)^{2+}$, [M = $C_{33}H_{53}N_9O_6Yb$]; λ_{em} = 376 nm (λ_{ex} = 308 nm).

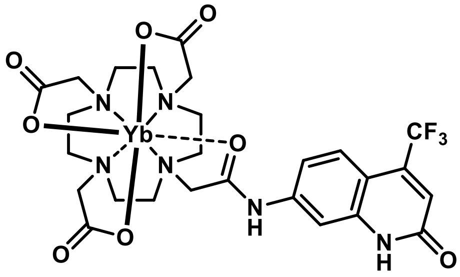

YbL0^{CF3}. 13 mg (99%). ^{1}H NMR (400 MHz, D_2O) δ ppm –73.21, –72.04, –71.60, –68.09, –62.33, –60.75, –46.09, –41.75, –24.57, –23.45, –18.85, –9.05, 2.90, 3.19, 3.45, 3.78, 4.09, 8.96, 9.53, 9.88, 11.10, 11.33, 15.06, 16.50, 17.38, 23.16, 27.61, 29.63, 36.41, 54.38, 82.64, 111.63, 112.37, 118.87, 130.55; RP-HPLC t_R = 8.63 min (method(c)); ESI-MS obsd 786.01, calcd 786.16 $(M + H)^+$; HR-ESI-MS obsd 808.1373, calcd 808.1362 [$(M + Na)^+$, M = $C_{26}H_{30}N_6O_8F_3Yb$]; λ_{em} = 391 nm (λ_{ex} = 329 nm), 981, 997, 1027 nm (λ_{ex} = 323 nm).

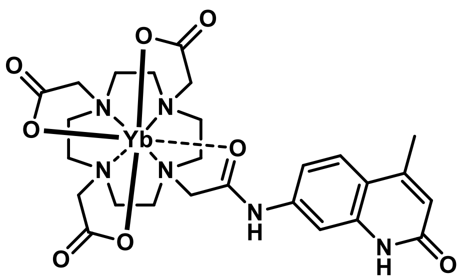

YbL0^{Me}. Known compound, the report of ^{1}H NMR data.[11] 3.9 mg (83%). ^{1}H NMR (400 MHz, D_2O) δ ppm –73.67, –72.65, –72.31, –68.33, –61.53, –59.87, –45.45, –41.60, –24.96, –23.92, –19.91, –11.15,

1.98, 2.11, 2.90, 3.18, 3.31, 3.35, 3.45, 3.63, 3.68, 3.78, 3.79, 3.85, 4.09, 4.32, 4.65, 7.84, 9.26, 9.80, 10.88, 11.86, 15.22, 17.12, 17.68, 23.49, 26.16, 28.97, 35.61, 57.18, 84.90, 112.82, 113.59, 119.59, 130.49. RP-HPLC t_R = 1.99 min (method(c)); ESI-MS obsd 731.96, calcd 731.62 $(M + H)^+$; HR-ESI-MS obsd 754.1647, calcd 754.1645 $[(M + Na)^+$, M = $C_{26}H_{33}N_6O_8Yb]$; λ_{em} = 366 nm (λ_{ex} = 329 nm), 980, 996, 1027 nm (λ_{ex} = 323 nm).

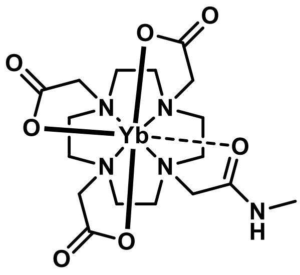

YbL0ᵐ. 18 mg (100% of expected mass). ^{1}H NMR (400 MHz, D_2O) δ ppm −82.57, −78.30, −78.15, −76.49, −75.34, −55.74, −50.33, −45.20, −42.21, −38.82, −37.51, −32.97, −28.89, −28.72, −27.23, −12.05, −2.82, 1.23, 2.68, 2.74, 3.13, 3.40, 3.54, 3.66, 3.82, 4.19, 9.76, 15.89, 16.71, 17.54, 18.54, 20.16, 22.27, 24.59, 24.84, 28.80, 69.45, 71.16, 79.48, 81.59, 121.16, 122.26, 127.52, 128.91, 133.79; RP-HPLC 3.15 min (method(d)); ESI-MS obsd 589.67, 1177.17, calcd 589.14 $(M + H)^+$, 1177.28 $(2M + H^+)^+$; HR-ESI-MS obsd 611.1276, calcd 611.1276 $(M + Na)^+$, [M = $C_{17}H_{28}N_5O_7Yb$].

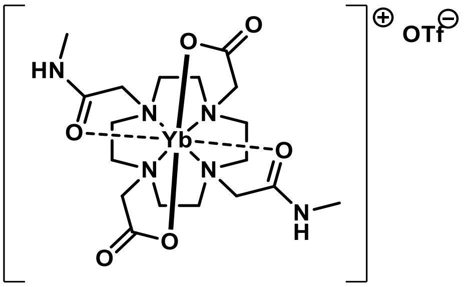

YbL1ᵐ. 15 mg (67% of expected mass). ^{1}H NMR (400 MHz, D_2O) δ ppm −72.74, −68.15, −62.41, −44.30, −19.16, −13.17, −7.77, −2.79, −0.21, 1.30, 1.32, 1.86, 8.43, 11.11, 12.52, 13.26, 22.97, 29.52, 64.76, 112.82, 116.55; RP-HPLC 2.40 min (method(d)); ESI-MS obsd 602.86, calcd 602.18 $(M)^+$; HR-ESI-MS obsd 602.1767, calcd 602.1769 $(M)^+$, [M = $C_{18}H_{32}N_6O_6Yb$].

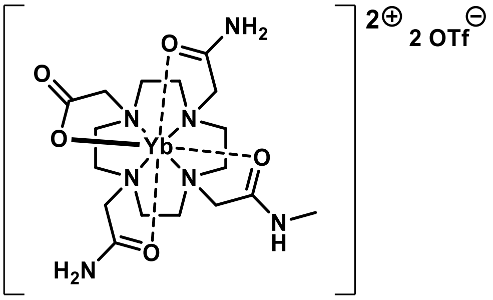

YbL2ᵐ. 10 mg (71% of expected mass). ^{1}H NMR (400 MHz, D_2O) δ ppm −66.08, −64.84, −64.01, −63.73, −57.09, −48.01, −38.12, −37.22, −22.49, −18.57, −18.16, −16.14, −10.92, 1.28, 1.98, 2.75, 3.36, 3.68, 6.91, 8.51, 10.36, 17.04, 17.22, 20.34, 20.76, 23.13, 29.19, 100.04, 106.59, 109.64, 113.63; RP-HPLC 2.32 min (method(d)); ESI-MS obsd 294.57, calcd 294.09 $(M)^{2+}$; HR-ESI-MS obsd 294.0922, 737.1376, calcd 294.0923 $(M)^{2+}$, 737.1369 $(M + CF_3SO_3^-)^+$, [M = $C_{17}H_{32}N_7O_5Yb$].

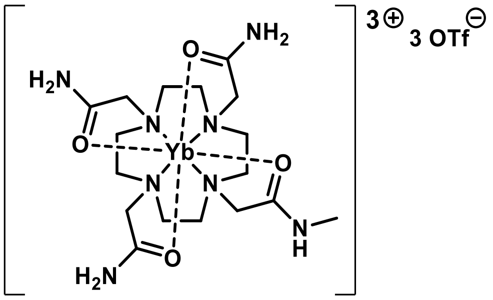

YbL3^m^. 24 mg (92% of expected mass). ^{1}H NMR (400 MHz, D_2O) δ ppm −60.15, −59.72, −59.43, −35.72, −33.50, −30.23, −29.75, −28.13, −27.22, −26.03, −25.47, −12.14, −6.82, 1.16, 2.74, 2.94, 3.14, 3.26, 3.59, 3.65, 4.11, 4.16, 8.07, 14.13, 15.77, 16.01, 16.66, 16.85, 18.75, 19.21, 19.91, 98.65, 100.83, 101.03, 101.70; RP-HPLC 2.27 min (method(d)); ESI-MS obsd 294.50, calcd 294.09 $(M)^{2+}$; HR-ESI-MS obsd 293.6001, calcd 293.6002 $(M)^{2+}$, [M = $C_{17}H_{33}N_8O_4Yb$].

^{1}H NMR SPECTRA OF Lu(III) AND Yb(III) COMPLEXES

^{1}H NMR spectra of Lu(III) complexes

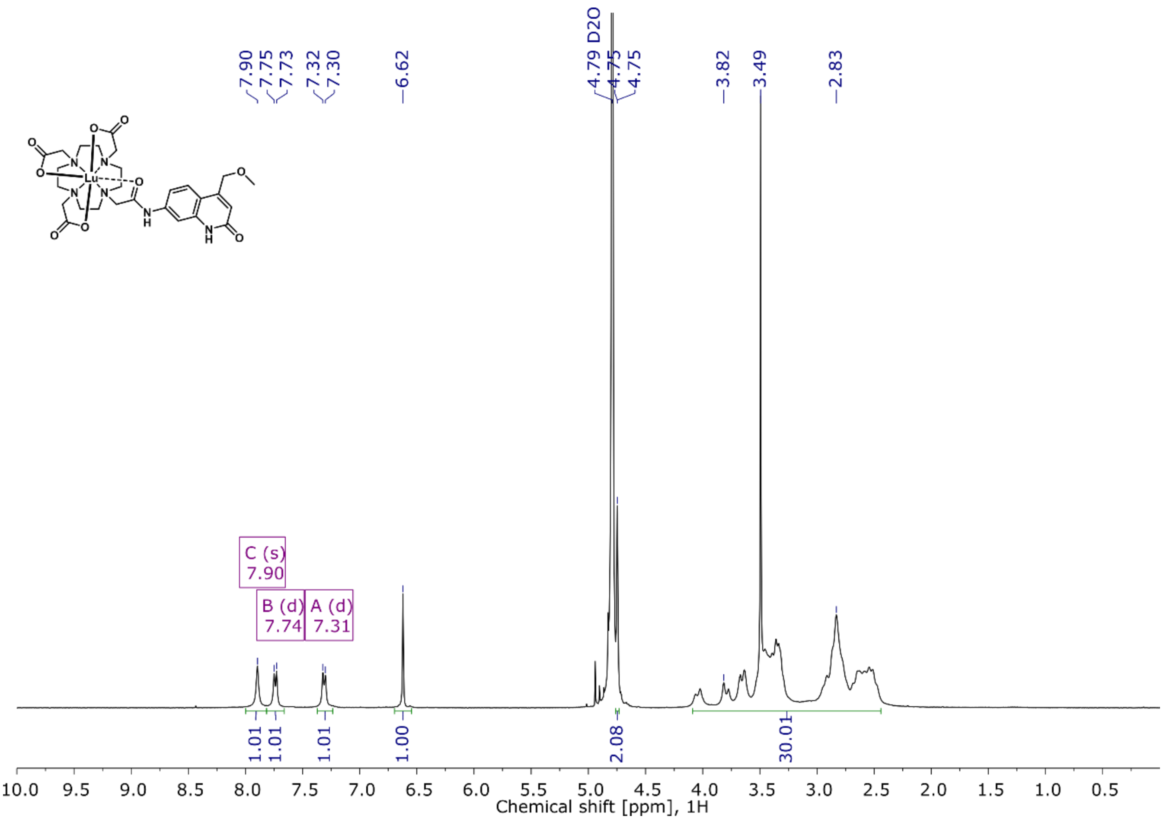

Figure S1. ^{1}H NMR spectrum (400 MHz) of **LuL0^{MOM}** measured in D_2O at 298 K.

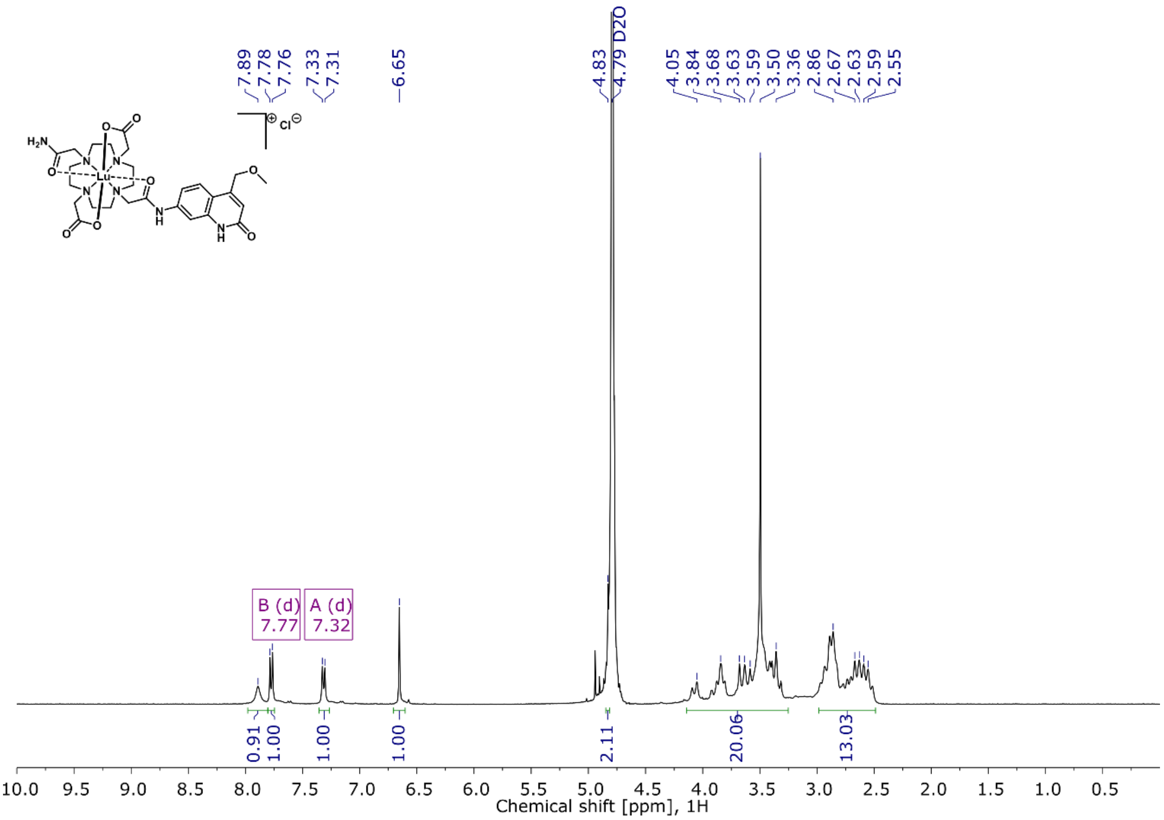

Figure S2. ^{1}H NMR spectrum (400 MHz) of **LuL1^{MOM}** measured in D_2O at 298 K.

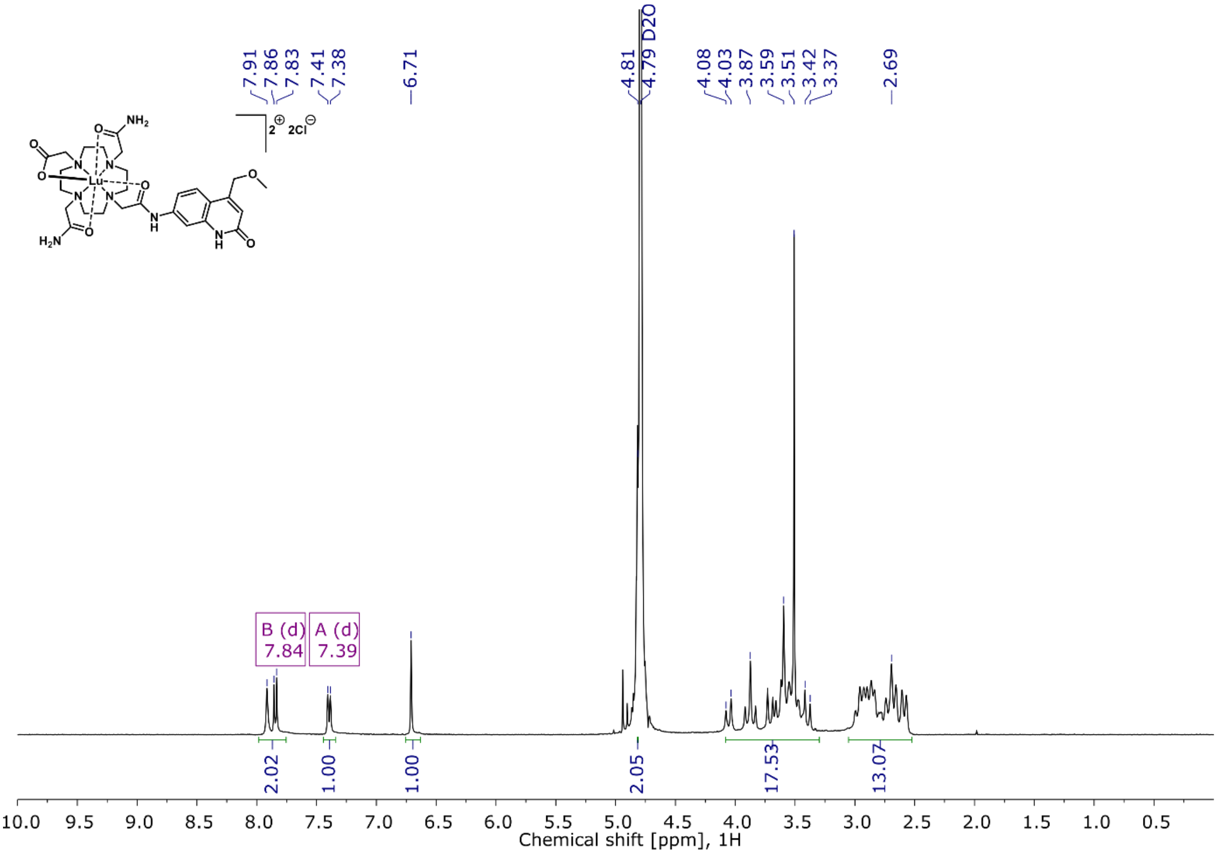

Figure S3. ^{1}H NMR spectrum (400 MHz) of **LuL2$^{\mathbf{MOM}}$** measured in D_2O at 298 K.

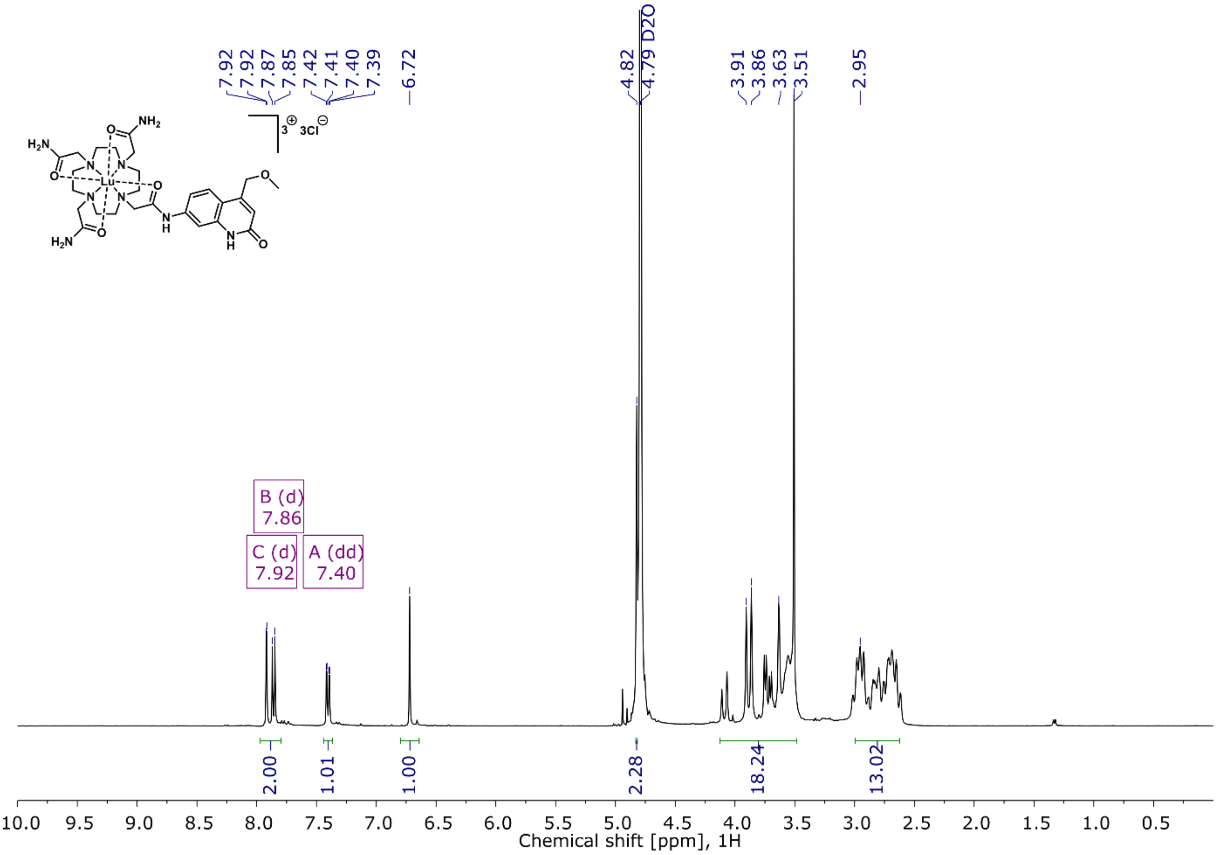

Figure S4. ^{1}H NMR spectrum (400 MHz) of **LuL3a$^{\mathbf{MOM}}$** measured in D_2O at 298 K.

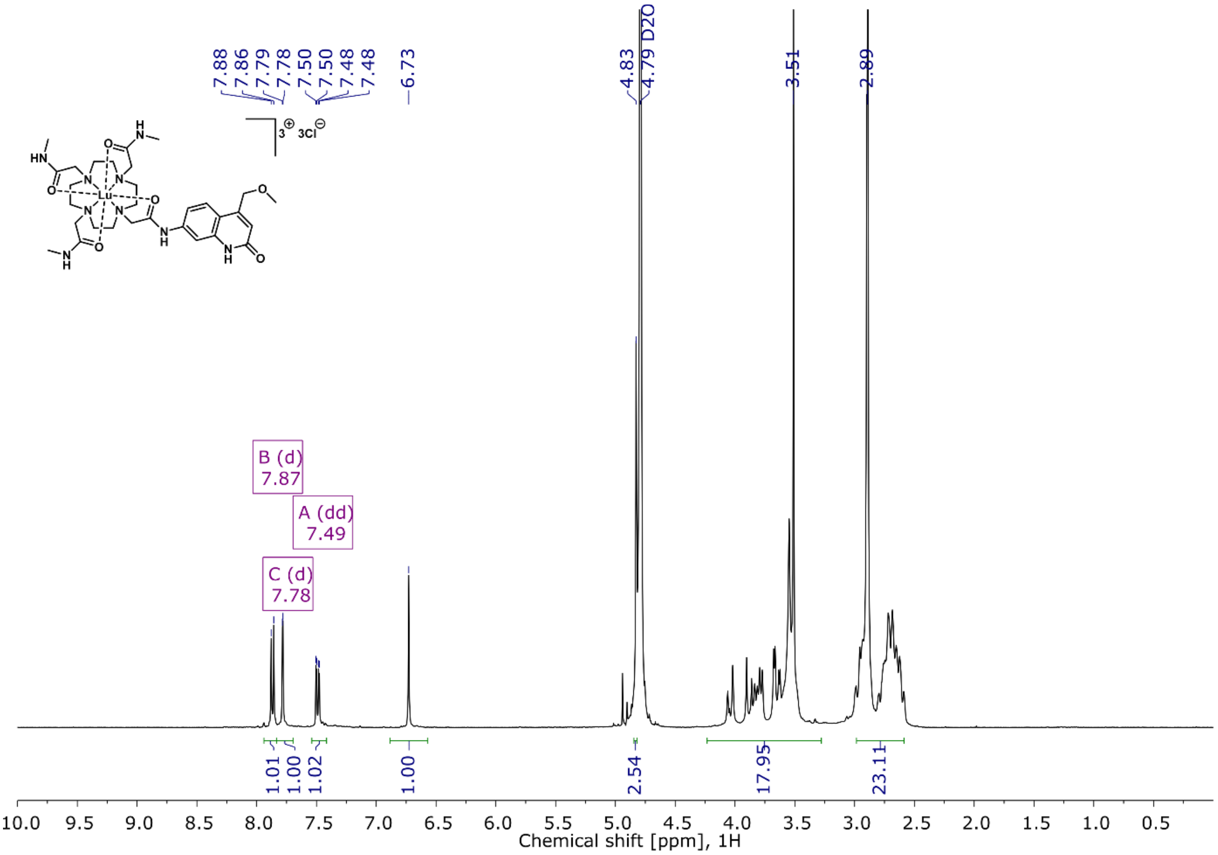

Figure S5. ^{1}H NMR spectrum (400 MHz) of **LuL3b^{MOM}** measured in D_2O at 298 K.

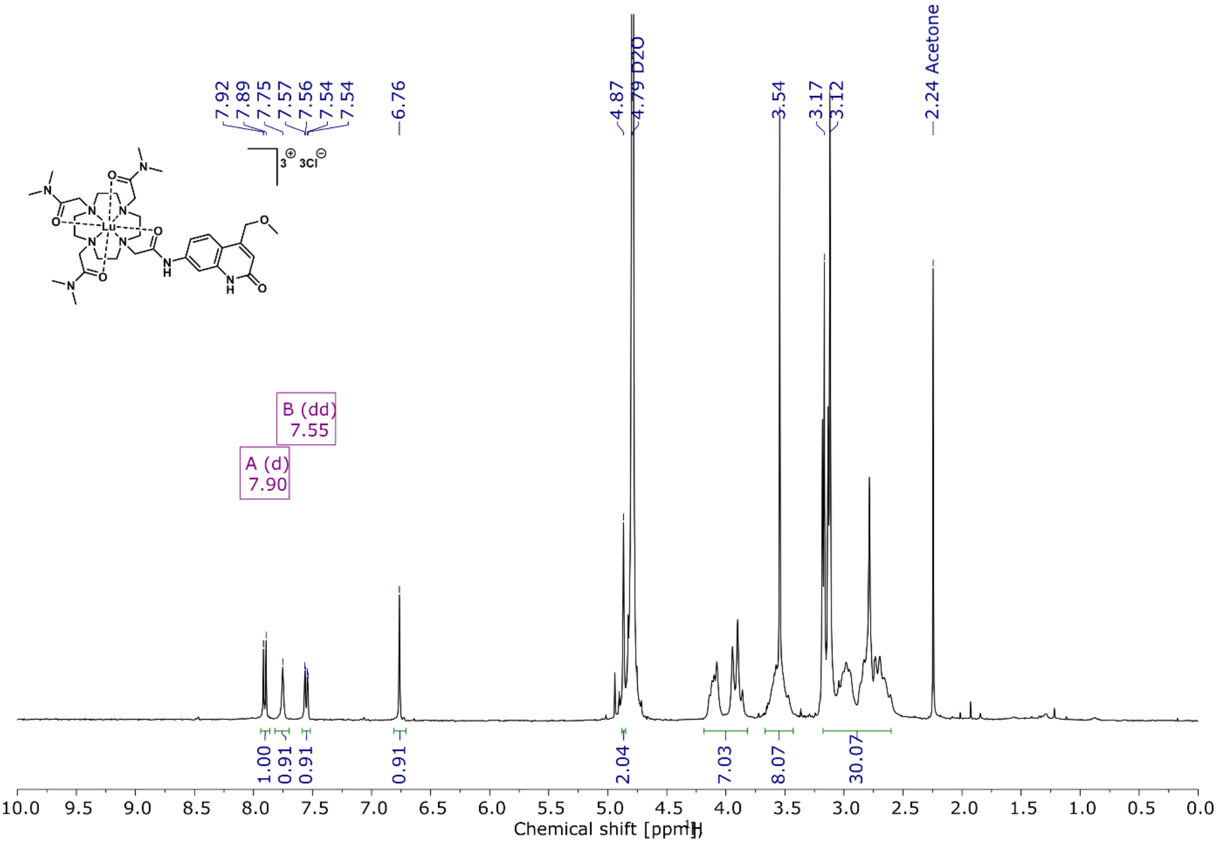

Figure S6. ^{1}H NMR spectrum (400 MHz) of **LuL3c^{MOM}** measured in D_2O at 298 K.

^{1}H NMR spectra of Yb(III) complexes

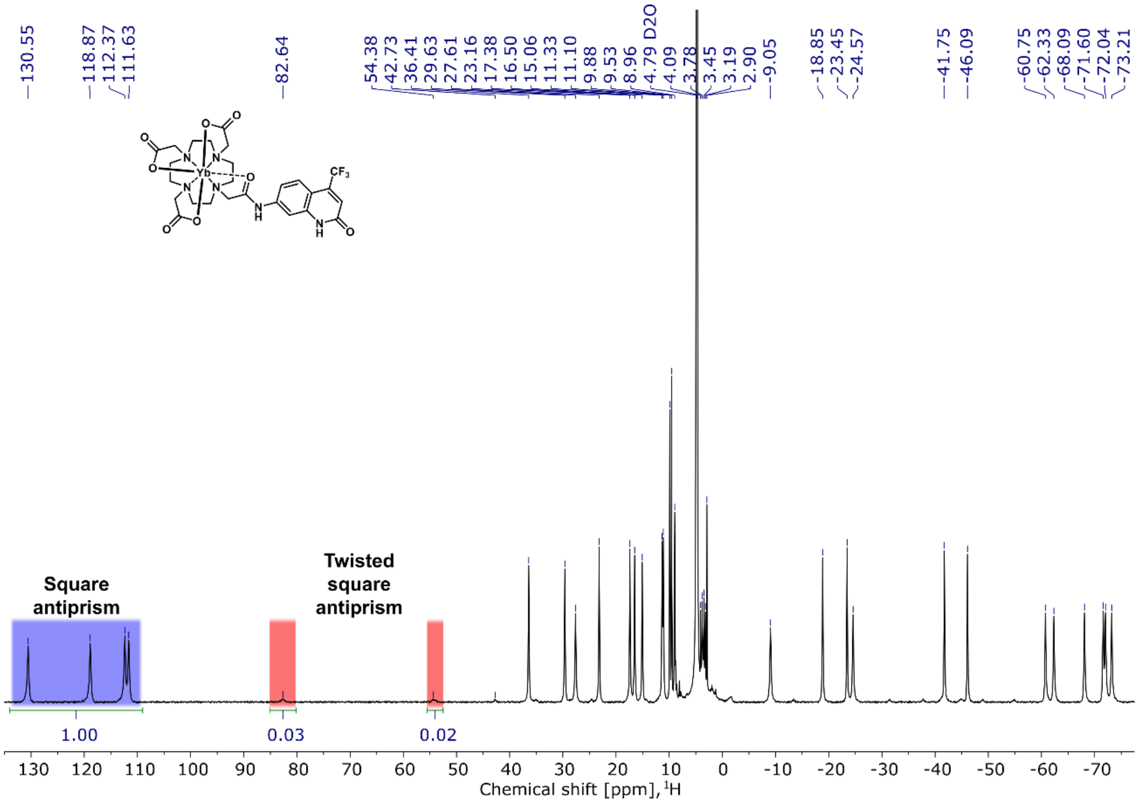

Figure S7. ^{1}H NMR spectrum (400 MHz) of **YbL0^{CF3}** measured in D_2O at 298 K with the regions highlighted in red and blue corresponding to TSAP and SAP cyclen axial ring protons respectively.

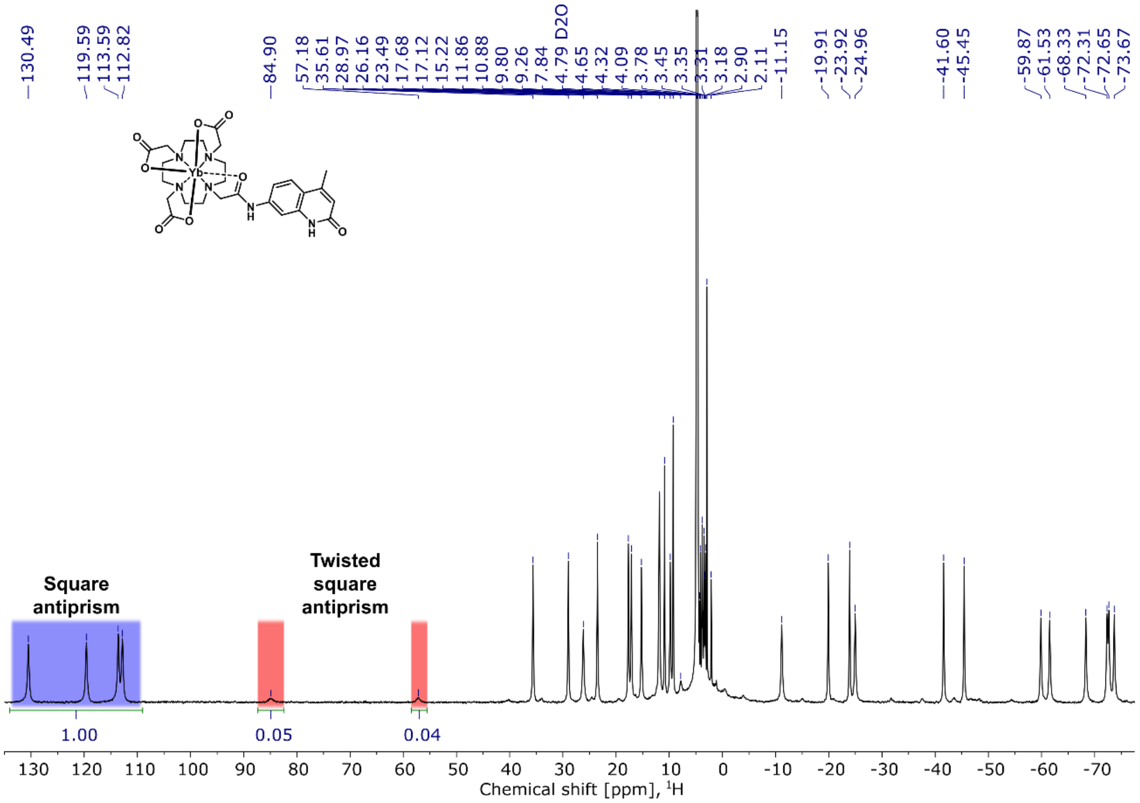

Figure S8. ^{1}H NMR spectrum (400 MHz) of **YbL0^{Me}** measured in D_2O at 298 K with the regions highlighted in red and blue corresponding to TSAP and SAP cyclen axial ring protons respectively.

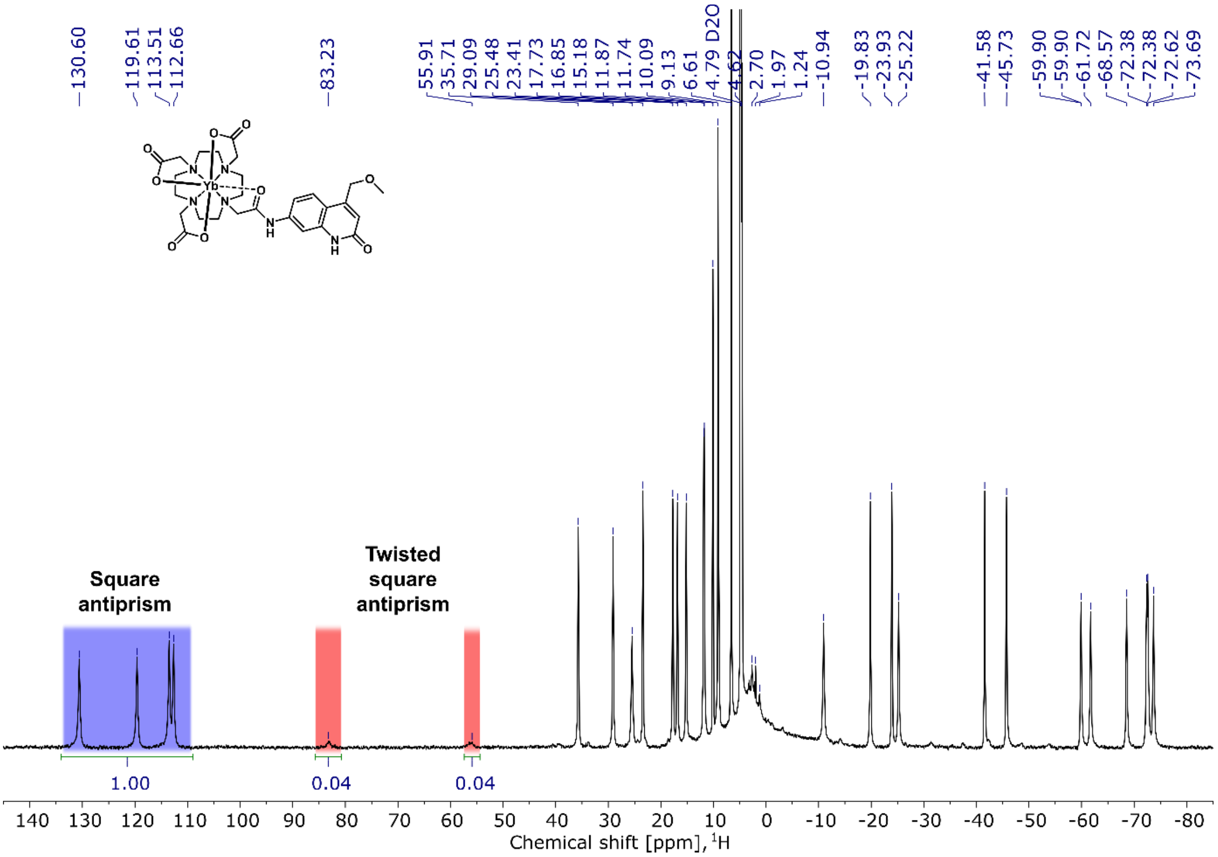

Figure S9. ^{1}H NMR spectrum (400 MHz) of **YbL0^{MOM}** measured in D_2O at 298 K with the regions highlighted in red and blue corresponding to TSAP and SAP cyclen axial ring protons respectively.

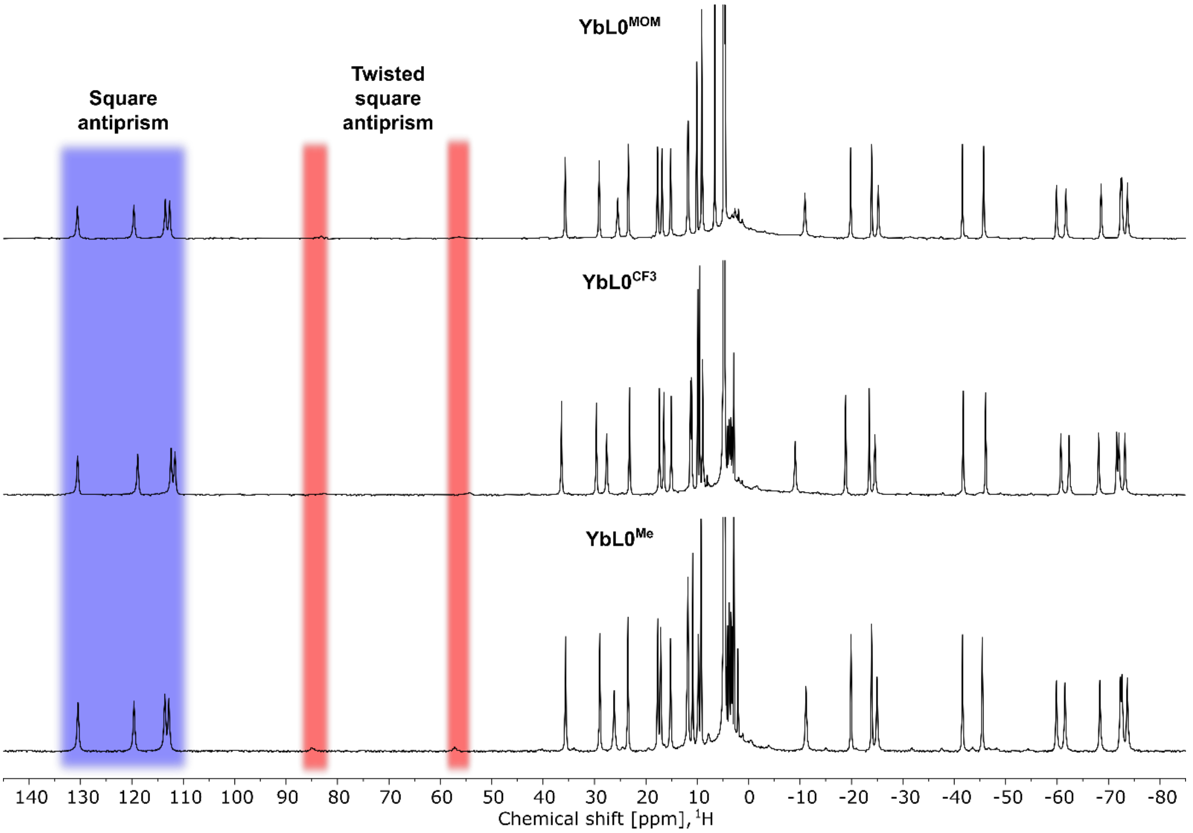

Figure S10. Stacked ^{1}H NMR spectra (400 MHz) of **YbL0** measured in D_2O at 298 K with the regions highlighted in red and blue corresponding to TSAP and SAP cyclen axial ring protons respectively.

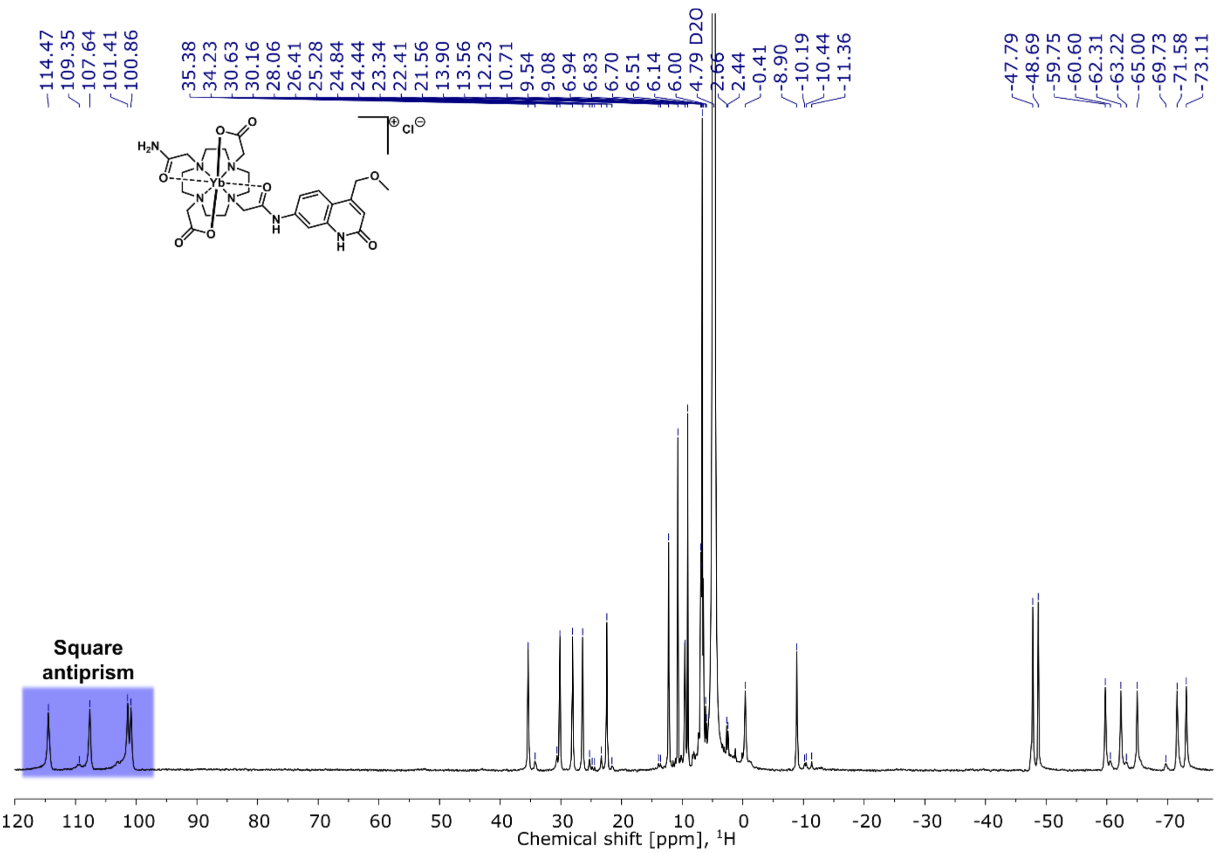

Figure S11. ^{1}H NMR spectrum (400 MHz) of **YbL1^{MOM}** measured in D_2O at 298 K.

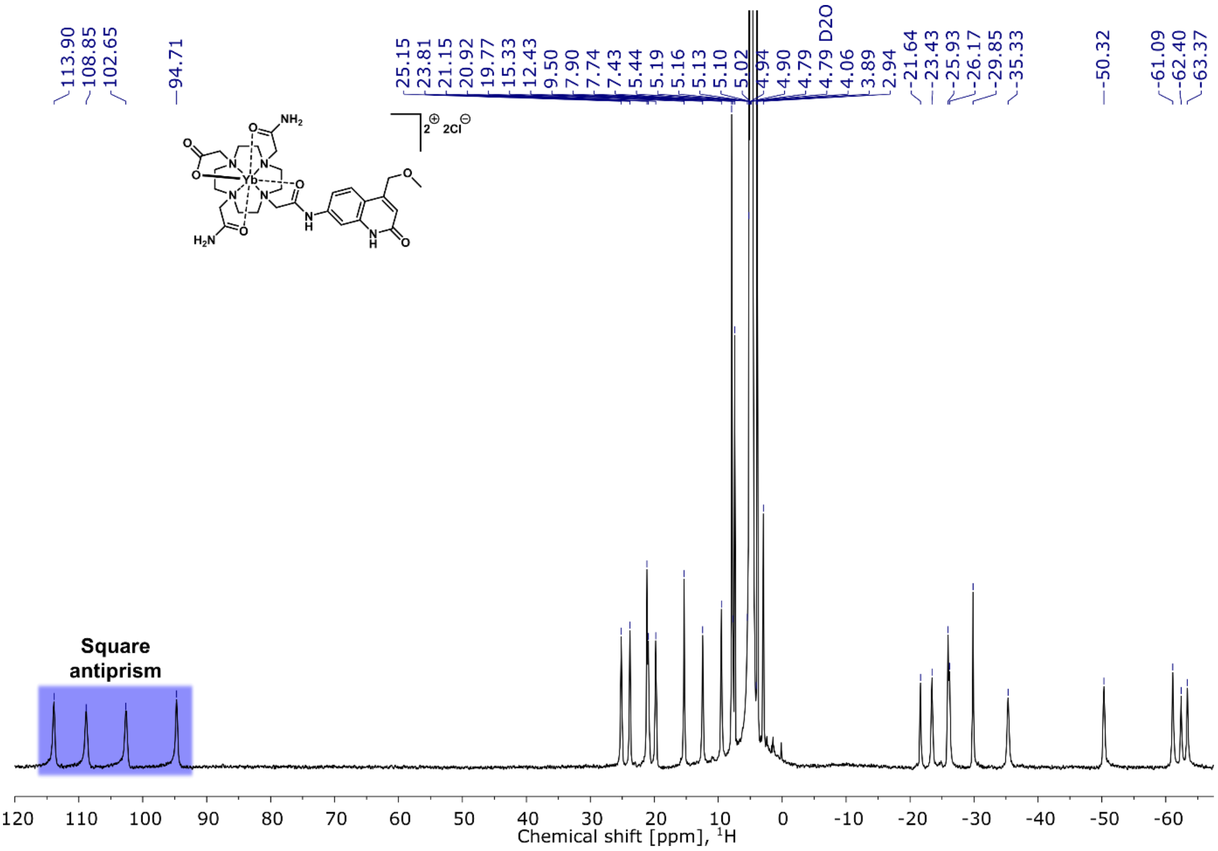

Figure S12. ^{1}H NMR spectrum (400 MHz) of **YbL2^{MOM}** measured in D_2O at 298 K.

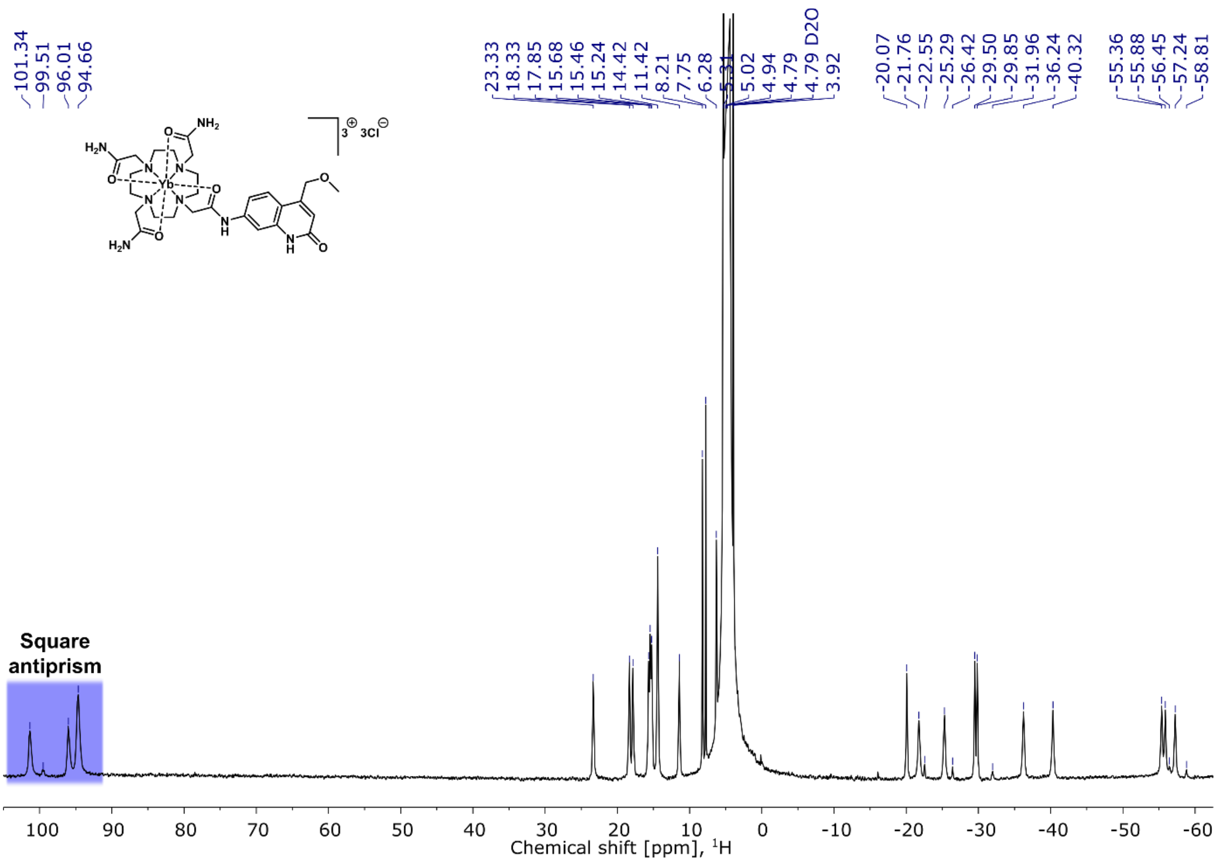

Figure S13. ¹H NMR spectrum (400 MHz) of **YbL3a$^{\mathbf{MOM}}$** measured in D_2O at 298 K.

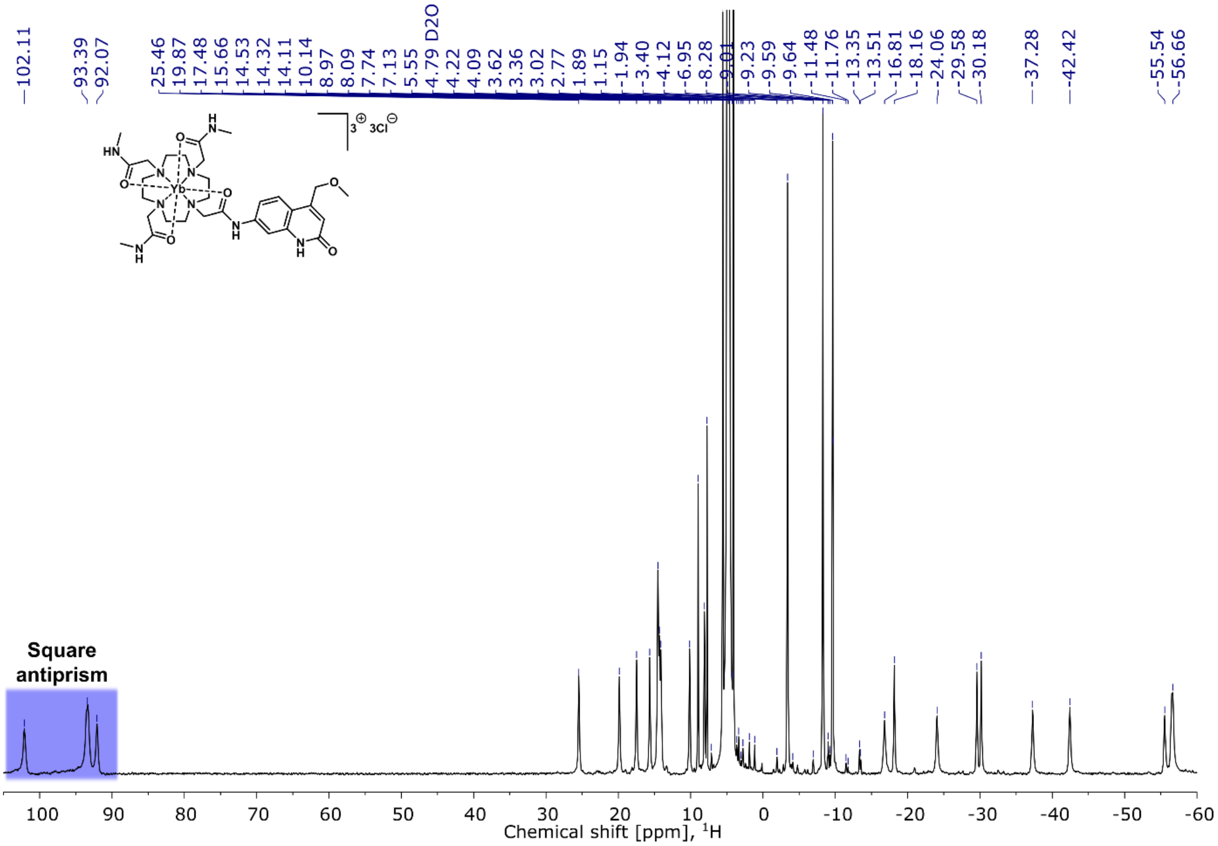

Figure S14. ¹H NMR spectrum (400 MHz) of **YbL3b$^{\mathbf{MOM}}$** measured in D_2O at 298 K.

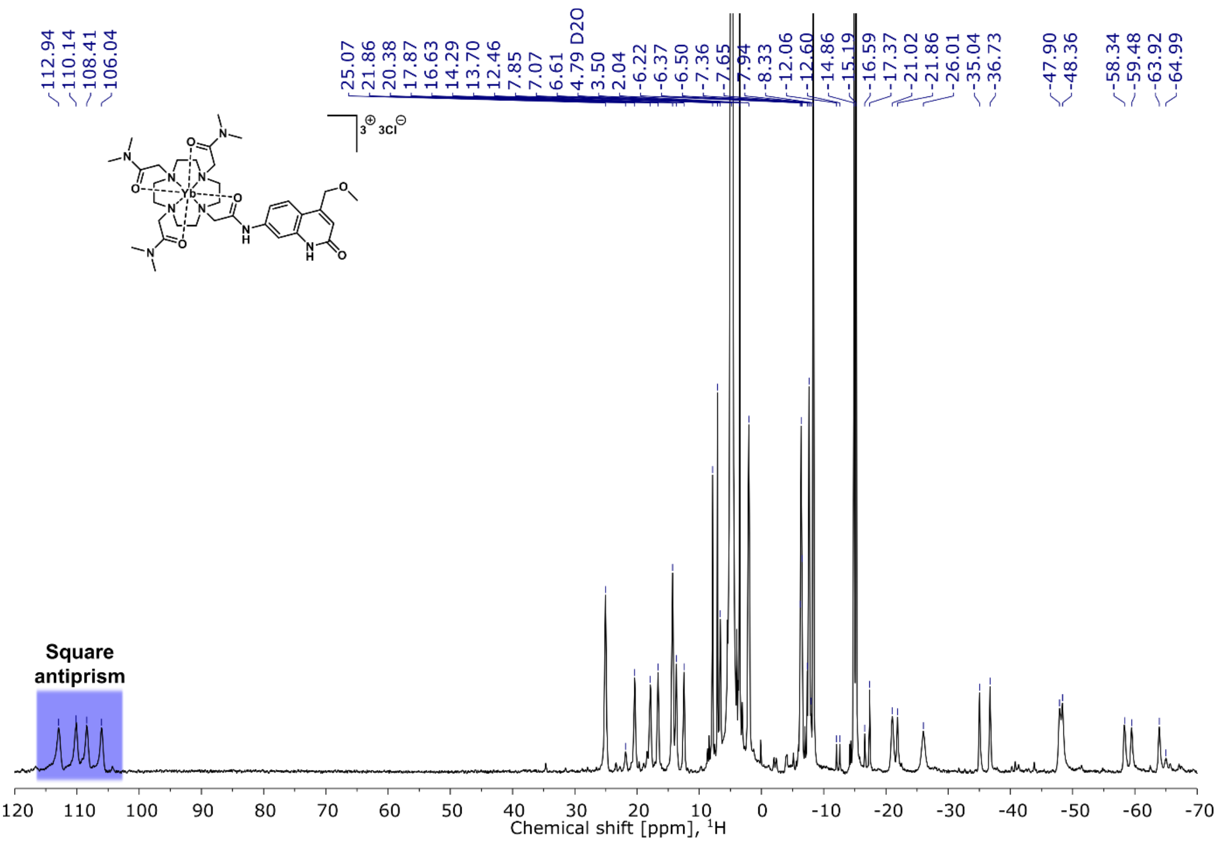

Figure S15. ${}^{1}H$ NMR spectrum (400 MHz) of **YbL3c^{MOM}** measured in D_2O at 298 K.

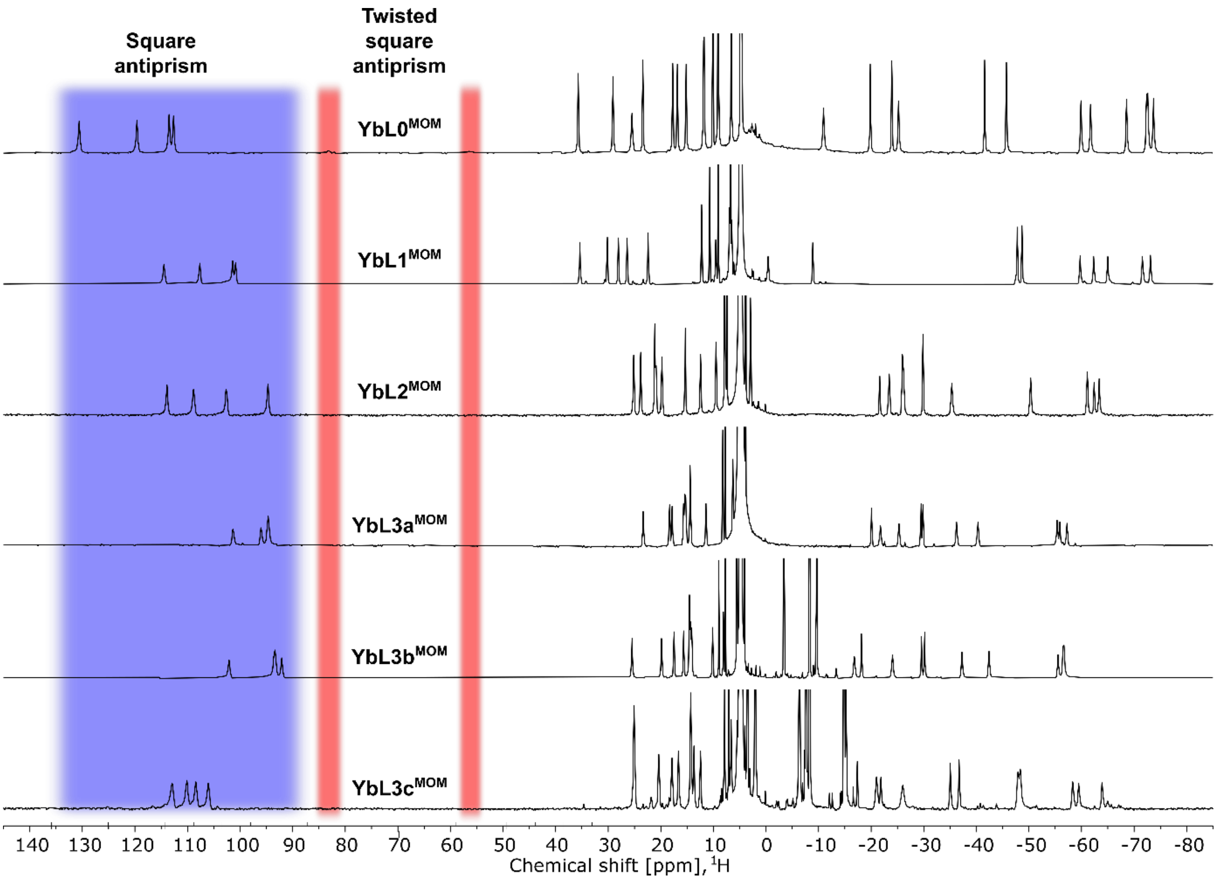

Figure S16. Stacked ${}^{1}H$ NMR spectra (400 MHz) of **YbL^{MOM}** complexes measured in D_2O at 298 K.

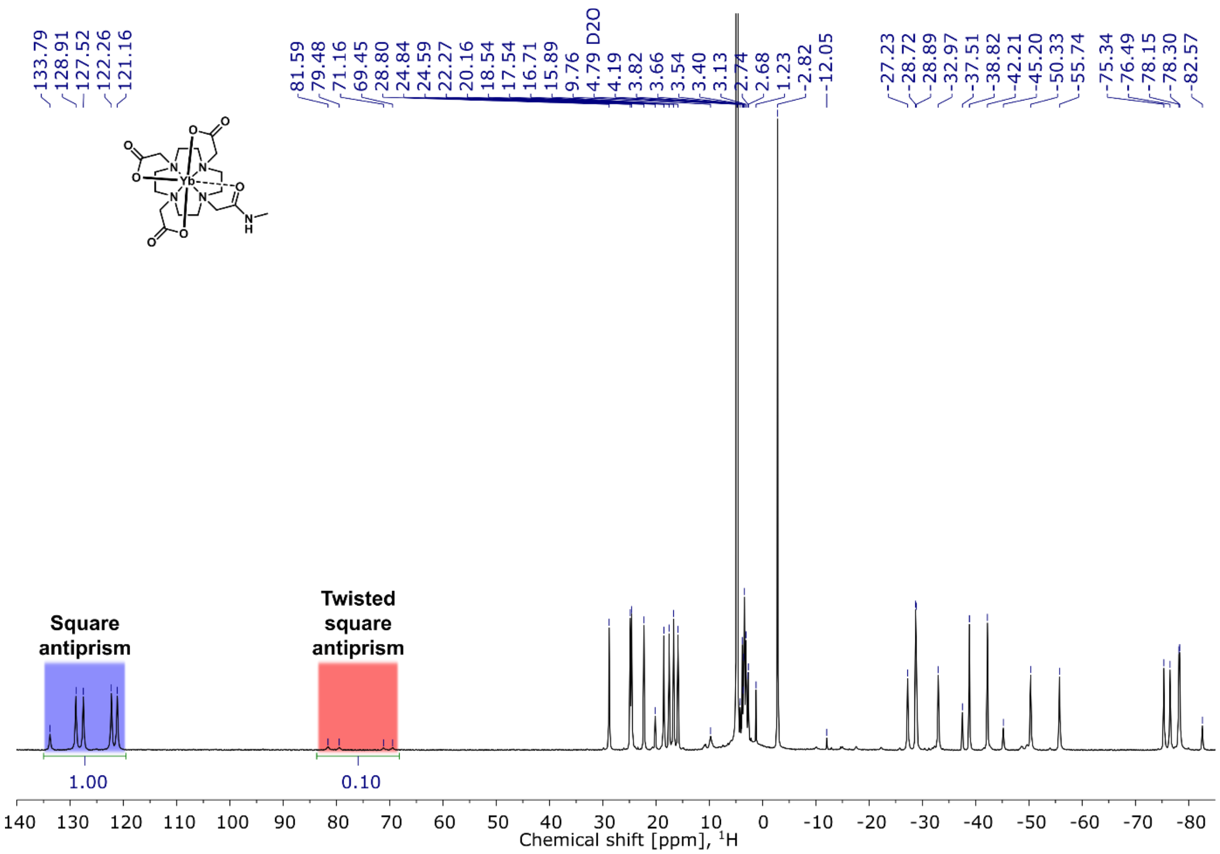

Figure S17. ^{1}H NMR spectrum (400 MHz) of **YbL0^{m}** measured in D_2O at 198 K with the regions highlighted in red and blue corresponding to TSAP and SAP cyclen axial ring protons respectively.

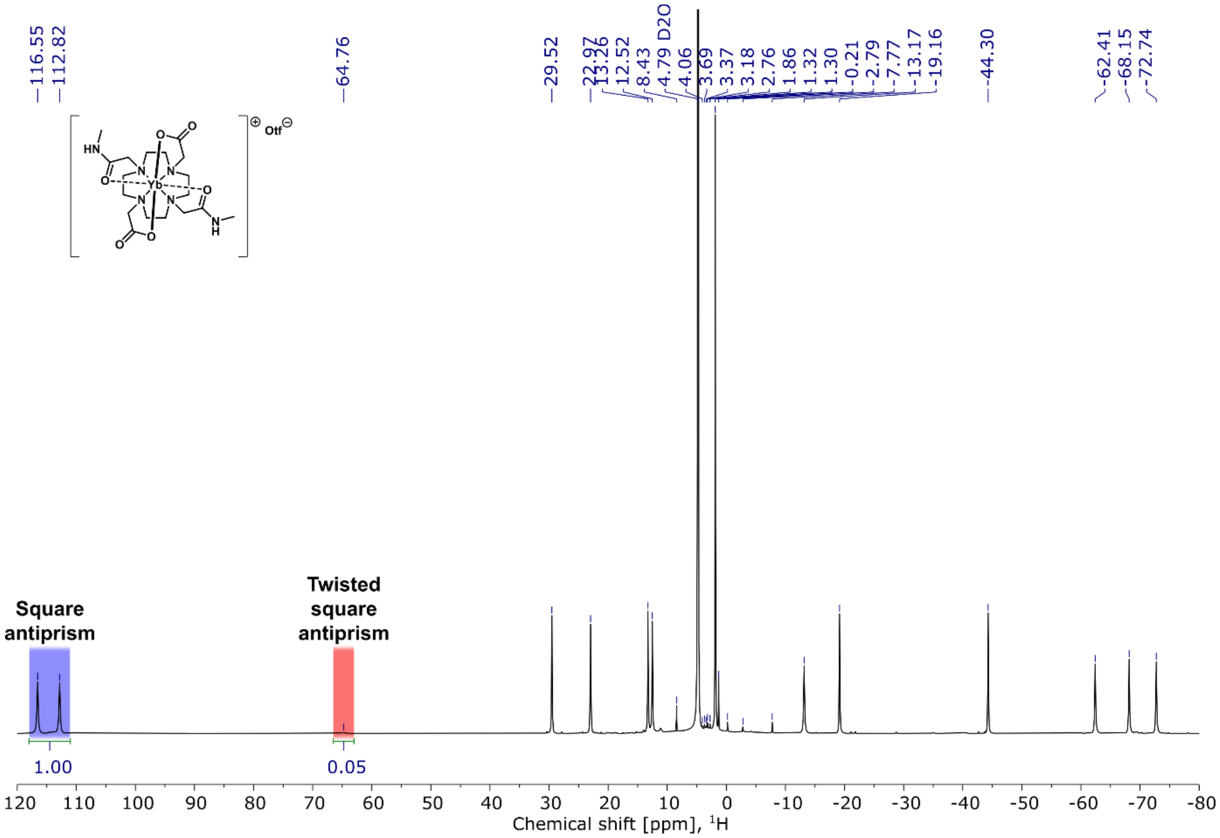

Figure S18. ^{1}H NMR spectrum (400 MHz) of **YbL1^{m}** measured in D_2O at 298 K.

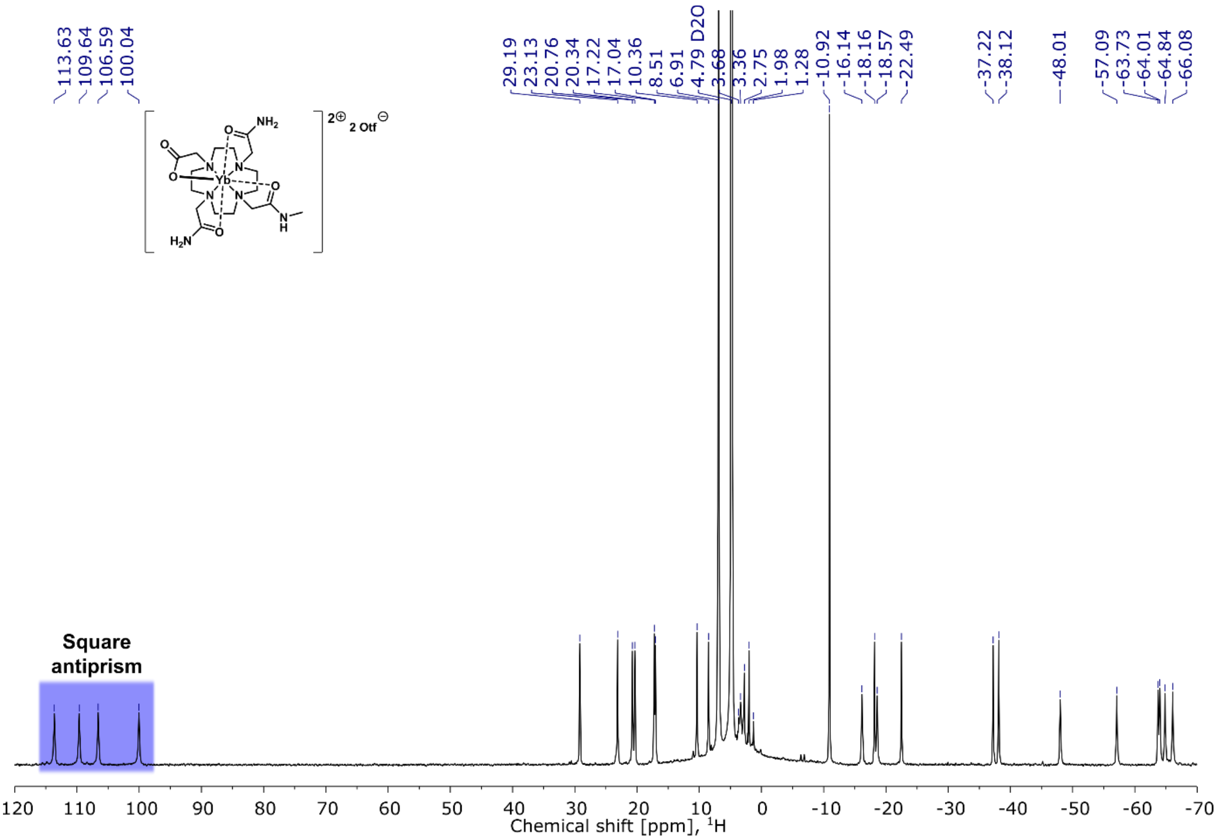

Figure S19. ^{1}H NMR spectrum (400 MHz) of **YbL2[m]** measured in D_2O at 298 K.

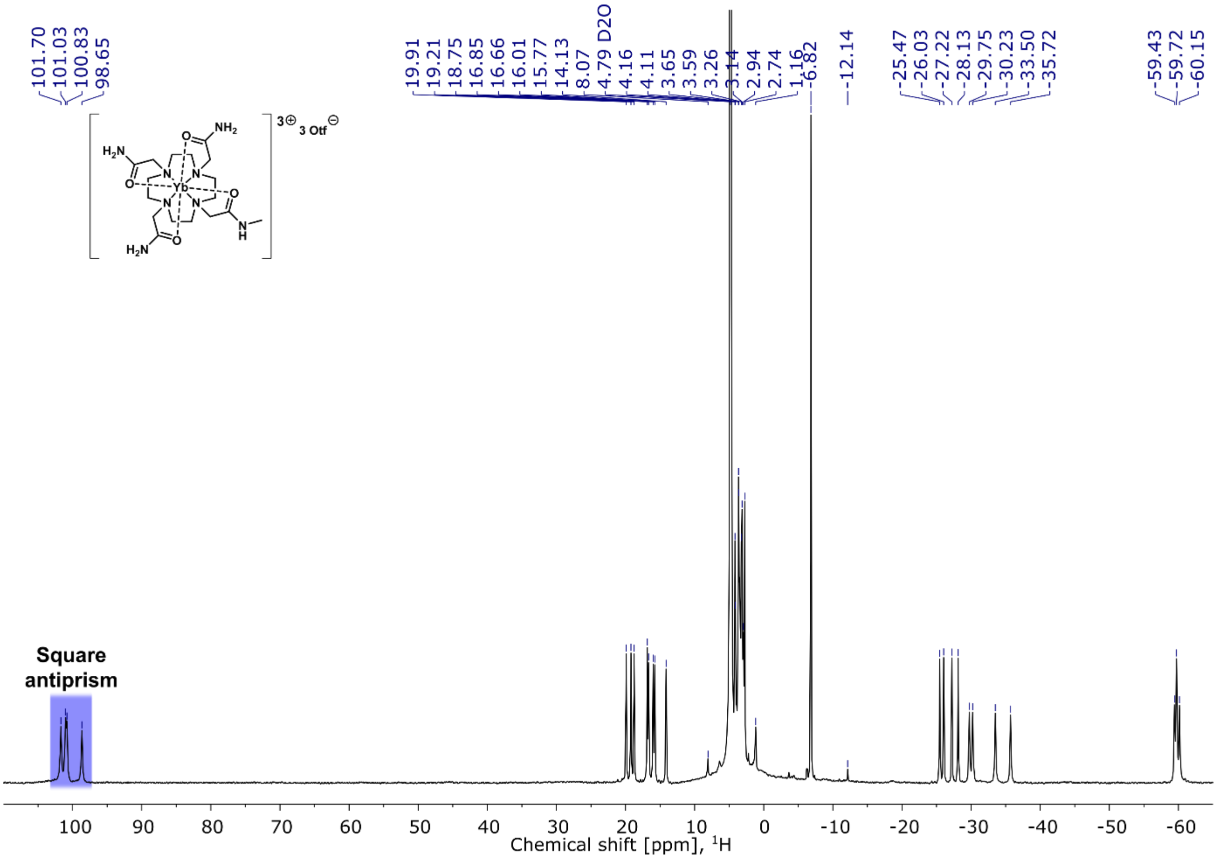

Figure S20. ^{1}H NMR spectrum (400 MHz) of **YbL3a[m]** measured in D_2O at 298 K.

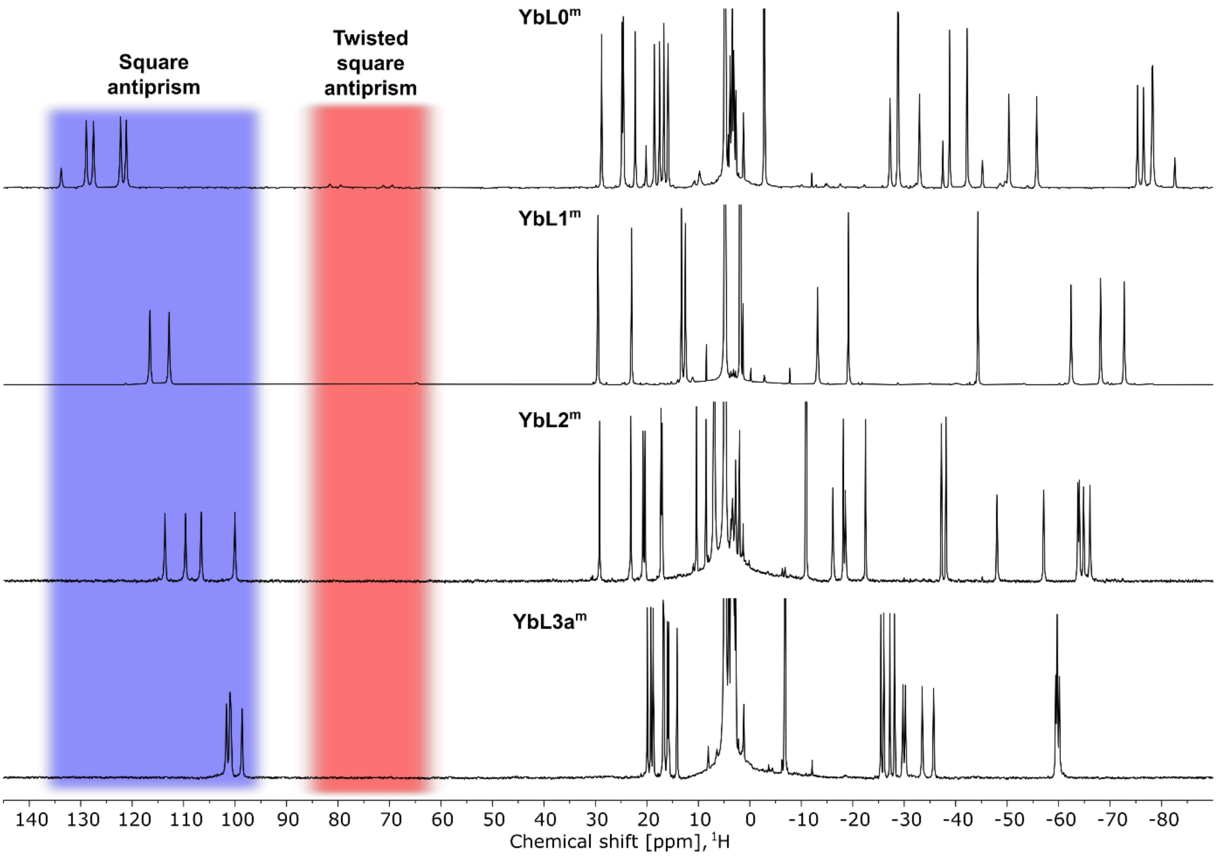

Figure S21. Stacked ^{1}H NMR spectra (400 MHz) of **YbL^m** complexes measured in D_2O at 298 K.

Temperature dependent ^{1}H NMR spectra of Yb(III) complexes

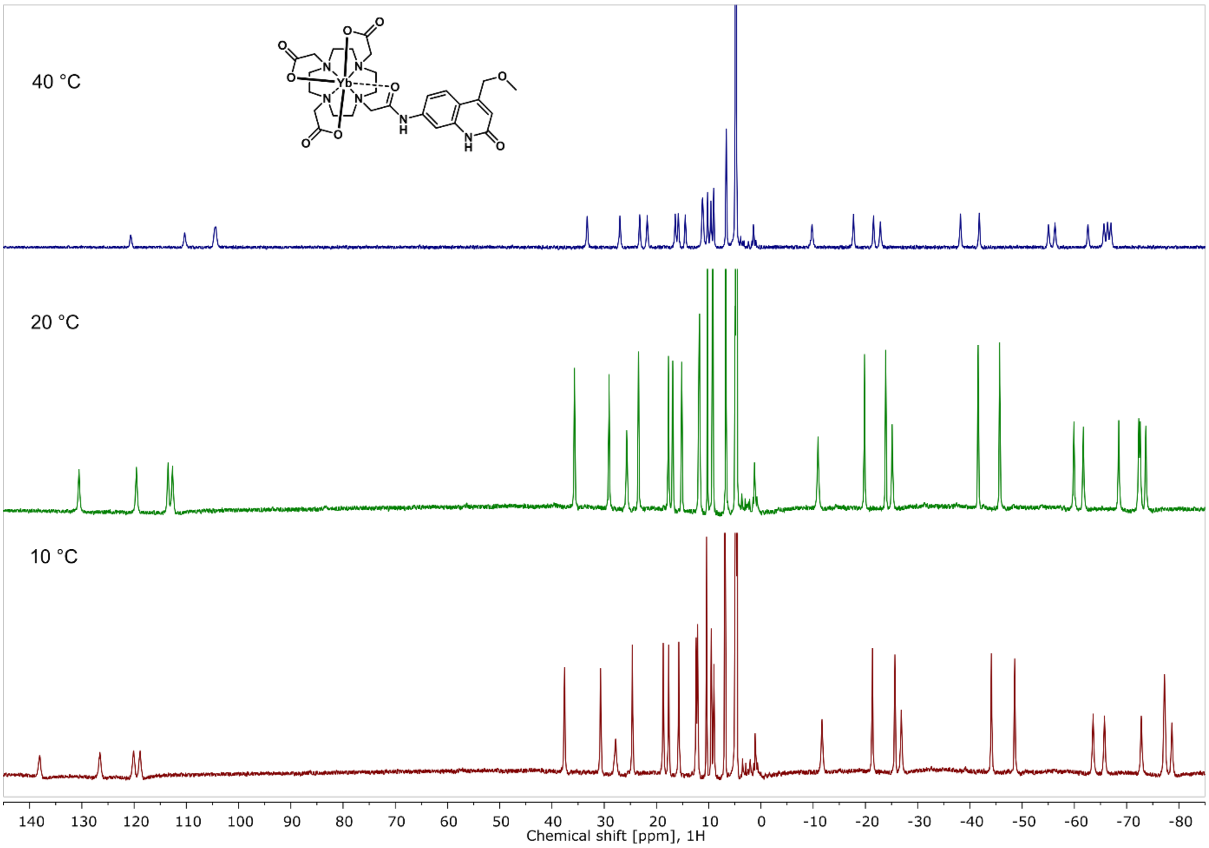

Figure S22. ^{1}H NMR spectra (400 MHz) of **YbL0^{MOM}** recorded at different temperatures.

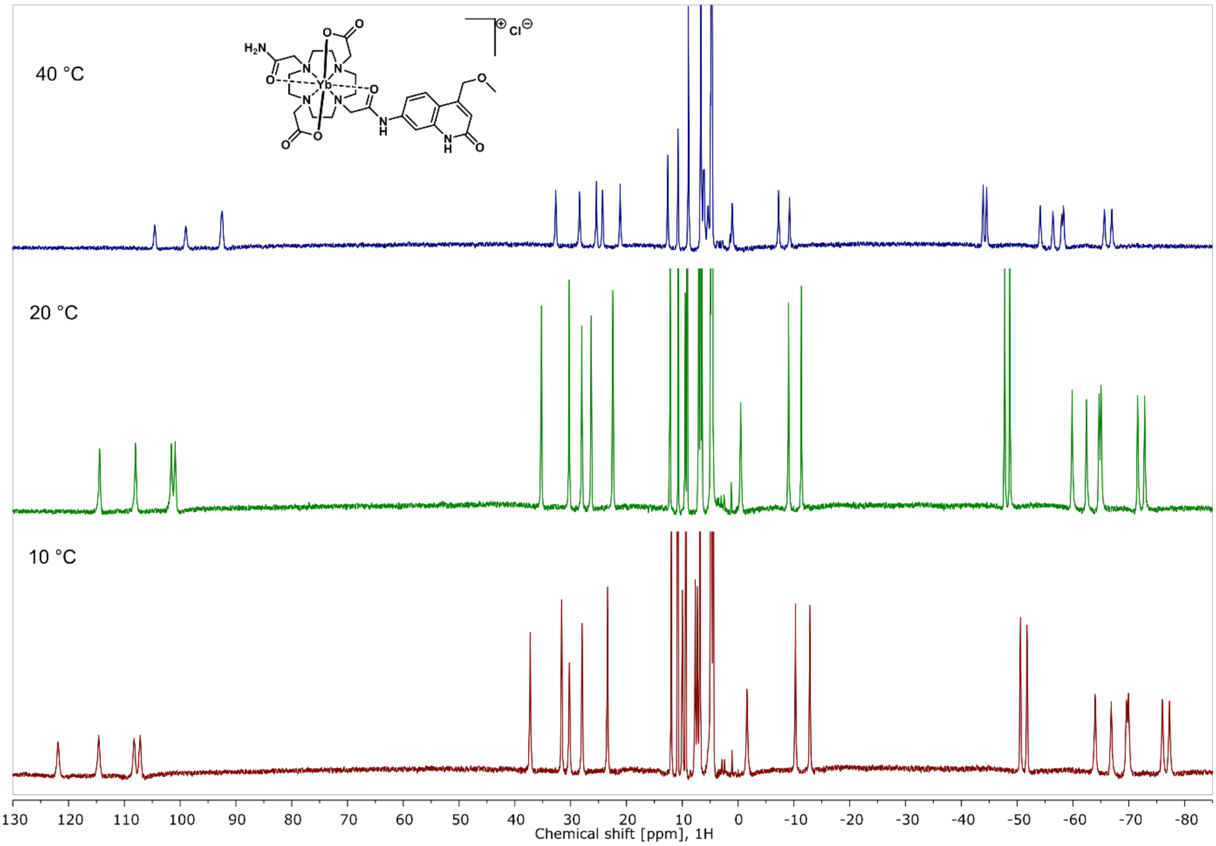

Figure S23. ^{1}H NMR spectra (400 MHz) of **YbL1^{MOM}** recorded at different temperatures.

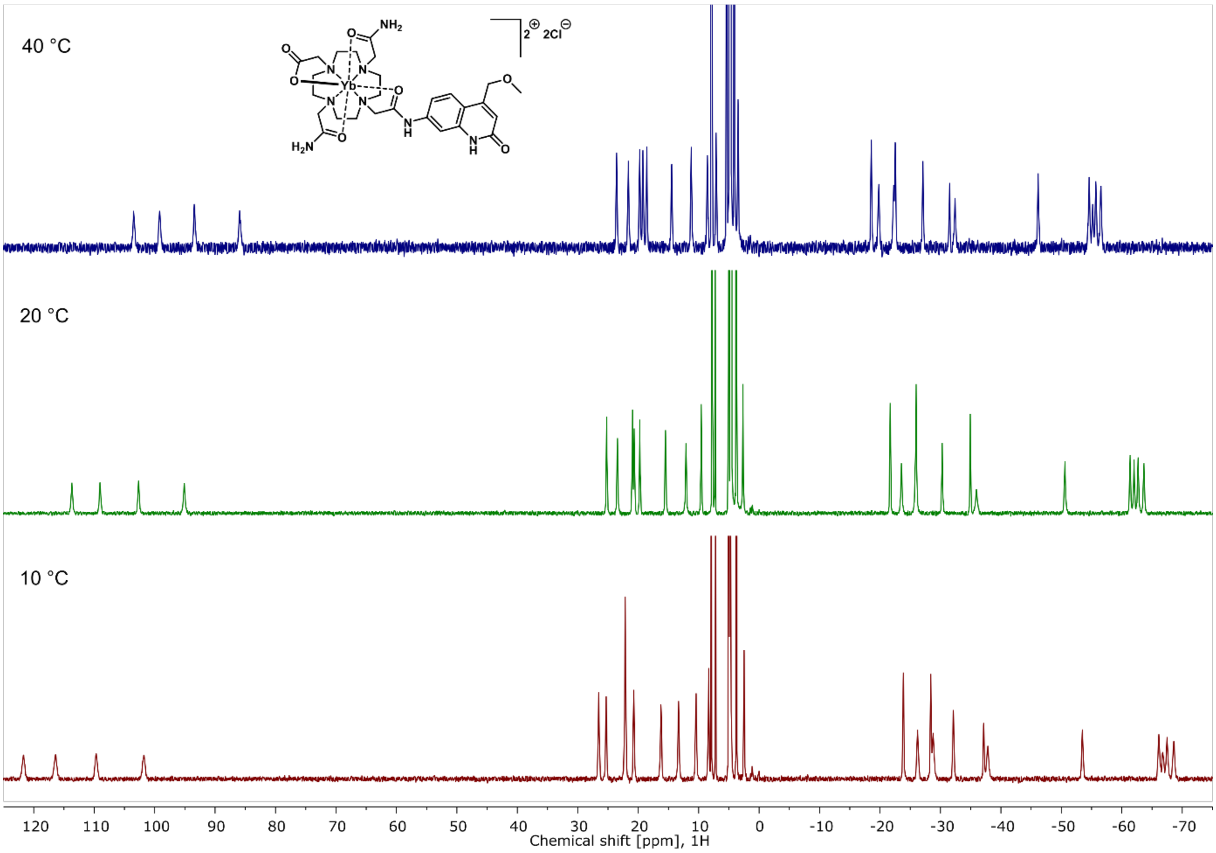

Figure S24. ^{1}H NMR spectra (400 MHz) of **YbL2^{MOM}** recorded at different temperatures.

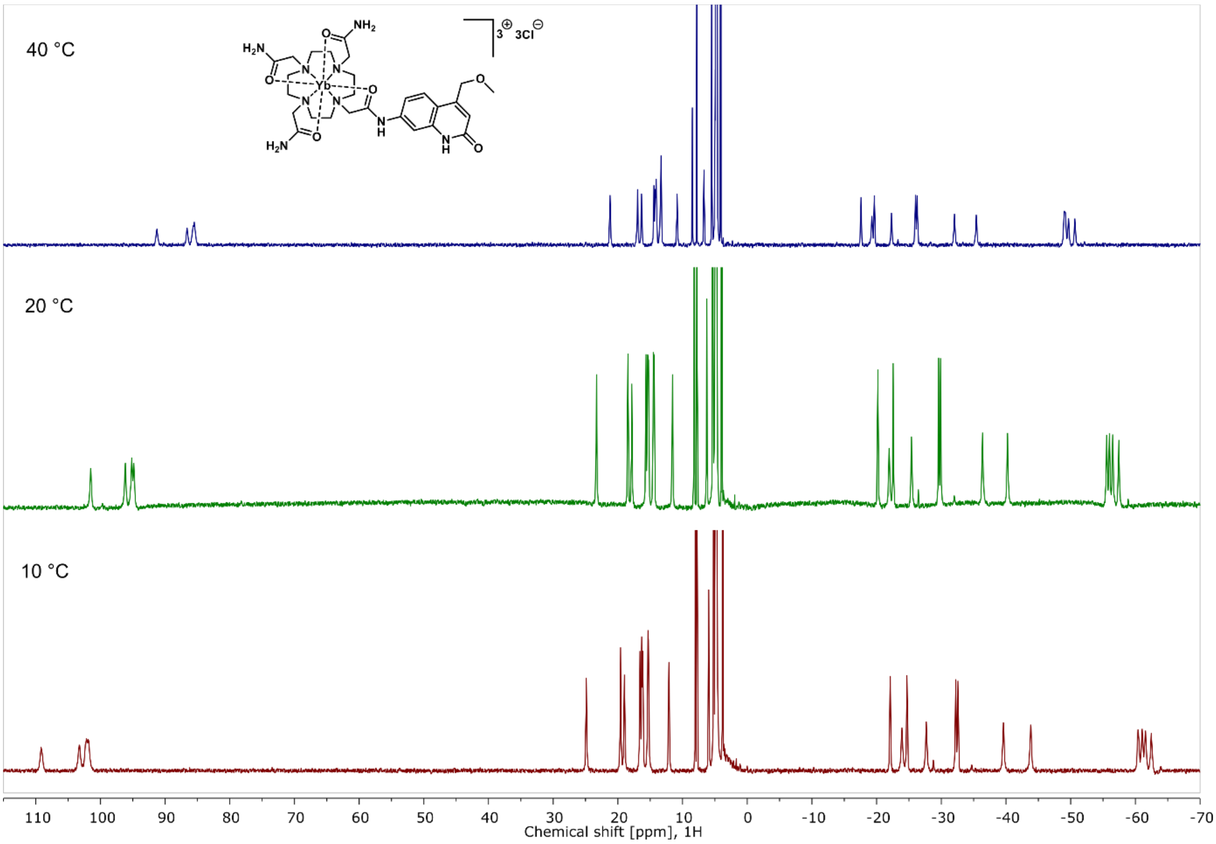

Figure S25. ^{1}H NMR spectra (400 MHz) of **YbL3a^{MOM}** recorded at different temperatures.

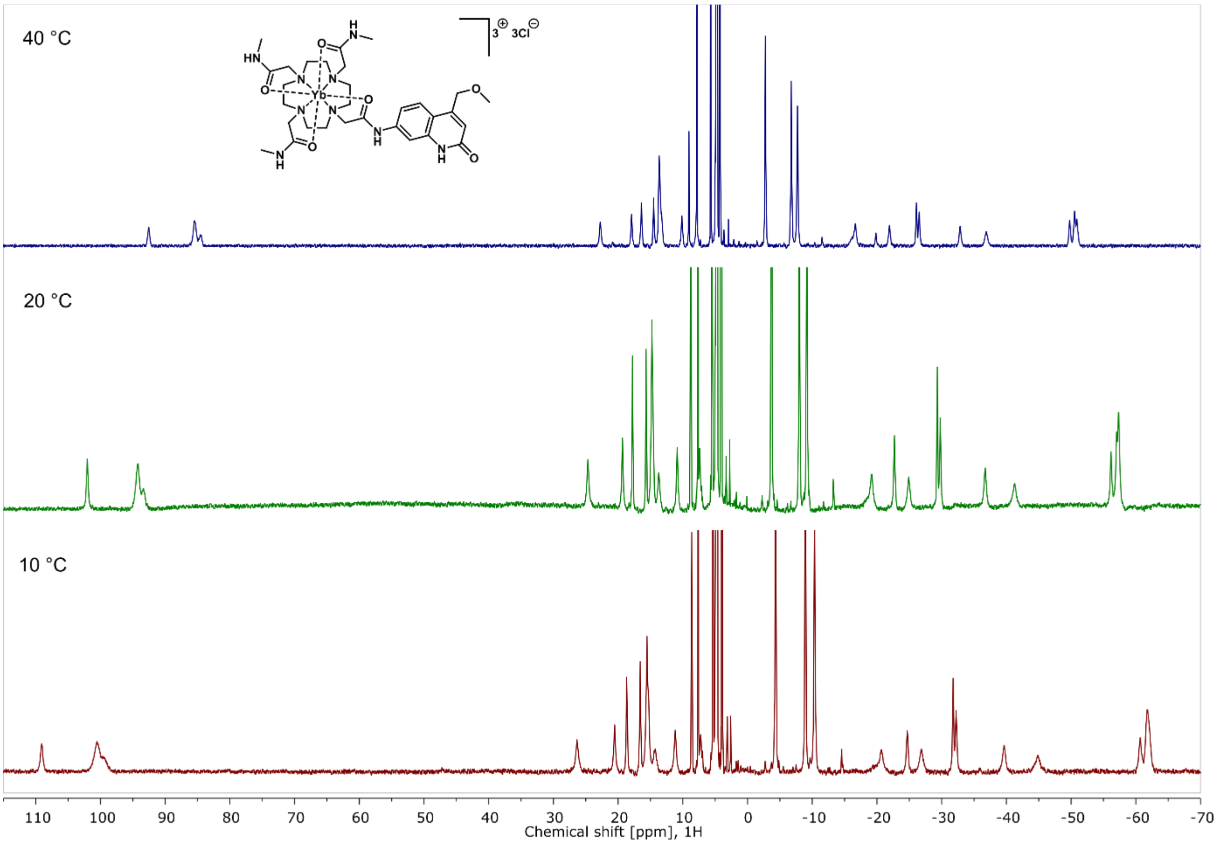

Figure S26. ^{1}H NMR spectra (400 MHz) of **YbL3b^{MOM}** recorded at different temperatures.

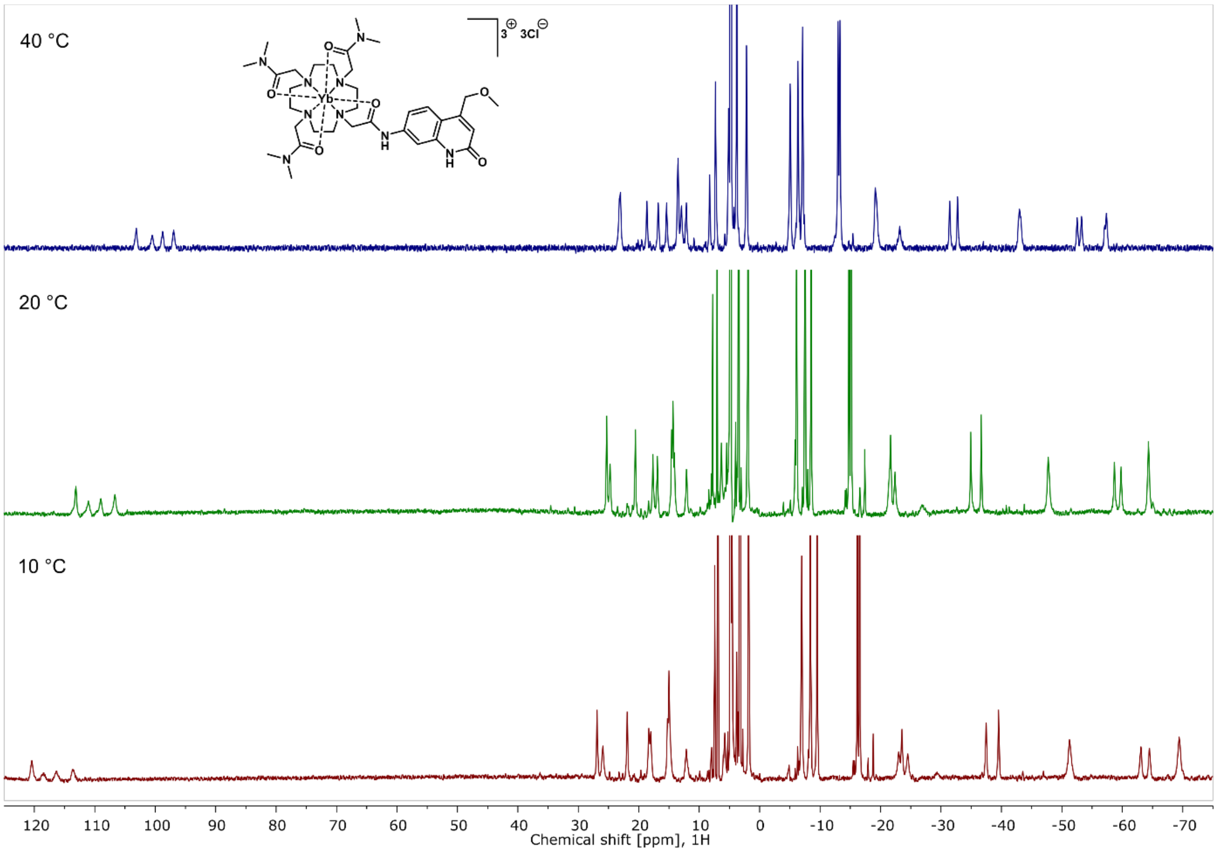

Figure S27. ^{1}H NMR spectra (400 MHz) of **YbL3c^{MOM}** recorded at different temperatures.

NMR characterization of YbL0^{MOM} complex

1D (^{1}H, ^{13}C{^{1}H}) and 2D (COSY, HSQC, EXSY) NMR spectroscopy experiments were recorded for **YbL0^{MOM}**.

^{1}H NMR spectrum display two sets of signals in the range 140−110 ppm and 90−50 ppm that can be attributed to the cyclen axial protons of the SAP and TSAP isomers, respectively, as previously described for similar Yb-complexes.[8, 12-18] EXSY data are consistent with this attribution, as evidenced by correlations between the two set of peaks.[16, 19-22] In addition, variable temperature NMR spectra were recorded. The results show that upon increasing the temperature from 283 K to 353 K the peaks corresponding to the minor species (TSAP) broaden and their integrations increase relative to the major species (SAP). This indicates that the exchange rate between the SAP and TSAP isomer increases.

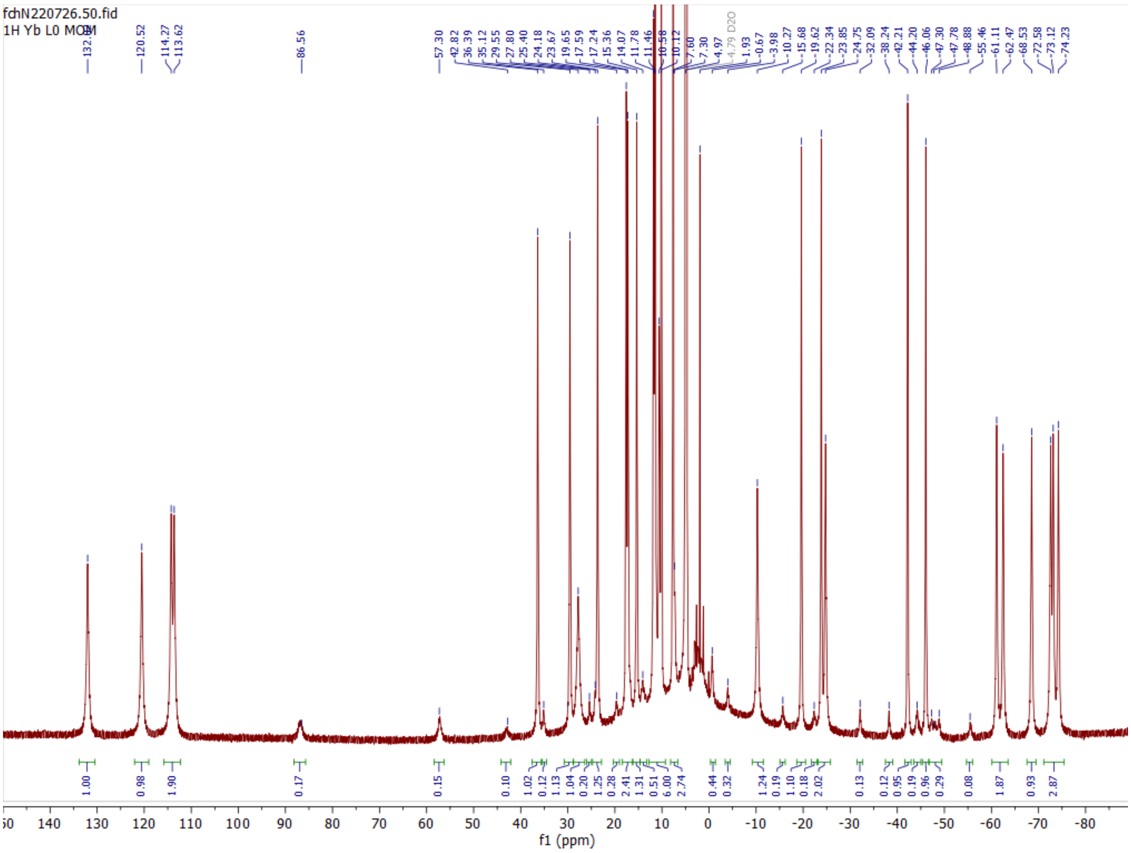

Figure S28. ^{1}H NMR spectrum (600 MHz) of **YbL0^{MOM}** measured in D_2O at 298 K.

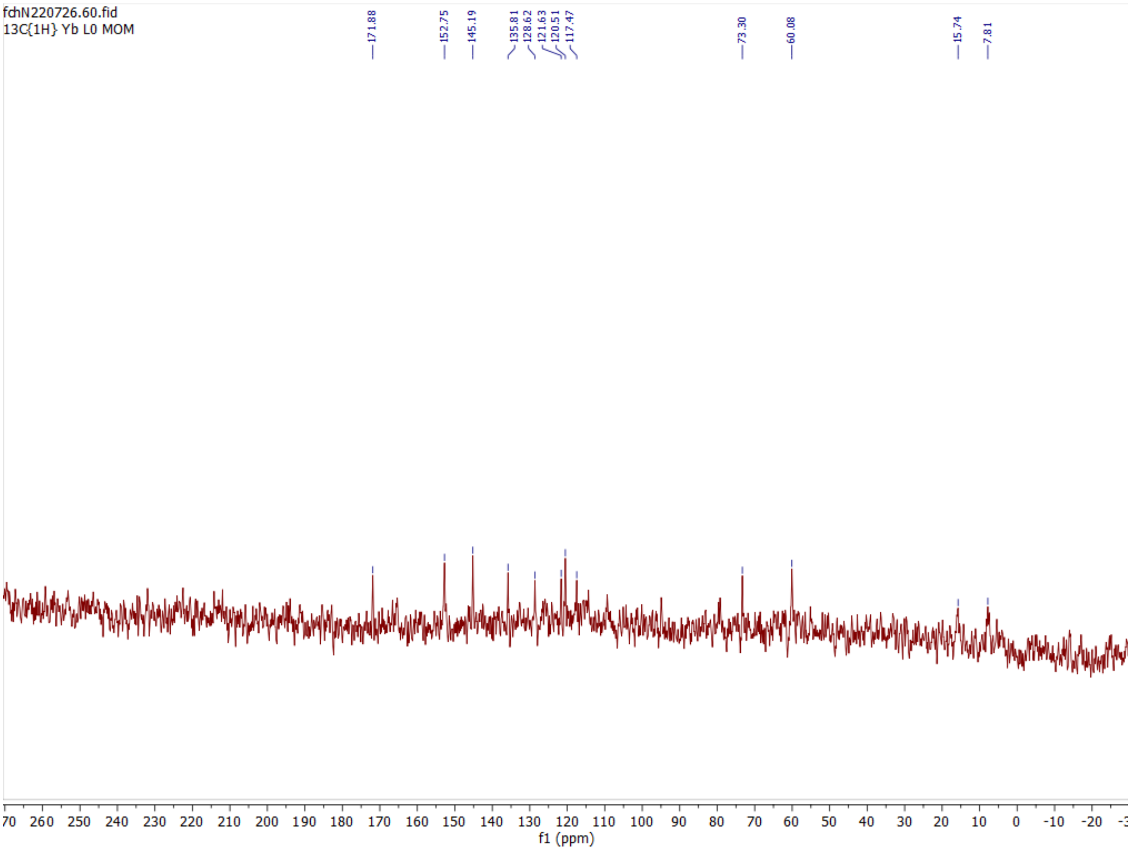

Figure S29. ^{13}C NMR spectrum (151 MHz) of **YbL0^{MOM}** measured in D_2O at 298 K.

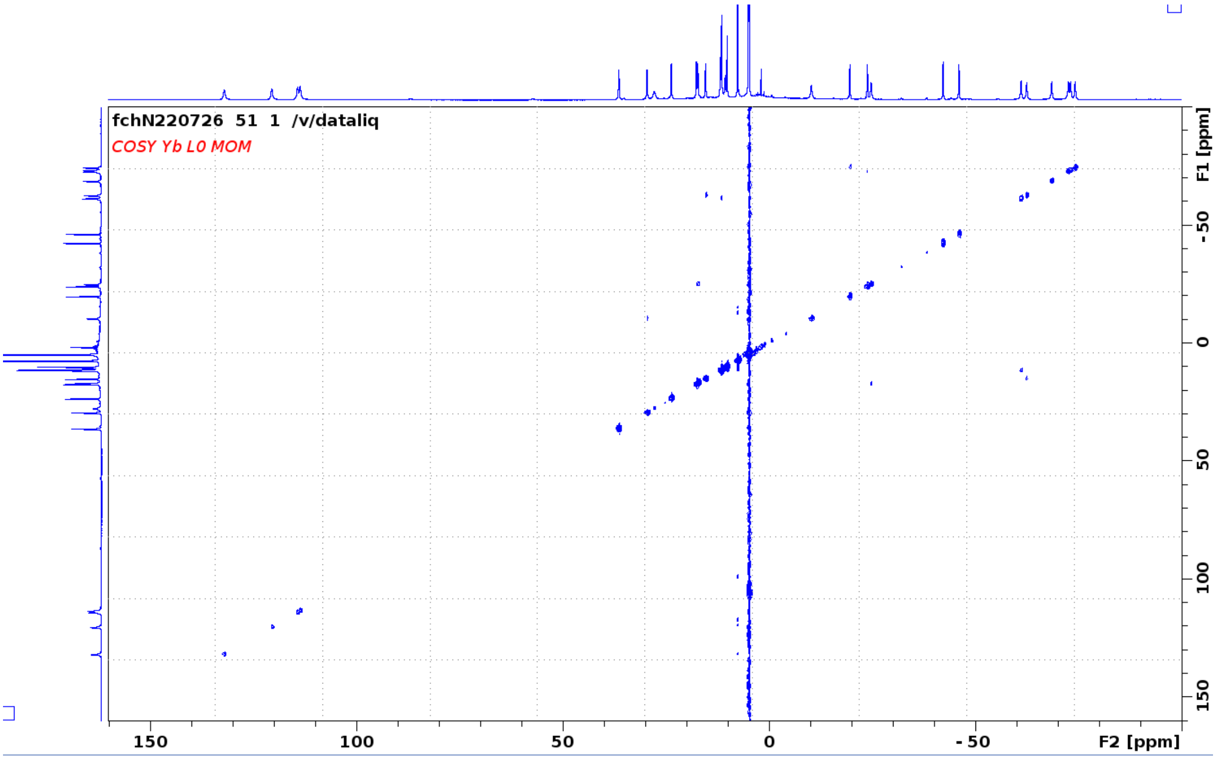

Figure S30. COSY NMR spectrum of **YbL0^{MOM}** measured in D_2O at 298 K.

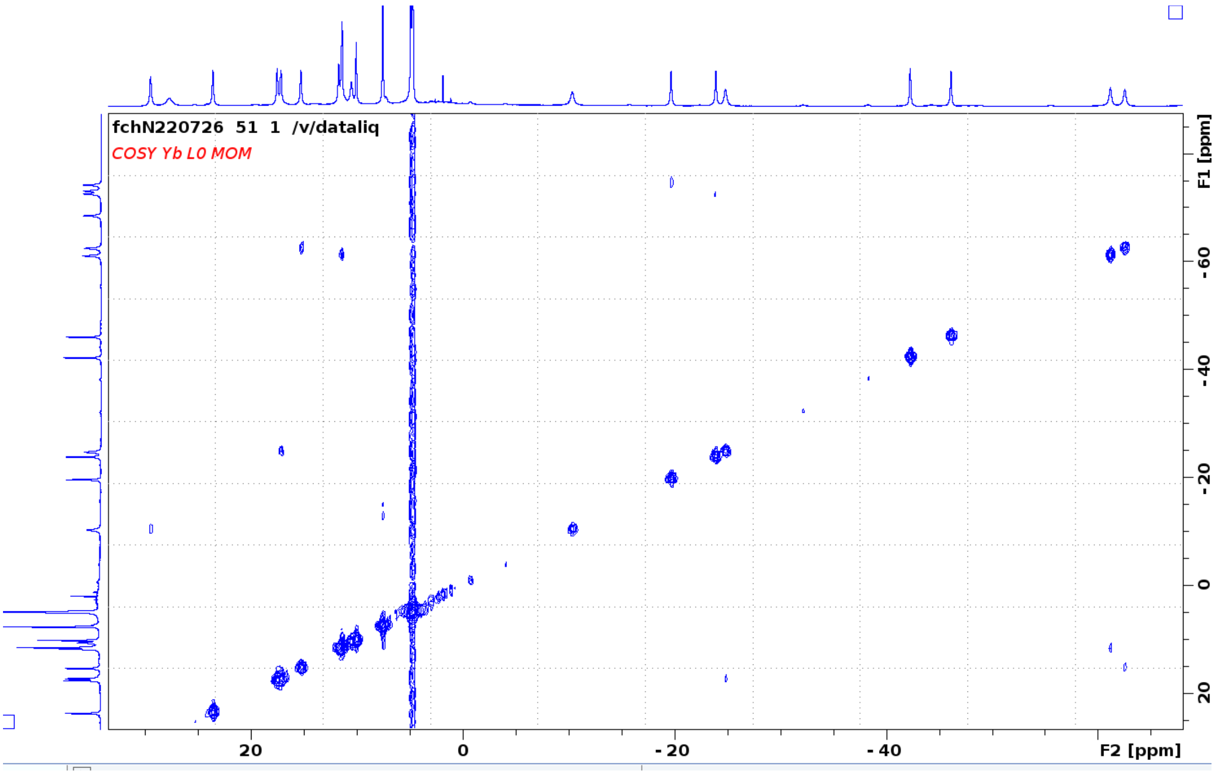

Figure S31. Zoom of the COSY NMR spectrum of **YbL0**$^{\mathbf{MOM}}$ measured in D_2O at 298 K.

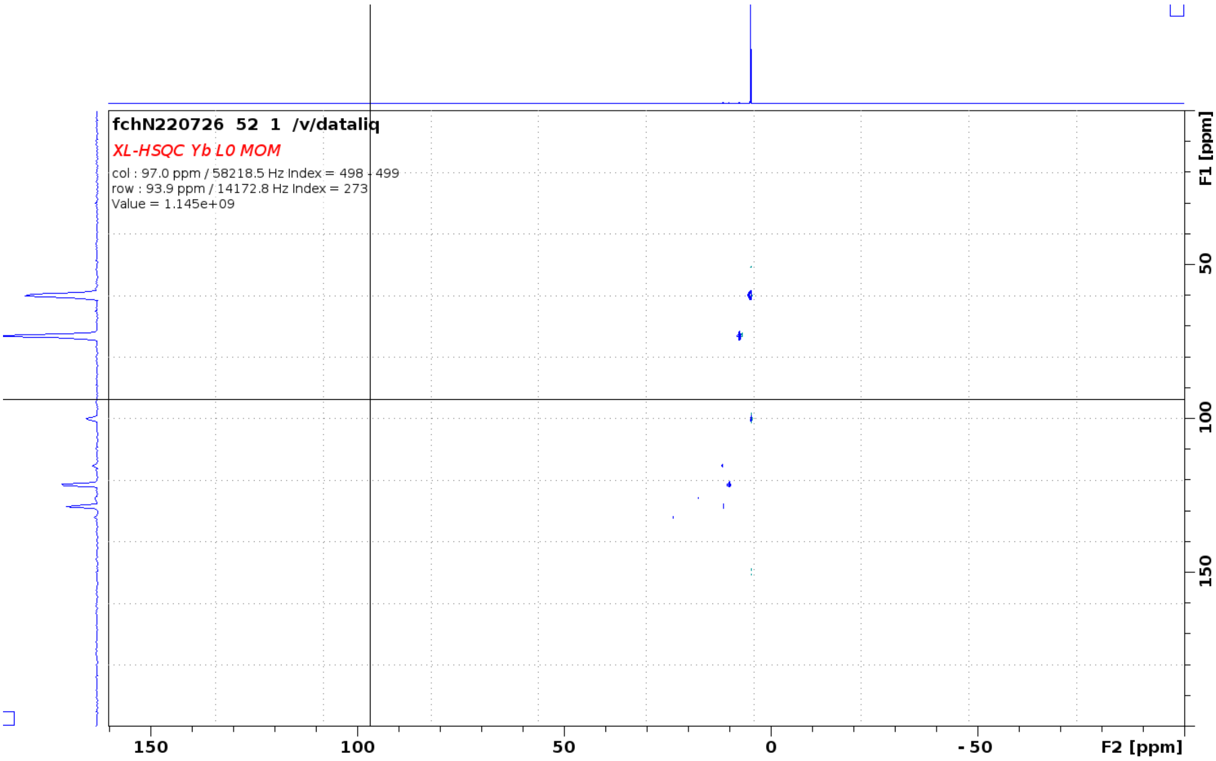

Figure S32. HSQC NMR spectrum of **YbL0**$^{\mathbf{MOM}}$ measured in D_2O at 298 K.

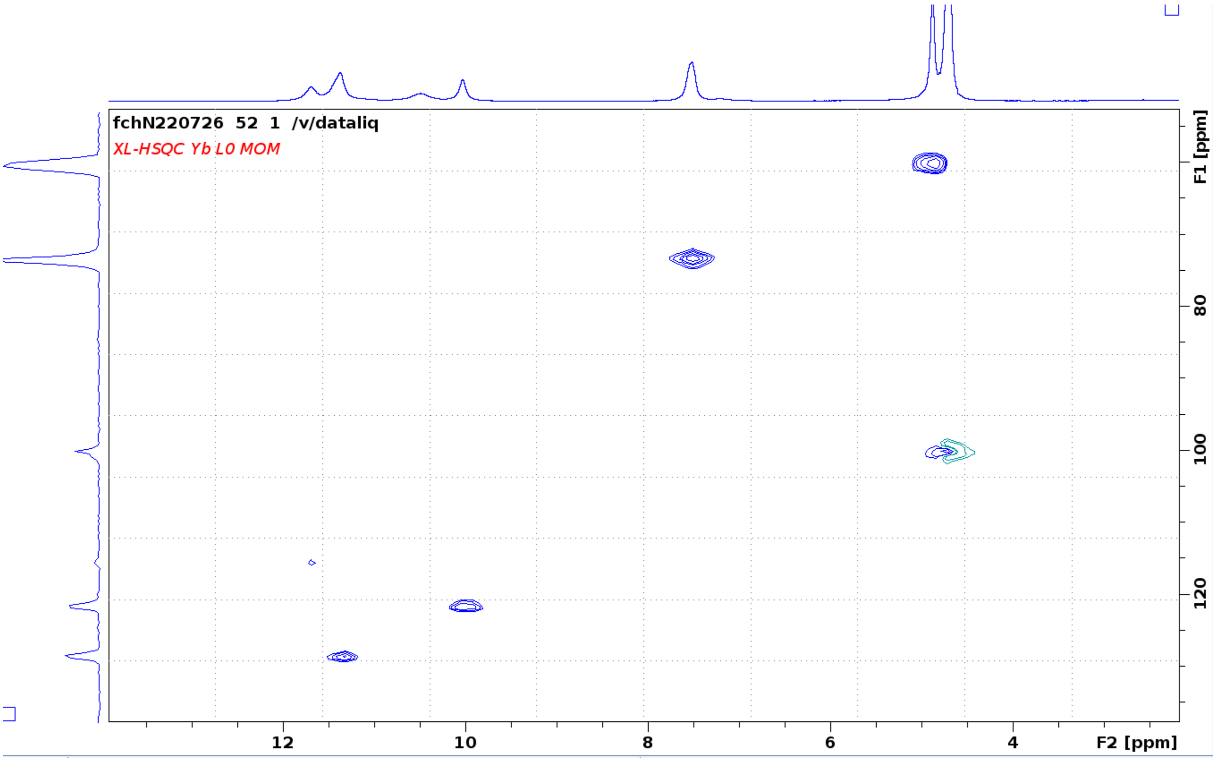

Figure S33. Zoom of HSQC NMR spectrum of **YbL0^{MOM}** measured in D_2O at 298 K.

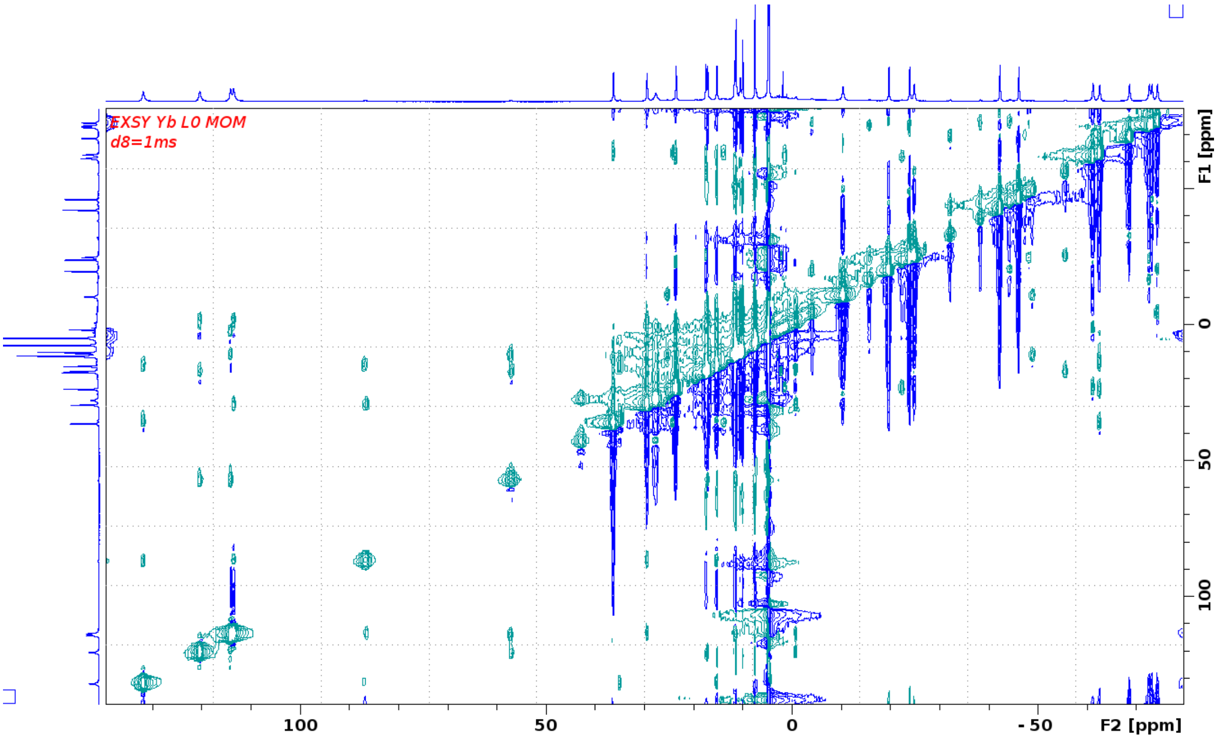

Figure S34. EXSY NMR spectrum of **YbL0^{MOM}** measured in D_2O at 298 K. Mixing time 1 ms.

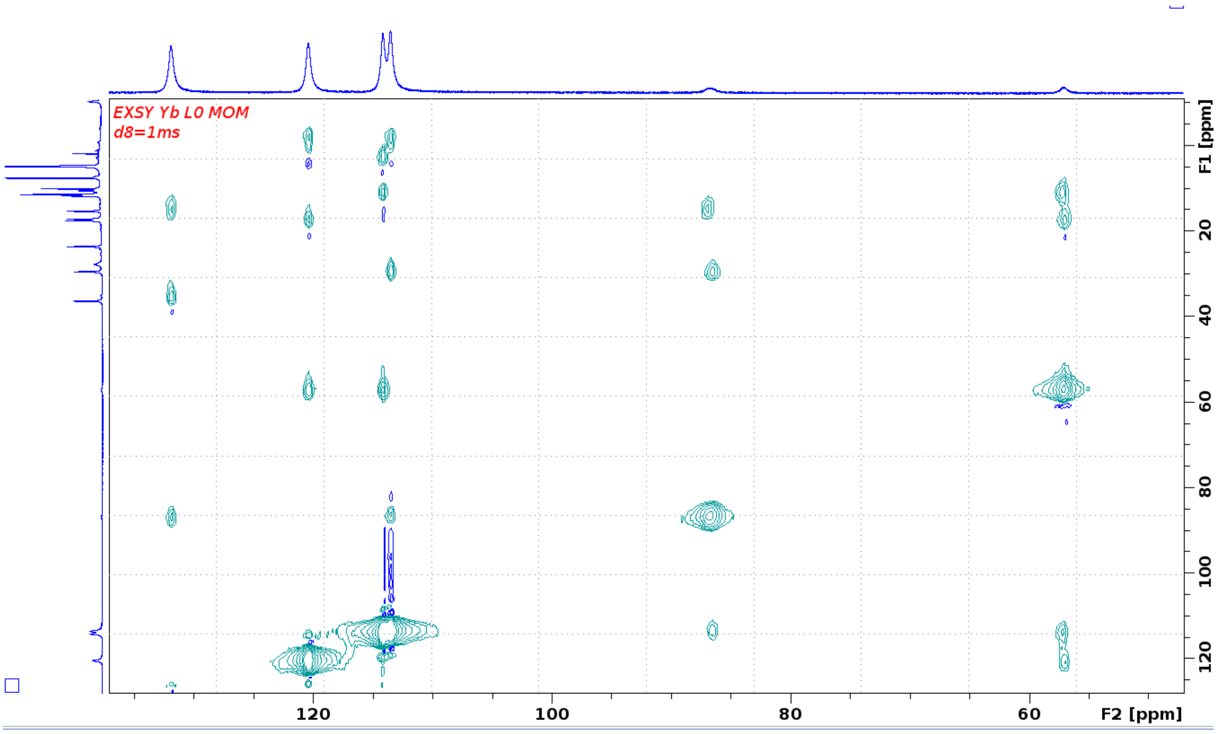

Figure S35. Zoom of EXSY NMR spectrum of **YbL0**$^{\mathbf{MOM}}$ measured in D_2O at 298 K. Mixing time 1 ms.

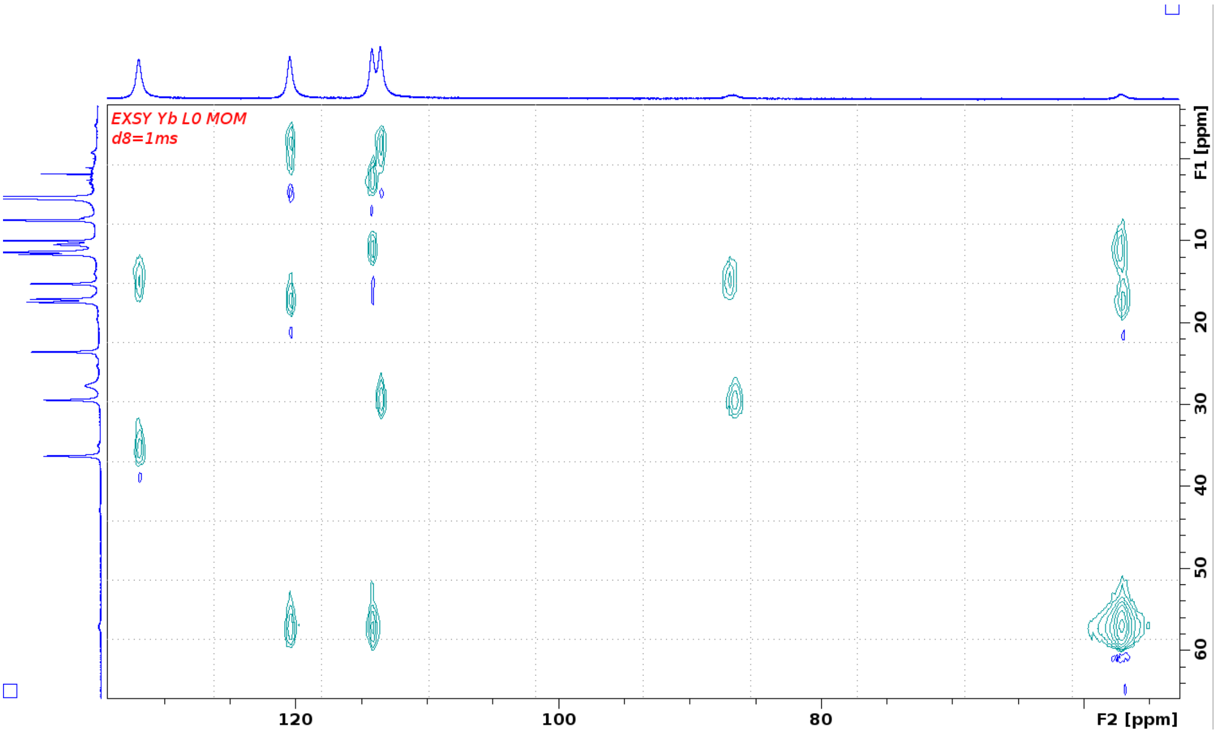

Figure S36. Zoom of EXSY NMR spectrum of **YbL0**$^{\mathbf{MOM}}$ measured in D_2O at 298 K. Mixing time 1 ms.

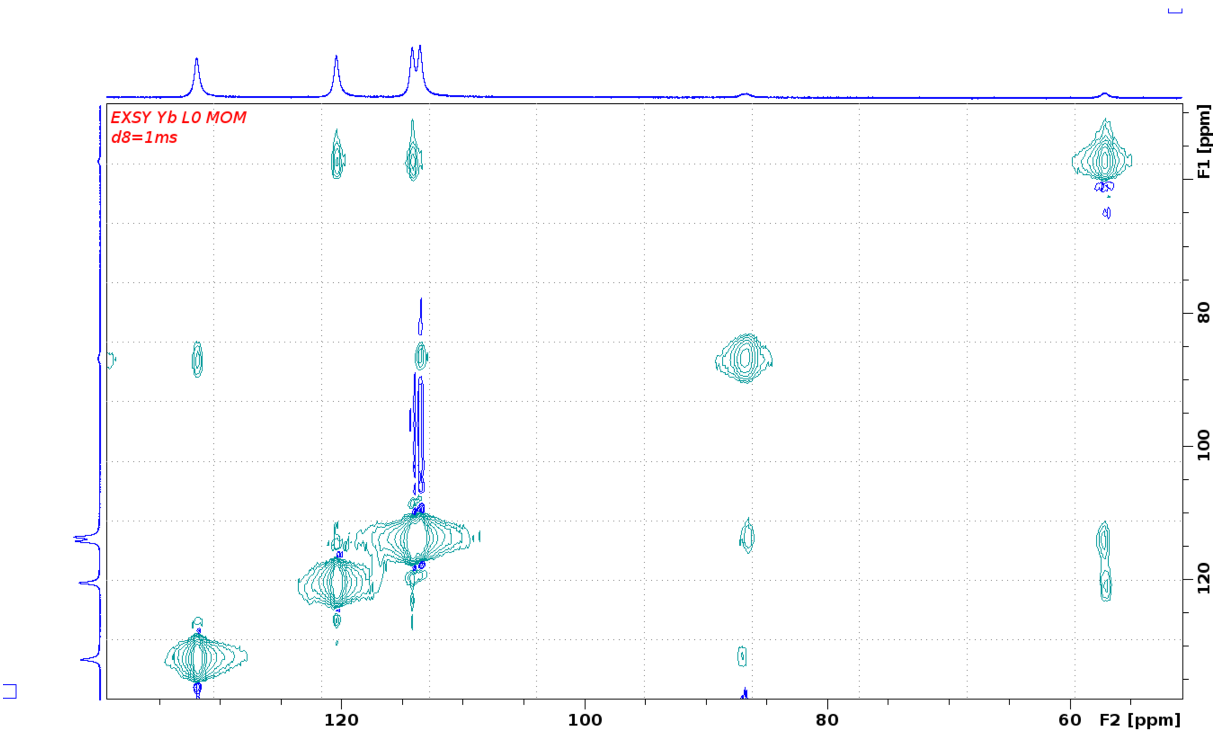

Figure S37. Zoom of EXSY NMR spectrum of **YbL0^{MOM}** measured in D_2O at 298 K. Mixing time 1 ms.

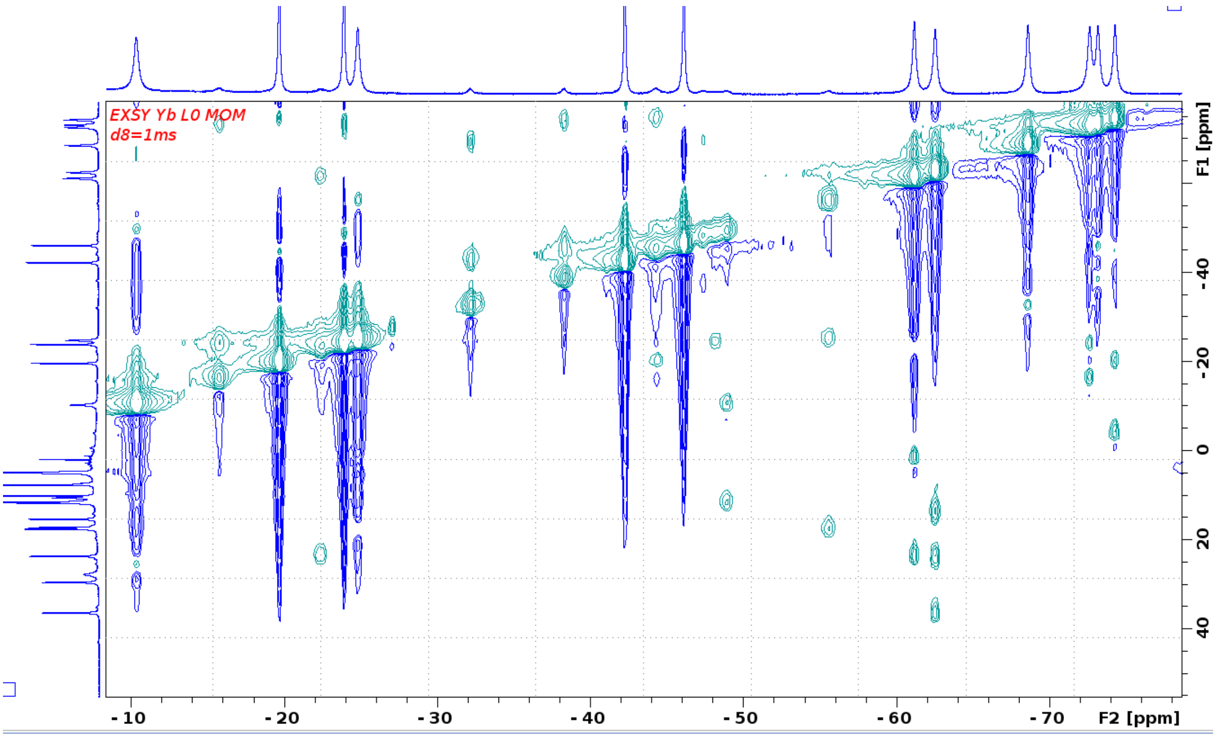

Figure S38. Zoom of EXSY NMR spectrum of **YbL0^{MOM}** measured in D_2O at 298 K. Mixing time 1 ms.

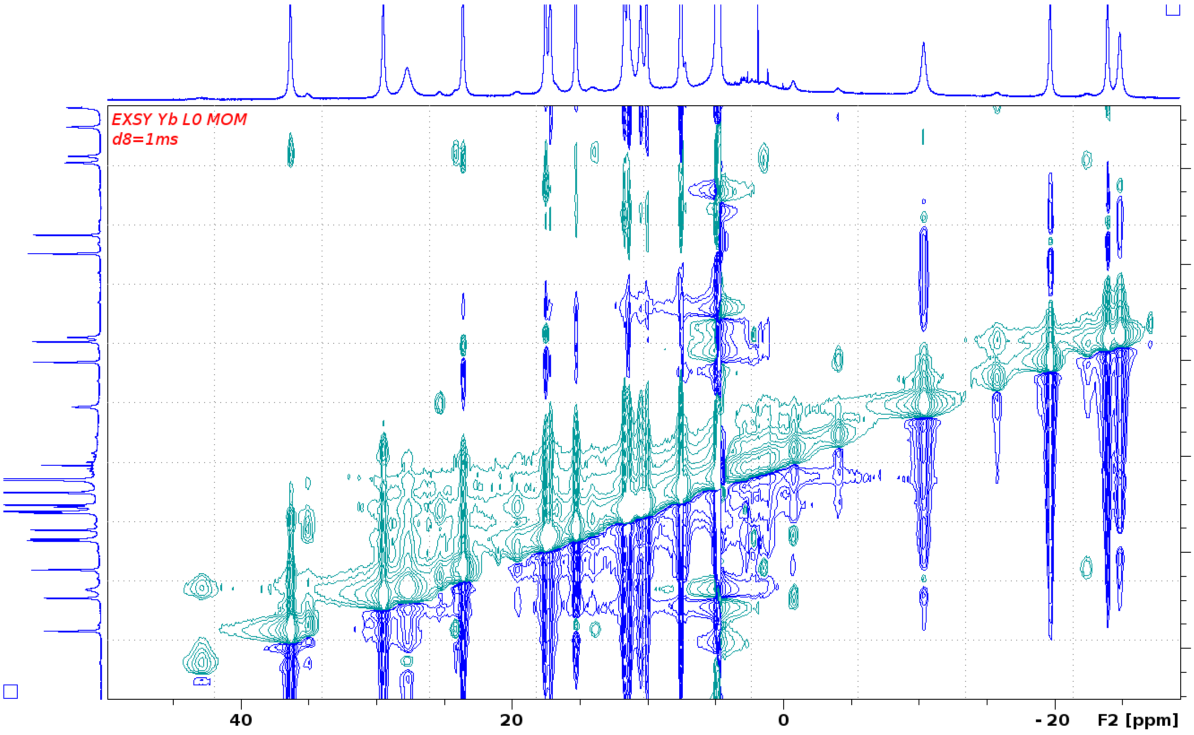

Figure S39. Zoom of EXSY NMR spectrum of **YbL0^{MOM}** measured in D_2O at 298 K. Mixing time 1 ms.

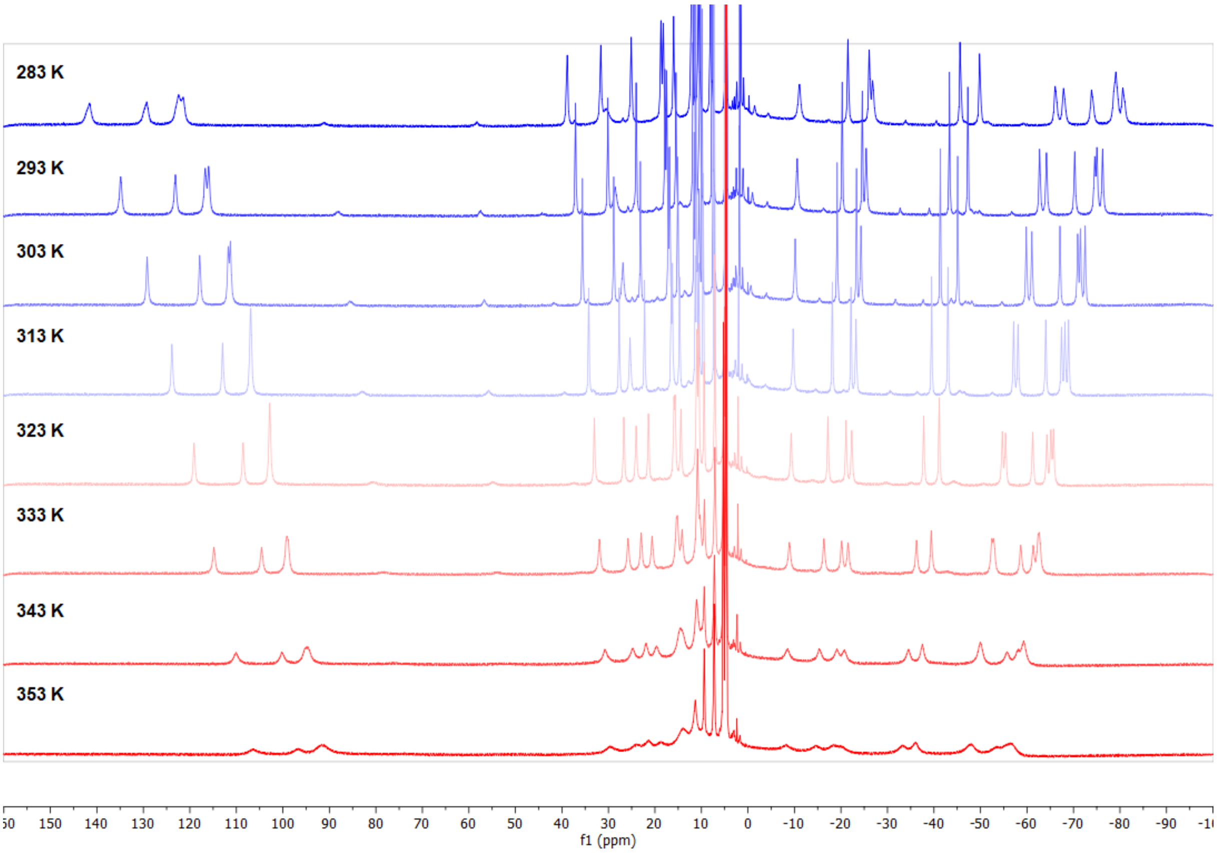

Figure S40. Variable temperature ^{1}H NMR spectra of **YbL0^{MOM}** measured in D_2O at 298 K.

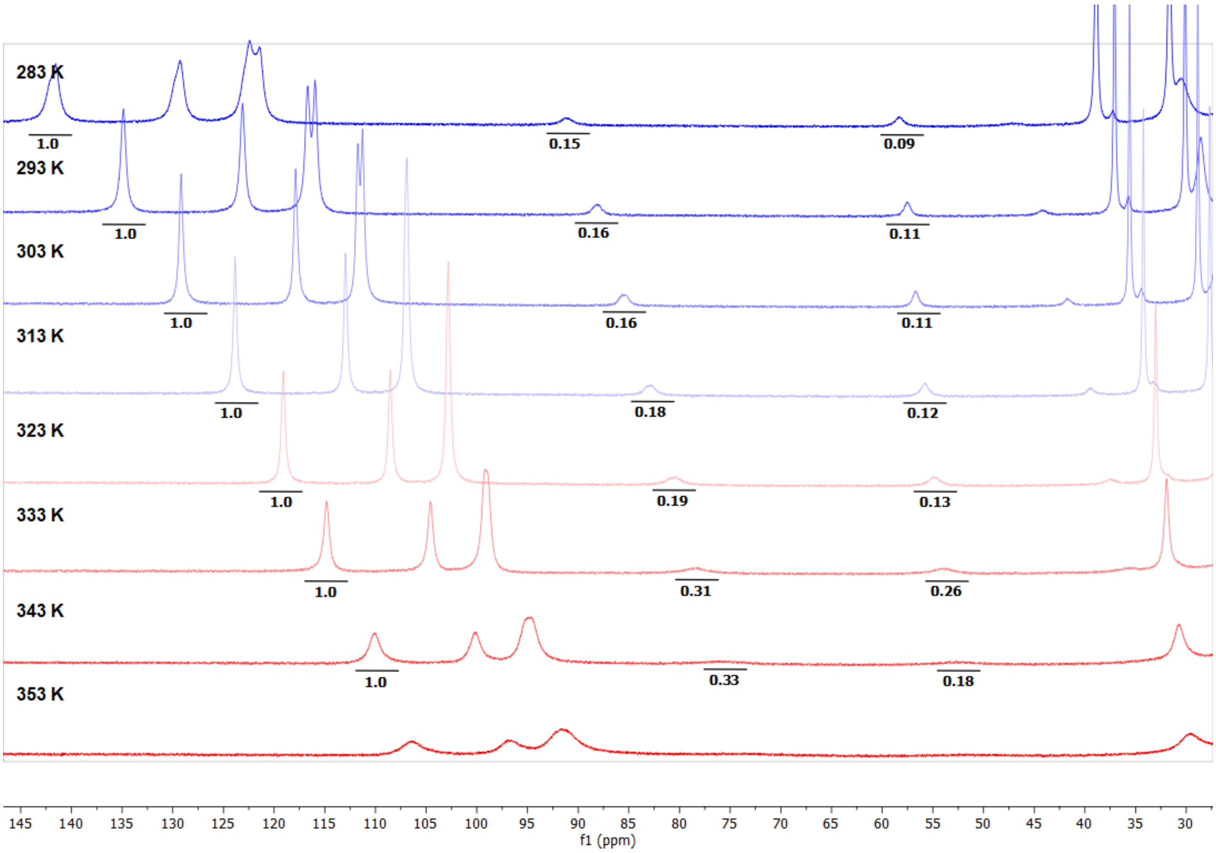

Figure S41. Zoom of variable temperature ^{1}H NMR spectra of **YbL0^{MOM}** measured in D_2O at 298 K with indications of relative integrations.

X-RAY CRYSTALLOGRAPHY

CCDC 2026787 contains the supplementary crystallographic data for this paper. The data can be obtained free of charge from The Cambridge Crystallographic Data Centre via www.ccdc.cam.ac.uk/structures.

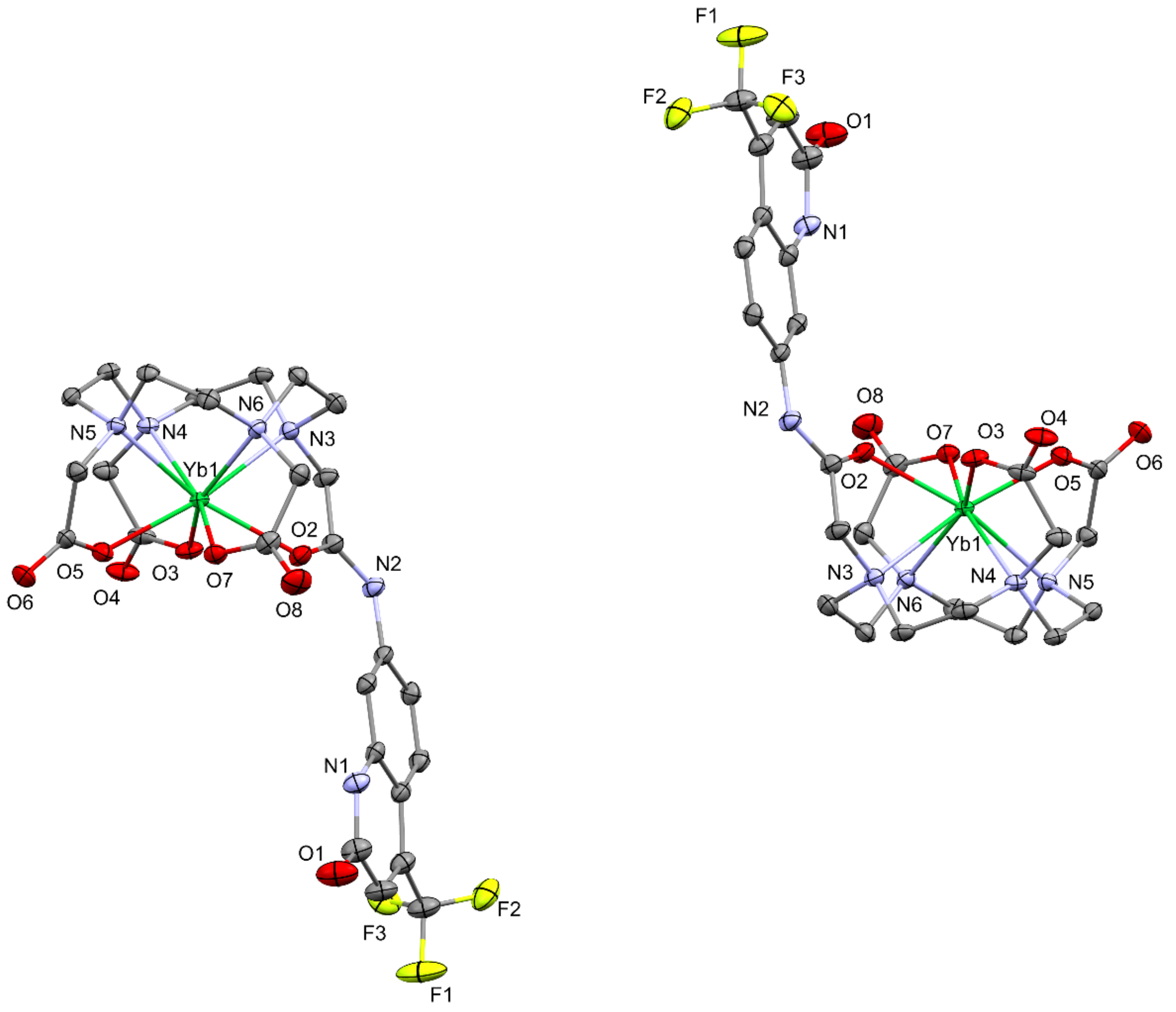

Figure S42. Packing in the crystal of **YbL0**$^{\mathbf{CF3}}$ with both Λ (left) and Δ (right) isomers in the cell unit. The H atoms, water and dioxane molecules were omitted for clarity. The crystal structure is depicted as ellipsoids displayed at 50% probability.

Crystallographic tables

Table S1. Crystal data and structure refinement.

Compound	**YbL0^{CF3}**
**Chemical formula**	$C_{26}H_{30}F_3N_6O_8Yb$ 0.5($C_4H_8O_2$) 3(H_2O)
***M*r**	882.70
**Crystal system, space group**	Triclinic $P\bar{1}$
**Temperature (K)**	170
***a*, *b*, *c* (Å)**	7.8382 (3) 8.8460 (3) 23.3083 (9)
**α, β, γ (°)**	91.6180 (10) 90.9720 (10) 95.9340 (10)
***V* (Å^3)**	1606.50 (10)
Z	2
**Radiation type**	Mo K_α
**μ (mm^{-1})**	3.00
**Crystal size (mm)**	0.17 × 0.12 × 0.11
**Diffractometer**	Bruker D8 APEX-II
**Absorption correction**	Multi-scan
**T_{min}, T_{max}**	0.5430, 0.7462
**No. of measured, independent and observed [I > 2s(I)] reflections**	26472 8375 8088
**R_{int}**	0.0221
**(sin θ/λ)$_{max}$ (Å^{-1})**	0.679
**$R[F^2 > 2\sigma(F^2)]$, $wR(F^2)$, S**	0.0199, 0.0454, 1.085
**No. of parameters**	485
**No. of restraints**	10
**H-atom treatment**	Independent and constrained
**Δ⟩$_{max}$, Δ⟩$_{min}$ (e Å^{-3})**	0.95, −1.17
**CCDC No.**	2026787

Selected Yb–O and Yb–N distances are displayed in Table S2. The table follows the convention outlined in Figure S29.

Table S2. Selected bond lengths (Å) and angles (°) for **YbL0^{CF3}**.

Parameter	**YbL0^{CF3}**
**Yb1–O1**	2.3354(14)
**Yb1–O2**	2.2448(14)
**Yb1–O3**	2.2344(14)
**Yb1–O4**	2.2800(14)
**Yb1–N1**	2.5456(17)
**Yb1–N2**	2.5229(16)
**Yb1–N3**	2.4929(16)
**Yb1–N4**	2.5143(16)
**4N$_{PL}$–Yb–4O$_{PL}$**	174.62(7)
**Yb–4N$_{PL}$**	1.4522(8)
**Yb–4O$_{PL}$**	1.0701(7)

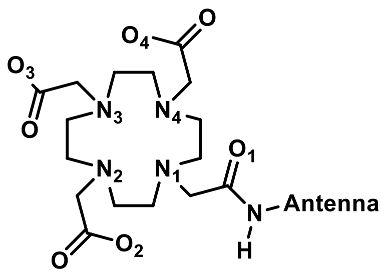

Figure S43. Numbering convention for Table S2 of **YbL0^{CF3}**.

ELECTROCHEMICAL CHARACTERIZATION

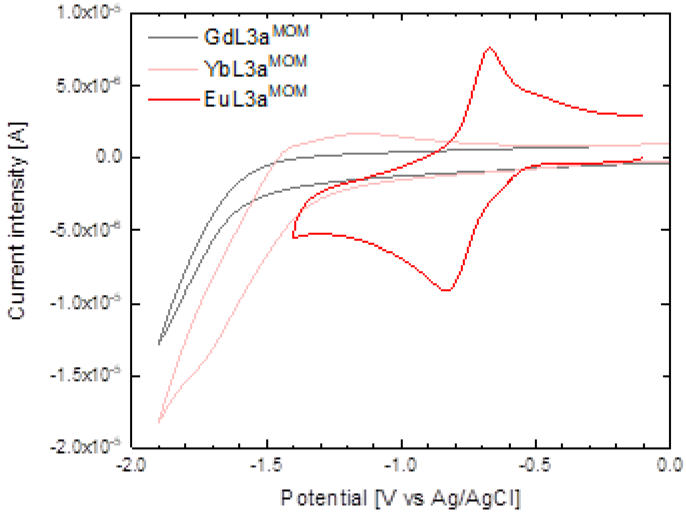

Figure S44. Comparison of **LnL3a^{MOM}** in water (0.1 M LiCl, pH 6.5). [**LnL3a^{MOM}**] = 1 mM; reference electrode, Ag/AgCl; working electrode, GC electrode; counter electrode, Pt wire; scan rate, 0.1 V/s. All measurements were conducted under Ar atmosphere.

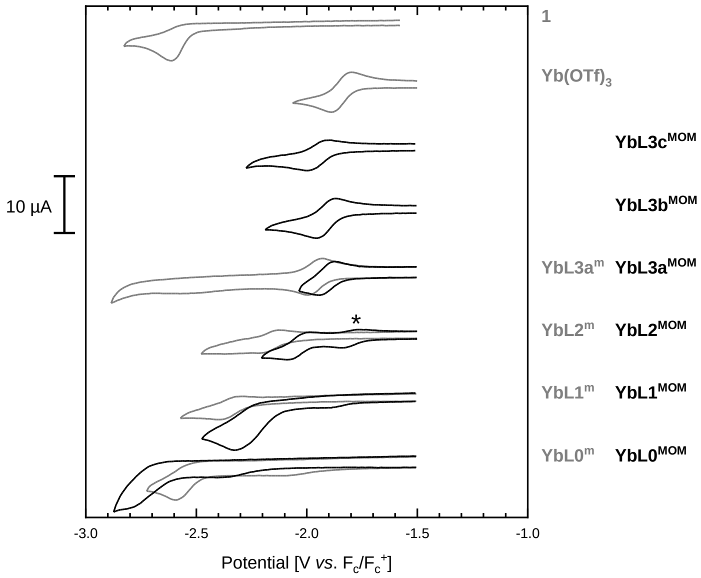

Figure S45. Comparison of model complexes **YbL^{m}** and **YbL^{MOM}** in DMF (0.1 M NBu_4ClO_4). [**YbL**] = 0.5 mM; reference electrode, Ag/$AgNO_3$ (10 mM in ACN); working electrode, GC electrode; counter electrode, Pt wire; scan rate, 0.1 V/s. All measurements were conducted in a glovebox.

Table S3. Peak potentials (E_{pa}, E_{pc}), half potential ($E_{1/2}$), and peak potential separation (ΔE_p) of **YbL**, **1** and $Yb(OTf)_3$ in DMF. Electrolyte, 0.1 M NBu_4ClO_4; scan rate, 0.1 V/s.

	E_{pa} (V *vs.* F_c/F_c^+)	E_{pc} (V *vs.* F_c/F_c^+)	E_{pc} (V *vs.* NHE)	$E_{1/2}$ (V *vs.* F_c/F_c^+)	$E_{1/2}$ (V *vs.* NHE)	ΔE_p (V *vs.* F_c/F_c^+)
**1**	--	–2.622	–2.222	--	--	--
$Yb(OTf)_3$	–1.795	–1.892	–1.492	–1.843	–1.443	0.096
**YbL3a^{m}**	–1.933	–2.001	–1.601	–1.967	–1.567	0.068
**YbL2^{m}**	–2.122	–2.230	–1.830	–2.176	–1.776	0.108
**YbL1^{m}**	–2.306	–2.400	–2.000	–2.353	–1.953	0.095
**YbL0^{m}**	–2.430	–2.595	–2.195	–2.512	–2.112	0.165
**YbL0^{MOM}**	--	–2.823	–2.423	--	--	--
**YbL1^{MOM}**	--	–2.324	–1.924	--	--	--
**YbL2^{MOM}**	–1.984	–2.095	–1.695	–2.040	–1.640	0.111
**YbL3a^{MOM}**	–1.870	–1.946	–1.546	–1.908	–1.508	0.076
**YbL3b^{MOM}**	–1.872	–1.957	–1.557	–1.914	–1.514	0.085
**YbL3c^{MOM}**	–1.907	–1.998	–1.598	–1.953	–1.553	0.091

Scan rate studies

Yb(OTf)$_3$

[**Yb(OTf)$_3$**] = 0.5 mM in DMF; electrolyte, 0.1 M NBu_4ClO_4; WE: GC, Ref: Ag/$AgNO_3$ (10 mM in ACN), CE: Pt wire, equilibration time: 10s, step 10 mV (0.025 – 0.1 V/s) or 20 mV (0.2 to 0.5 V/s).

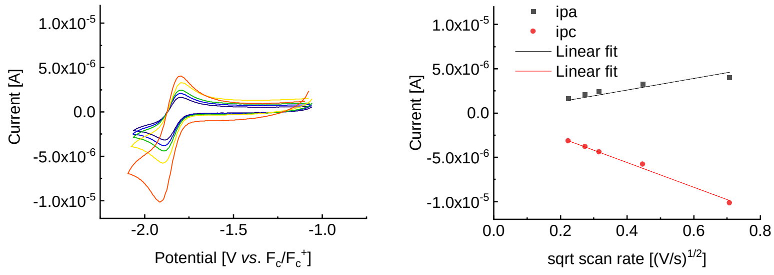

Figure S46. *Left*: cyclic voltammograms at different scan rates; *Right*: Plot of peak current intensities *vs*. square root of scan rate.

Table S4. Peak potentials (E_{pa}, E_{pc}), peak current intensities (i_{pa}, i_{pc}), half-reduction potential ($E_{1/2}$), and difference between peak potentials (ΔE_p) at different scan rates.

scan rate<br>V/s	E_{pa}<br>V	E_{pc}<br>V	i_{pa}<br>µA	i_{pc}<br>µA	$E_{1/2}$<br>V	ΔE_p<br>V
0.05	–1.796	–1.887	1.647	–3.150	–1.841	0.090
0.075	–1.796	–1.891	2.081	–3.790	–1.843	0.094
0.1	–1.795	–1.892	2.447	–4.395	–1.843	0.096
0.2	–1.794	–1.897	3.295	–5.770	–1.845	0.103
0.5	–1.797	–1.916	4.047	–10.16	–1.857	0.119

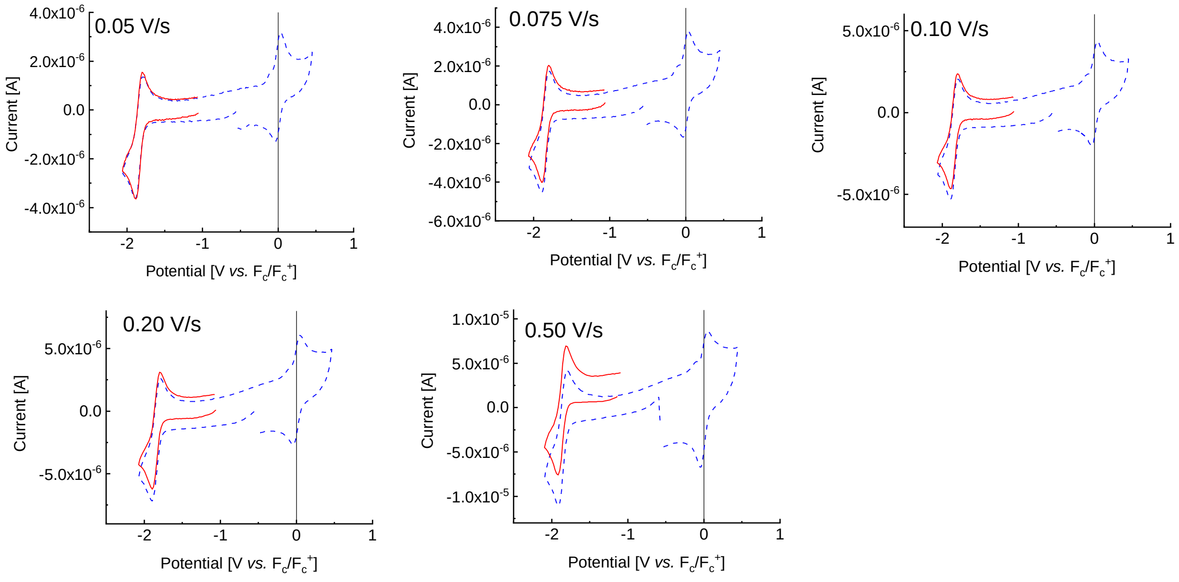

Figure S47. Cyclic voltammograms at different scan rates of Yb(OTf)$_3$ (straight line) and comparison with CVs with ferrocene (dotted line).

1

[**1**] = 0.5 mM in DMF; electrolyte, 0.1 M NBu_4ClO_4; WE: GC, Ref: Ag/$AgNO_3$ (10 mM in ACN), CE: Pt wire, equilibration time: 10s, step 10 mV (0.025 – 0.1 V/s) or 20 mV (0.2 to 0.5 V/s).

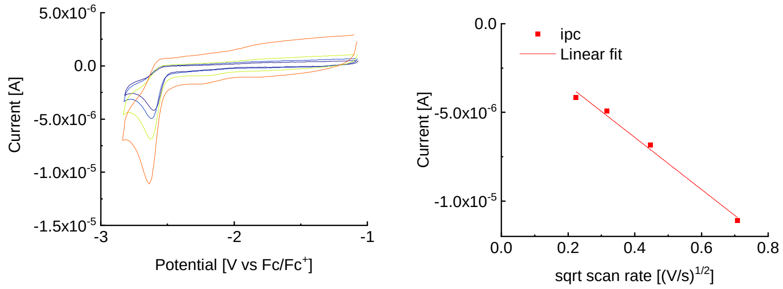

Figure S48. *Left*: cyclic voltammograms at different scan rates; *Right*: Plot of peak current intensities *vs*. square root of scan rate.

Table S5. Peak potential (E_{pc}), and peak current intensity (i_{pc}) at different scan rates.

scan rate V/s	E_{pc} V	i_{pc} μA
0.05	–2.603	–4.163
0.1	–2.622	–4.931
0.2	–2.626	–6.850
0.5	–2.640	–11.08

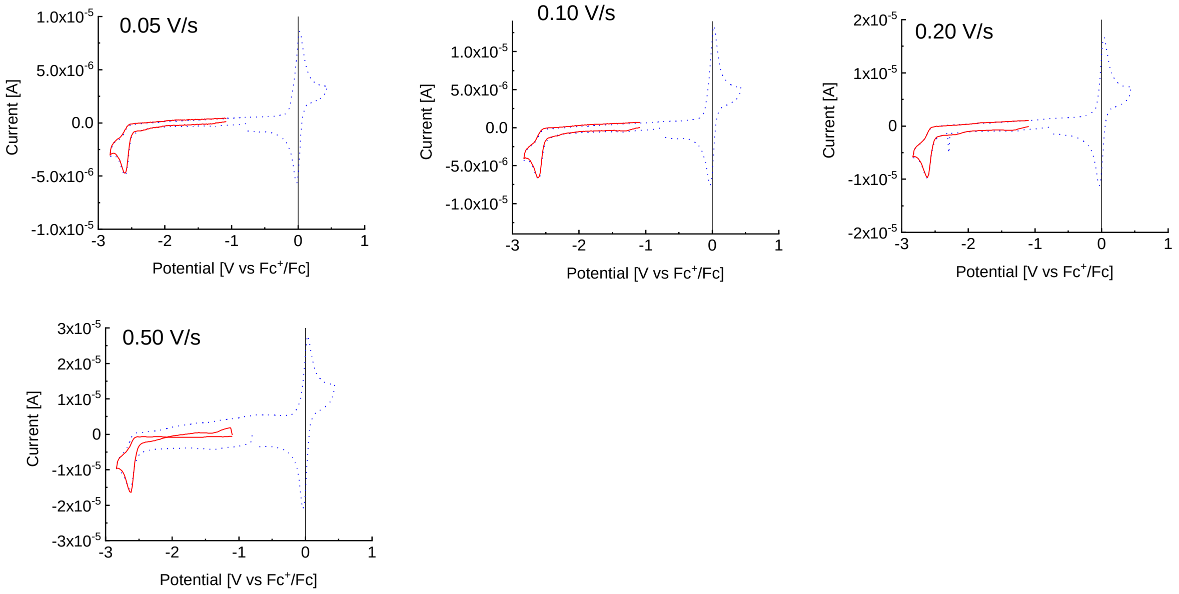

Figure S49. Cyclic voltammograms at different scan rates of **1** (straight line) and comparison with CVs with ferrocene (dotted line).

YbL0^m

[**YbL^{0m}**] = 0.5 mM in DMF; electrolyte, 0.1 M NBu_4ClO_4; WE: GC, Ref: Ag/$AgNO_3$ (10 mM in ACN), CE: Pt wire, equilibration time: 10s, step 10 mV (0.025 – 0.1 V/s) or 20 mV (0.2 to 0.5 V/s).

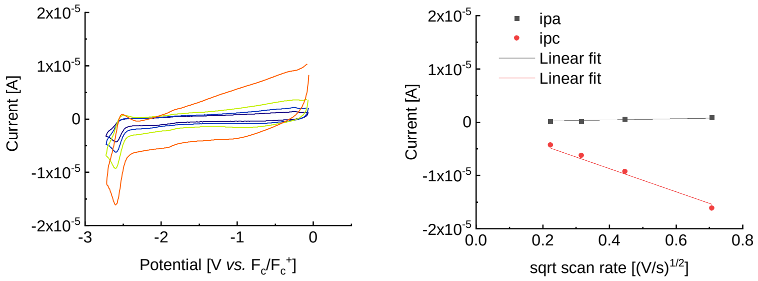

Figure S50. *Left*: cyclic voltammograms at different scan rates; *Right*: Plot of peak current intensities *vs*. square root of scan rate.

Table S6. Peak potentials (E_{pa}, E_{pc}), peak current intensities (i_{pa}, i_{pc}), half-reduction potential ($E_{1/2}$), and difference between peak potentials (ΔE_p) at different scan rates.

scan rate<br>V/s	E_{pa}<br>V	E_{pc}<br>V	i_{pa}<br>µA	i_{pc}<br>µA	$E_{1/2}$<br>V	ΔE_p<br>V
0.05	–2.404	–2.596	0.077	–4.300	–2.500	0.192
0.1	–2.430	–2.595	0.132	–6.242	–2.512	0.165
0.2	–2.478	–2.607	0.626	–9.270	–2.543	0.128
0.5	–2.508	–2.600	0.867	–16.14	–2.554	0.091

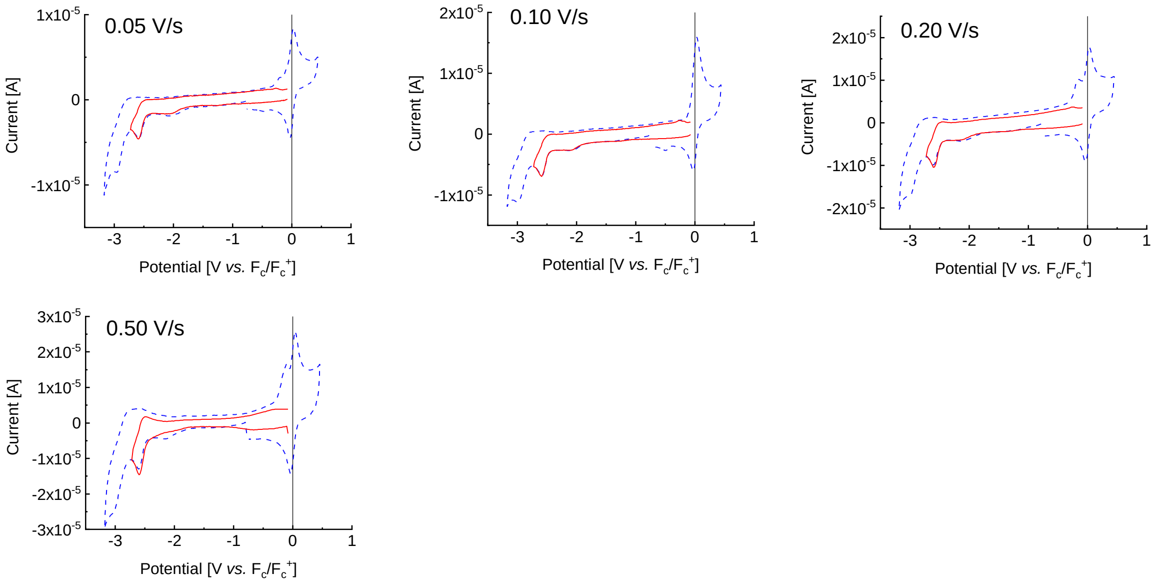

Figure S51. Cyclic voltammograms at different scan rates of **YbL^{0m}** (straight line) and comparison with CVs with ferrocene (dotted line).

YbL1^{m}

[**YbL^{1m}**] = 0.5 mM in DMF; electrolyte, 0.1 M NBu_4ClO_4; WE: GC, Ref: Ag/$AgNO_3$ (10 mM in ACN), CE: Pt wire, equilibration time: 10s, step 10 mV (0.025 – 0.1 V/s) or 20 mV (0.2 to 0.5 V/s).

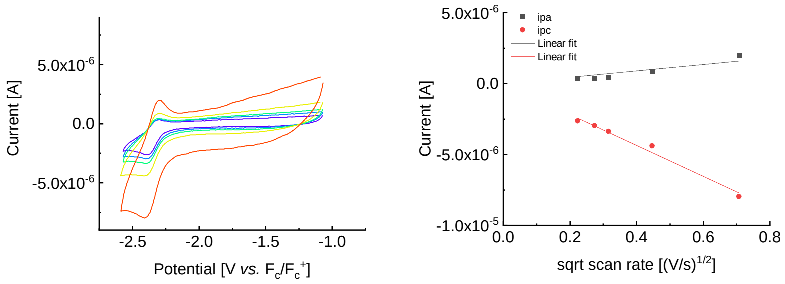

Figure S52. *Left*: cyclic voltammograms at different scan rates; *Right*: Plot of peak current intensities *vs*. square root of scan rate.

Table S7. Peak potentials (E_{pa}, E_{pc}), peak current intensities (i_{pa}, i_{pc}), half-reduction potential ($E_{1/2}$), and difference between peak potentials (ΔE_p) at different scan rates.

scan rate V/s	E_{pa} V	E_{pc} V	i_{pa} µA	i_{pc} µA	$E_{1/2}$ V	ΔE_p V
0.05	–2.297	–2.391	0.327	–2.634	–2.344	0.095
0.075	–2.302	–2.399	0.349	–2.974	–2.351	0.098
0.1	–2.306	–2.400	0.432	–3.377	–2.353	0.095
0.2	–2.295	–2.414	0.872	–4.408	–2.355	0.119
0.5	–2.297	–2.408	1.967	–7.974	–2.353	0.111

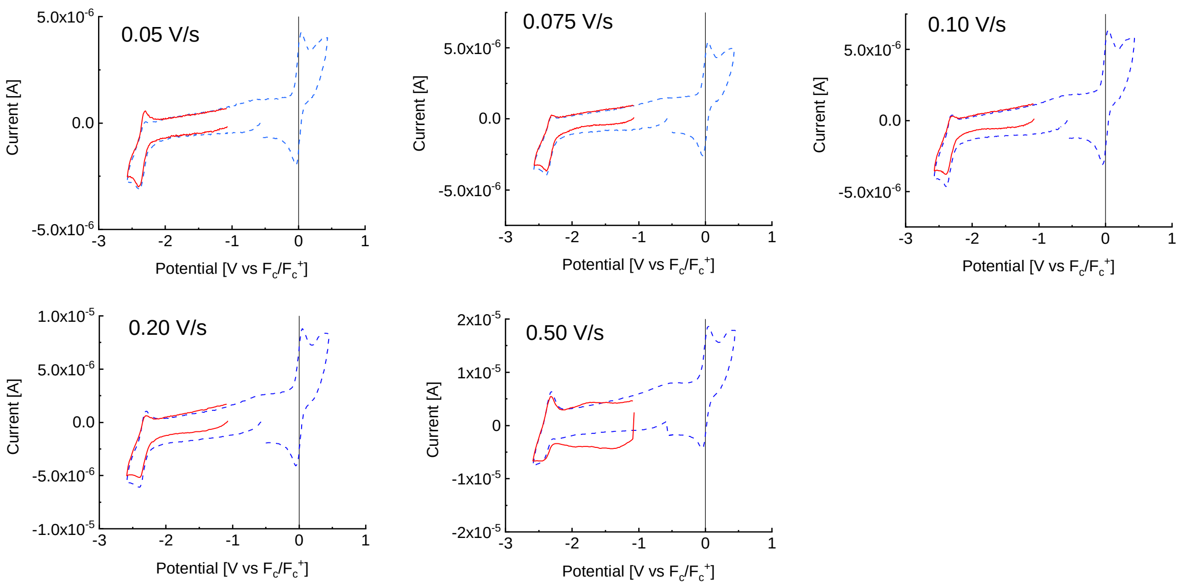

Figure S53. Cyclic voltammograms at different scan rates of **YbL^{1m}** (straight line) and comparison with CVs with ferrocene (dotted line).

$YbL2^{m}$

[**YbL^{2m}**] = 0.5 mM in DMF; electrolyte, 0.1 M NBu_4ClO_4; WE: GC, Ref: Ag/$AgNO_3$ (10 mM in ACN), CE: Pt wire, equilibration time: 10s, step 10 mV (0.025 – 0.1 V/s) or 20 mV (0.2 to 0.5 V/s).

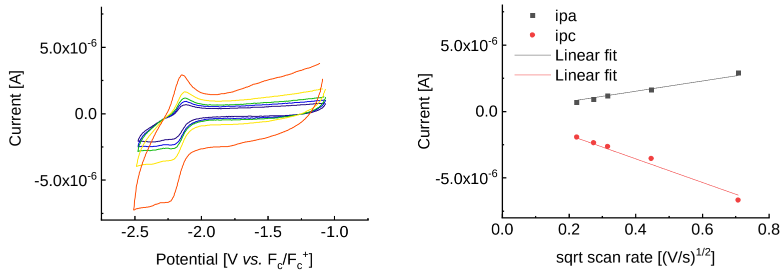

Figure S54. *Left*: cyclic voltammograms at different scan rates; *Right*: Plot of peak current intensities *vs*. square root of scan rate.

Table S8. Peak potentials (E_{pa}, E_{pc}), peak current intensities (i_{pa}, i_{pc}), half-reduction potential ($E_{1/2}$), and difference between peak potentials (ΔE_p) at different scan rates.

scan rate V/s	E_{pa} V	E_{pc} V	i_{pa} µA	i_{pc} µA	$E_{1/2}$ V	ΔE_p V
0.05	–2.115	–2.240	0.665	–1.94	–2.177	0.125
0.075	–2.118	–2.234	0.923	–2.36	–2.176	0.116
0.1	–2.122	–2.230	1.17	–2.63	–2.176	0.108
0.2	–2.123	–2.246	1.64	–3.53	–2.184	0.123
0.5	–2.143	–2.270	2.90	–6.68	–2.206	0.127

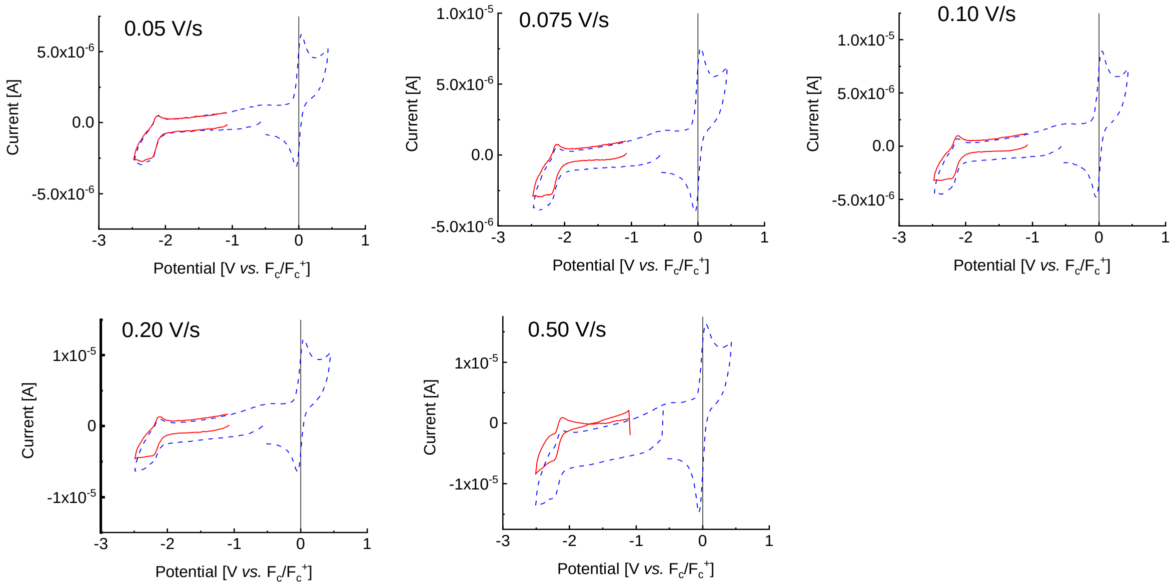

Figure S55. Cyclic voltammograms at different scan rates of **YbL^{2m}** (straight line) and comparison with CVs with ferrocene (dotted line).

YbL3^m

[**YbL^{3m}**] = 0.5 mM in DMF; electrolyte, 0.1 M NBu_4ClO_4; WE: GC, Ref: Ag/$AgNO_3$ (10 mM in ACN), CE: Pt wire, equilibration time: 10s, step 10 mV (0.025 – 0.1 V/s) or 20 mV (0.2 to 0.5 V/s).

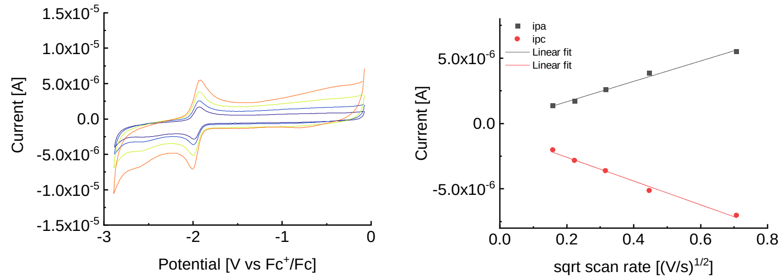

Figure S56. *Left*: cyclic voltammograms at different scan rates; *Right*: Plot of peak current intensities *vs*. square root of scan rate.

Table S9. Peak potentials (E_{pa}, E_{pc}), peak current intensities (i_{pa}, i_{pc}), half-reduction potential ($E_{1/2}$), and difference between peak potentials (ΔE_p) at different scan rates.

scan rate V/s	E_{pa} V	E_{pc} V	i_{pa} µA	i_{pc} µA	$E_{1/2}$ V	ΔE_p V
0.05	–1.933	–1.996	1.716	–2.836	–1.964	0.063
0.1	–1.933	–2.001	2.586	–3.611	–1.967	0.068
0.2	–1.934	–1.998	3.864	–5.129	–1.966	0.064
0.5	–1.923	–2.004	5.508	–7.024	–1.963	0.081

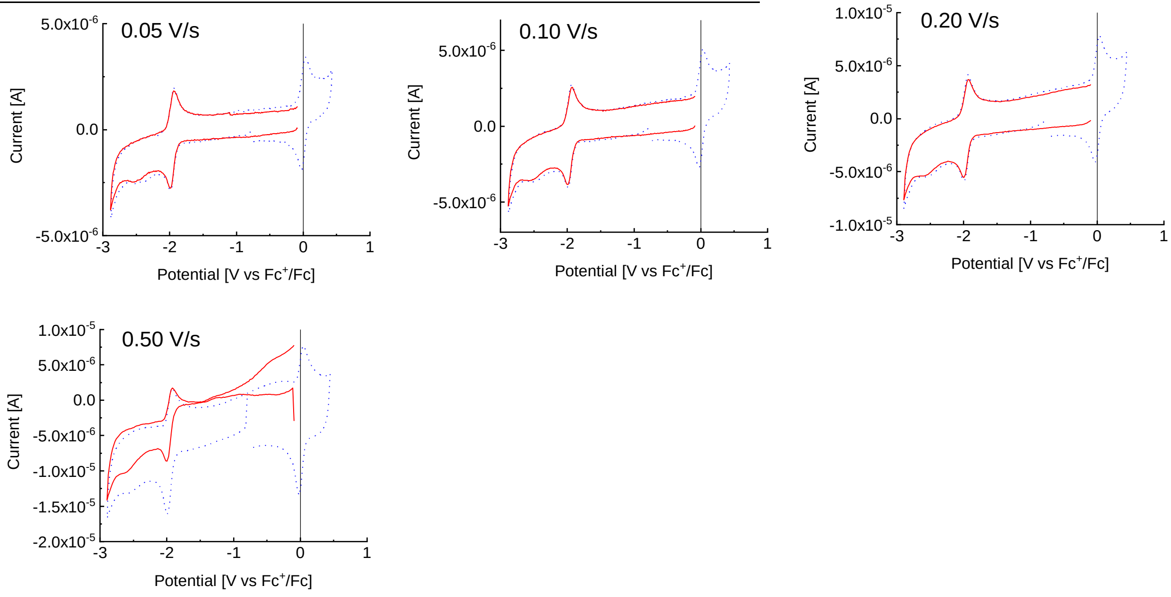

Figure S57. Cyclic voltammograms at different scan rates of **YbL^{3m}** (straight line) and comparison with CVs with ferrocene (dotted line).

YbL3a^{MOM}

[**YbL3a^{MOM}**] = 0.5 mM in DMF; electrolyte, 0.1 M NBu_4ClO_4; WE: GC, Ref: Ag/$AgNO_3$ (10 mM in ACN), CE: Pt wire, equilibration time: 10s, step 10 mV (0.025 – 0.1 V/s) or 20 mV (0.2 to 0.5 V/s).

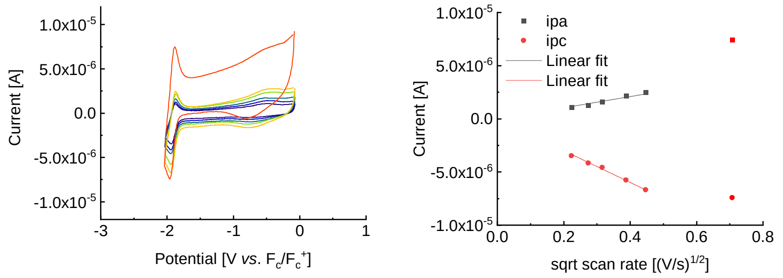

Figure S58. *Left*: cyclic voltammograms at different scan rates; *Right*: Plot of peak current intensities *vs*. square root of scan rate.

Table S10. Peak potentials (E_{pa}, E_{pc}), peak current intensities (i_{pa}, i_{pc}), half-reduction potential ($E_{1/2}$), and difference between peak potentials (ΔE_p) at different scan rates.

scan rate V/s	E_{pa} V	E_{pc} V	i_{pa} µA	i_{pc} µA	$E_{1/2}$ V	ΔE_p V
0.05	–1.877	–1.937	1.069	–3.474	–1.907	0.060
0.075	–1.874	–1.946	1.255	–4.152	–1.910	0.072
0.1	–1.870	–1.946	1.610	–4.575	–1.908	0.076
0.15	–1.869	–1.948	2.157	–5.771	–1.908	0.080
0.2	–1.872	–1.951	2.470	–6.693	–1.911	0.080
0.5	–1.884	–1.959	7.448	–7.434	–1.921	0.076

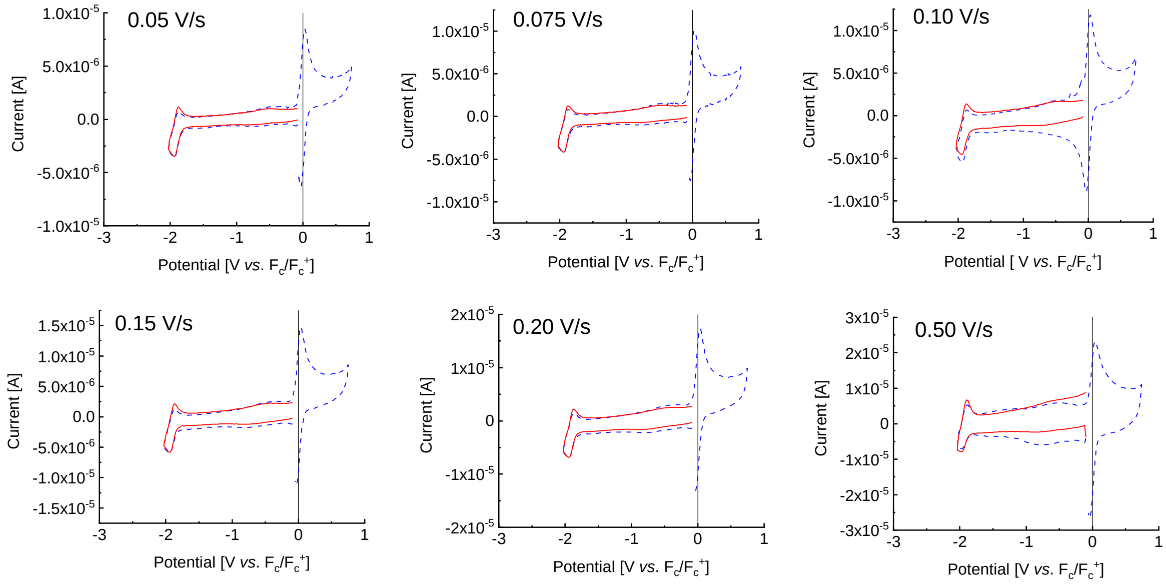

Figure S59. Cyclic voltammograms at different scan rates of **YbL3a^{MOM}** (straight line) and comparison with CVs with ferrocene (dotted line).

YbL3b^{MOM}

[**YbL3b^{MOM}**] = 0.5 mM in DMF; electrolyte, 0.1 M NBu_4ClO_4; WE: GC, Ref: $Ag/AgNO_3$ (10 mM in ACN), CE: Pt wire, equilibration time: 10s, step 10 mV (0.025 – 0.1 V/s) or 20 mV (0.2 to 0.5 V/s).

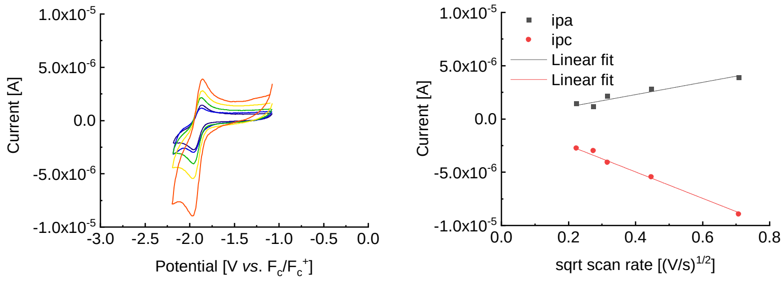

Figure S60. *Left*: cyclic voltammograms at different scan rates; *Right*: Plot of peak current intensities *vs*. square root of scan rate.

Table S11. Peak potentials (E_{pa}, E_{pc}), peak current intensities (i_{pa}, i_{pc}), half-reduction potential ($E_{1/2}$), and difference between peak potentials (ΔE_p) at different scan rates.

scan rate<br>V/s	E_{pa}<br>V	E_{pc}<br>V	i_{pa}<br>µA	i_{pc}<br>µA	$E_{1/2}$<br>V	ΔE_p<br>V
0.05	–1.865	–1.959	1.448	–2.736	–1.912	0.094
0.075	–1.868	–1.960	1.161	–2.984	–1.914	0.091
0.1	–1.872	–1.957	2.142	–4.062	–1.914	0.085
0.2	–1.857	–1.964	2.797	–5.446	–1.911	0.107
0.5	–1.854	–1.969	3.901	–8.946	–1.912	0.115

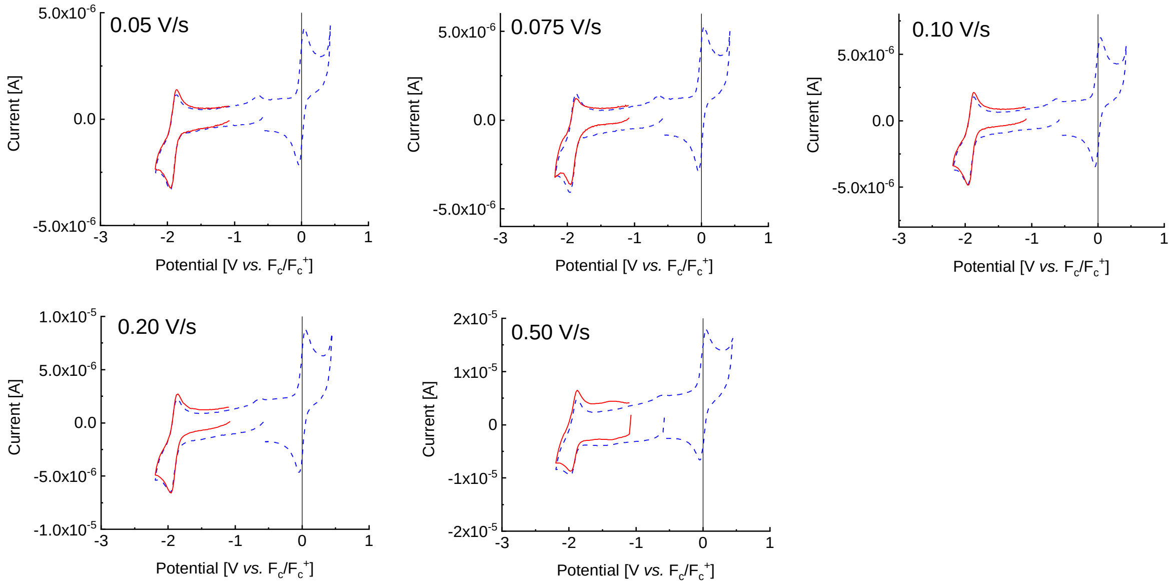

Figure S61. Cyclic voltammograms at different scan rates of **YbL3b^{MOM}** (straight line) and comparison with CVs with ferrocene (dotted line).

YbL3c^{MOM}

[**YbL3c^{MOM}**] = 0.5 mM in DMF; electrolyte, 0.1 M NBu_4ClO_4; WE: GC, Ref: Ag/$AgNO_3$ (10 mM in ACN), CE: Pt wire, equilibration time: 10s, step 10 mV (0.025 – 0.1 V/s) or 20 mV (0.2 to 0.5 V/s).

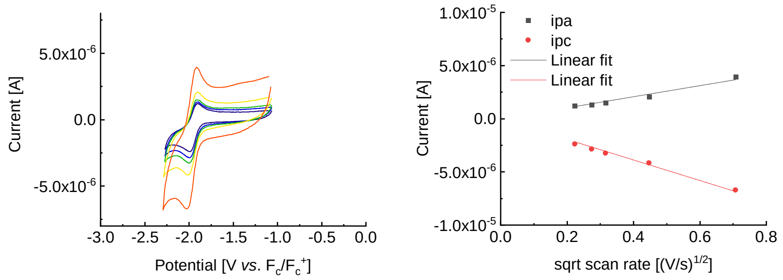

Figure S62. *Left*: cyclic voltammograms at different scan rates; *Right*: Plot of peak current intensities *vs*. square root of scan rate.

Table S12. Peak potentials (E_{pa}, E_{pc}), peak current intensities (i_{pa}, i_{pc}), half-reduction potential ($E_{1/2}$), and difference between peak potentials (ΔE_p) at different scan rates.

scan rate V/s	E_{pa} V	E_{pc} V	i_{pa} µA	i_{pc} µA	$E_{1/2}$ V	ΔE_p V
0.05	–1.902	–1.993	1.208	–2.398	–1.948	0.091
0.075	–1.903	–1.994	1.327	–2.859	–1.949	0.091
0.1	–1.907	–1.998	1.490	–3.254	–1.953	0.091
0.2	–1.907	–2.014	2.070	–4.155	–1.961	0.107
0.5	–1.914	–2.029	3.950	–6.701	–1.972	0.115

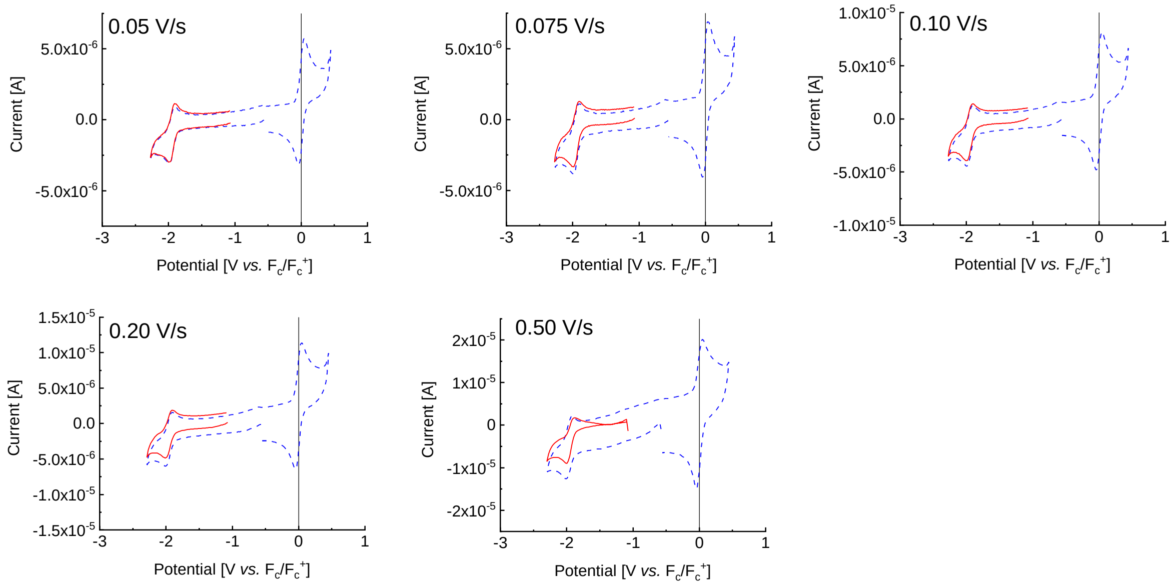

Figure S63. Cyclic voltammograms at different scan rates of **YbL3c^{MOM}** (straight line) and comparison with CVs with ferrocene (dotted line).

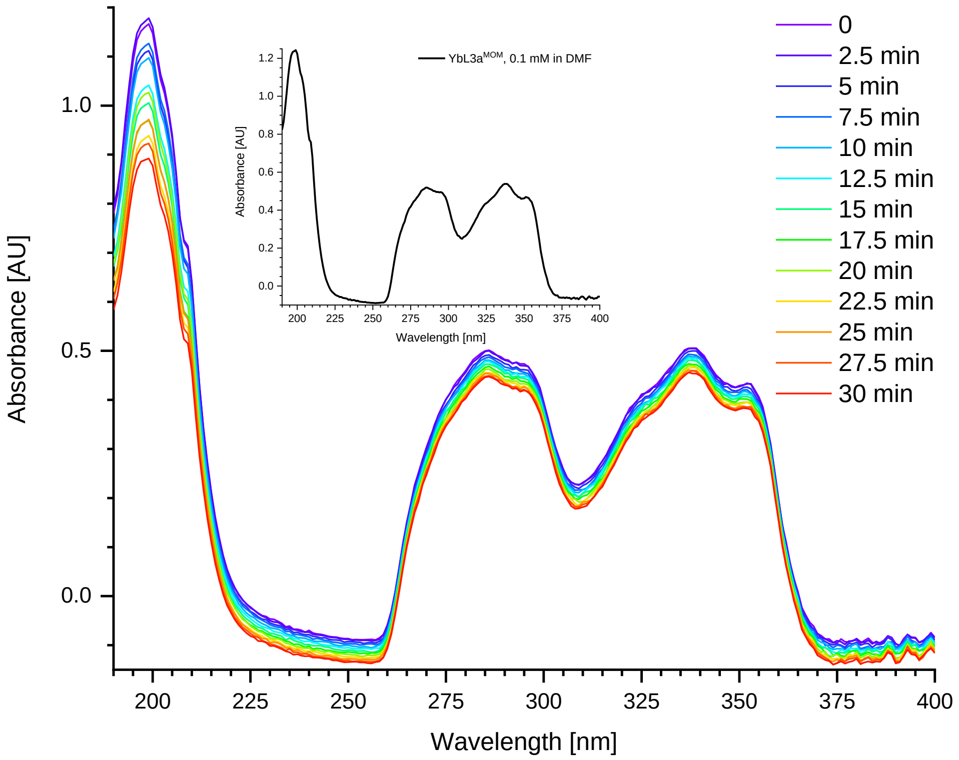

Figure S64. UV-Vis spectroelectrochemistry in DMF (0.1 M NBu_4ClO_4) showing the intact antenna upon reduction of Yb(III) to Yb(II) in **YbL3a^{MOM}** (0.1 mM) at a potential of –2.15 V vs Fc^+/Fc^0. The small insert shows the UV-Vis spectrum of **YbL3a^{MOM}** before applying the potential.

PHOTOPHYSICAL CHARACTERIZATION

Absorption, emission and excitation spectra

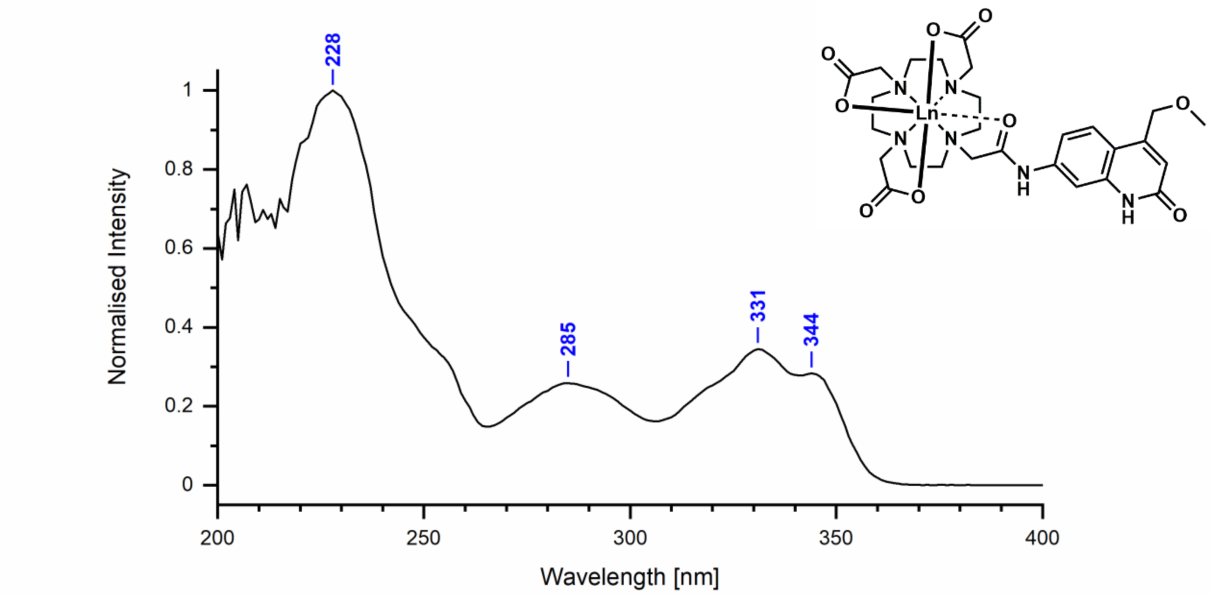

Figure S65. Normalized UV absorption spectrum of **YbL0^{MOM}** (10 µM) in 10 mM PIPES-buffered aqueous solution, pH 6.5. Blue numbers indicate the local maxima of the spectra.

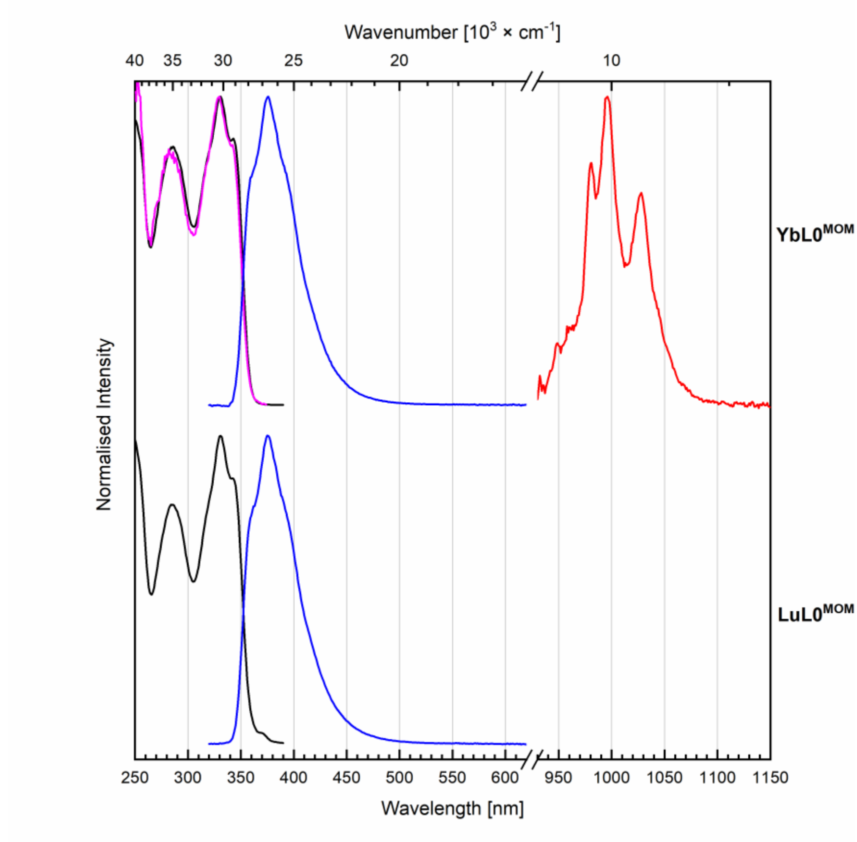

Figure S66. The excitation spectra of ligand fluorescence and Yb(III) emission (black and magenta lines, left, **YbL0^{MOM}** λ_{em} = 405 and 997 nm, respectively; **LuL0^{MOM}**, λ_{em} = 425 nm) and steady-state emission spectra of **LnL0^{MOM}** at r.t. (blue and red lines, right, Ln = Yb, λ_{ex} = 329 and 323 nm, respectively; Ln = Lu, λ_{ex} = 308 nm). [**LnL0^{MOM}**] = 10 µM, 10 mM PIPES-buffered aqueous solution, pH 6.5.

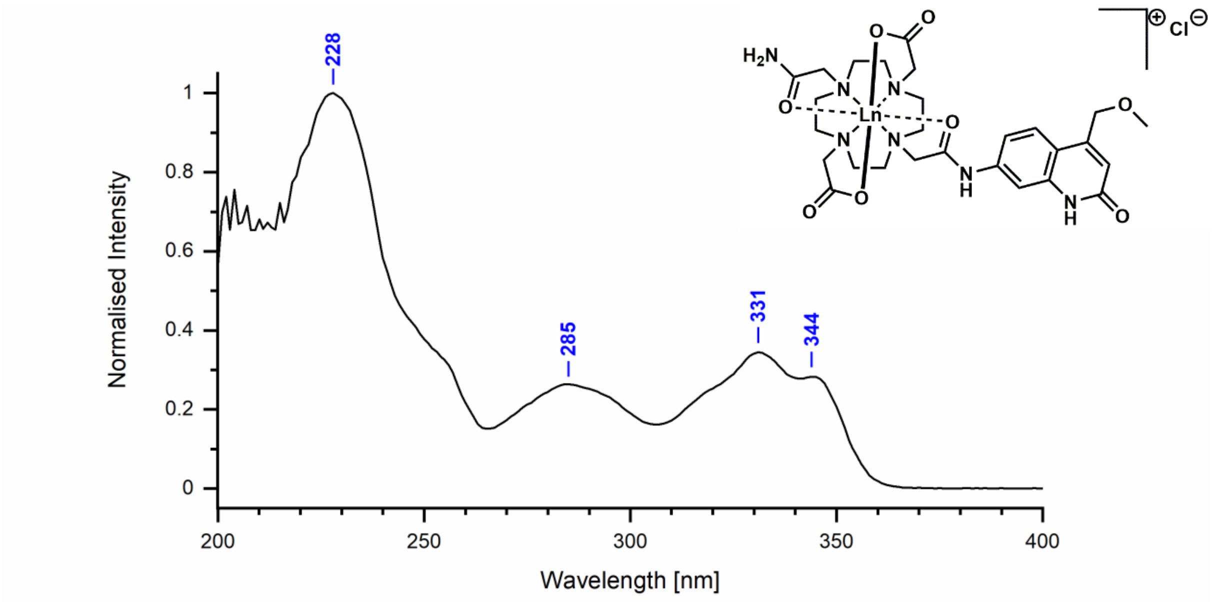

Figure S67. Normalized UV absorption spectrum of **YbL1^{MOM}** (10 µM) in 10 mM PIPES-buffered aqueous solution, pH 6.5. Blue numbers indicate the local maxima of the spectra.

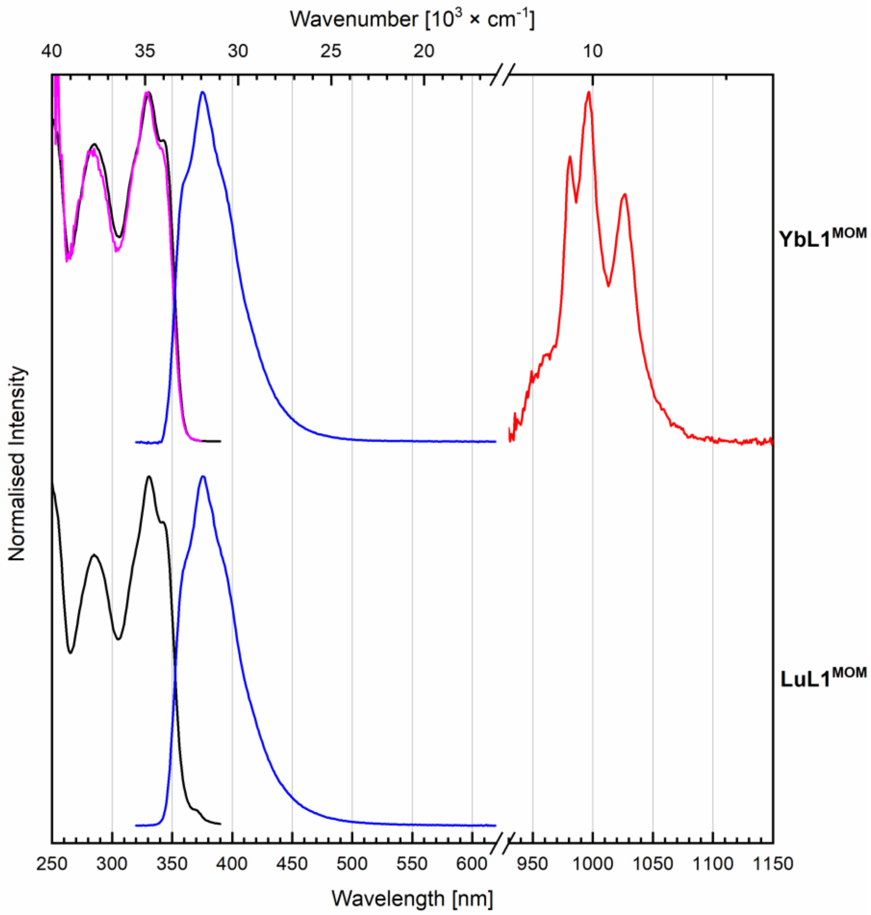

Figure S68. The excitation spectra of ligand fluorescence and Yb(III) emission (black and magenta lines, left, **YbL1^{MOM}** λ_{em} = 405 and 997 nm, respectively; **LuL1^{MOM}**, λ_{em} = 425 nm) and steady-state emission spectra of **LnL1^{MOM}** at r.t. (blue and red lines, right, Ln = Yb, λ_{ex} = 329 and 323 nm, respectively; Ln = Lu, λ_{ex} = 308 nm). [**LnL1^{MOM}**] = 10 µM, 10 mM PIPES-buffered aqueous solution, pH 6.5.

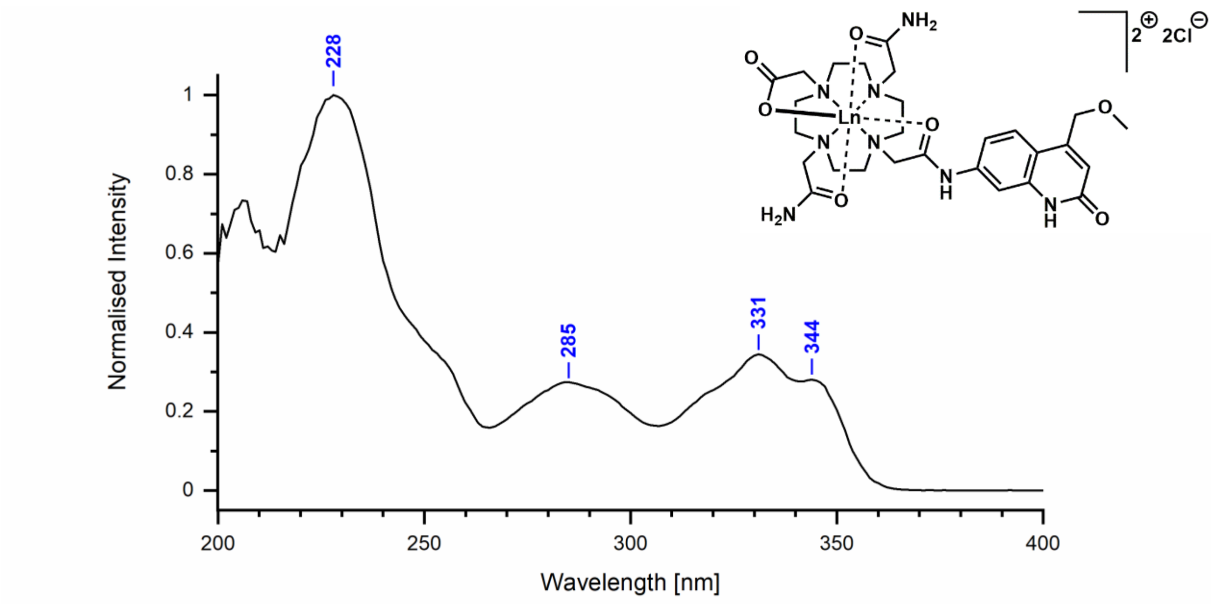

Figure S69. Normalized UV absorption spectrum of **YbL2^{MOM}** (10 μM) in 10 mM PIPES-buffered aqueous solution, pH 6.5. Blue numbers indicate the local maxima of the spectra.

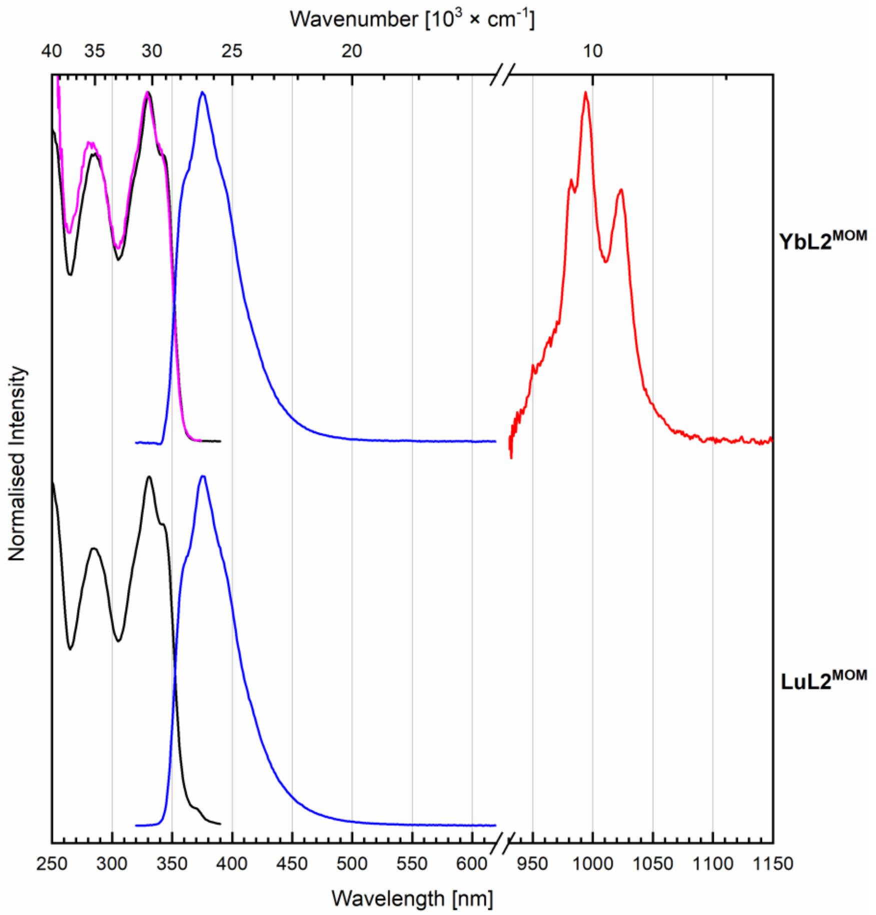

Figure S70. The excitation spectra of ligand fluorescence and Yb(III) emission (black and magenta lines, left, **YbL2^{MOM}** λ_{em} = 405 and 998 nm, respectively; **LuL2^{MOM}**, λ_{em} = 425 nm) and steady-state emission spectra of **LnL2^{MOM}** at r.t. (blue and red lines, right, Ln = Yb, λ_{ex} = 329 and 323 nm, respectively; Ln = Lu, λ_{ex} = 308 nm). [**LnL2^{MOM}**] = 10 μM, 10 mM PIPES-buffered aqueous solution, pH 6.5.

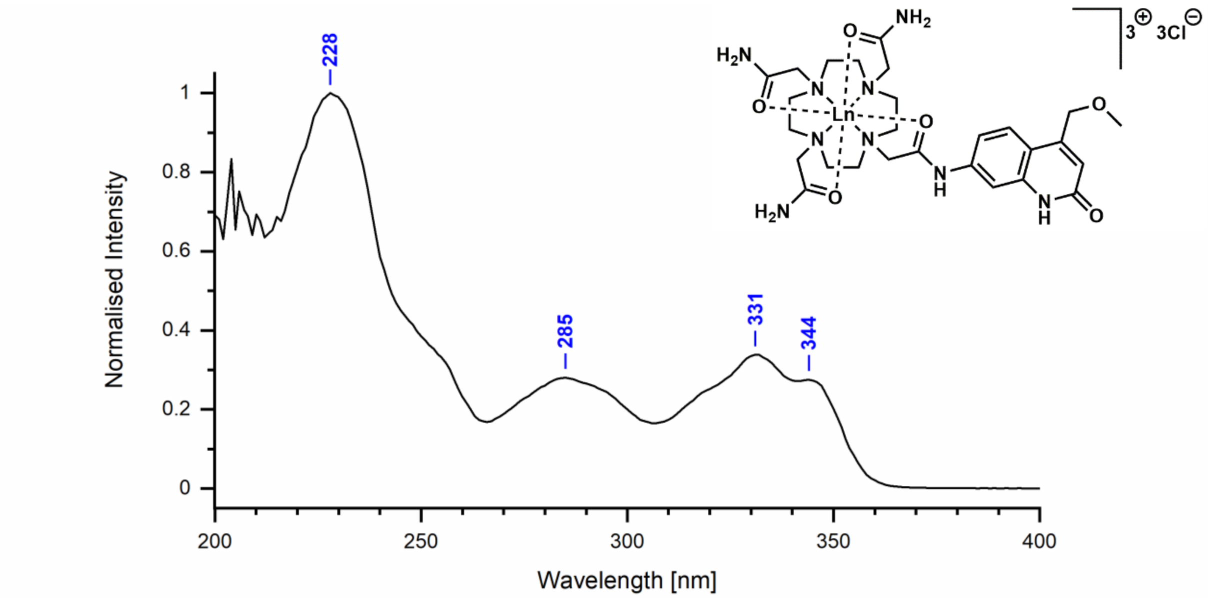

Figure S71. Normalized UV absorption spectrum of **YbL3a^{MOM}** (10 µM) in 10 mM PIPES-buffered aqueous solution, pH 6.5. Blue numbers indicate the local maxima of the spectra.

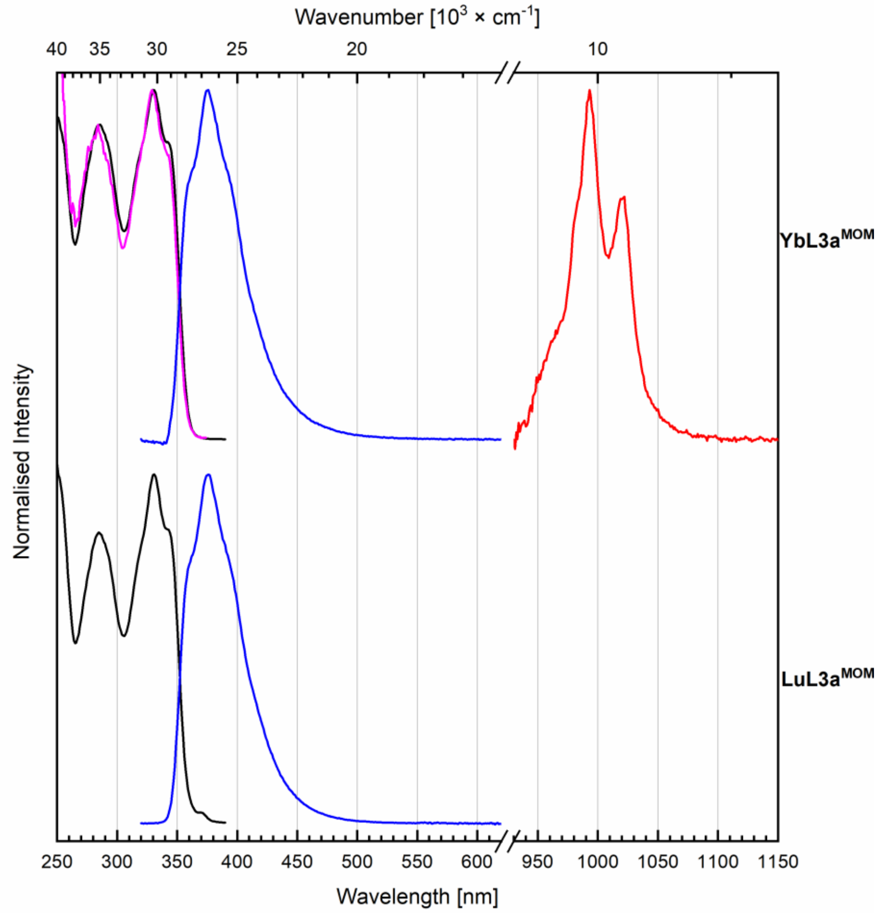

Figure S72. The excitation spectra of ligand fluorescence and Yb(III) emission (black and magenta lines, left, **YbL3a^{MOM}** λ_{em} = 405 and 998 nm, respectively; **LuL3a^{MOM}**, λ_{em} = 425 nm) and steady-state emission spectra of **LnL3a^{MOM}** at r.t. (blue and red lines, right, Ln = Yb, λ_{ex} = 329 and 323 nm, respectively; Ln = Lu, λ_{ex} = 308 nm). [**LnL3a^{MOM}**] = 10 µM, 10 mM PIPES-buffered aqueous solution, pH 6.5.

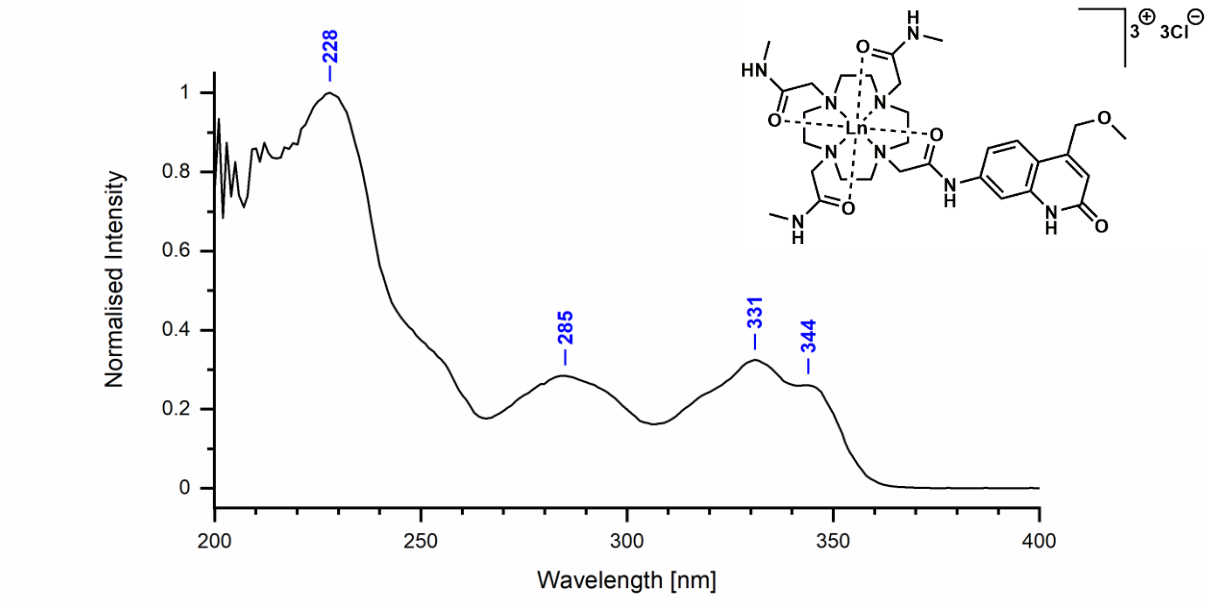

Figure S73. Normalized UV absorption spectrum of **YbL3b^{MOM}** (10 µM) in 10 mM PIPES-buffered aqueous solution, pH 6.5. Blue numbers indicate the local maxima of the spectra.

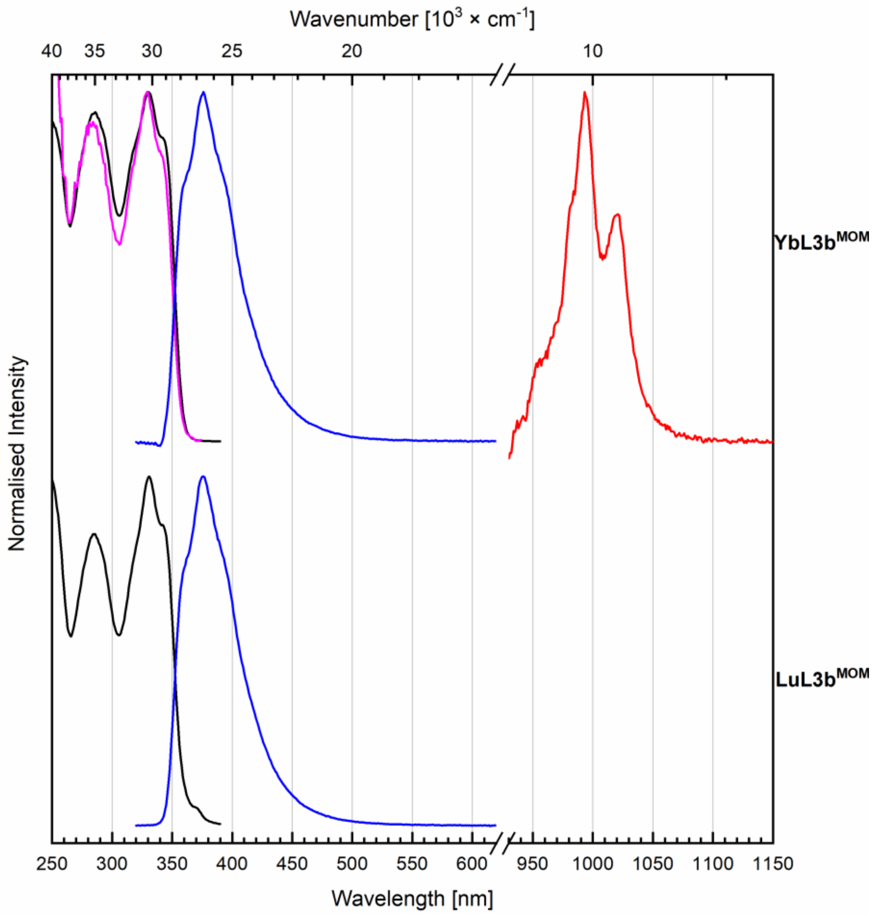

Figure S74. The excitation spectra of ligand fluorescence and Yb(III) emission (black and magenta lines, left, **YbL3b^{MOM}** λ_{em} = 405 and 997 nm, respectively; **LuL3b^{MOM}**, λ_{em} = 425 nm) and steady-state emission spectra of **LnL3b^{MOM}** at r.t. (blue and red lines, right, Ln = Yb, λ_{ex} = 329 and 323 nm, respectively; Ln = Lu, λ_{ex} = 308 nm). [**LnL3b^{MOM}**] = 10 µM, 10 mM PIPES-buffered aqueous solution, pH 6.5.

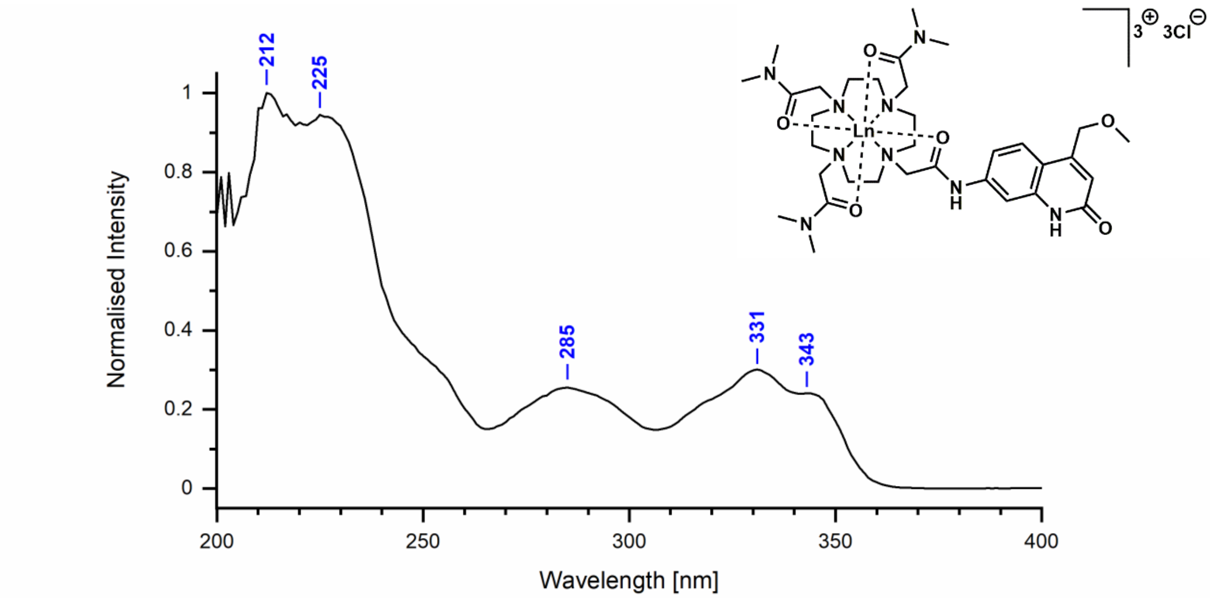

Figure S75. Normalized UV absorption spectrum of **YbL3c^{MOM}** (10 µM) in 10 mM PIPES-buffered aqueous solution, pH 6.5. Blue numbers indicate the local maxima of the spectra.

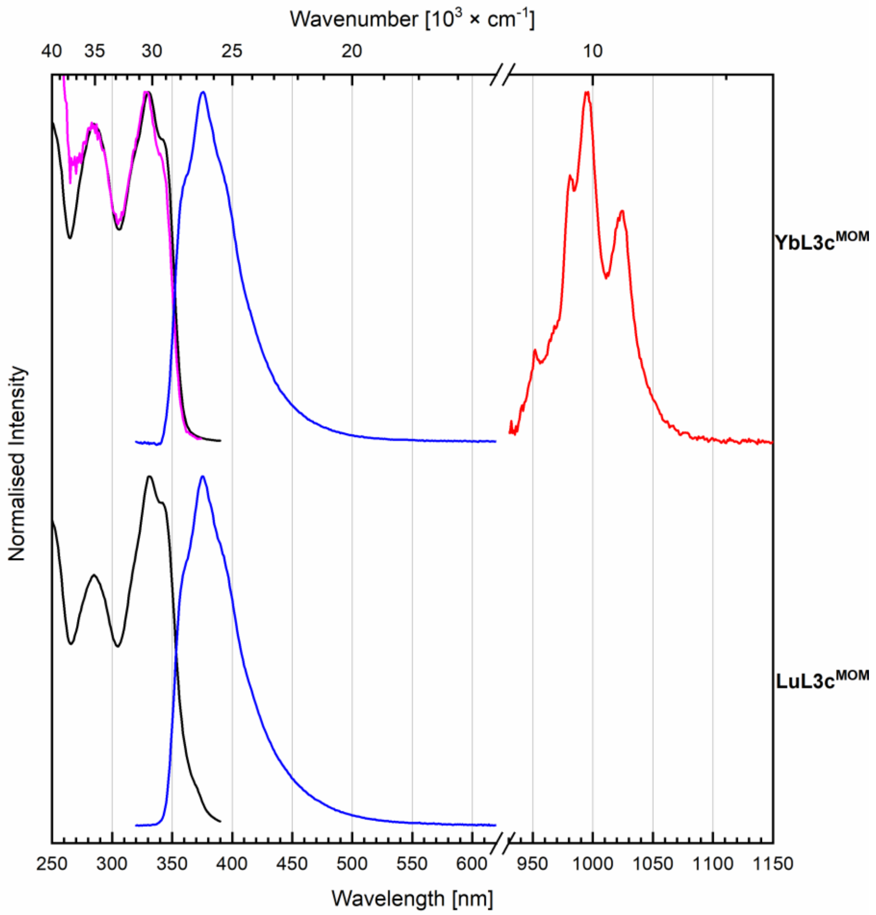

Figure S76. The excitation spectra of ligand fluorescence and Yb(III) emission (black and magenta lines, left, **YbL3c^{MOM}** λ_{em} = 405 and 997 nm, respectively; **LuL3c^{MOM}**, λ_{em} = 425 nm) and steady-state emission spectra of **LnL3c^{MOM}** at r.t. (blue and red lines, right, Ln = Yb, λ_{ex} = 329 and 323 nm, respectively; Ln = Lu, λ_{ex} = 308 nm). [**LnL3c^{MOM}**] = 10 µM, 10 mM PIPES-buffered aqueous solution, pH 6.5.

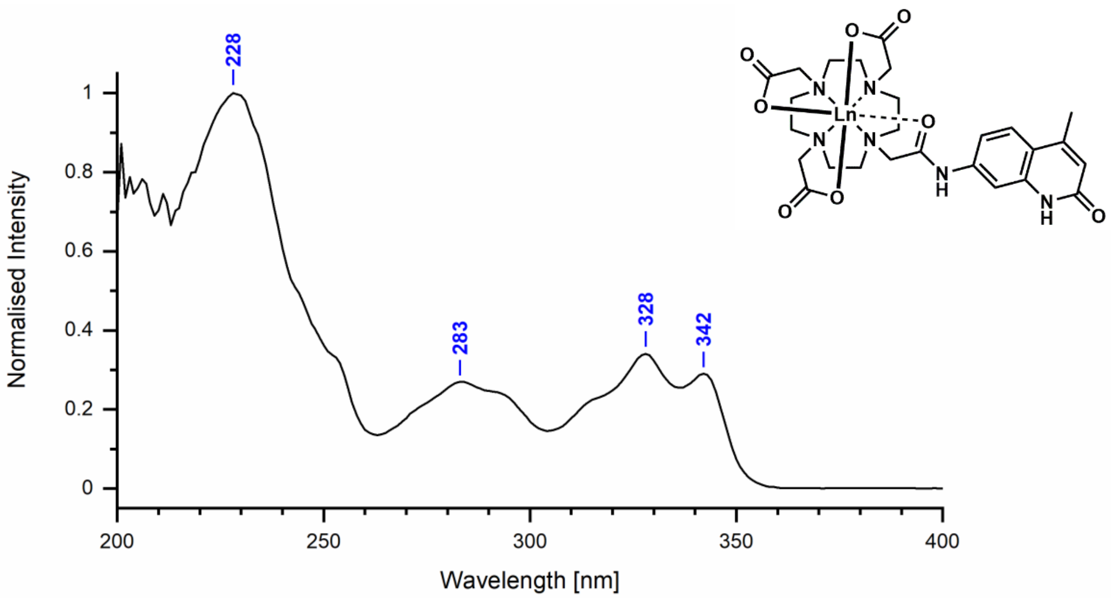

Figure S77. Normalized UV absorption spectrum of **YbL0^{Me}** (10 µM) in 10 mM PIPES-buffered aqueous solution, pH 6.5. Blue numbers indicate the local maxima of the spectra.

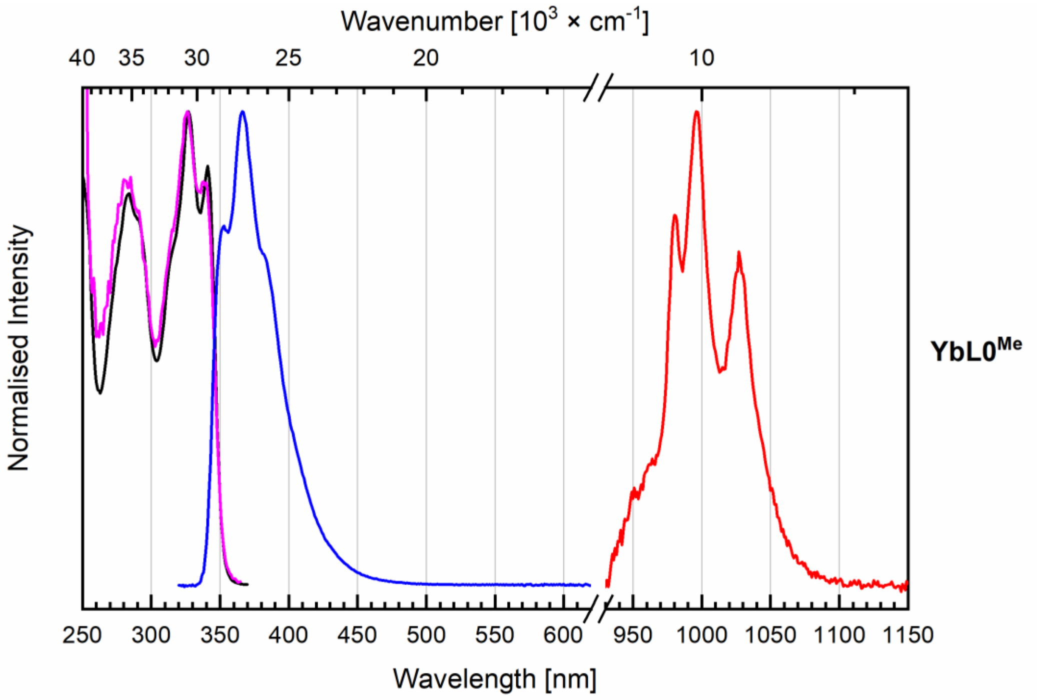

Figure S78. The excitation spectra of ligand fluorescence and Yb(III) emission (black and magenta lines, left, λ_{em} = 385 and 997 nm, respectively) and steady-state emission spectra of **YbL0^{Me}** at r.t. (blue and red lines, right, λ_{ex} = 329 and 323 nm, respectively). [**YbL0^{Me}**] = 10 µM, 10 mM PIPES-buffered aqueous solution, pH 6.5.

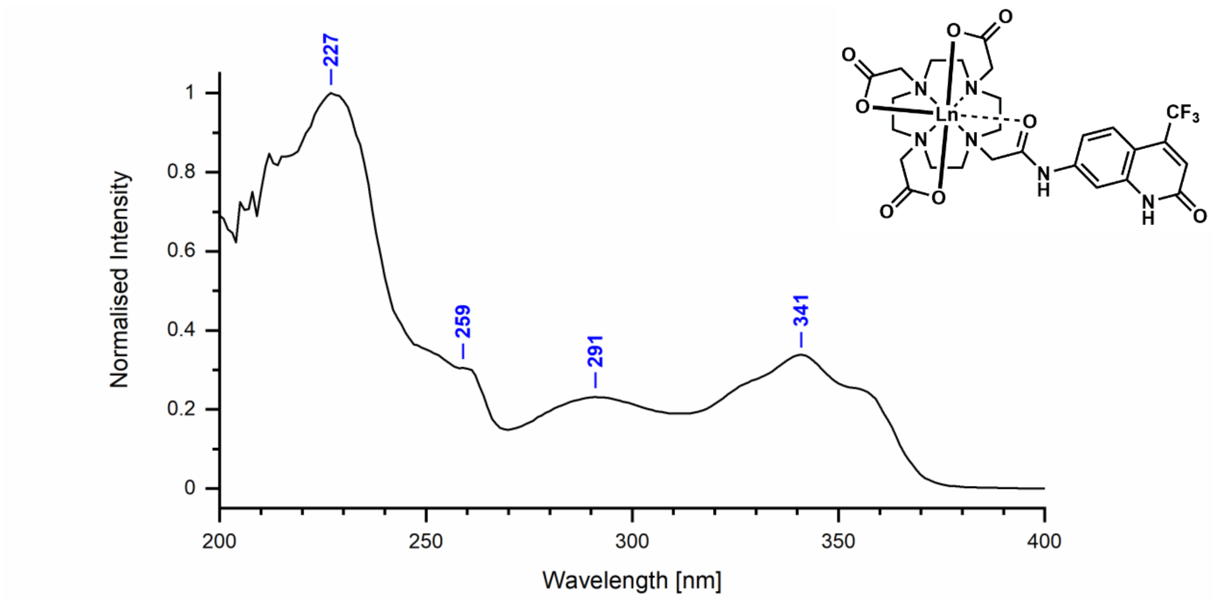

Figure S79. Normalized UV absorption spectrum of **YbL0**^{CF3} (10 µM) in 10 mM PIPES-buffered aqueous solution, pH 6.5. Blue numbers indicate the local maxima of the spectra.

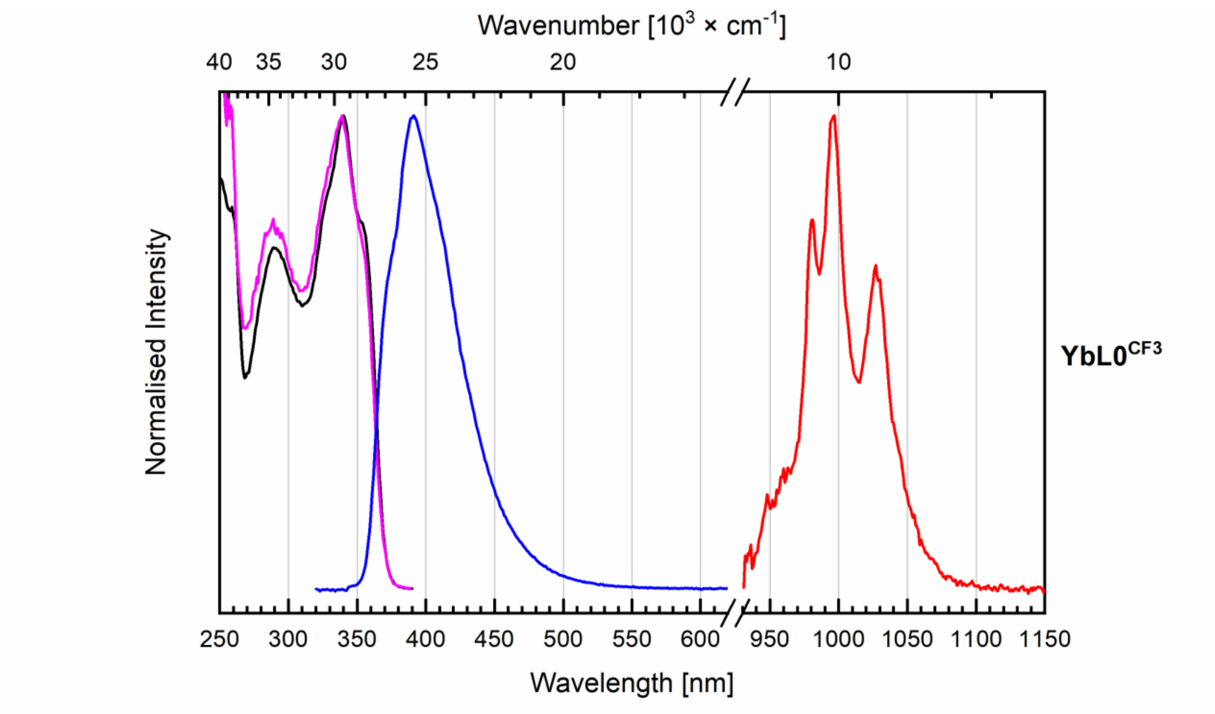

Figure S80. The excitation spectra of ligand fluorescence and Yb(III) emission (black and magenta lines, left, λ_{em} = 415 and 997 nm, respectively) and steady-state emission spectra of **YbL0**^{CF3} at r.t. (blue and red lines, right, λ_{ex} = 329 and 323 nm, respectively). [**YbL0**^{CF3}] = 10 µM, 10 mM PIPES-buffered aqueous solution, pH 6.5.

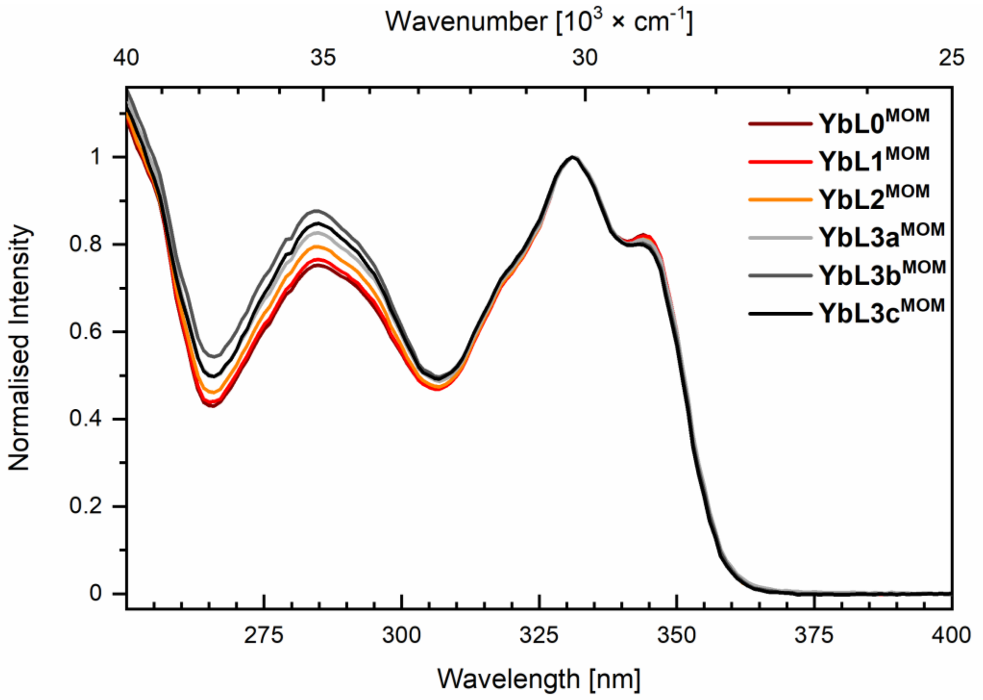

Figure S81. Superimposed normalized UV absorption spectra of **YbL**$^{\mathbf{MOM}}$ complexes in 10 mM PIPES-buffered aqueous solutions at pH 6.5. [**YbL**$^{\mathbf{MOM}}$] = 10 μM.

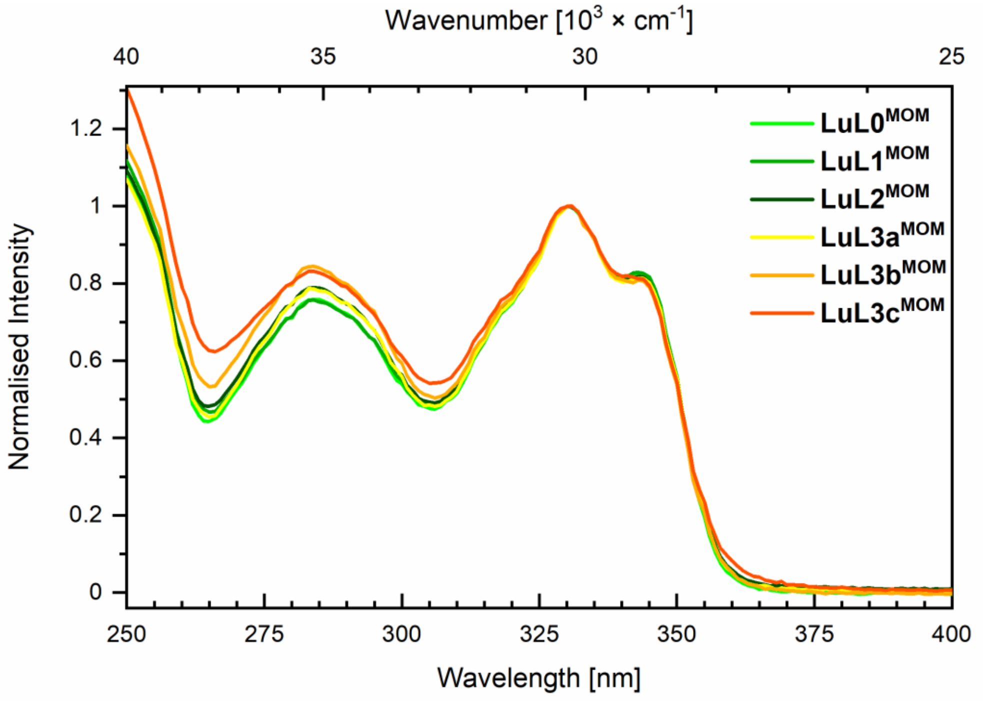

Figure S82. Superimposed normalized UV absorption spectra of **LuL**$^{\mathbf{MOM}}$ complexes in 10 mM PIPES-buffered aqueous solutions at pH 6.5. [**LuL**$^{\mathbf{MOM}}$] = 10 μM.

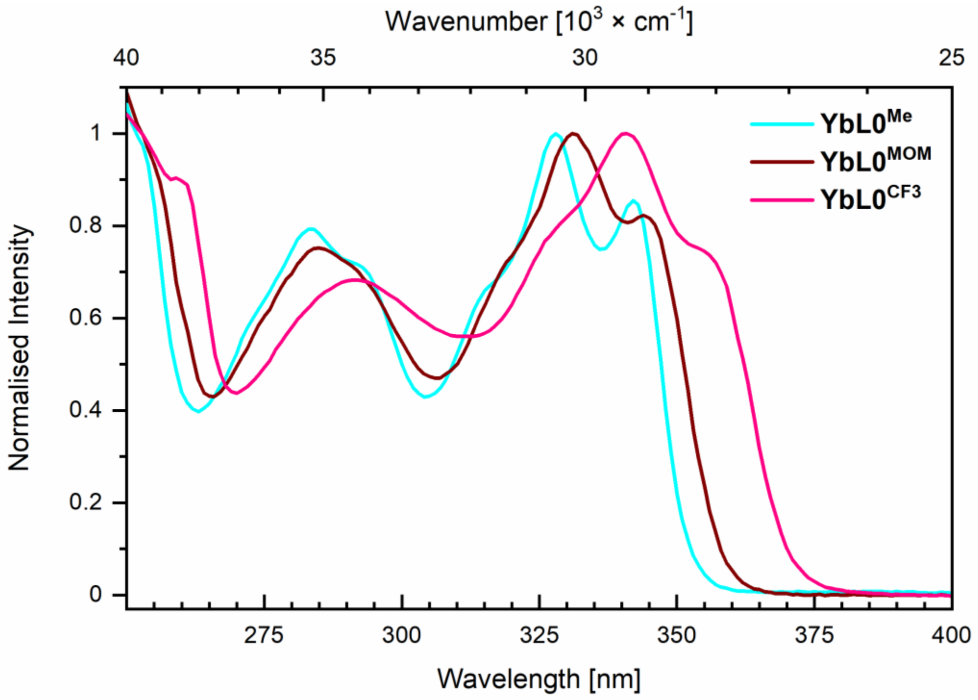

Figure S83. Superimposed normalized UV absorption spectra of **YbL0** complexes in 10 mM PIPES-buffered aqueous solutions at pH 6.5. [**YbL0**] = 10 µM.

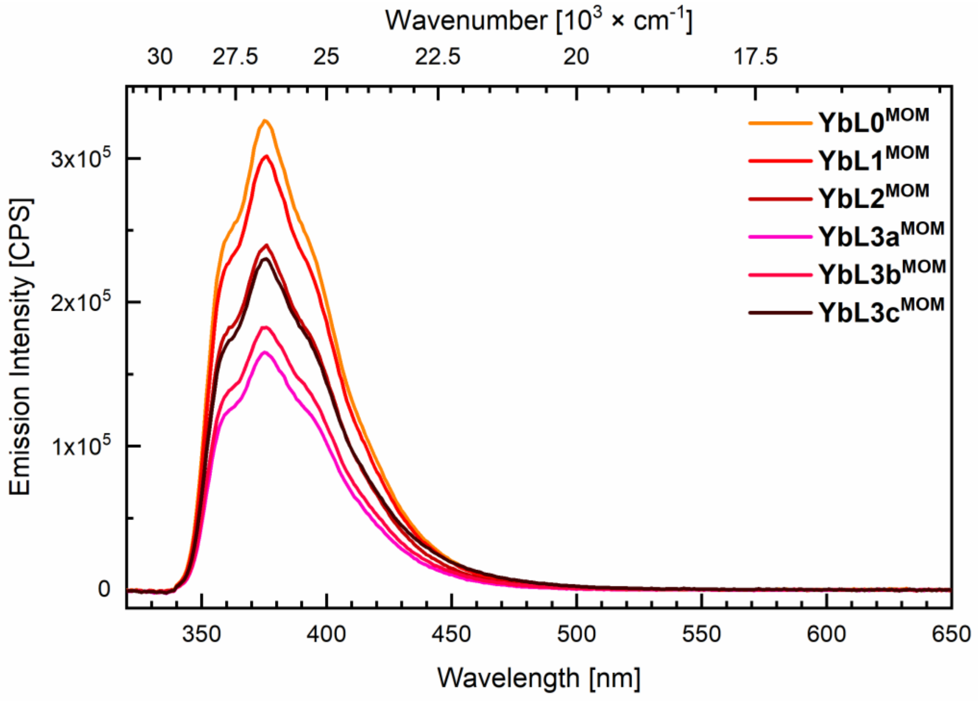

Figure S84. Steady-state fluorescence spectra of **YbL**^{MOM} complexes indicating relative emission intensities under identical samples absorptions. [**YbL**^{MOM}] = 10 µM in 10 mM PIPES-buffered solutions, pH 6.5, λ_{ex} = 329.5 nm, front slit: 2 nm, exit slit: 1.5 nm.

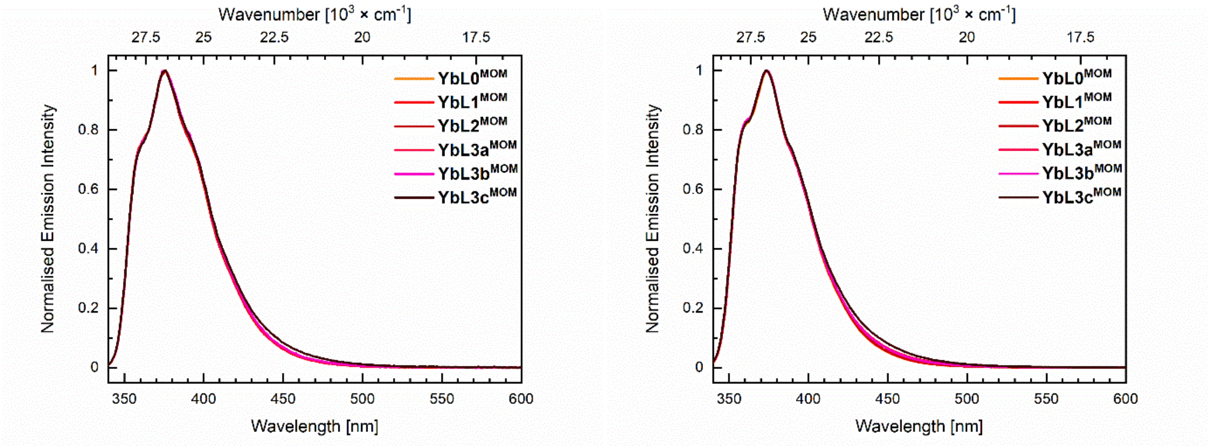

Figure S85. Normalized steady-state fluorescence spectra of **YbL^{MOM}** complexes under identical samples absorptions from two independent measurements. [**YbL^{MOM}**] = 10 µM in 10 mM PIPES-buffered solutions, pH 6.5; left: λ_{ex} = 329.5 nm, front slit: 2 nm, exit slit: 1.5 nm; right: λ_{ex} = 323 nm, front slit: 8–14 nm, exit slit: 1 nm.

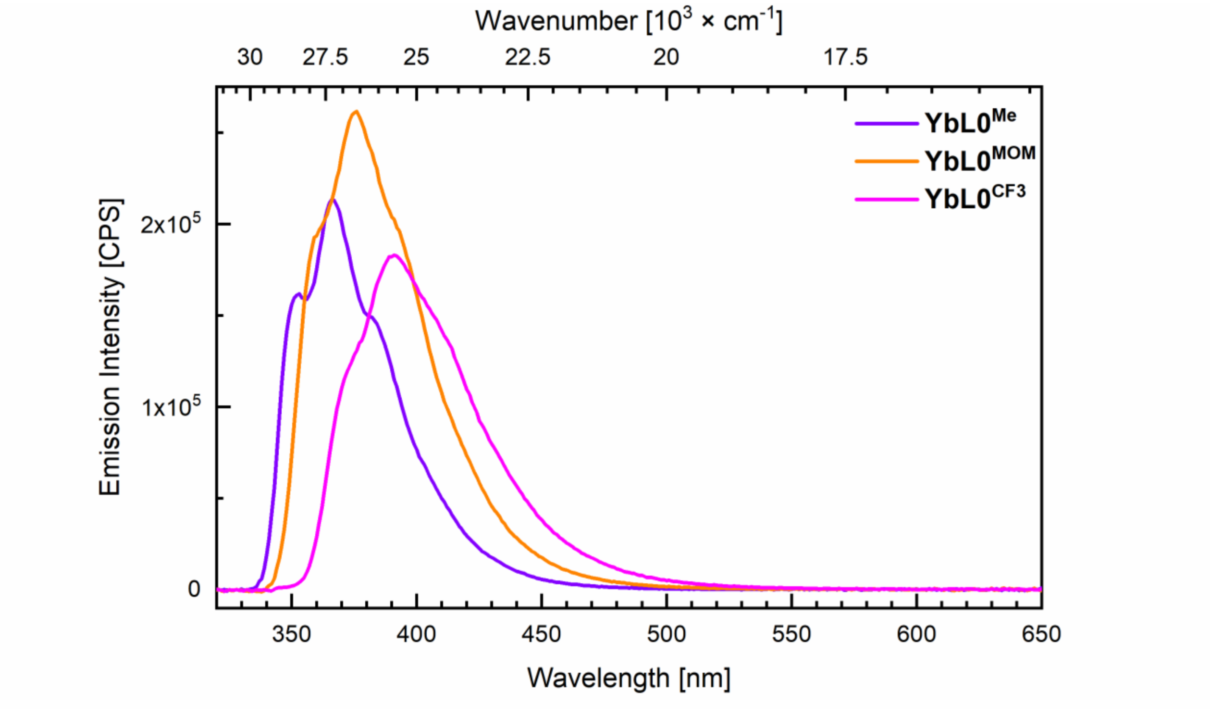

Figure S86. Steady-state fluorescence spectra of **YbL0** complexes indicating relative emission intensities under identical samples absorptions. [**YbL0**] = 10 µM in 10 mM PIPES-buffered solutions, pH 6.5, λ_{ex} = 329 nm, front slit: 2 nm, exit slit: 1.5 nm.

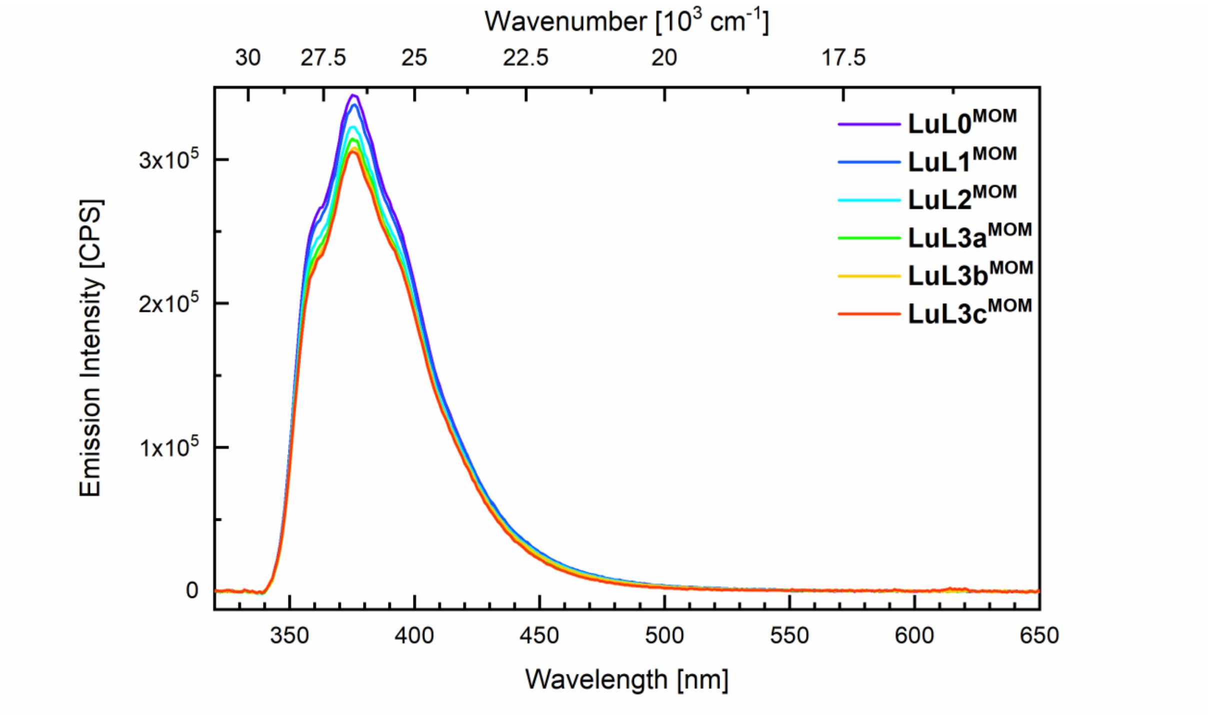

Figure S87. Steady-state fluorescence spectra of **LuL**$^{\mathbf{MOM}}$ complexes indicating relative emission intensities under identical samples absorptions. [**LuL**$^{\mathbf{MOM}}$] = 10 µM in 10 mM PIPES-buffered solutions, pH 6.5, λ_{ex} = 308 nm, front slit: 2 nm, exit slit: 2 nm.

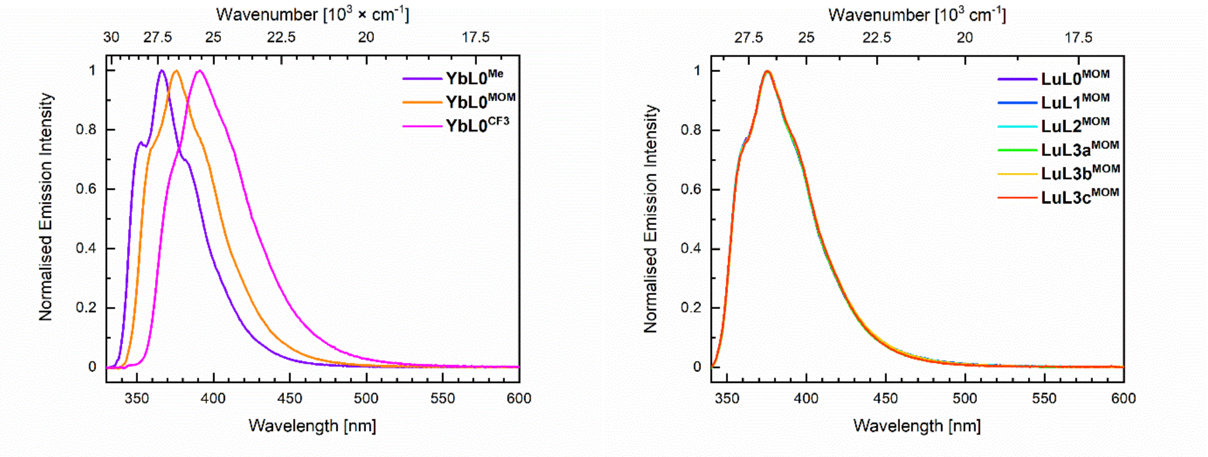

Figure S88. Normalized steady-state fluorescence spectra of **YbL0** (left) and **LuL**$^{\mathbf{MOM}}$ (right) complexes under identical samples absorptions. [**LnL**] = 10 µM in 10 mM PIPES-buffered solutions, pH 6.5; left: λ_{ex} = 329 nm, front slit: 2 nm, exit slit: 1.5 nm; right: λ_{ex} = 308 nm, front slit: 2 nm, exit slit: 2 nm.

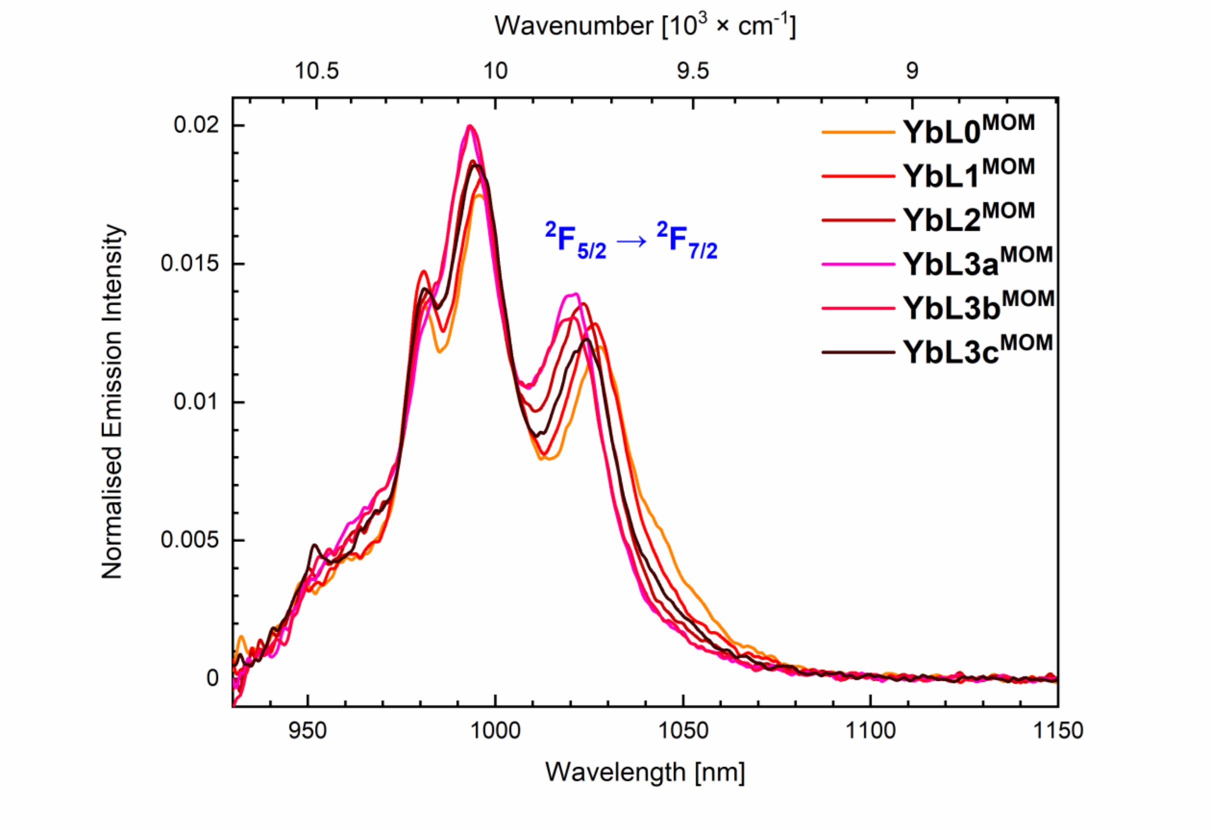

Figure S89. Emission spectra of the **YbL^{MOM}** complexes (A = 0.1) in 10 mM PIPES in H_2O, pH 6.5, λ_{ex} = 323 nm.

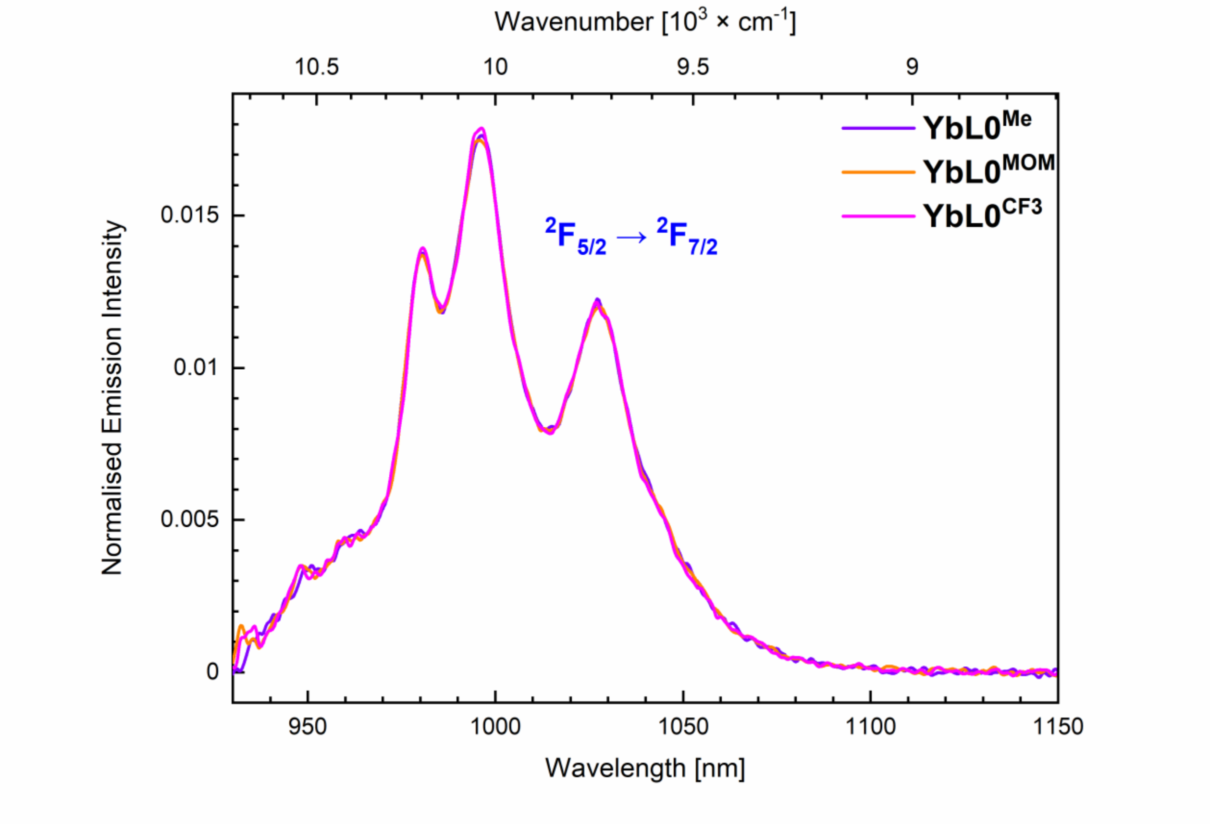

Figure S90. Emission spectra of the **YbL0** complexes (A = 0.1) in 10 mM PIPES in H_2O, pH 6.5, λ_{ex} = 323 nm.

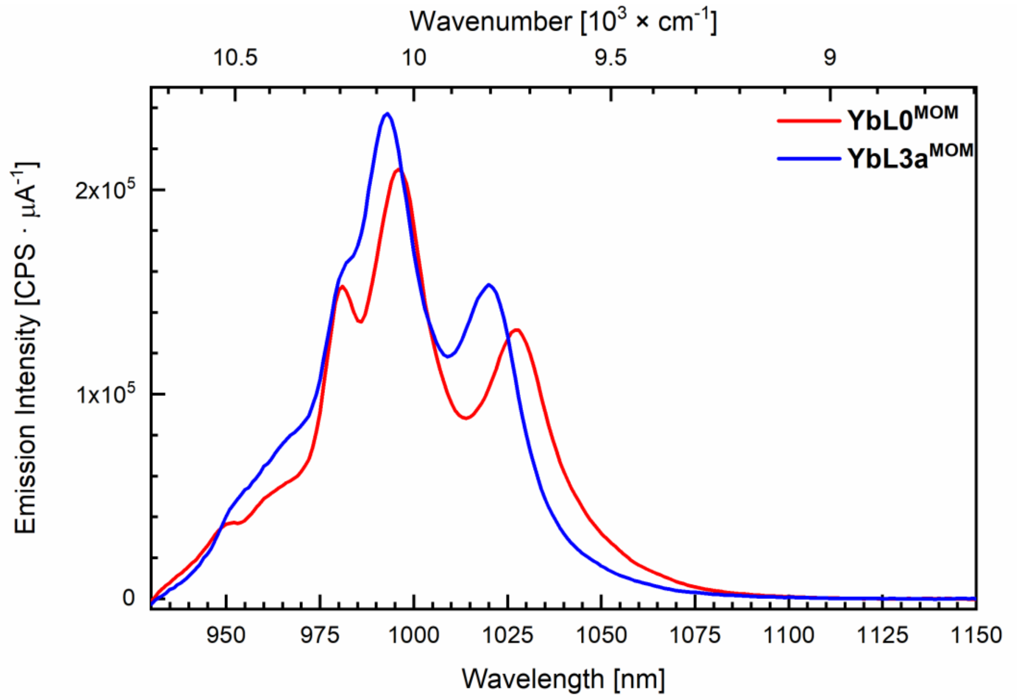

Figure S91. Emission spectra of the **YbL0,3a^{MOM}** complexes (A = 0.1) in 10 mM PIPES in D_2O, pD 6.9, λ_{ex} = 331 nm.

Table S13. Residual ligand fluorescence quantum yields from several independent experiments of **LnL** (Ln = Yb, Lu) in H_2O. Measurements were performed with [**LnL**] = 10 µM in 10 mM PIPES-buffered H_2O at pH 6.5.

Complex	$\Phi_{L,1}$ [a] [%]	$\Phi_{L,2}$ [a] [%]	$\Phi_{L,3}$ [a] [%]	$\Phi_{L,av}$ [b] [%]	St. Dev. [%]
**YbL0**^{MOM}	5.93	5.85	5.81	5.87	0.05
**YbL1**^{MOM}	5.47	5.38	-	5.43	0.05
**YbL2**^{MOM}	4.35	4.27	-	4.31	0.04
**YbL3a**^{MOM}	3.06	3.13	3.00	3.07	0.03
**YbL3b**^{MOM}	3.39	3.53	-	3.46	0.07
**YbL3c**^{MOM}	4.35	4.35	-	4.35	0
**YbL0**^{CF3}	4.65	4.88	-	4.77	0.11
**YbL0**^{Me}	4.20	4.17	4.23	4.20	0.03
**LuL0**^{MOM}	8.00	8.39	8.50	8.30	0.21
**LuL1**^{MOM}	7.94	8.15	9.11	8.40	0.51
**LuL2**^{MOM}	7.58	7.82	8.52	7.97	0.40
**LuL3a**^{MOM}	7.29	7.53	8.26	7.69	0.41
**LuL3b**^{MOM}	7.30	7.43	8.17	7.63	0.38
**LuL3c**^{MOM}	7.15	-	-	7.15	-

[a] Determined relative to QS (Φ = 0.59 [5]) in H_2SO_4 (0.05 M). [b] Average quantum yield from two or three independent measurements.

Table S14. Residual ligand fluorescence quantum yields from several independent experiments of **LnL** (Ln = Yb, Lu) in D_2O. Measurements were performed with [**LnL**] = 10 µM in 10 mM PIPES-buffered D_2O at pD 6.9.[6]

Complex	$\Phi_{L,1}$ [a] [%]	$\Phi_{L,2}$ [a] [%]	$\Phi_{L,3}$ [a] [%]	$\Phi_{L,av}$ [b] [%]	St. Dev. [%]
**YbL0^{MOM}**	5.49	6.35	5.86	5.90	0.35
**YbL1^{MOM}**	5.05	5.97	-	5.51	0.46
**YbL2^{MOM}**	4.04	4.51	-	4.28	0.24
**YbL3a^{MOM}**	3.44	3.48	3.09	3.34	0.18
**YbL3b^{MOM}**	3.39	3.83	-	3.61	0.22
**YbL3c^{MOM}**	4.71	4.83	-	4.77	0.06
**LuL0^{MOM}**	8.40	8.35	-	8.35	0.05
**LuL1^{MOM}**	8.14	8.46	-	8.30	0.16
**LuL2^{MOM}**	7.89	8.25	-	8.07	0.18
**LuL3a^{MOM}**	7.35	7.97	-	7.66	0.31
**LuL3b^{MOM}**	8.37	8.23	-	8.30	0.07
**LuL3c^{MOM}**	8.86	-	-	8.86	-

[a] Determined relative to QS (Φ = 0.59 [5]) in H_2SO_4 (0.05 M). [b] Average quantum yield from two or three independent measurements.

Table S15. Antenna fluorescence quantum yields and lifetimes ($\tau_{f,L}$) of **LnL^MOM** (Ln = Yb, Lu) fluorescence in D_2O (λ_{ex} = 341.5 nm, λ_{em} = 377 nm, monoexponential reconvolution fit), and calculated $\tau_{rad,L}$, $k_{rad,L}$, $k_{f,L}$ (s^{-1}), $k_{nr,L}$, and k_{PeT}.

Compound	Φ_L [%] (Rel. Φ_L [%])	$\tau_{f,L}$ (ns)	$\tau_{rad,L}$ (ns)	$k_{rad,L}$ (ns^{-1})	$k_{f,L}$ (ns^{-1})	$k_{nr,L}$ (ns^{-1})	k_{PeT} (ns^{-1})
**LuL0^MOM**	8.35	0.46	5.49	0.182	2.18	2.00	-
**LuL1^MOM**	8.30	0.47	5.63	0.178	2.14	1.96	-
**LuL2^MOM**	8.07	0.45	5.54	0.180	2.24	2.06	-
**LuL3a^MOM**	7.66	0.45	5.82	0.172	2.24	2.07	-
**LuL3b^MOM**	8.30	0.44	5.17	0.193	2.33	2.14	-
**LuL3c^MOM**	8.86	0.42	4.71	0.212	2.40	2.19	-
**YbL0^MOM**	5.90	0.31	5.30	0.189	3.20	3.01	1.01
**YbL1^MOM**	5.51	0.30	5.51	0.181	3.29	3.11	1.15
**YbL2^MOM**	4.28	0.24	5.71	0.175	4.09	3.92	1.86
**YbL3a^MOM**	3.34	0.22	6.52	0.153	4.59	4.44	2.37
**YbL3b^MOM**	3.61	0.21	5.81	0.172	4.76	4.59	2.46
**YbL3c^MOM**	4.77	0.26	5.54	0.181	3.79	3.61	1.42

Measurements were performed in 10 mM PIPES buffer solutions in D_2O at pD 6.5, [**LnL^MOM**] = 10 µM. [a] Determined relative to quinine sulfate (Φ = 0.59) in H_2SO_4 (0.05 M) in H_2O.[5] $k_{f,L}$, $\tau_{rad,L}$, $k_{rad,L}$, $k_{nr,L}$, and k_{PeT} were calculated as follows: $k_{f,L} = 1/\tau_{f,L}$, $\tau_{rad,L} = \tau_{f,L}/\Phi_L$, $k_{rad,L} = 1/\tau_{rad,L}$, $k_{nr,L} = k_{f,L} - k_{rad,L}$, $k_{PeT} \approx k_{nr,L}(Yb) - k_{nr,L}(Lu)$.

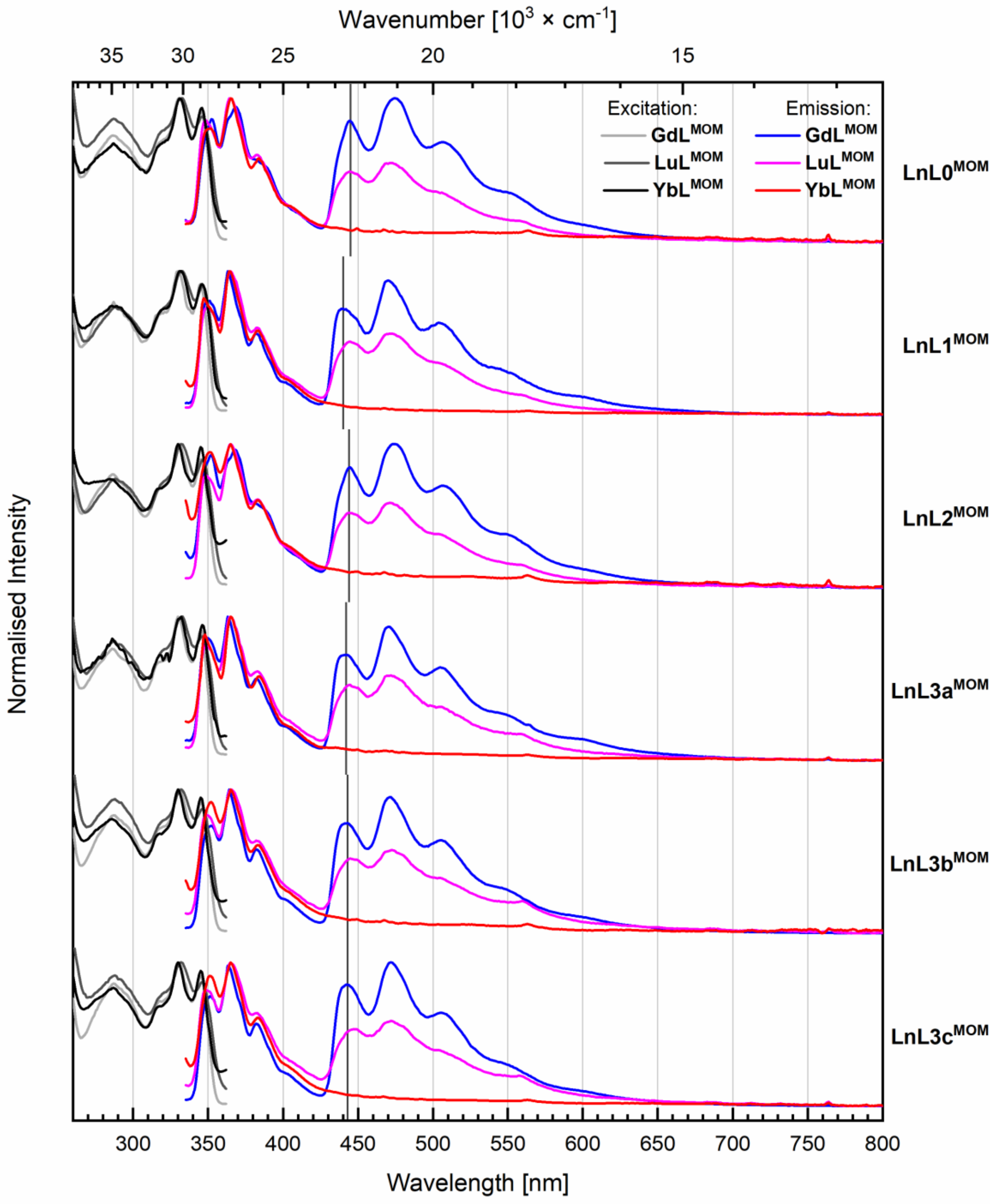

Figure S92. Ligand phosphorescence excitation and steady-state emission spectra of **LnL**$^{\mathbf{MOM}}$ (Ln = Gd, Yb, Lu) at 77 K with 10% glycerol added to 10 mM PIPES-buffered aqueous solutions (pH 6.5). [**LnL**$^{\mathbf{MOM}}$] = 10 μM, λ_{ex} = 344 (Ln = Gd), 329 (Ln = Yb), 308 nm (Ln = Lu); λ_{em} = 442 (Ln = Gd), 385 (Ln = Yb), 390 nm (Ln = Lu). The dark grey lines are at the maxima of the first visible vibronic component of the phosphorescence spectra (λ_{em} = 441–445 nm for **GdL**$^{\mathbf{MOM}}$).

Table S16. Integration of S_1 and T_1 emission band of **LnL^{MOM}** (Ln = Yb, Gd, Lu) from the normalized emission spectra recorded at 77 K, and ratios S_1/T_1. Ligand steady-state emission spectra of **LnL^{MOM}** (Ln = Gd, Yb, Lu) were recorded at 77 K with 10% glycerol added to 10 mM PIPES-buffered aqueous solutions (pH 6.5). Integration ranges: 335–426 nm (S_1), 426–750 nm (T_1).

	**Yb**			**Gd**			**Lu**		
	I_{S1}	I_{T1}	I_{S1}/I_{T1}	I_{S1}	I_{T1}	I_{S1}/I_{T1}	I_{S1}	I_{T1}	I_{S1}/I_{T1}
**L0^{MOM}**	42.7	16.6	2.57	43.1	94.9	0.45	44.6	52.1	0.86
**L1^{MOM}**	41.9	7.79	5.38	39.2	86.4	0.45	42.4	52.8	0.80
**L2^{MOM}**	49.6	18.7	2.65	46.0	97.1	0.47	43.4	55.9	0.78
**L3a^{MOM}**	42.9	10.3	4.17	41.1	88.7	0.46	44.3	57.6	0.77
**L3b^{MOM}**	47.2	13.8	3.42	38.7	86.4	0.45	47.3	61.3	0.77
**L3c^{MOM}**	46.4	10.8	4.30	38.7	88.7	0.44	47.7	61.4	0.78
**L3a^{MOM*}**	54.7	38.9	1.40	45.2	93.6	0.48	-	-	
**L3a$^{MOM\#}$**				51.1	89.2	0.57	-	-	

* under Ar, λ_{ex} = 331 nm.

# under air, λ_{ex} = 331.

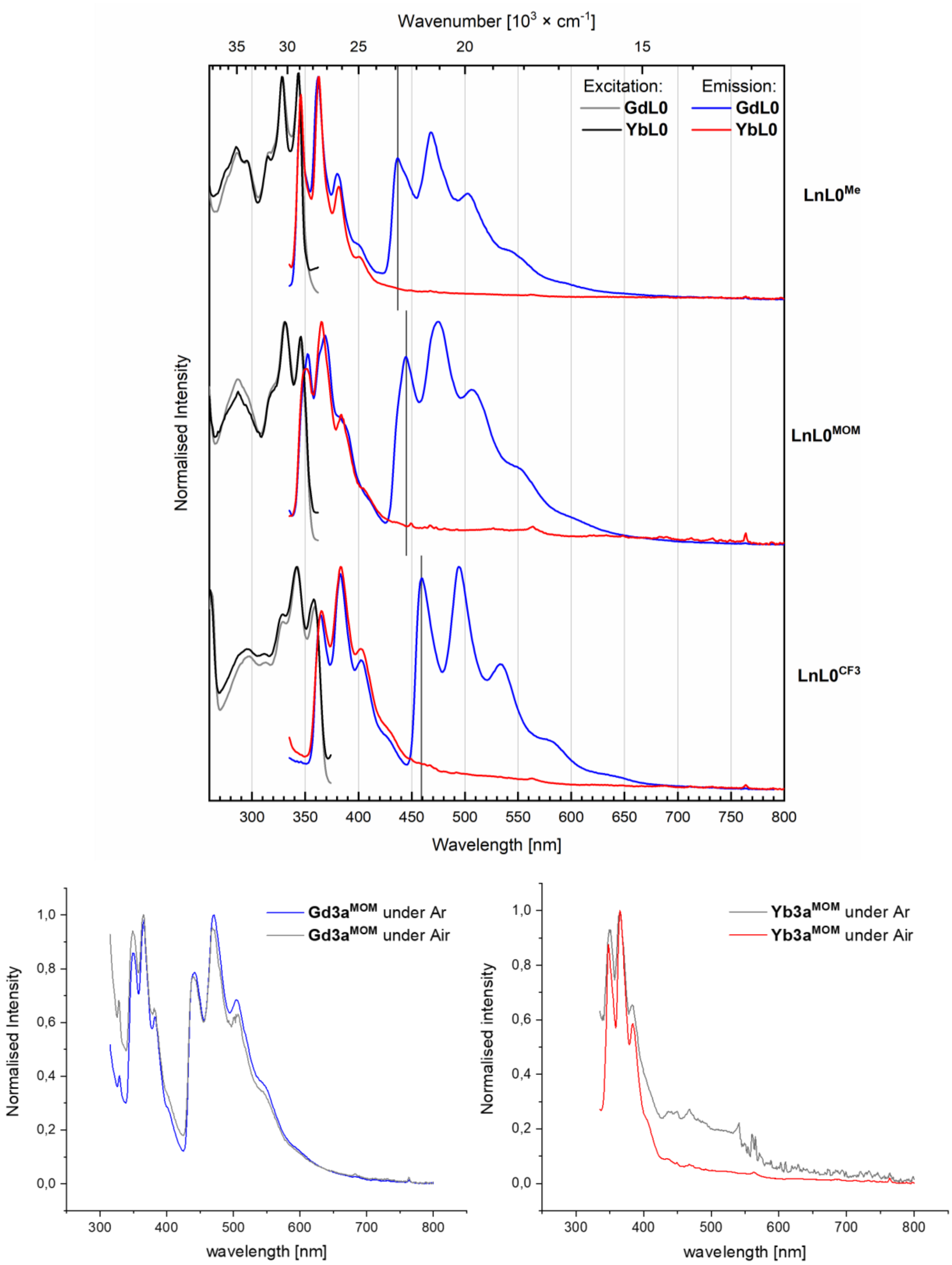

Figure S93. **Top**: Ligand phosphorescence excitation and steady-state emission spectra of **LnL0** (Ln = Gd, Yb) at 77 K with 10% glycerol added to 10 mM PIPES-buffered aqueous solutions (pH 6.5). [**LnL0**] = 10 µM, λ_{ex} = 344 (Ln = Gd), 329 (Ln = Yb); λ_{em} = 437–459 (Ln = Gd), 385 (Ln = Yb). The dark grey lines are at the maxima of the first visible vibronic component of the phosphorescence spectra (λ_{em} = 437–459 nm for **GdL^{MOM}**). **Bottom**: Steady-state emission spectra of **GdL3a^{MOM}** (left) and **Yb3La^{MOM}** (right) of samples prepared under air or Ar, λ_{ex} = 331 nm for **Yb3a^{MOM}** under Ar.

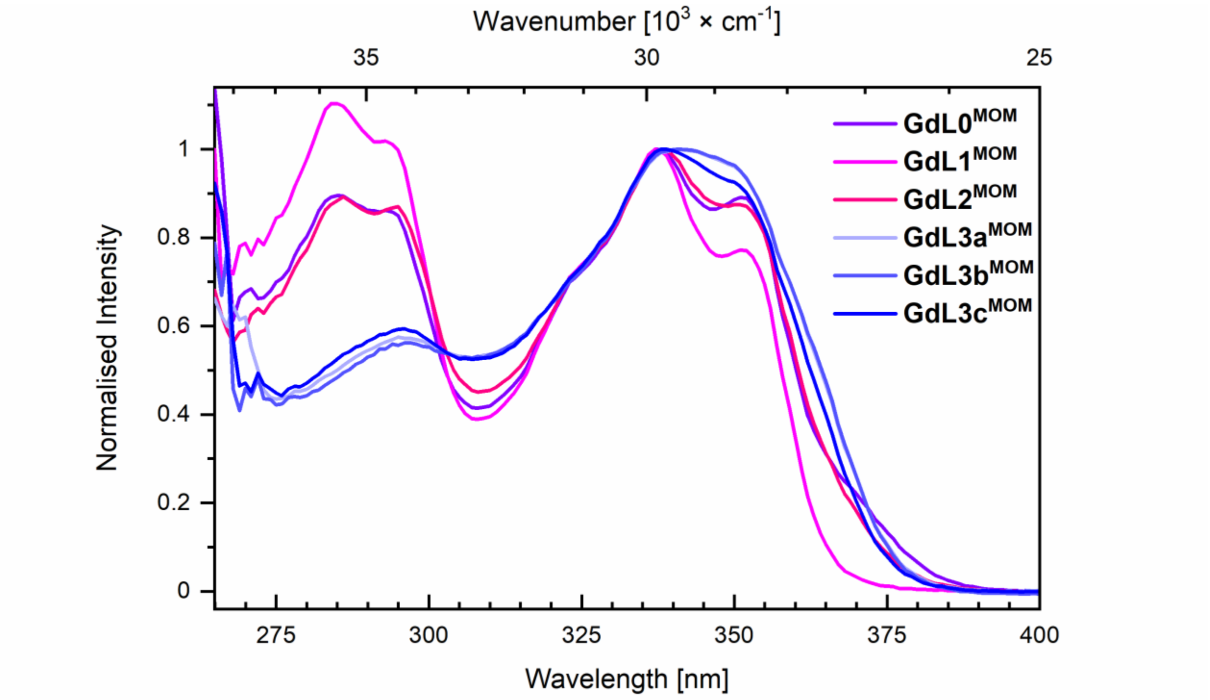

Figure S94. Superimposed normalized UV absorption spectra of **GdL^{MOM}** in DMF. [**GdL^{MOM}**] = 10 µM.

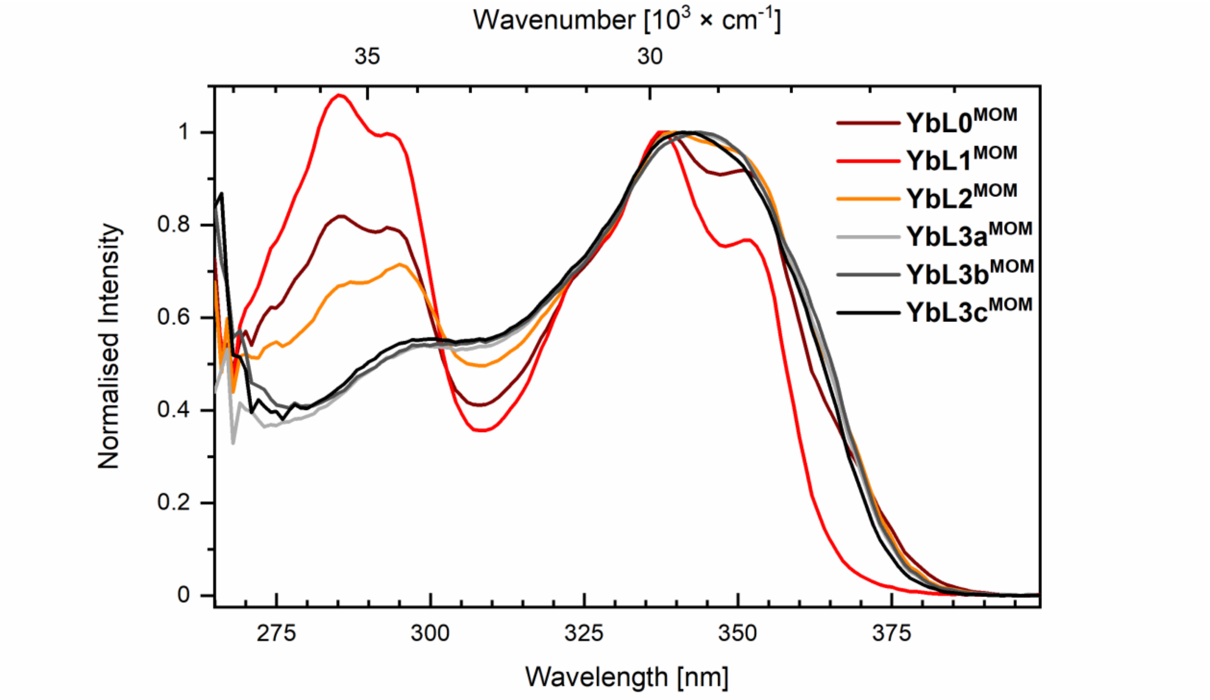

Figure S95. Superimposed normalized UV absorption spectra of **YbL^{MOM}** in DMF. [**YbL^{MOM}**] = 10 µM.

Table S17. Residual ligand fluorescence quantum yields of **LnL**^{MOM} (Ln = Gd, Yb) in DMF.

Complex	Φ_L [a] [%] (Rel. Φ_L [%])	Complex	Φ_L [a] [%] (Rel. Φ_L [%])
**YbL0**^{MOM}	2.9 (100)	**GdL0**^{MOM}	4.7 (100)
**YbL1**^{MOM}	3.3 (115)	**GdL1**^{MOM}	3.7 (80)
**YbL2**^{MOM}	1.5 (53)	**GdL2**^{MOM}	3.3 (71)
**YbL3a**^{MOM}	1.1 (37)	**GdL3a**^{MOM}	2.1 (46)
**YbL3b**^{MOM}	0.8 (26)	**GdL3b**^{MOM}	2.3 (48)
**YbL3c**^{MOM}	0.9 (30)	**GdL3c**^{MOM}	2.9 (61)

[a] Determined relative to QS (Φ = 0.59[5]) in water containing H_2SO_4 (0.05 M), using n = 1.4305.[23]

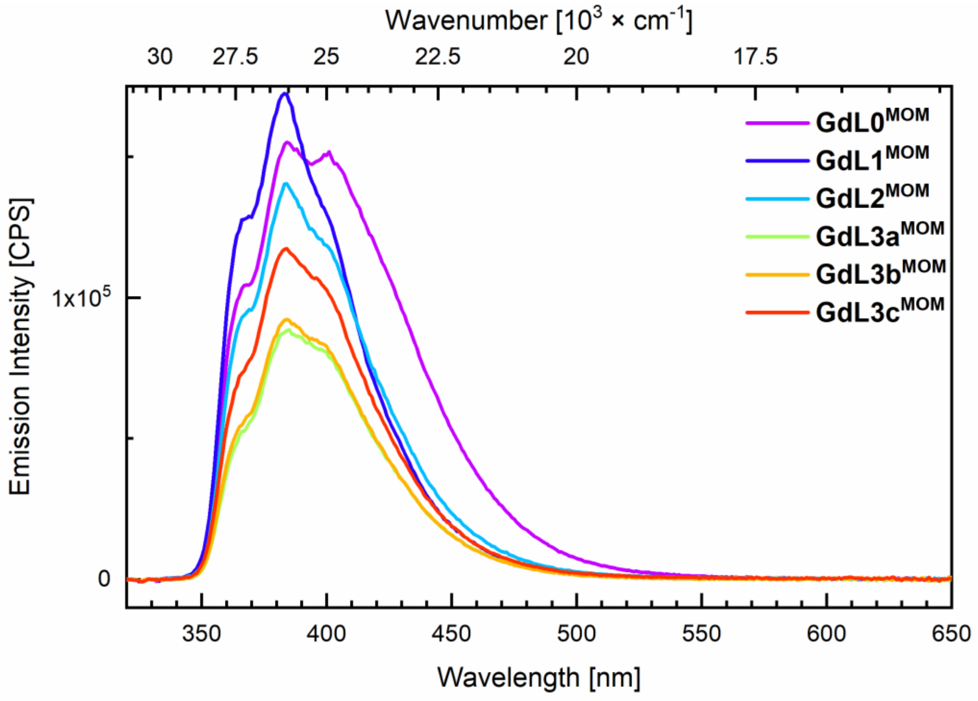

Figure S96. Steady-state fluorescence spectra of **GdL**^{MOM} complexes indicating relative emission intensities under identical samples absorptions. [**GdL**^{MOM}] = 10 μM in DMF, λ_{ex} = 335 nm, front slit: 2 nm, exit slit: 1.5 nm.

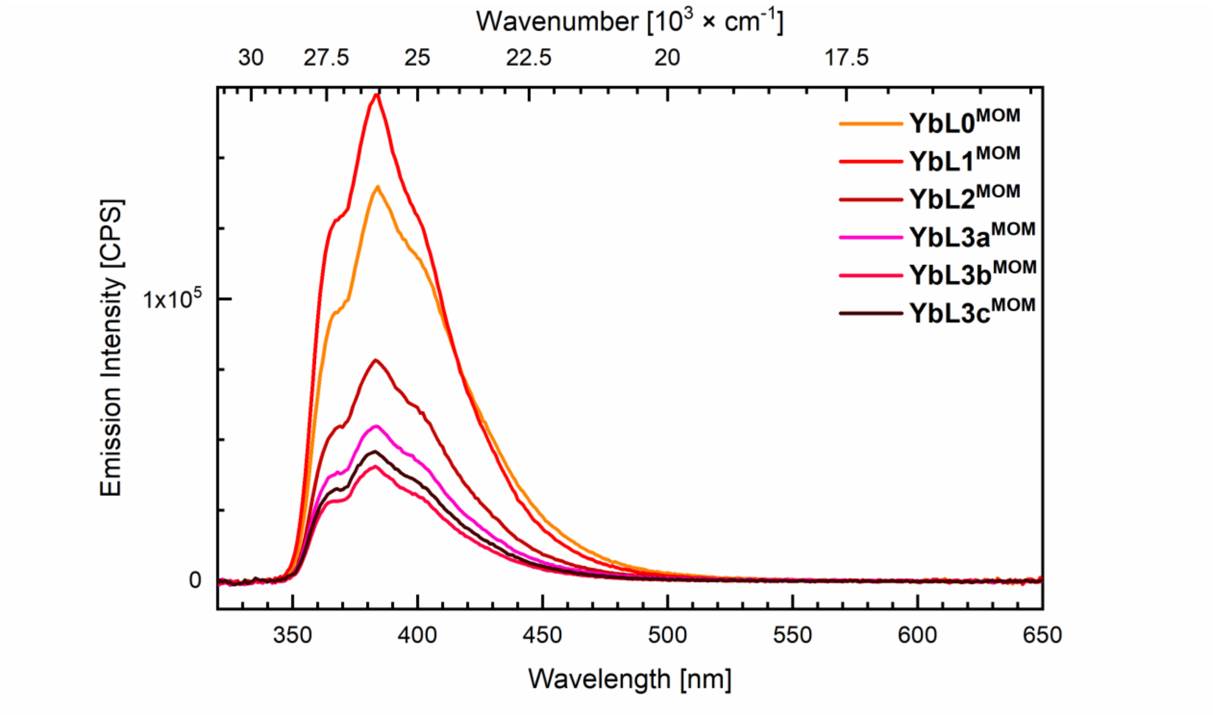

Figure S97. Steady-state fluorescence spectra of **YbL**$^{\mathbf{MOM}}$ indicating relative emission intensities under identical samples absorptions. [**YbL**$^{\mathbf{MOM}}$] = 10 µM in DMF, λ_{ex} = 335 nm, front slit: 2 nm, exit slit: 1.5 nm.

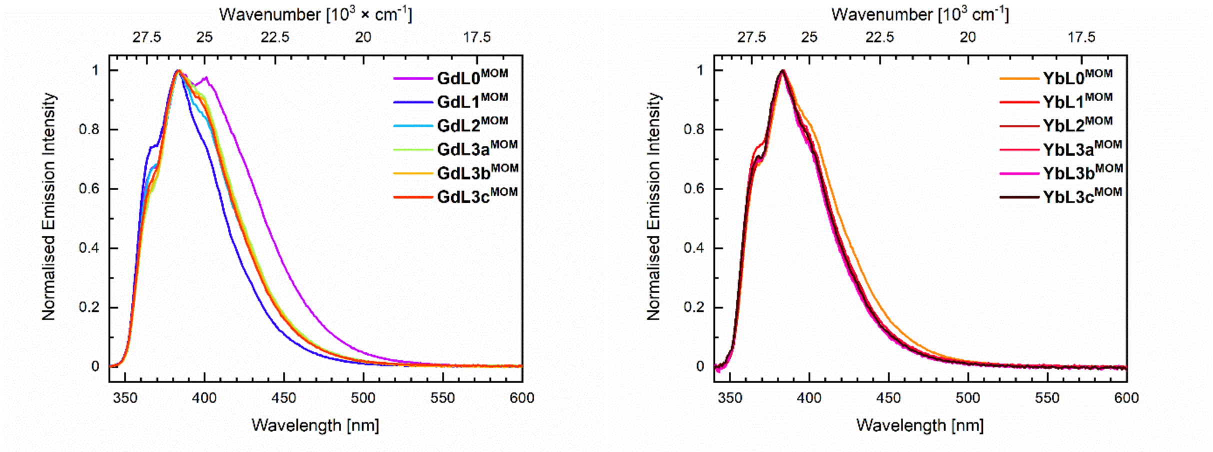

Figure S98. Normalized steady-state fluorescence spectra of **LnL**$^{\mathbf{MOM}}$ (left: Ln = Gd, right: Ln = Yb) under identical samples absorptions. [**LnL**$^{\mathbf{MOM}}$] = 10 µM in DMF, λ_{ex} = 335 nm, front slit: 2 nm, exit slit: 1.5 nm.

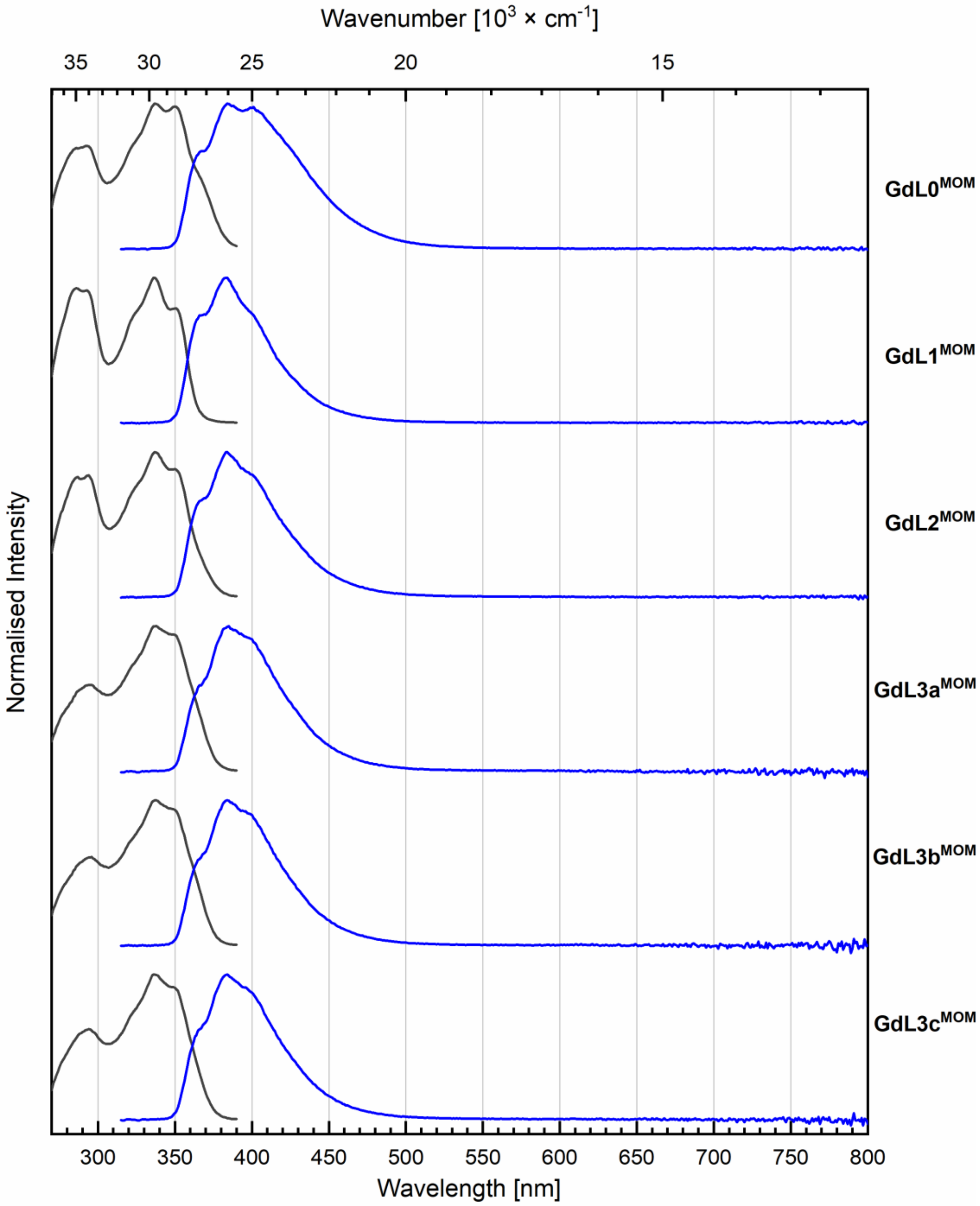

Figure S99. Ligand fluorescence excitation (dark grey, left, λ_{em} = 405 nm) and steady-state emission (blue, right, λ_{ex} = 335 nm) spectra of $\mathbf{GdL^{MOM}}$ in DMF. [$\mathbf{GdL^{MOM}}$] = 10 µM.

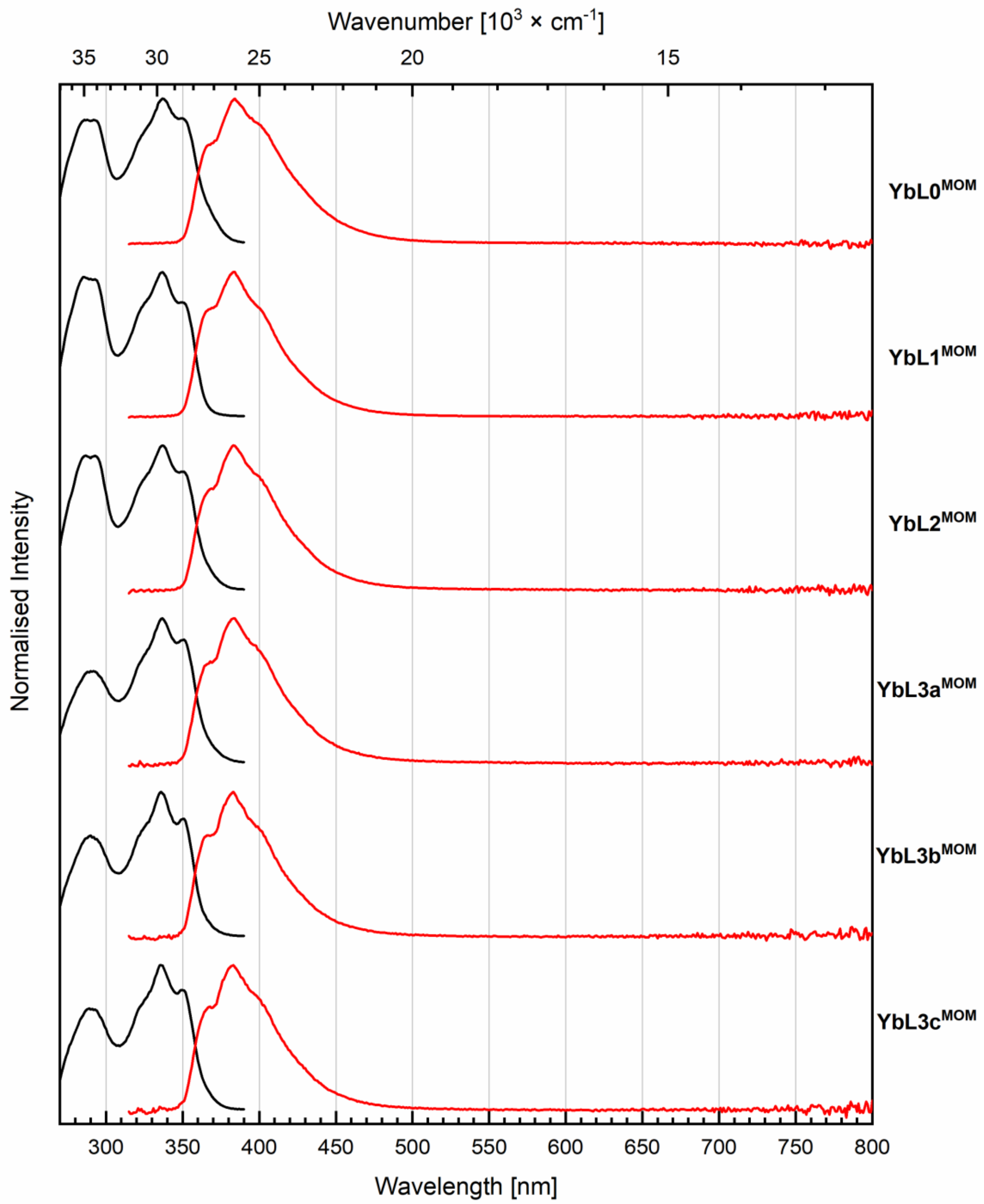

Figure S100. Ligand fluorescence excitation (black, left, λ_{em} = 405 nm) and steady-state emission (red, right, λ_{ex} = 335 nm) spectra of **YbL^{MOM}** in DMF. [**YbL^{MOM}**] = 10 µM.

Fluorescence decay fits and residuals

Table S18. Measured lifetimes ($\tau_{f,L}$) of **YbL^MOM** ligand fluorescence in H_2O (λ_{ex} = 341.5 nm, λ_{em} = 377 nm) (monoexponential reconvolution fit) from two or three independent measurements. The χ^2 value is the goodness of fit (χ^2 = 1 is the best fit).

Compound	$\tau_{f,L1}$ (ns)	χ^2 (1st)	$\tau_{f,L2}$ (ns)	χ^2 (2nd)	$\tau_{f,Lav}$ (ns)	Stand.Dev.
**YbL0^MOM**	0.342	1.107	0.344	1.415	0.343	0.001
**YbL1^MOM**	0.322	1.169	0.359	1.281	0.340	0.018
**YbL2^MOM**	0.255	1.352	0.251	1.318	0.253	0.002
**YbL3a^MOM**	0.267	1.613	0.257	1.636	0.262	0.005
**YbL3b^MOM**	0.225	1.396	0.228	1.346	0.226	0.001
**YbL3c^MOM**	0.265	1.376	0.295	1.440	0.280	0.015

Table S19. Measured lifetimes ($\tau_{f,L}$) of **YbL^MOM** ligand fluorescence in D_2O (λ_{ex} = 341.5 nm, λ_{em} = 377 nm) (monoexponential reconvolution fit) from two or three independent measurements. The χ^2 value is the goodness of fit (χ^2 = 1 is the best fit).

Compound	$\tau_{f,L1}$ (ns)	χ^2 (1st)	$\tau_{f,L2}$ (ns)	χ^2 (2nd)	$\tau_{f,L3}$ (ns)	χ^2 (3rd)	$\tau_{f,Lav}$ (ns)	Stand.Dev.
**YbL0^MOM**	0.312	1.232	0.338	1.007	0.289	1.272	0.313	0.020
**YbL1^MOM**	0.299	1.234	0.309	1.015	-	-	0.304	0.005
**YbL2^MOM**	0.233	1.283	0.255	1.042	-	-	0.244	0.011
**YbL3a^MOM**	0.224	1.229	0.258	1.300	0.171	1.374	0.218	0.036
**YbL3b^MOM**	0.220	1.164	0.200	1.146	-	-	0.210	0.010
**YbL3c^MOM**	0.266	1.281	0.263	1.134	-	-	0.264	0.002

Table S20. Measured lifetimes ($\tau_{f,L}$) of **LuL^MOM** ligand fluorescence in H_2O (λ_{ex} = 341.5 nm, λ_{em} = 377 nm) and Rose Bengal emission in MeOH (λ_{em} = 577 nm) (monoexponential reconvolution fit) from two independent measurements. The χ^2 value is the goodness of fit (χ^2 = 1 is the best fit).

Compound	$\tau_{f,L1}$ (ns)	χ^2 (1st)	$\tau_{f,L2}$ (ns)	χ^2 (2nd)	$\tau_{f,Lav}$ (ns)	Stand.Dev.
**Rose Bengal** [a]	0.513	1.287	-	-	-	-
**LuL0^MOM**	0.472	1.085	0.463	1.263	0.468	0.005
**LuL1^MOM**	0.477	1.156	0.458	1.257	0.467	0.010
**LuL2^MOM**	0.476	1.180	0.442	1.328	0.459	0.017
**LuL3a^MOM**	0.452	1.175	0.442	1.377	0.447	0.005
**LuL3b^MOM**	0.449	1.087	0.445	1.440	0.447	0.002
**LuL3c^MOM**	0.430	1.326	-	-	0.430	-

[a] The literature value for Rose Bengal in MeOH: $\tau_{f,L}$ = 0.543±0.009 ns at λ_{ex} = 575 nm.[24]

Table S21. Measured lifetimes ($\tau_{f,L}$) of **LuL**$^{\mathbf{MOM}}$ ligand fluorescence in D_2O (λ_{ex} = 341.5 nm, λ_{em} = 377 nm) (monoexponential reconvolution fit) from two independent measurements. The χ^2 value is the goodness of fit (χ^2 = 1 is the best fit).

Compound	$\tau_{f,L1}$ (ns)	χ^2 (1st)	$\tau_{f,L2}$ (ns)	χ^2 (2nd)	$\tau_{f,Lav}$ (ns)	Stand.Dev.
**LuL0**$^{\mathbf{MOM}}$	0.473	1.042	0.445	1.278	0.459	0.014
**LuL1**$^{\mathbf{MOM}}$	0.474	1.138	0.460	1.358	0.467	0.007
**LuL2**$^{\mathbf{MOM}}$	0.453	0.995	0.442	1.215	0.447	0.005
**LuL3a**$^{\mathbf{MOM}}$	0.456	1.024	0.436	1.207	0.446	0.010
**LuL3b**$^{\mathbf{MOM}}$	0.436	1.040	0.423	1.353	0.429	0.006
**LuL3c**$^{\mathbf{MOM}}$	0.417	1.232	-	-	0.417	-

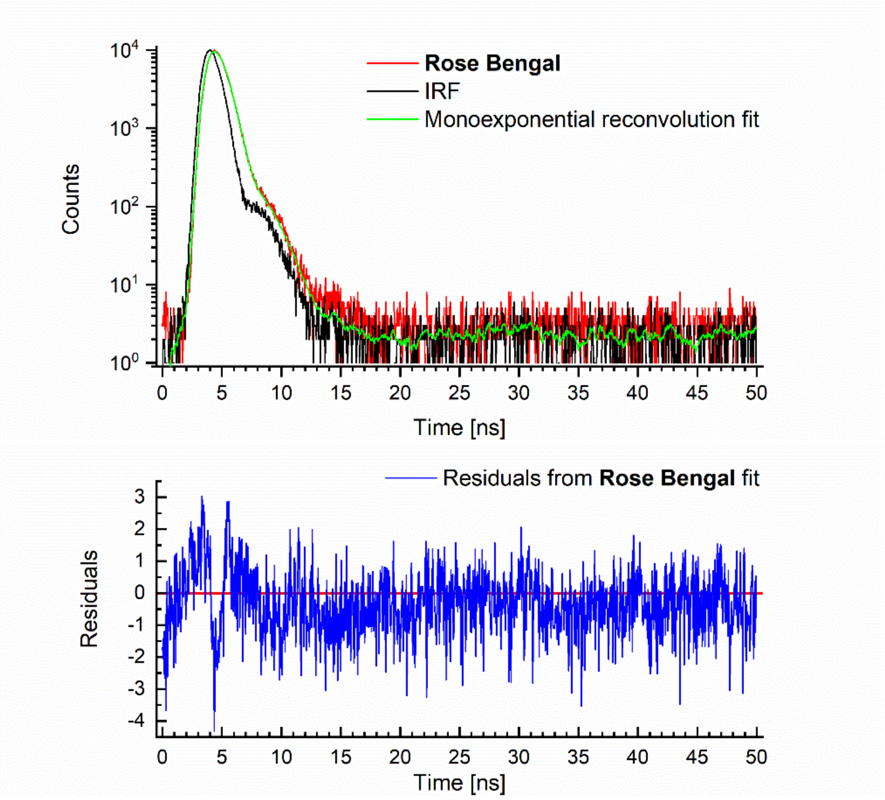

Figure S101. The fluorescence decay and reconvolution fit of Rose Bengal in MeOH (top) and residuals of the fit (bottom).

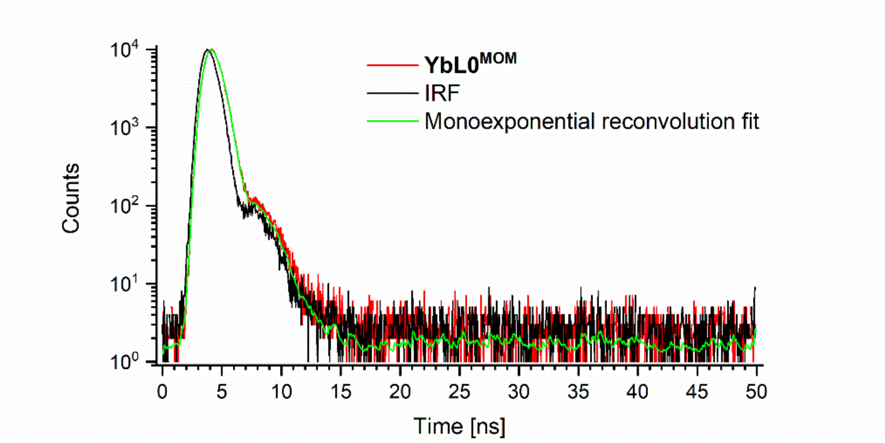

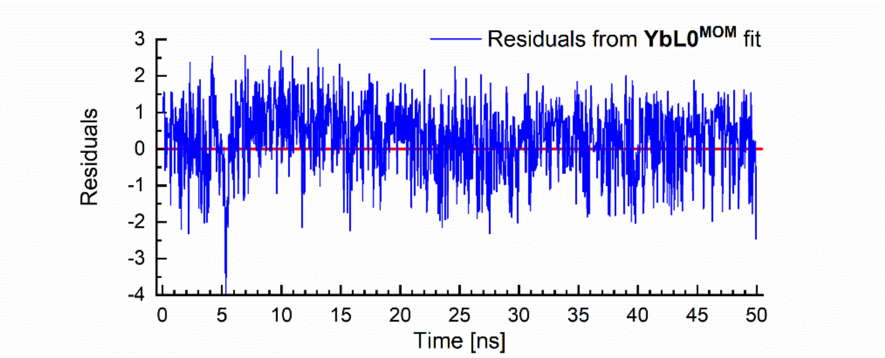

Figure S102. The fluorescence decay and reconvolution fit of **YbL0**$^{\mathbf{MOM}}$ in H_2O (top) and residuals of the fit (bottom).

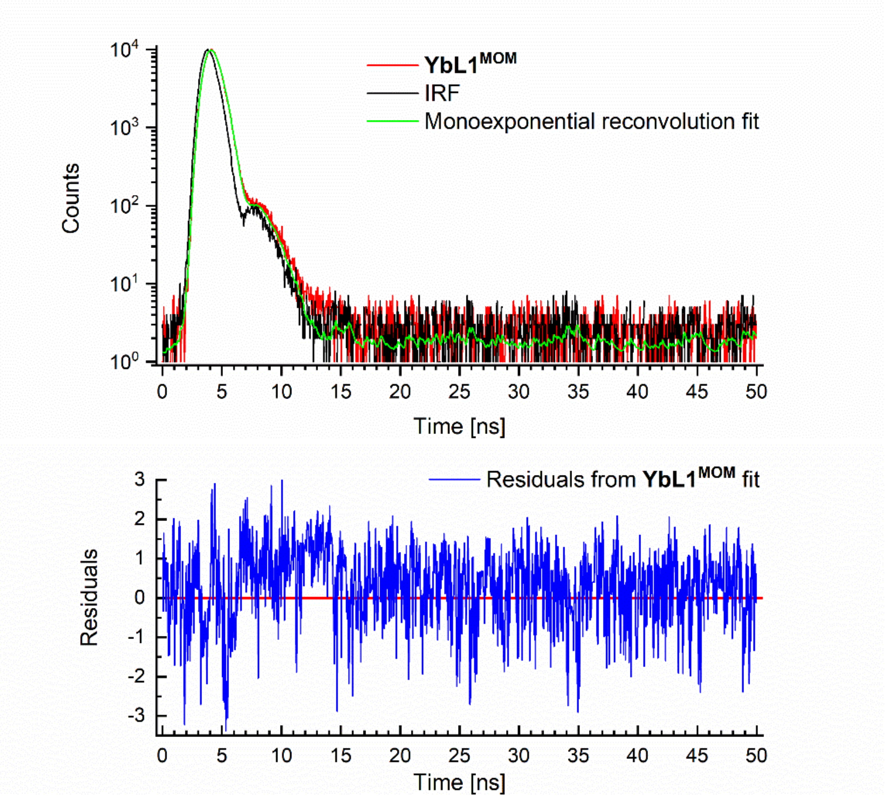

Figure S103. The fluorescence decay and reconvolution fit of **YbL1**$^{\mathbf{MOM}}$ in H_2O (top) and residuals of the fit (bottom).

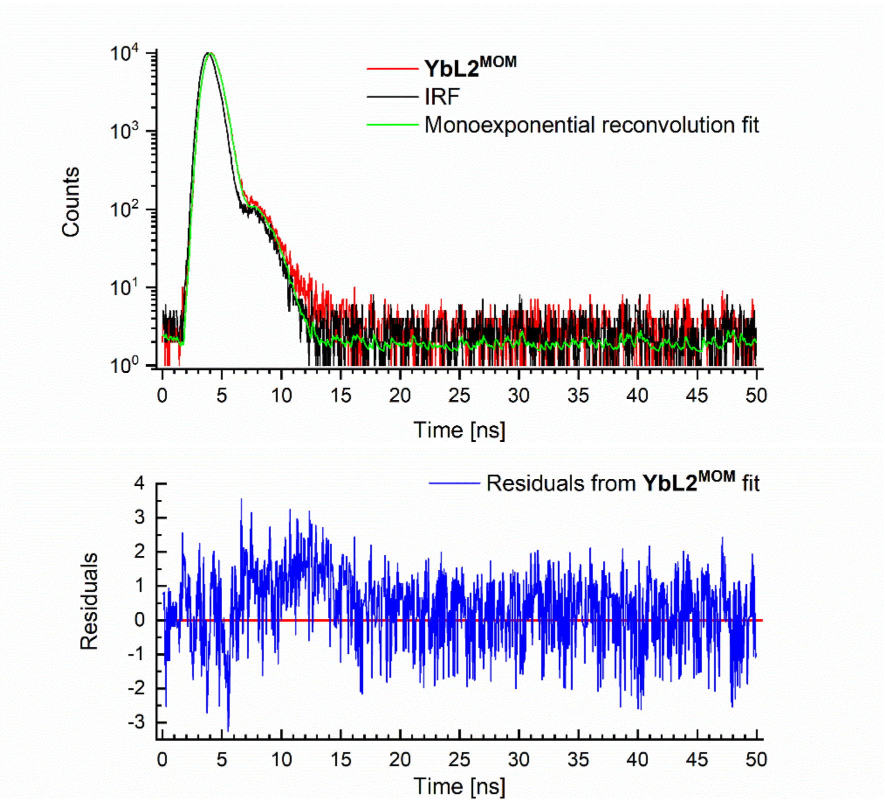

Figure S104. The fluorescence decay and reconvolution fit of **YbL2^{MOM}** in H_2O (top) and residuals of the fit (bottom).

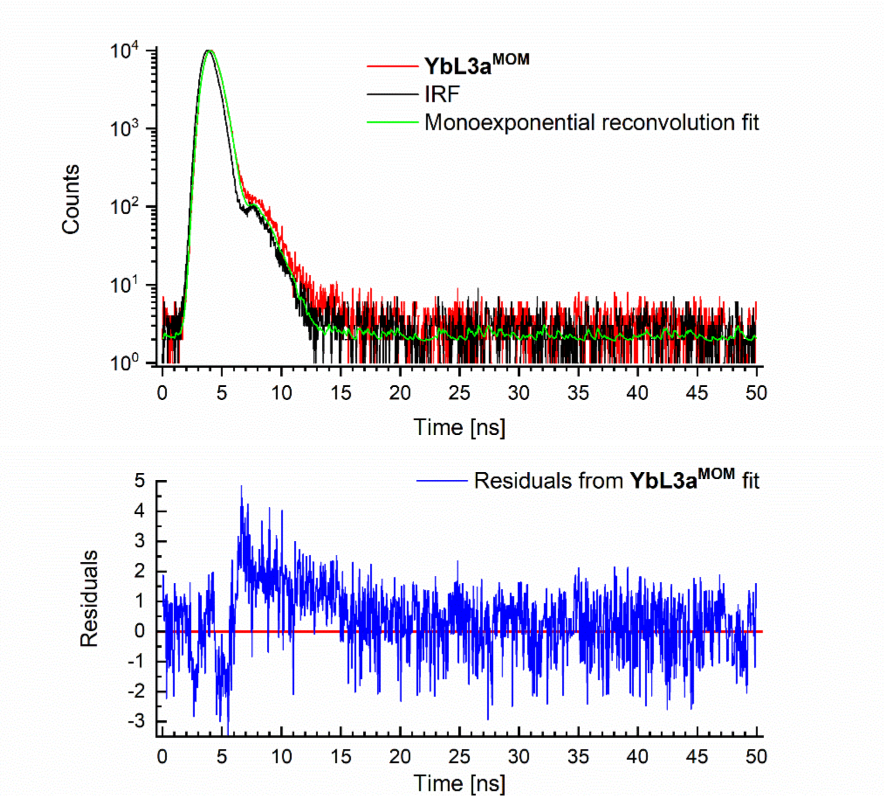

Figure S105. The fluorescence decay and reconvolution fit of **YbL3a^{MOM}** in H_2O (top) and residuals of the fit (bottom).

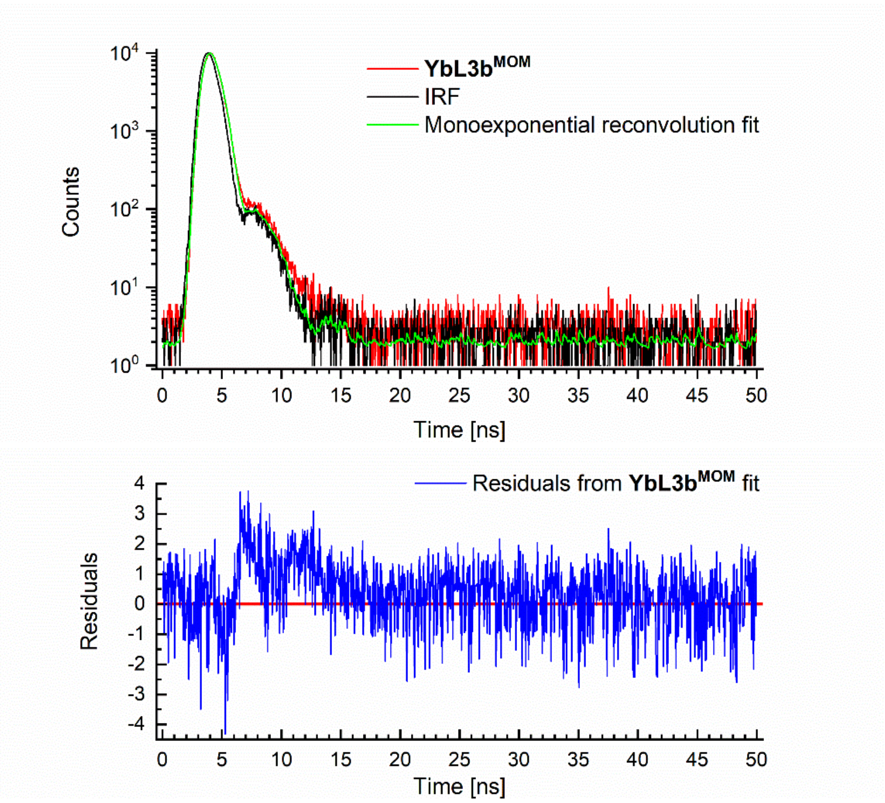

Figure S106. The fluorescence decay and reconvolution fit of **YbL3b**^{MOM} in H_2O (top) and residuals of the fit (bottom).

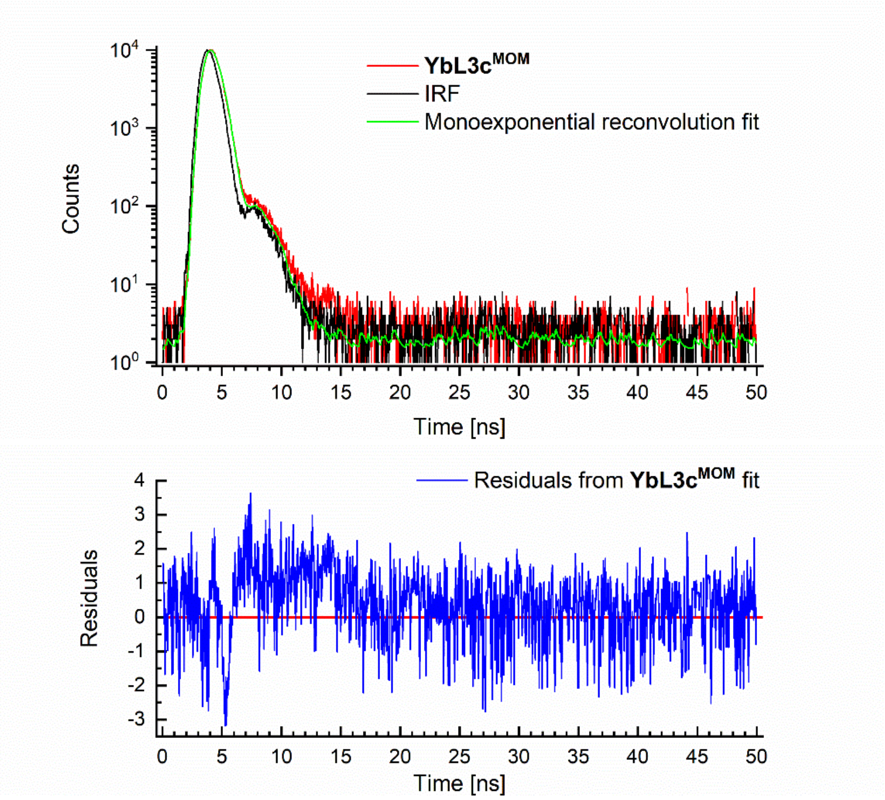

Figure S107. The fluorescence decay and reconvolution fit of **YbL3c**^{MOM} in H_2O (top) and residuals of the fit (bottom).

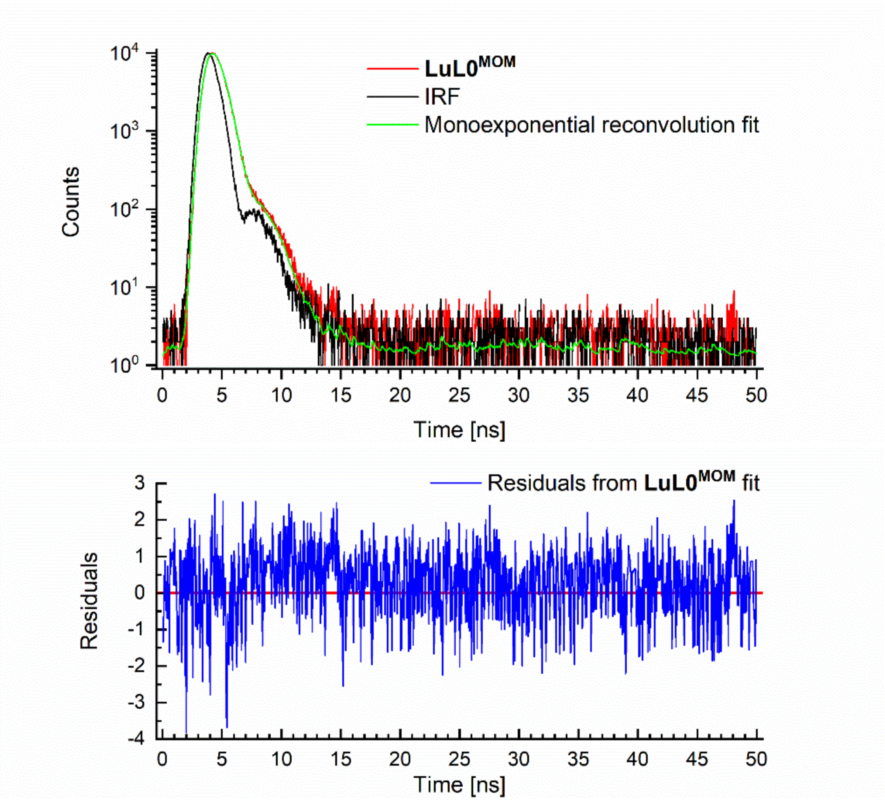

Figure S108. The fluorescence decay and reconvolution fit of **LuL0^{MOM}** in H_2O (top) and residuals of the fit (bottom).

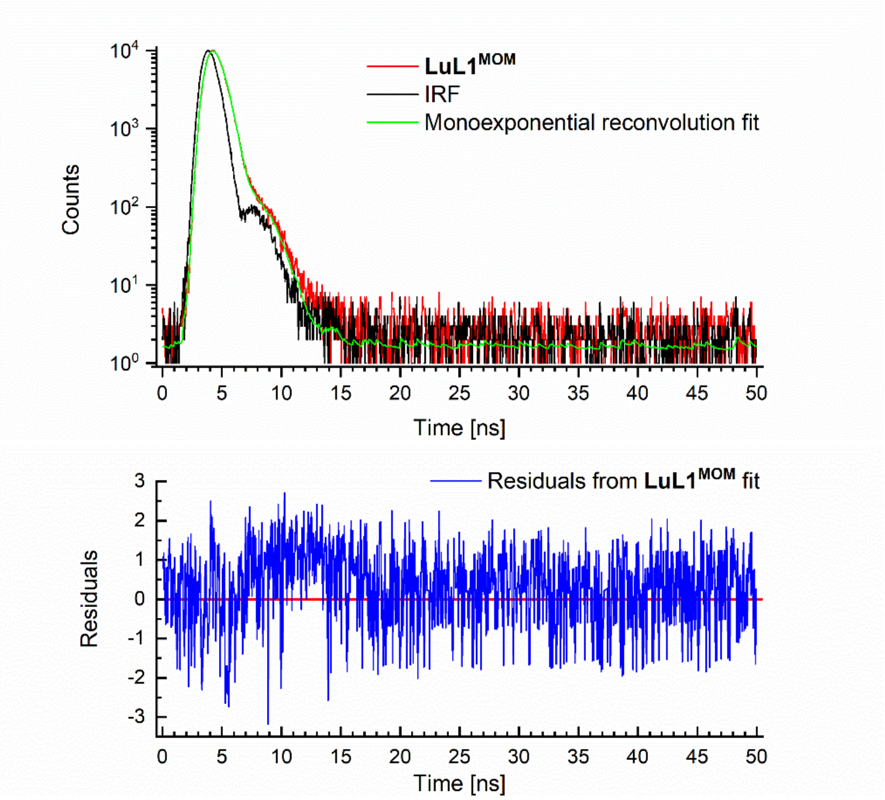

Figure S109. The fluorescence decay and reconvolution fit of **LuL1^{MOM}** in H_2O (top) and residuals of the fit (bottom).

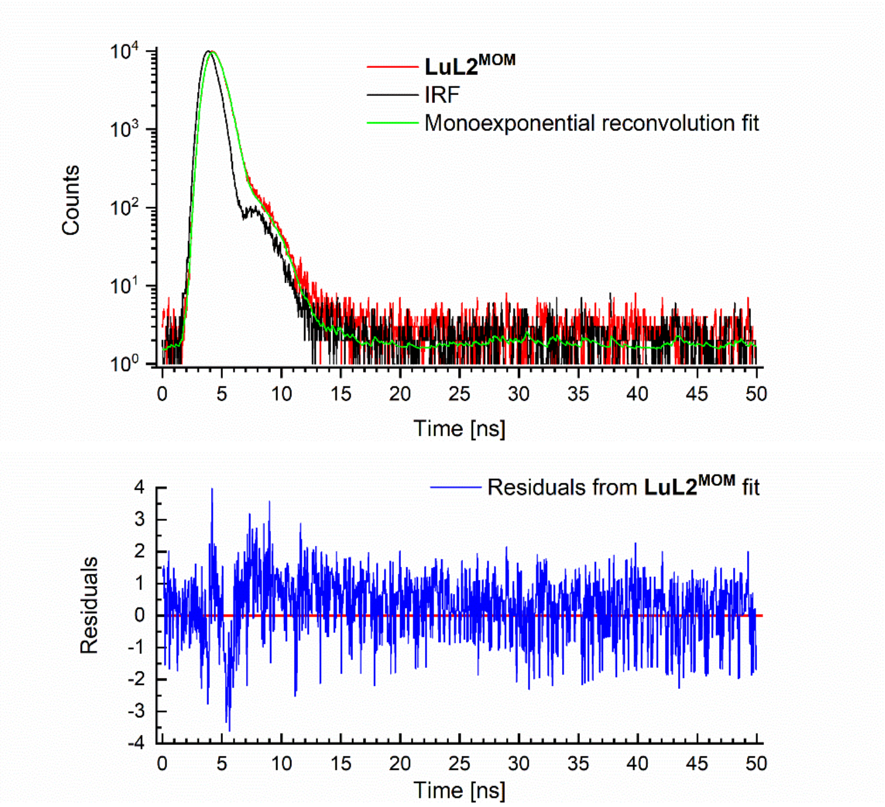

Figure S110. The fluorescence decay and reconvolution fit of **LuL2**$^{\mathbf{MOM}}$ in H_2O (top) and residuals of the fit (bottom).

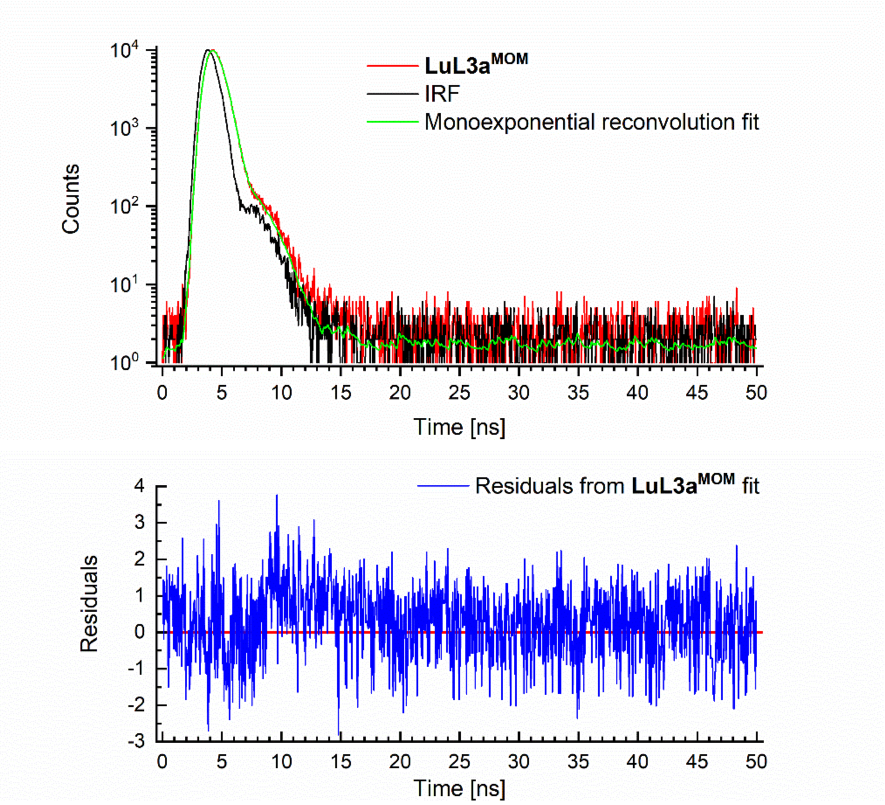

Figure S111. The fluorescence decay and reconvolution fit of **LuL3a**$^{\mathbf{MOM}}$ in H_2O (top) and residuals of the fit (bottom).

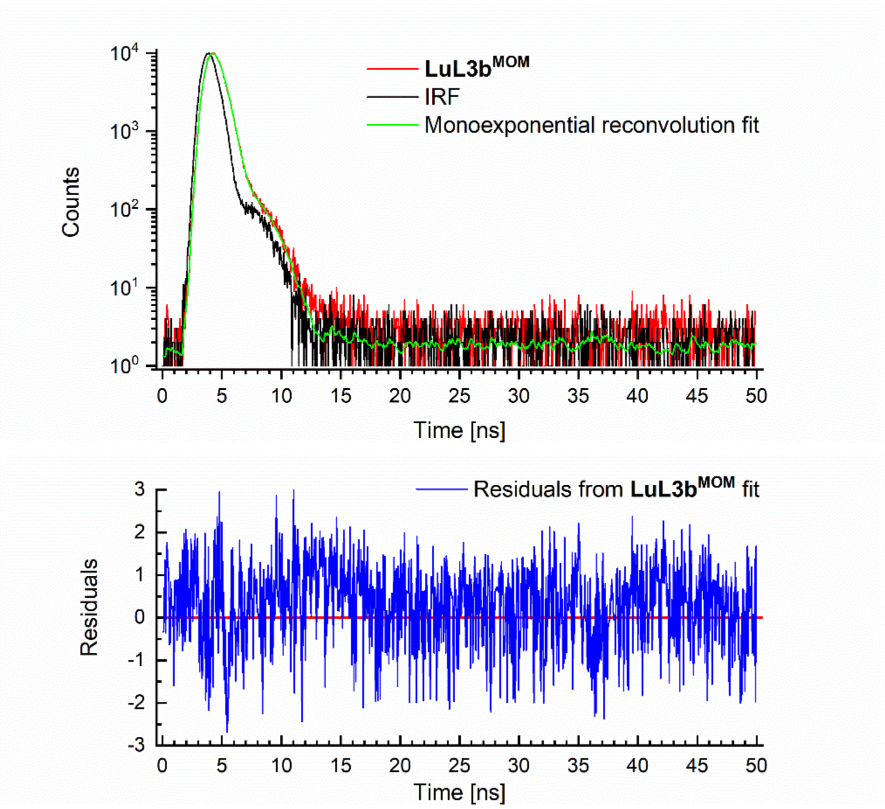

Figure S112. The fluorescence decay and reconvolution fit of **LuL3b^{MOM}** in H_2O (top) and residuals of the fit (bottom).

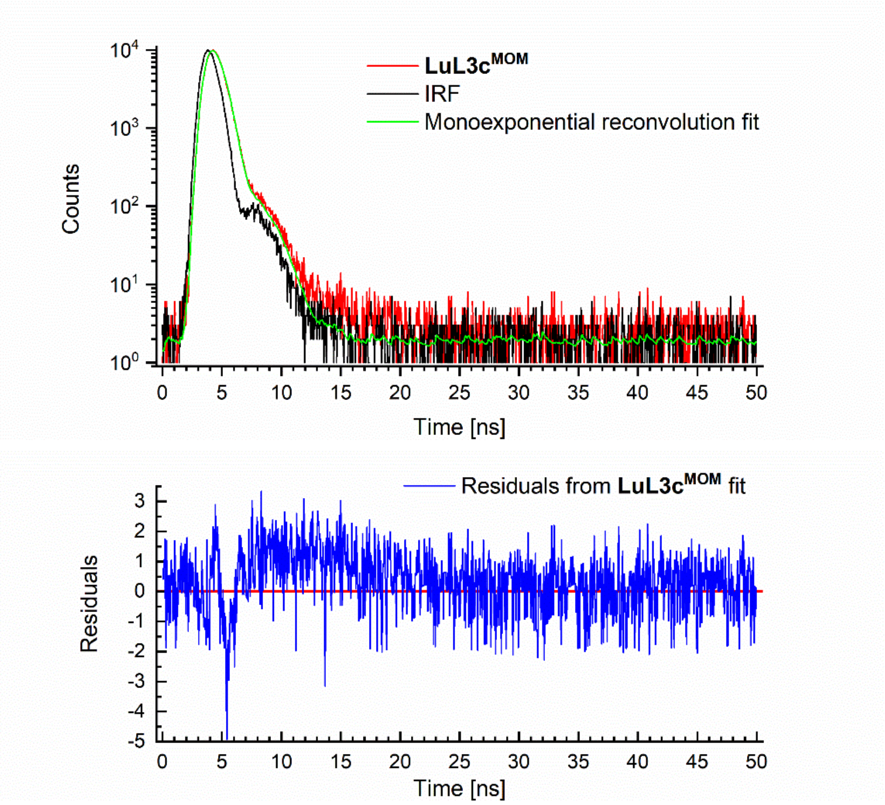

Figure S113. The fluorescence decay and reconvolution fit of **LuL3c^{MOM}** in H_2O (top) and residuals of the fit (bottom).

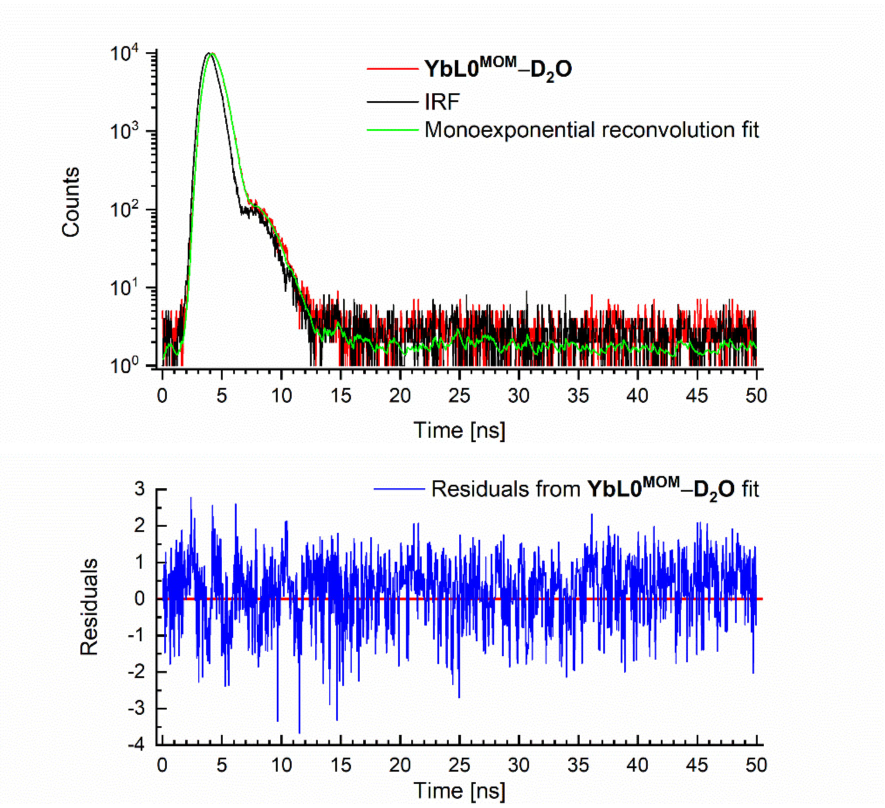

Figure S114. The fluorescence decay and reconvolution fit of **YbL0^{MOM}** in D_2O (top) and residuals of the fit (bottom).

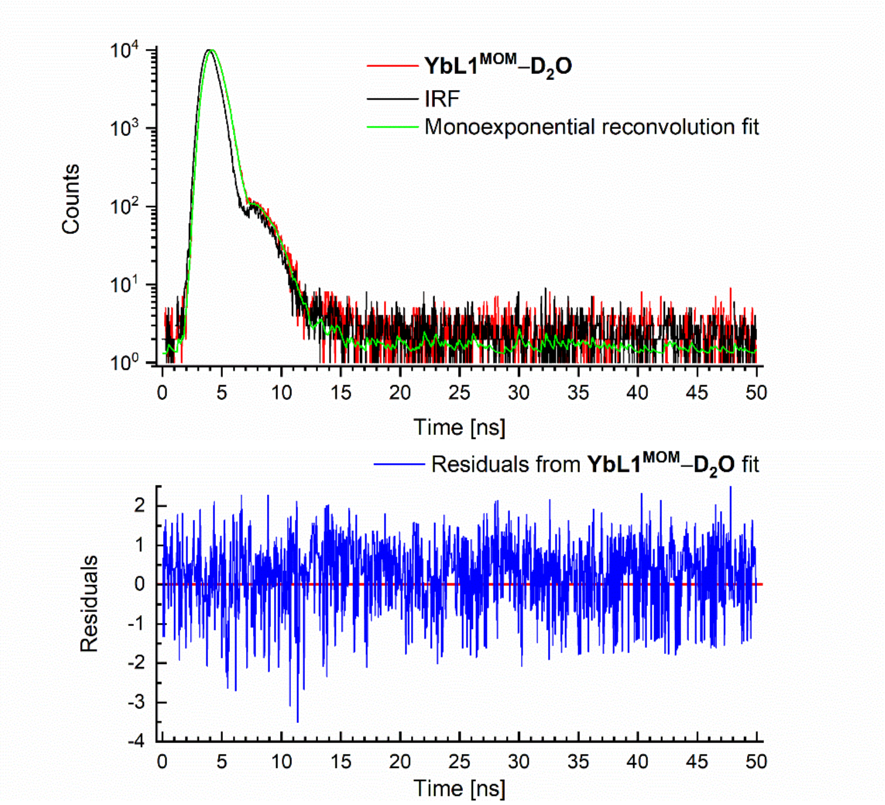

Figure S115. The fluorescence decay and reconvolution fit of **YbL1^{MOM}** in D_2O (top) and residuals of the fit (bottom).

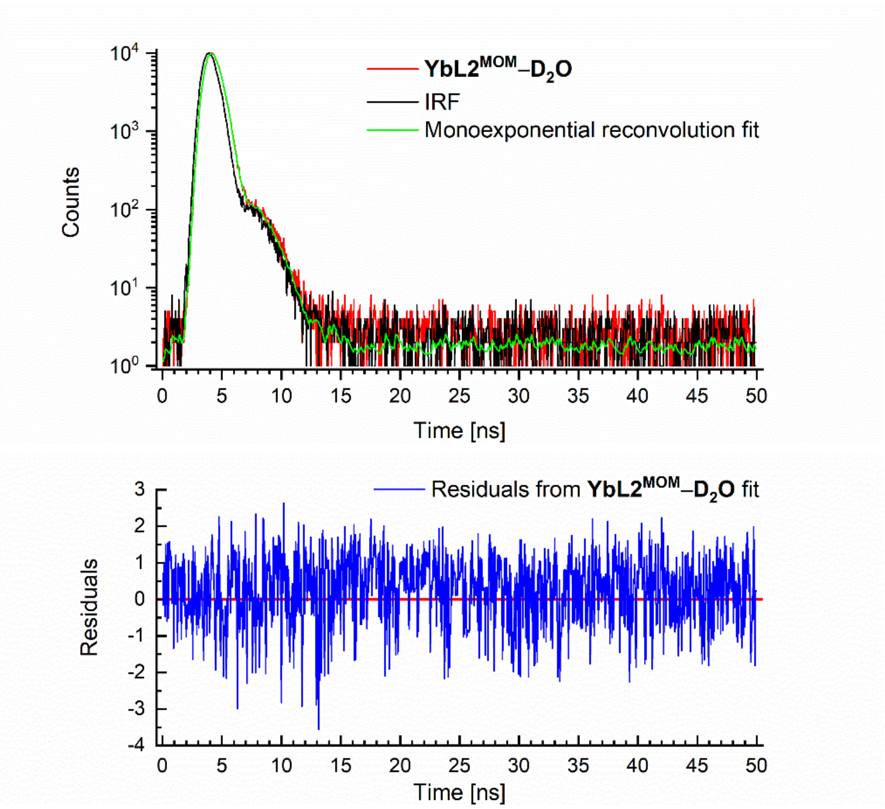

Figure S116. The fluorescence decay and reconvolution fit of **YbL2**$^{\mathbf{MOM}}$ in D_2O (top) and residuals of the fit (bottom).

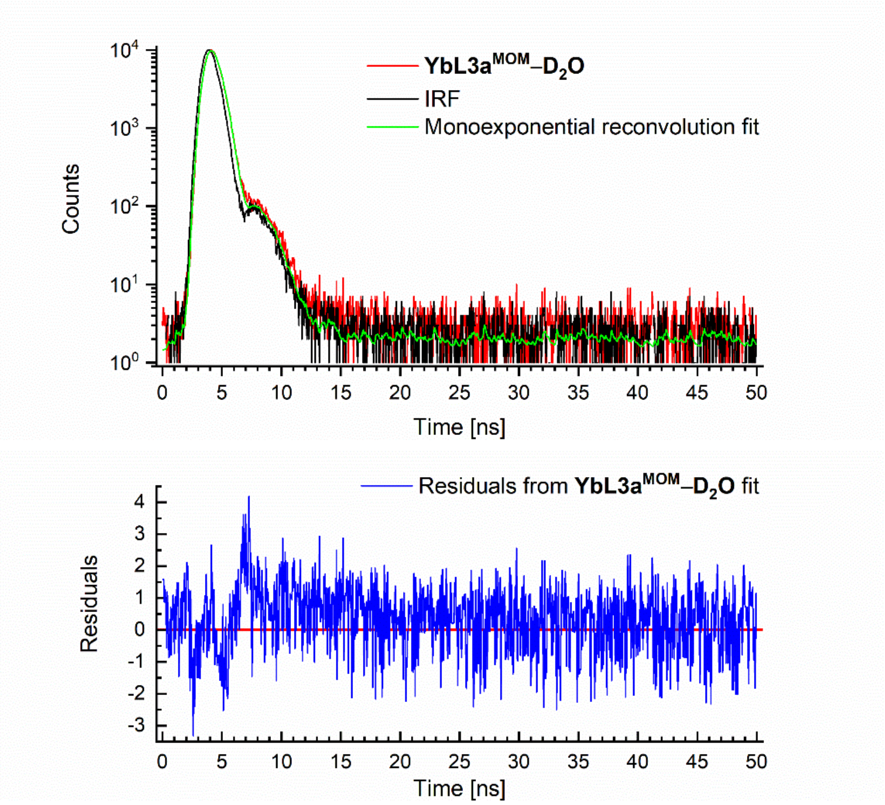

Figure S117. The fluorescence decay and reconvolution fit of **YbL3a**$^{\mathbf{MOM}}$ in D_2O (top) and residuals of the fit (bottom).

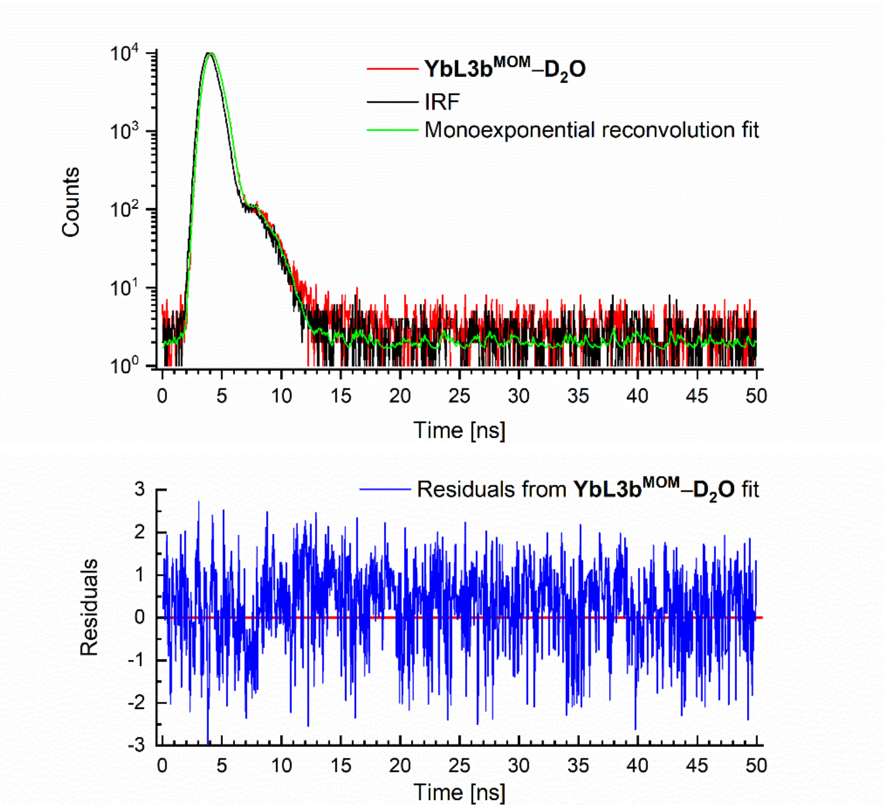

Figure S118. The fluorescence decay and reconvolution fit of **YbL3b^{MOM}** in D_2O (top) and residuals of the fit (bottom).

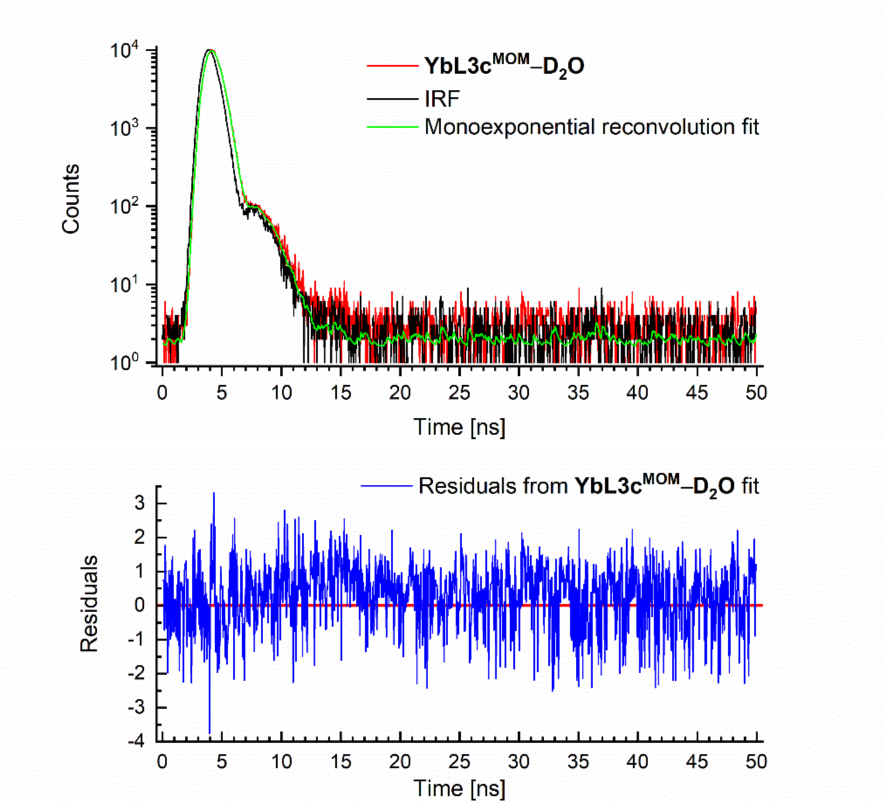

Figure S119. The fluorescence decay and reconvolution fit of **YbL3c^{MOM}** in D_2O (top) and residuals of the fit (bottom).

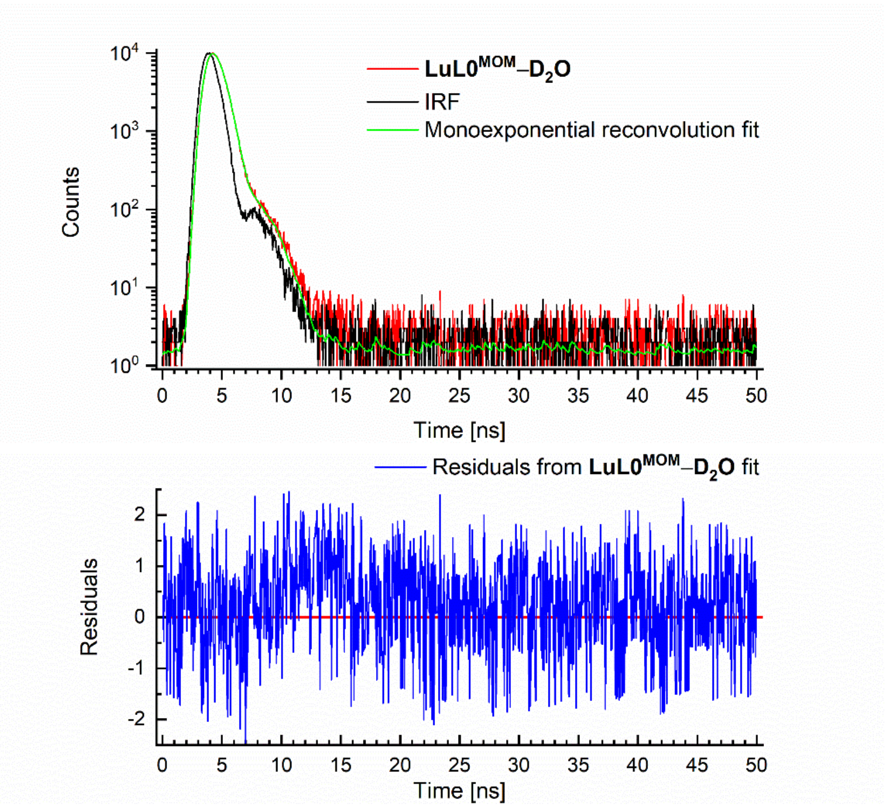

Figure S120. The fluorescence decay and reconvolution fit of **LuL0^{MOM}** in D_2O (top) and residuals of the fit (bottom).

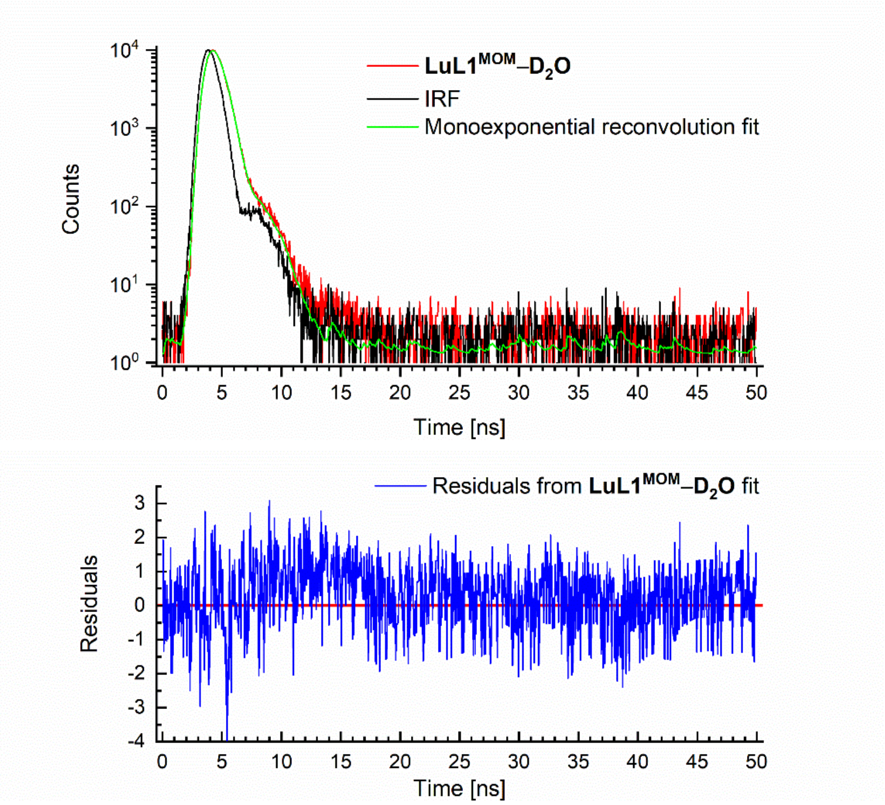

Figure S121. The fluorescence decay and reconvolution fit of **LuL1^{MOM}** in D_2O (top) and residuals of the fit (bottom).

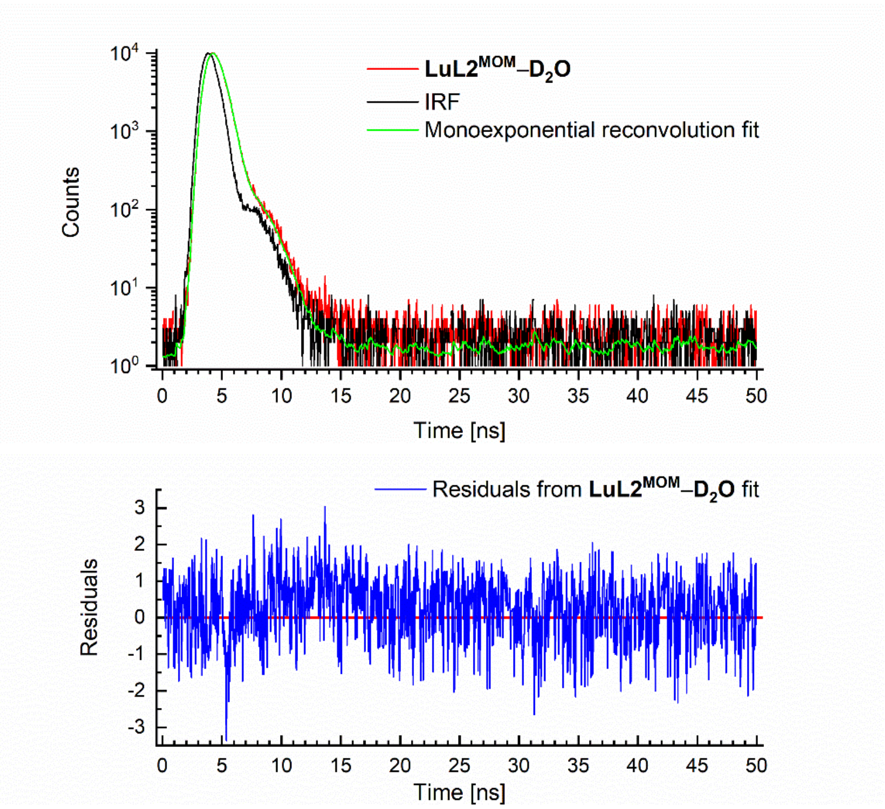

Figure S122. The fluorescence decay and reconvolution fit of **LuL2^{MOM}** in D_2O (top) and residuals of the fit (bottom).

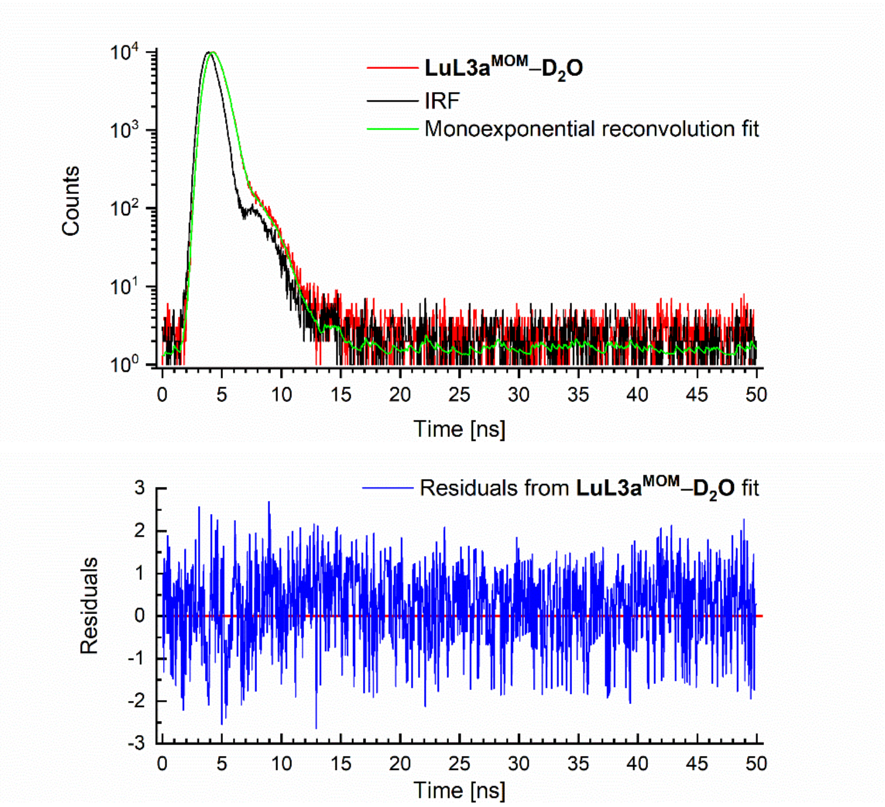

Figure S123. The fluorescence decay and reconvolution fit of **LuL3a^{MOM}** in D_2O (top) and residuals of the fit (bottom).

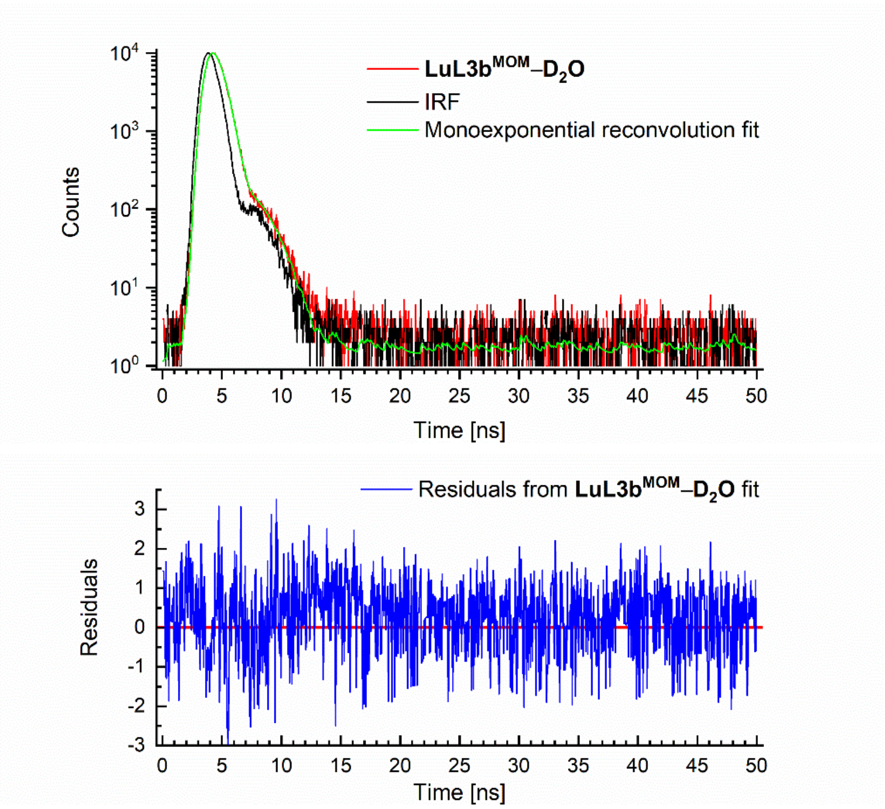

Figure S124. The fluorescence decay and reconvolution fit of **LuL3b**$^{\mathbf{MOM}}$ in D_2O (top) and residuals of the fit (bottom).

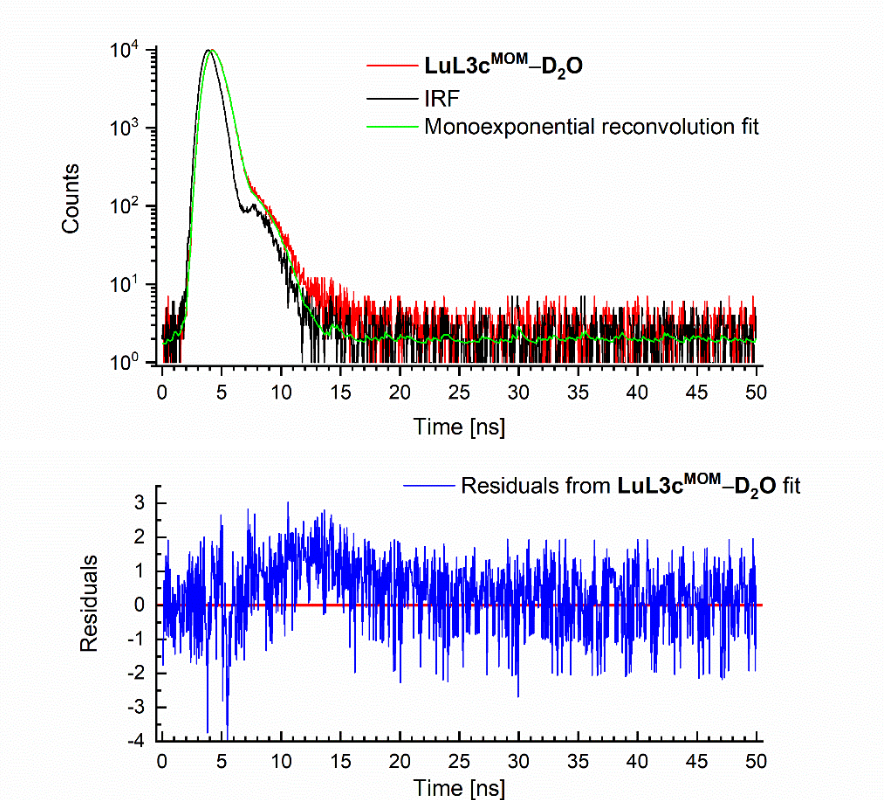

Figure S125. The fluorescence decay and reconvolution fit of **LuL3c**$^{\mathbf{MOM}}$ in D_2O (top) and residuals of the fit (bottom).

Photostability experiments

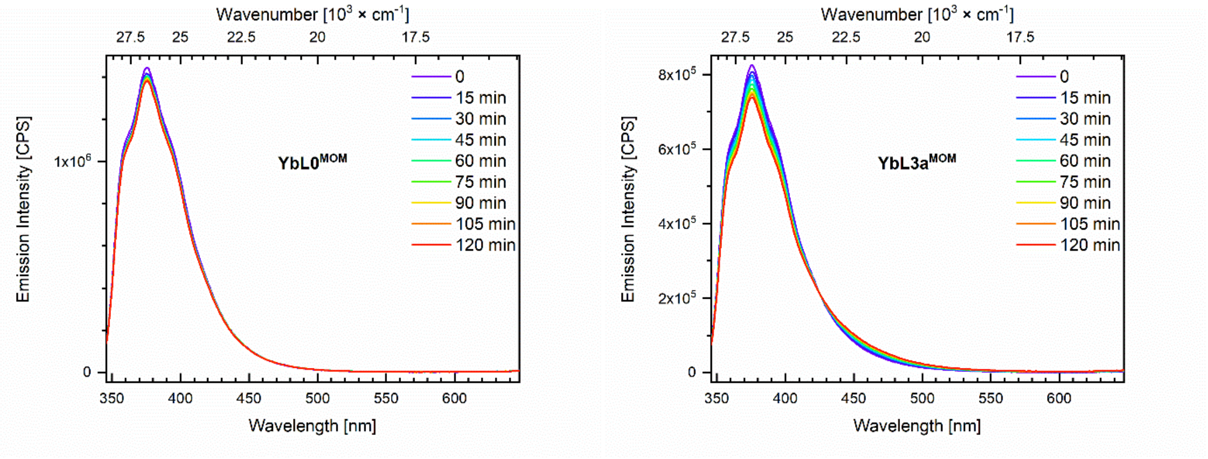

Figure S126. Steady-state fluorescence spectra of aerated **YbL0,3a**$^{\mathbf{MOM}}$ upon continuous light irradiation under identical samples absorptions (A = 0.10). [**YbL0,3a**$^{\mathbf{MOM}}$] = 10 µM in 10 mM PIPES-buffered solutions, pH = 6.5; λ_{ex} = 331 nm, front slit: 3 nm, exit slit: 2.5 nm.

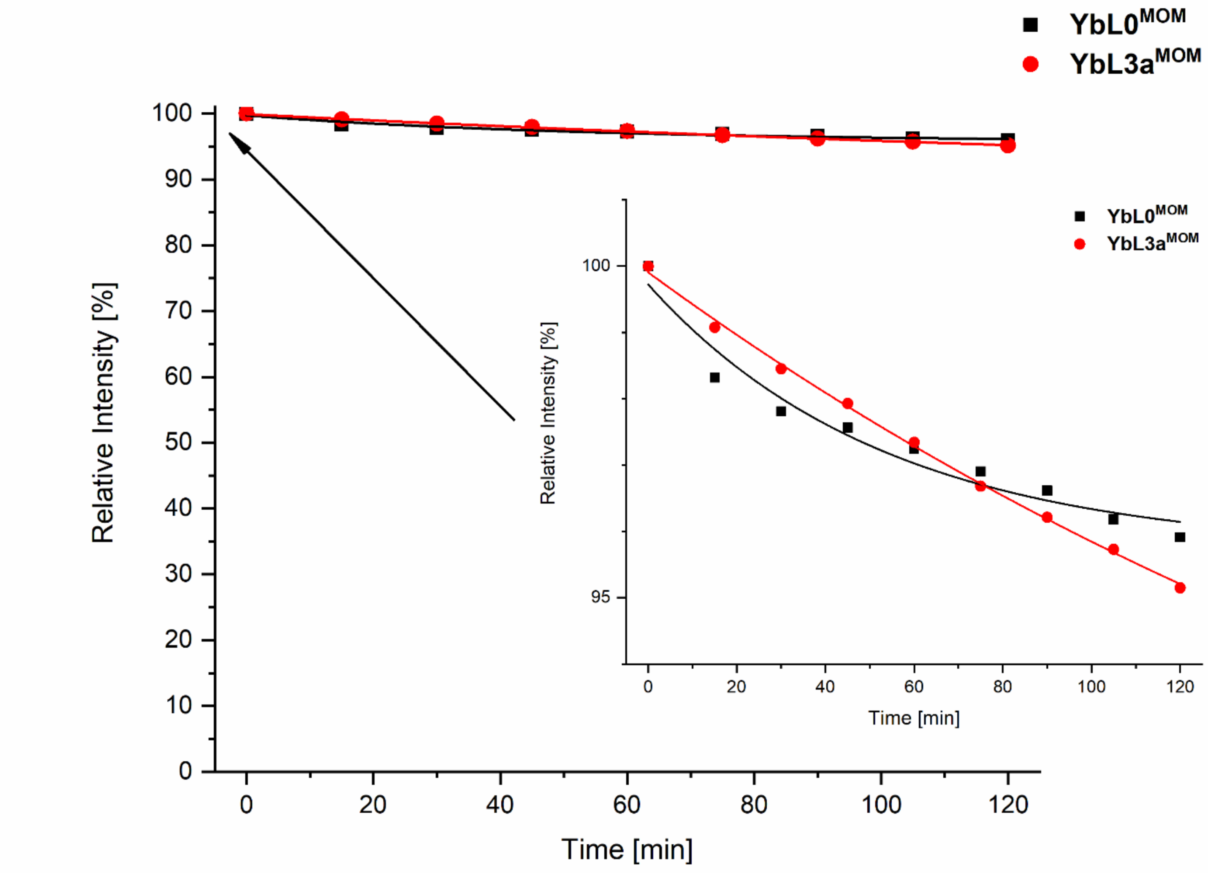

Figure S127. Relative emission intensity of aerated **YbL0,3a**$^{\mathbf{MOM}}$ upon continuous light irradiation under identical sample absorptions. After blank signal subtraction each spectrum was integrated (346–647 nm), and the integrated intensity was divided by that at t_0 and multiplied by 100%. The lines next to the data values are only to guide the eye.

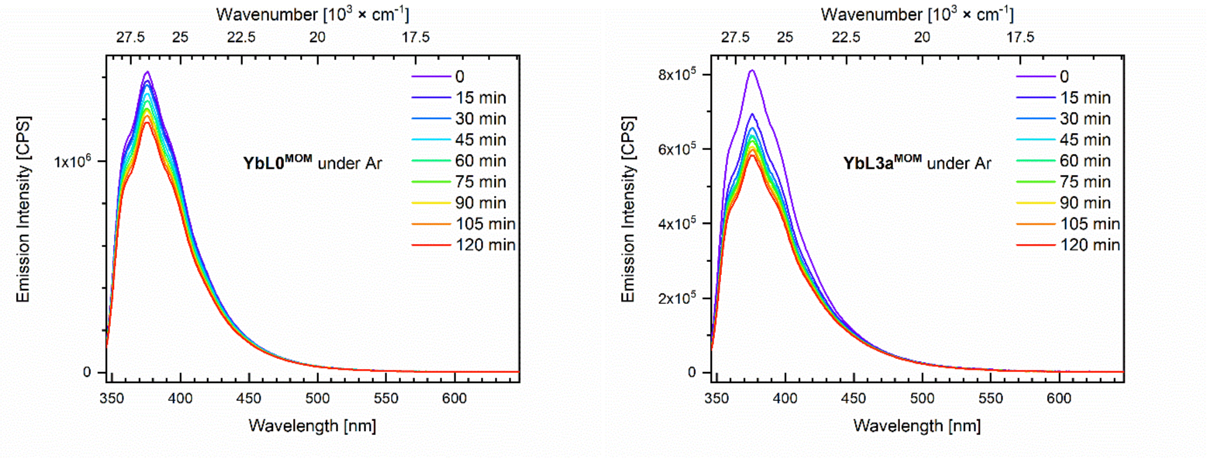

Figure S128. Steady-state fluorescence spectra of **YbL0,3a**^{MOM} under Ar upon continuous light irradiation under identical samples absorptions (A = 0.10). [**YbL0,3a**^{MOM}] = 10 µM in 10 mM PIPES-buffered solutions, pH = 6.5; λ_{ex} = 331 nm, front slit: 3 nm, exit slit: 2.5 nm.

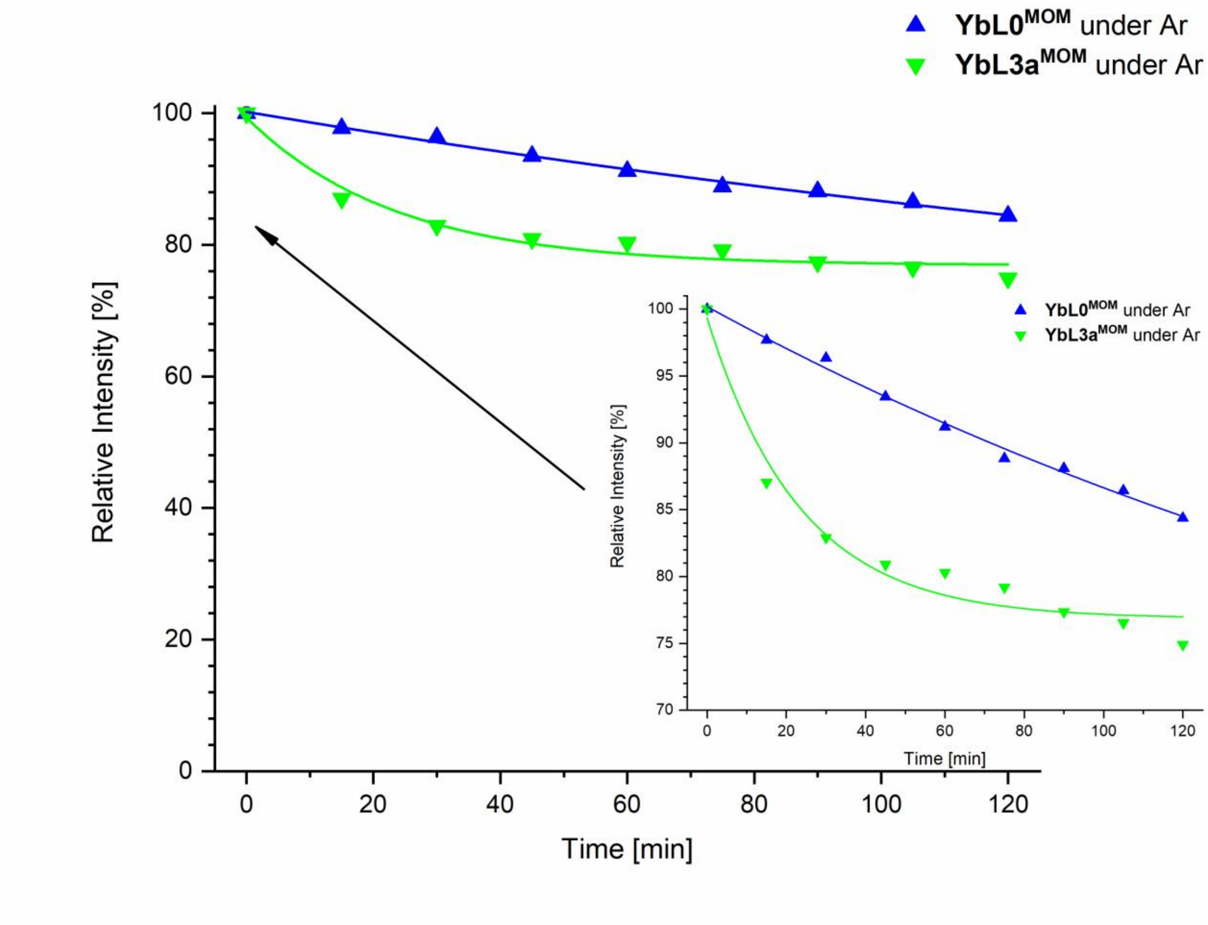

Figure S129. Relative emission intensity of **YbL0,3a**^{MOM} under Ar upon continuous light irradiation under identical sample absorptions. After blank signal subtraction each spectrum was integrated (346–647 nm), and the integrated intensity was divided by that at t_0 and multiplied by 100%. The lines next to the data values are only to guide the eye.

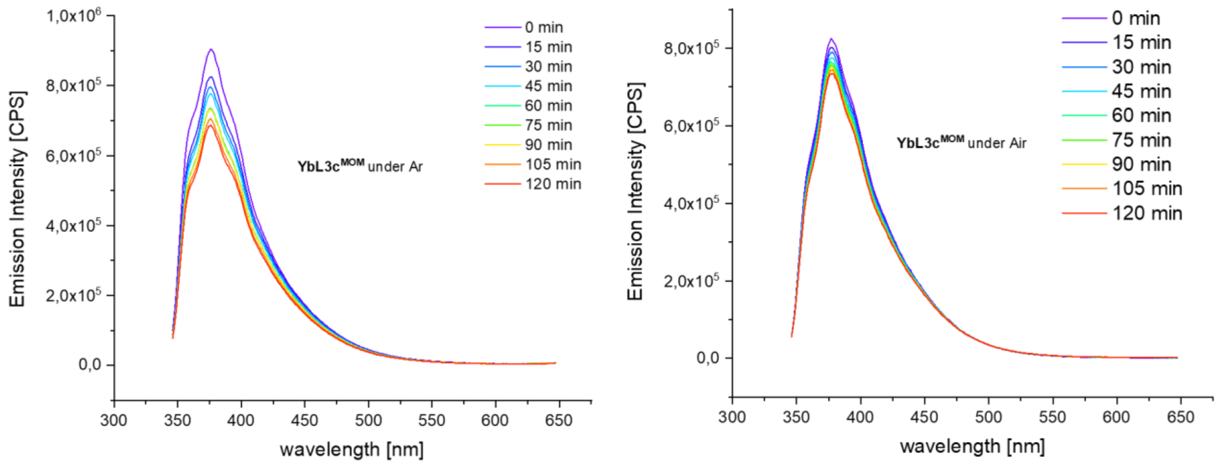

Figure S130. Steady-state fluorescence spectra of **YbL3c^{MOM}** under Ar upon continuous light irradiation under identical samples absorptions (A = 0.10). [**YbL3c^{MOM}**] = 10 µM in 10 mM PIPES-buffered solutions, pH = 6.5; λ_{ex} = 331 nm, front slit: 3 nm, exit slit: 2.5 nm.

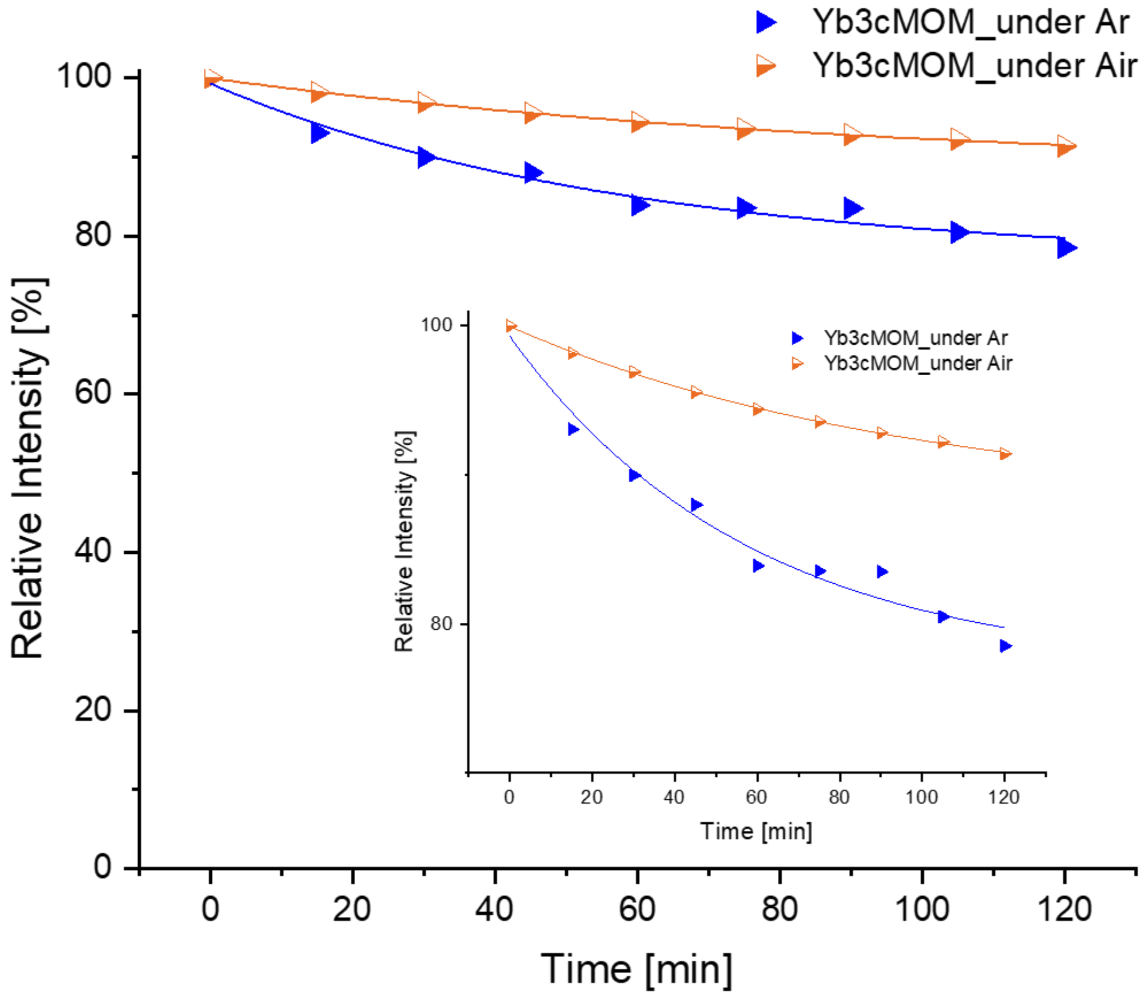

Figure S131. Relative emission intensity of **YbL3c^{MOM}** under Ar upon continuous light irradiation under identical sample absorptions. After blank signal subtraction each spectrum was integrated (346–647 nm), and the integrated intensity was divided by that at t_0 and multiplied by 100%.

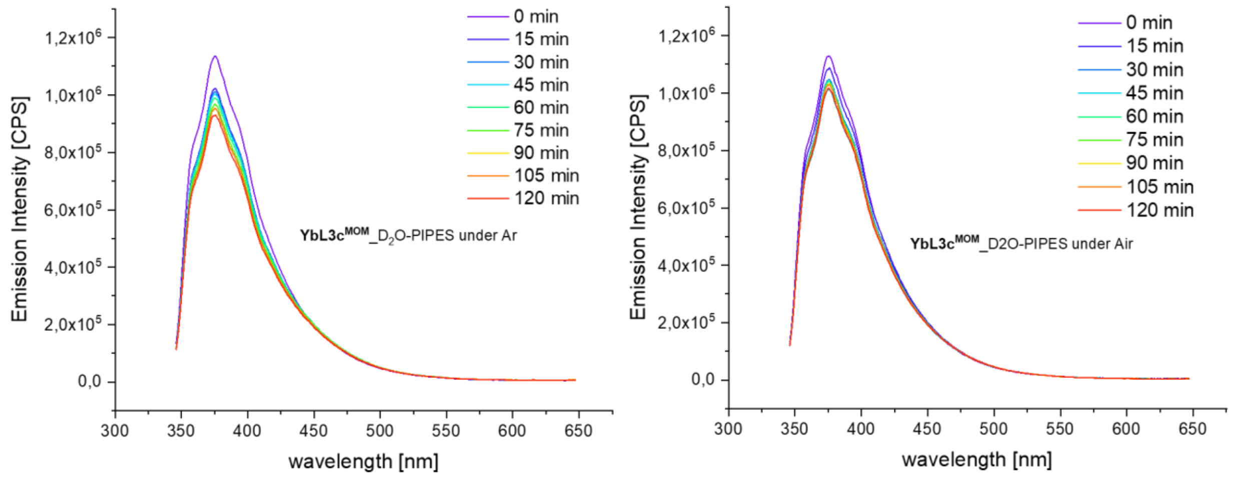

Figure S132. Steady-state fluorescence spectra of **YbL3c^{MOM}** under Ar upon continuous light irradiation under identical samples absorptions ($A = 0.10$). [**YbL3c^{MOM}**] = 10 µM in 10 mM D2O PIPES-buffered solutions, pH = 6.5; λ_{ex} = 331 nm, front slit: 3 nm, exit slit: 2.5 nm.

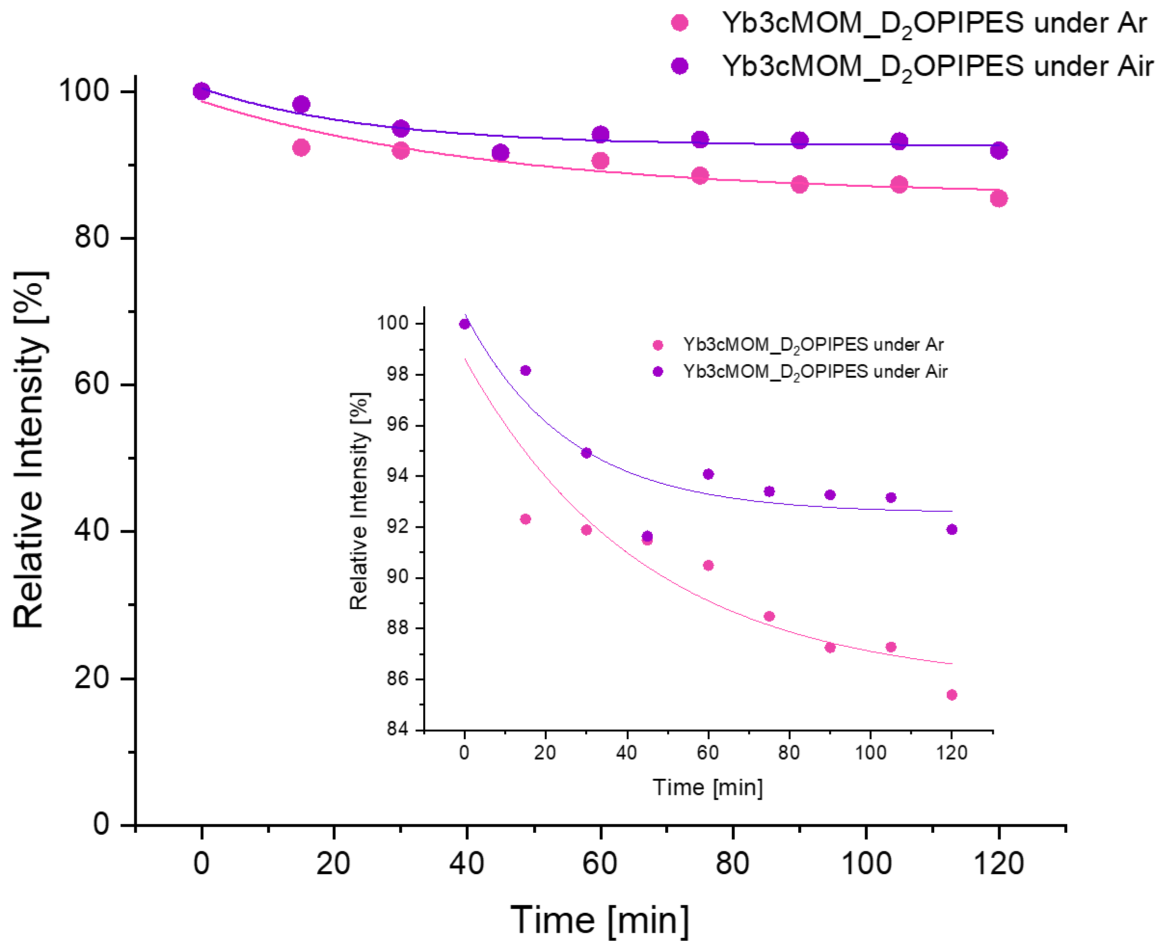

Figure S133. Relative emission intensity of **YbL3c^{MOM}** in D_2O-PIPES under Ar upon continuous light irradiation under identical sample absorptions. After blank signal subtraction each spectrum was integrated (346–647 nm), and the integrated intensity was divided by that at t_0 and multiplied by 100%.

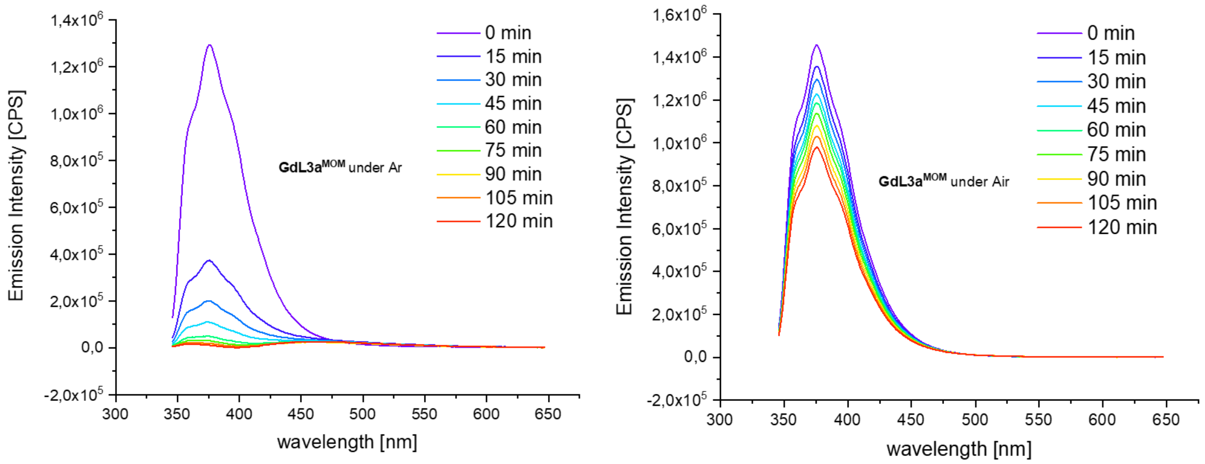

Figure S134. Steady-state fluorescence spectra of **GdL3a^{MOM}** under Ar upon continuous light irradiation under identical samples absorptions (A = 0.10). [**GdL3a^{MOM}**] = 10 µM in 10 mM PIPES-buffered solutions, pH = 6.5; λ_{ex} = 331 nm, front slit: 3 nm, exit slit: 2.5 nm.

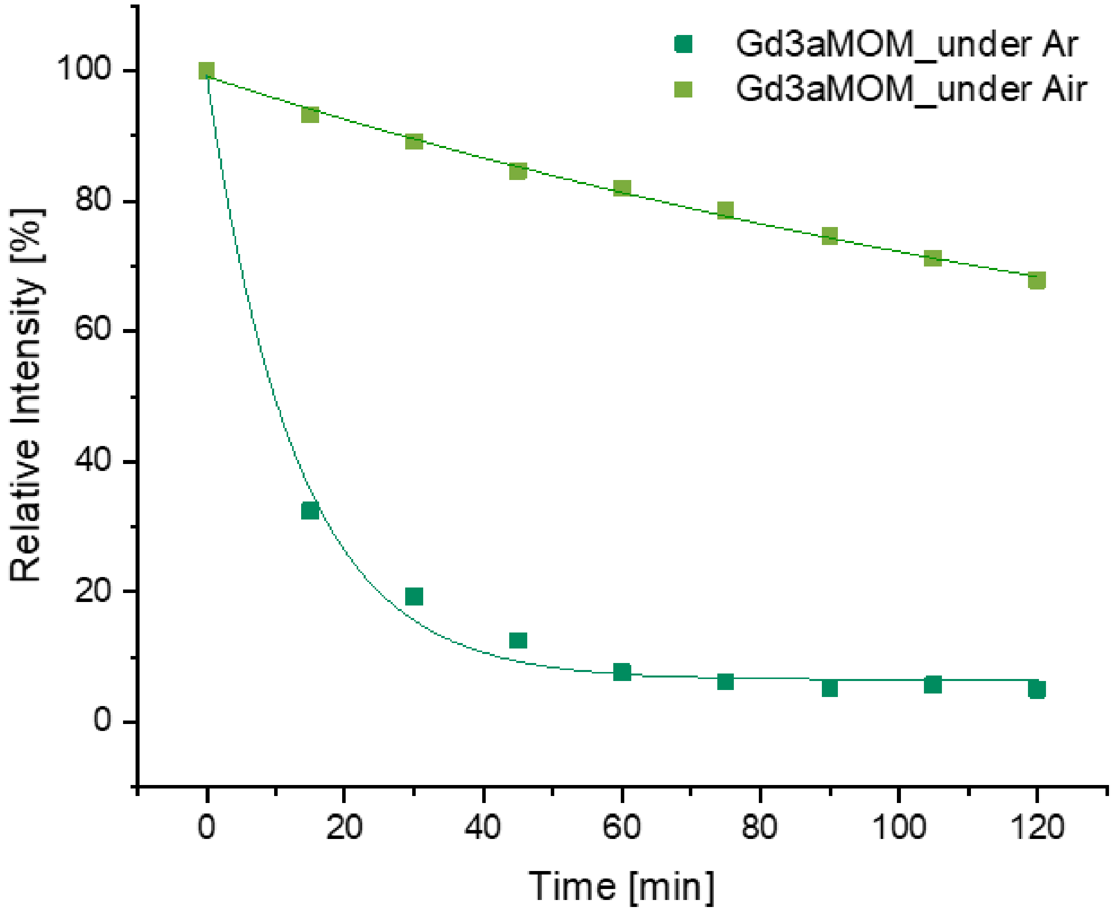

Figure S135. Relative emission intensity of **GdL3a^{MOM}** under Ar upon continuous light irradiation under identical sample absorptions. After blank signal subtraction each spectrum was integrated (346–647 nm), and the integrated intensity was divided by that at t_0 and multiplied by 100%.

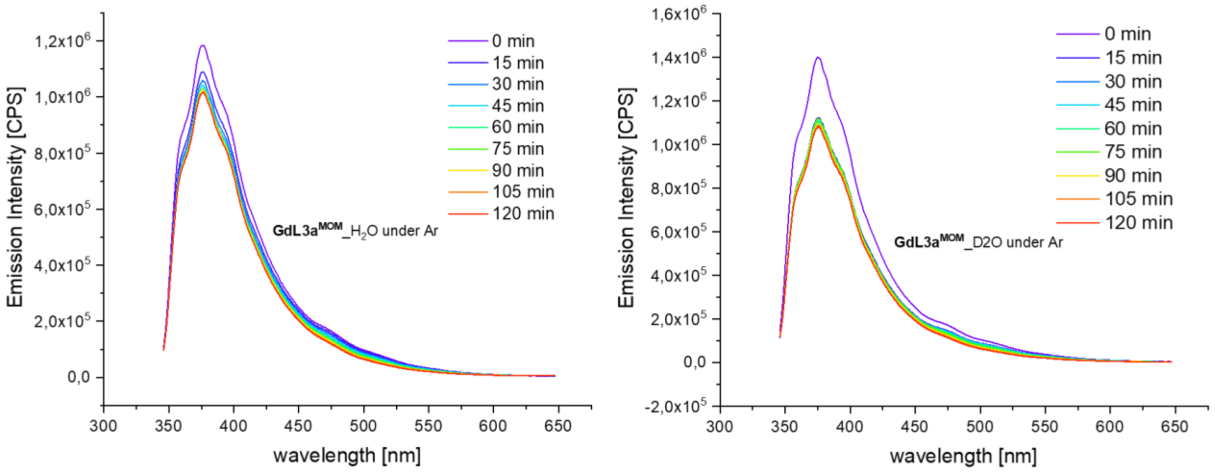

Figure S136. Steady-state fluorescence spectra of **GdL3a**$^{\mathbf{MOM}}$ under Ar upon continuous light irradiation under identical samples absorptions (A = 0.10). [**GdL3a**$^{\mathbf{MOM}}$] = 10 µM in H_2O (pH: 6.78-8.4,) and D_2O (pH: 7.6-8.5); λ_{ex} = 331 nm, front slit: 3 nm, exit slit: 2.5 nm.

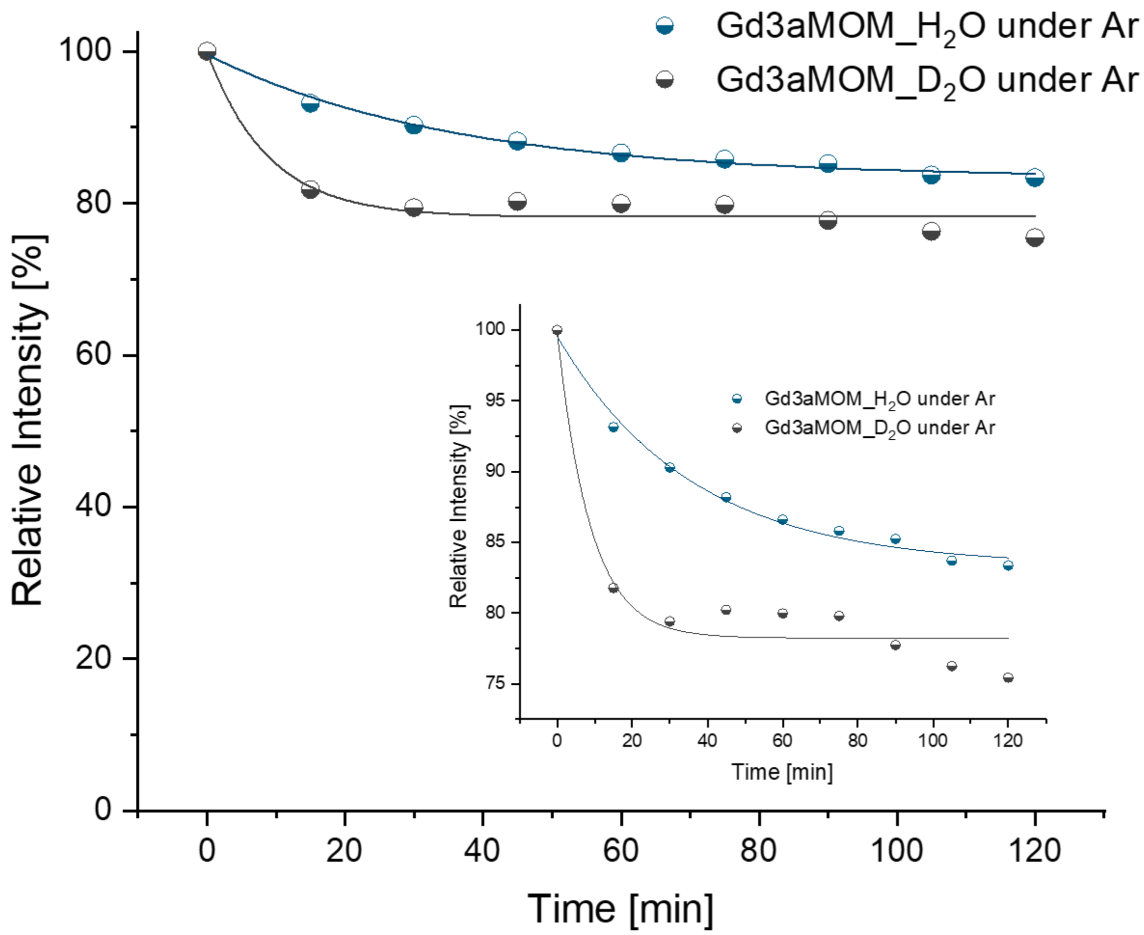

Figure S137. Relative emission intensity of **GdL3a**$^{\mathbf{MOM}}$ under Ar upon continuous light irradiation under identical sample absorptions. After blank signal subtraction each spectrum was integrated (346–647 nm), and the integrated intensity was divided by that at t_0 and multiplied by 100%.

CALCULATION OF FRANCK-CONDON FACTORS

The Franck-Condon factors were calculated for $T_1 \rightarrow {}^2F_{5/2}$ and $T_1 \rightarrow S_0$ processes via Equation S4:

$$FC_{(T=0)} = |\langle \chi_{a,p} | \chi_{b,0} \rangle = \frac{e^{-S} \cdot S^p}{p!} \qquad \text{(S4)}$$

$$E_{Stokes} = (2S - 1) \cdot \hbar\omega_Q \qquad \text{(S5)}$$

where S is the Huang-Rhys factor related to Stokes shift (either taken as S = 2 for *4f-4f* transitions,[25] or calculated as S = 1.7 using Ref [26] and rounded to ~2), p is the reduced energy gap (estimated in units of $\hbar\omega_Q$ between |a⟩ and |b⟩ υ=0 levels).

Table S22. Huang-Rhys factor calculated for **YbL^{MOM}**.

Complex	Absorption max [cm^{-1}]	Emission max [cm^{-1}]	E_{Stokes} [cm^{-1}]	$\hbar\omega_Q$ [cm^{-1}] [a]	S [b]
**YbL0^{MOM}**	30211	27473	2738	1137	2
**YbL1^{MOM}**	30211	27473	2738	1137	2
**YbL2^{MOM}**	30211	27397	2814	1137	2
**YbL3a^{MOM}**	30211	27397	2814	1137	2
**YbL3b^{MOM}**	30211	27397	2814	1137	2
**YbL3c^{MOM}**	30211	27397	2814	1058	2

[a] Calculated as difference between the local maxima of antenna π→π* absorption bands (344/343 nm vs 331 nm, from Figure S65). [b] Calculated by using Eq. S5.

Table S23. Franck-Condon factors and other parameters calculated for **YbL^{MOM}**.

Complex	$\hbar\omega_Q$ [cm^{-1}] [a]	T_1 [cm^{-1}] [b]	p(T_1) [c]	Δ(T_1-$^2F_{5/2}$) [cm^{-1}] [d]	p(Δ(T_1-$^2F_{5/2}$)) [e]	FC ($T_1 \rightarrow {}^2F_{5/2}$) [f]	FC ($T_1 \rightarrow S_0$) [f]	$\frac{FC(T_1 \rightarrow {}^2F_{5/2})}{FC(T_1 \rightarrow S_0)}$
**YbL0^{MOM}**	1137	22472	20	12212	11	$6.9\ 10^{-6}$	$5.8\ 10^{-14}$	$1.2\ 10^{8}$
**YbL1^{MOM}**	1137	22727	20	12467	11	$6.9\ 10^{-6}$	$5.8\ 10^{-14}$	$1.2\ 10^{8}$
**YbL2^{MOM}**	1137	22523	20	12263	11	$6.9\ 10^{-6}$	$5.8\ 10^{-14}$	$1.2\ 10^{8}$
**YbL3a^{MOM}**	1137	22624	20	12364	11	$6.9\ 10^{-6}$	$5.8\ 10^{-14}$	$1.2\ 10^{8}$
**YbL3b^{MOM}**	1137	22573	20	12313	11	$6.9\ 10^{-6}$	$5.8\ 10^{-14}$	$1.2\ 10^{8}$
**YbL3c^{MOM}**	1058	22573	21	12313	12	$1.2\ 10^{-6}$	$5.6\ 10^{-15}$	$2.1\ 10^{8}$

[a] Calculated as difference between the local maxima of antenna π→π* absorption bands (344/343 nm vs 331 nm, from Figure S65). [b] Taken from the 77 K spectra of **GdL^{MOM}**, see figure S74. [c] Calculated as $T_1/\hbar\omega_Q$. [d] Calculated as the difference between T_1 and $^2F_{5/2}$ (10260 cm^{-1}). [e] Calculated as ($T_1 - {}^2F_{5/2}$)/ $\hbar\omega_Q$. [f] Calculated by using Eq. S4.

DRIVING FORCE FOR PHOTOINDUCED ELECTRON TRANSFER

The driving force for photoinduced electron transfer from the excited carbostyril (MOM) was calculated according to Equation (S6):

$$\Delta G(eT) = \left(E_{ox}^{MOM} - E_{red}^{YbL}\right) - E_S^{MOM} - \frac{{e_0}^2}{\varepsilon r} \qquad \text{(S6)}$$

with $\Delta G(eT)$ the free energy of electron transfer, E_{ox}^{MOM} the oxidation potential of the ground state carbostyril antenna (1.76 V vs. NHE),[1] E_{red}^{YbL} the reduction potential of **YbL** complex, E_S^{MOM} the excited state of oxidation potential of the antenna estimated from its singlet excited state (3.53 eV),[1] and $\frac{{e_0}^2}{\varepsilon r}$ the attraction between the radical ion pair (~0.15 eV for an exciplex).[7]

Table S24. Reduction potential of **YbL$^{\mathbf{MOM}}$** and PeT driving force from the excited carbostyril (MOM).

Complex	E_{red}^{YbL} [V vs NHE]	$\Delta G(eT)$[eV]
**YbL0$^{\mathbf{MOM}}$**	–2.423	0.50
**YbL1$^{\mathbf{MOM}}$**	–1.924	0.00
**YbL2$^{\mathbf{MOM}}$**	–1.695	–0.23
**YbL3a$^{\mathbf{MOM}}$**	–1.546	–0.37
**YbL3b$^{\mathbf{MOM}}$**	–1.557	–0.36
**YbL3c$^{\mathbf{MOM}}$**	–1.598	–0.32

In order to have all **YbL** complexes with $\Delta G(eT) < 0$ the following criteria would need to be fulfilled:

- The **YbL** complexes to be easier to reduce ($E_{red}^{YbL} > –1.924$ V vs NHE)
- The antenna to be easier to oxidize ($E_{ox}^{MOM} < 1.26$ V vs NHE)
- Or a combined effect of at least –500 mV

In order to have all **YbL** complexes with $\Delta G(eT) > 0$ the following criteria would need to be fulfilled:

- The **YbL** complexes to be harder to reduce ($E_{red}^{YbL} < –1.924$ V vs NHE)
- The antenna to be harder to oxidize ($E_{ox}^{MOM} > 2.13$ V vs NHE)
- Or a combined effect of at least +370 mV

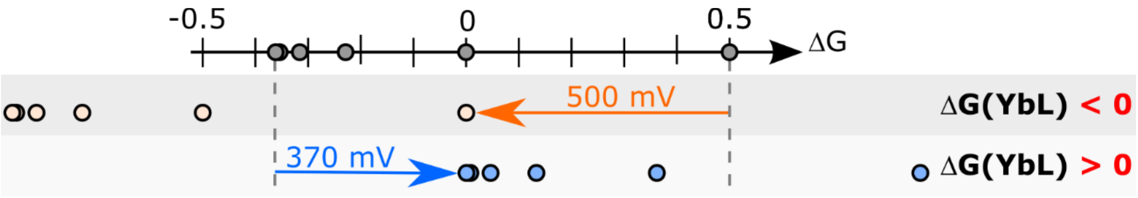

Figure S138. Impact of **YbL** reduction potential and antenna oxidation potential on $\Delta G(eT)$.

$$\Delta G(H^+_{reduction}) = \left(E_{ox}^{MOM} - E_{red}^{H^+}\right) - E_T^{MOM} - \frac{{e_0}^2}{\varepsilon r} = (1.76\ eV - 0\ eV) - 2.80\ eV - 0\ eV = -1.04\ eV \qquad \text{(S7)}$$

E_{ox}^{MOM} is the antenna oxidaton potential, $E_{red}^{H^+}$ is the H^+ reduction potential (both vs NHE), E_T^{MOM} is the antenna triplet energy, while 0 eV is assumed as a worst-case estimate for $\frac{{e_0}^2}{\varepsilon r}$, i.e. when there is no Coulombic stabilization.

^1H, ^{13}C, and ^{19}F NMR spectra

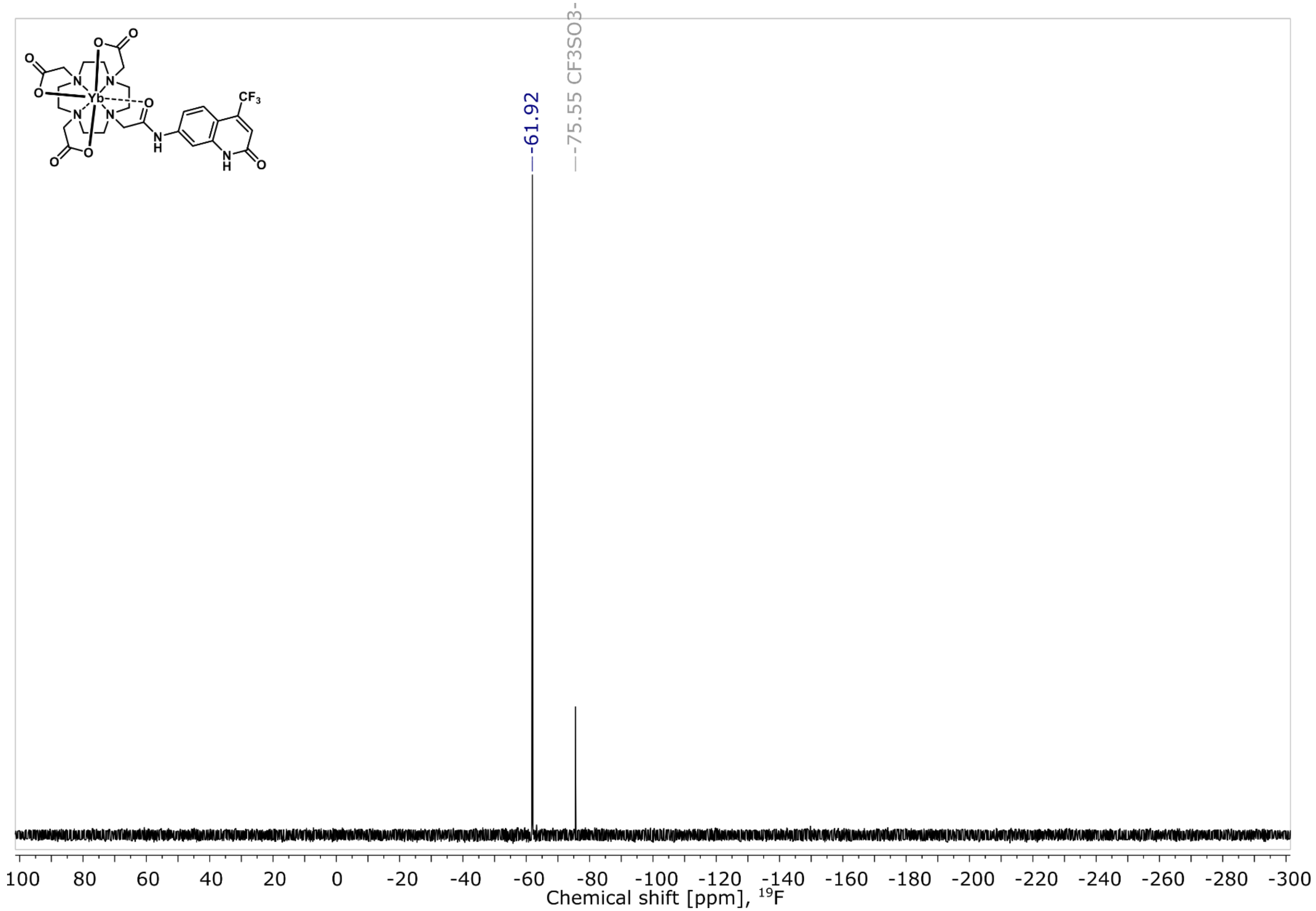

Figure S139. ^{19}F NMR spectrum (376 MHz) of **YbL0^{CF3}** measured in D_2O at r.t.

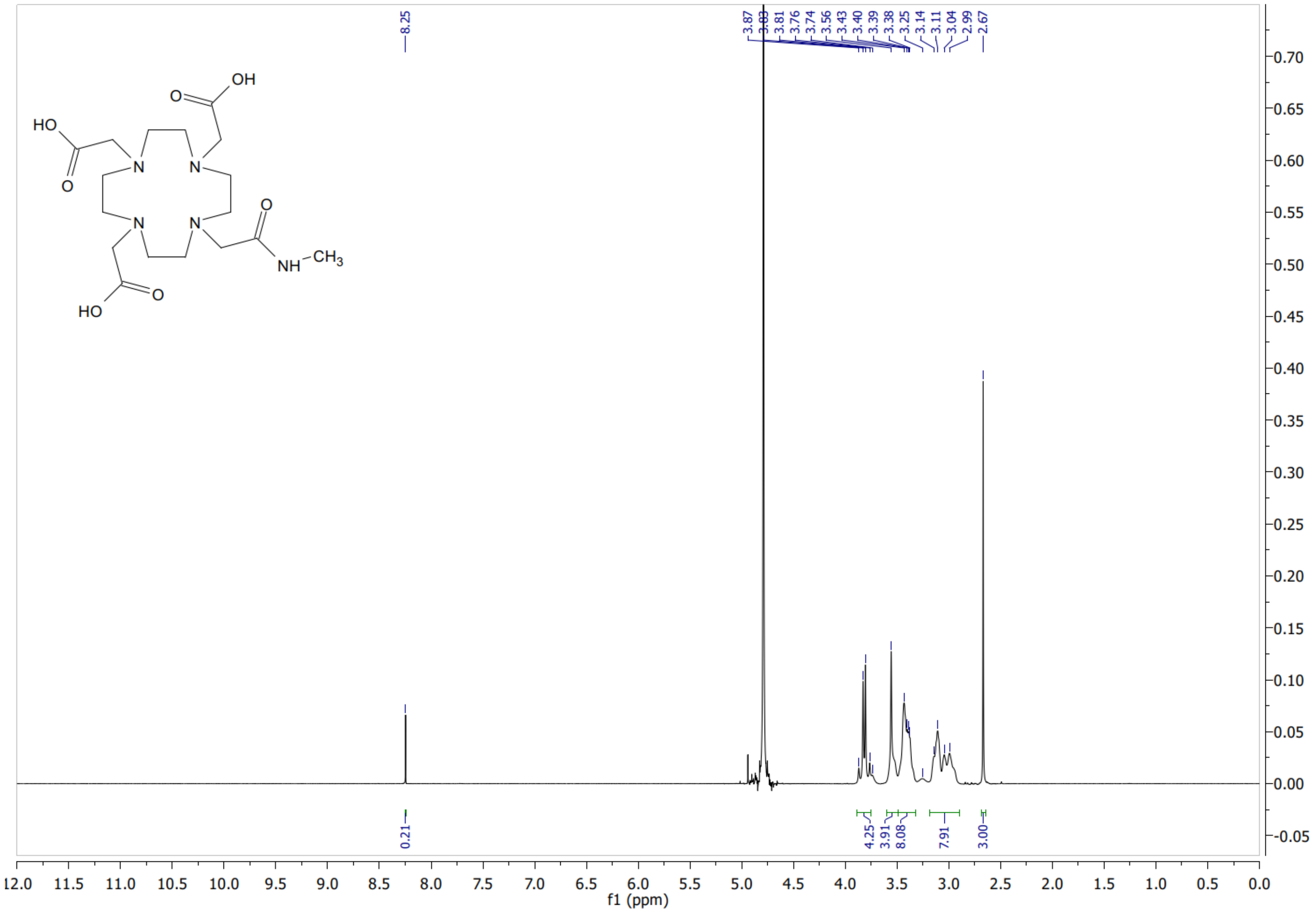

Figure S140. ^{1}H NMR spectrum of **L0^m** (400 MHz, D_2O).

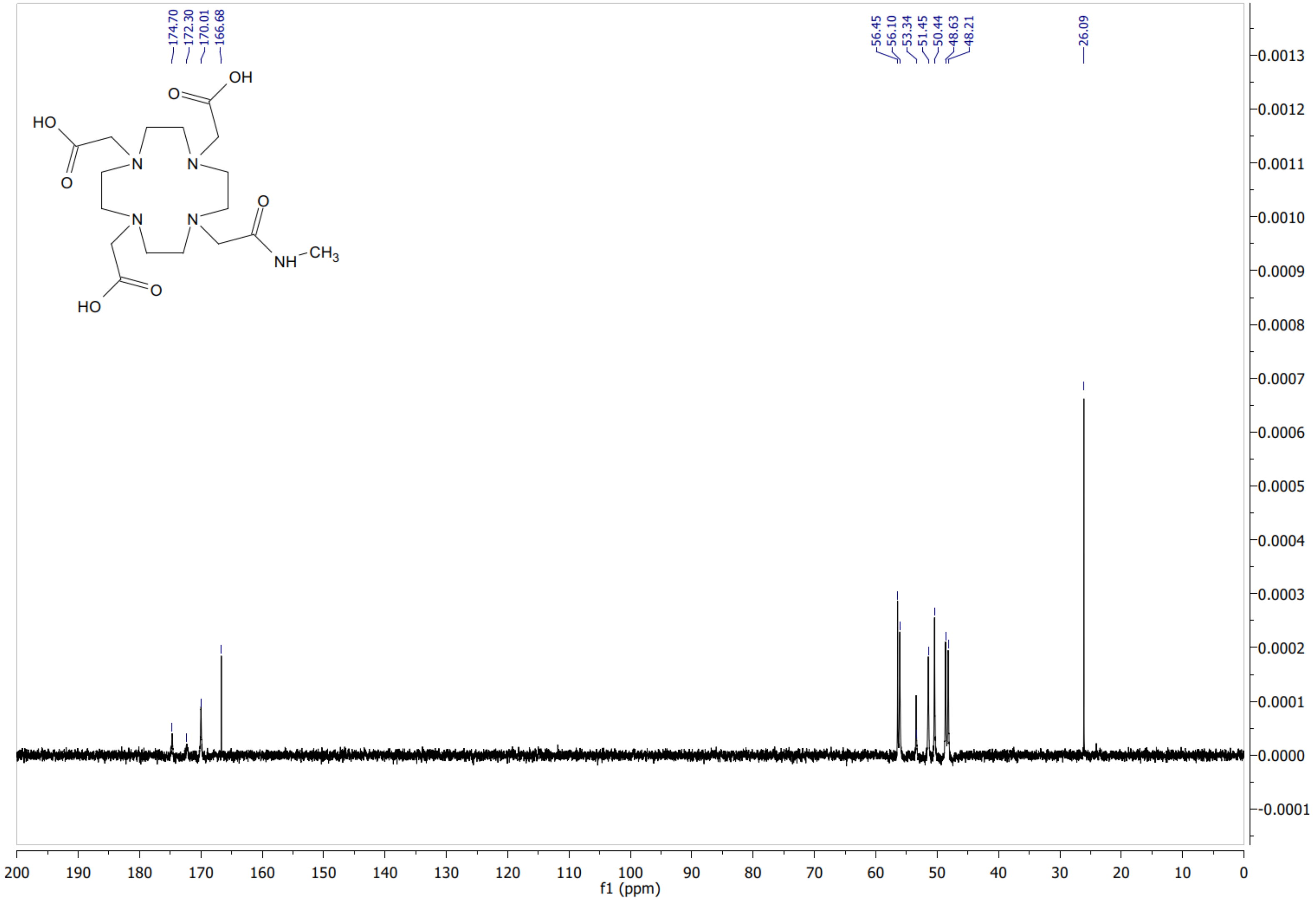

Figure S141. ^{13}C NMR spectrum of **L0^{m}** (101 MHz, D_2O).

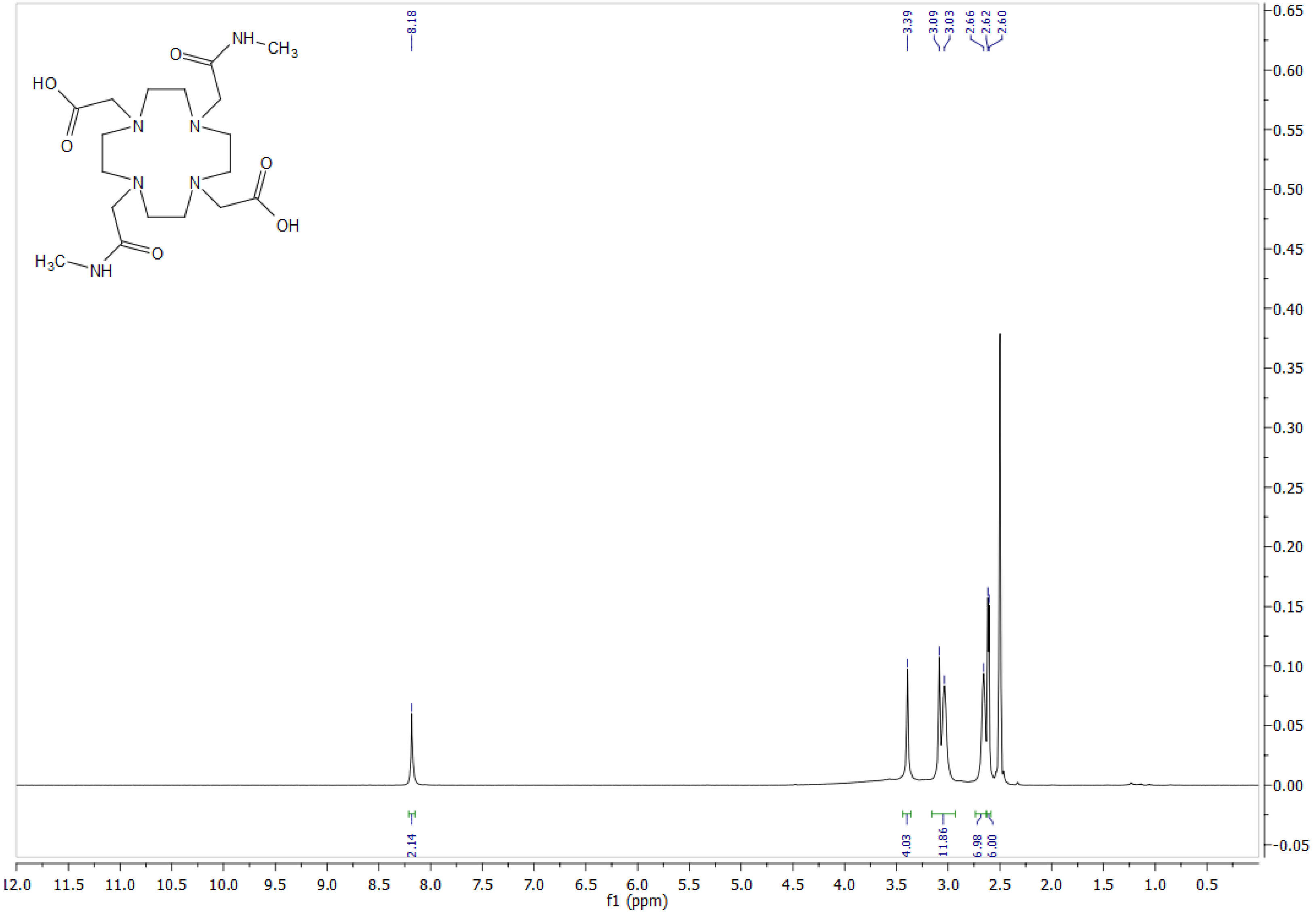

Figure S142. ^{1}H NMR spectrum of **L1^m** (400 MHz, DMSO-d_6).

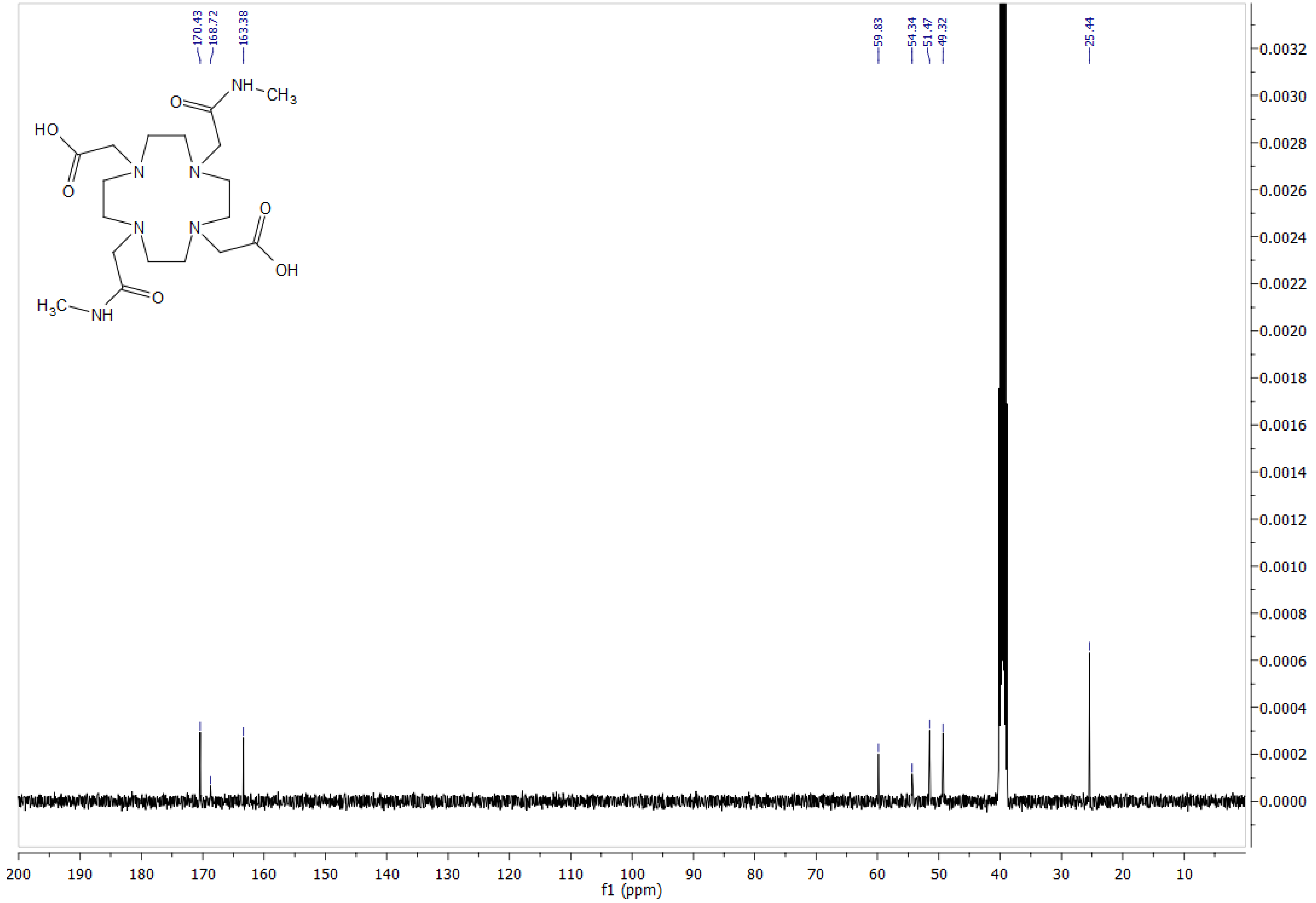

Figure S143. ^{13}C NMR spectrum of **L1^{m}** (101 MHz, DMSO-d_6).

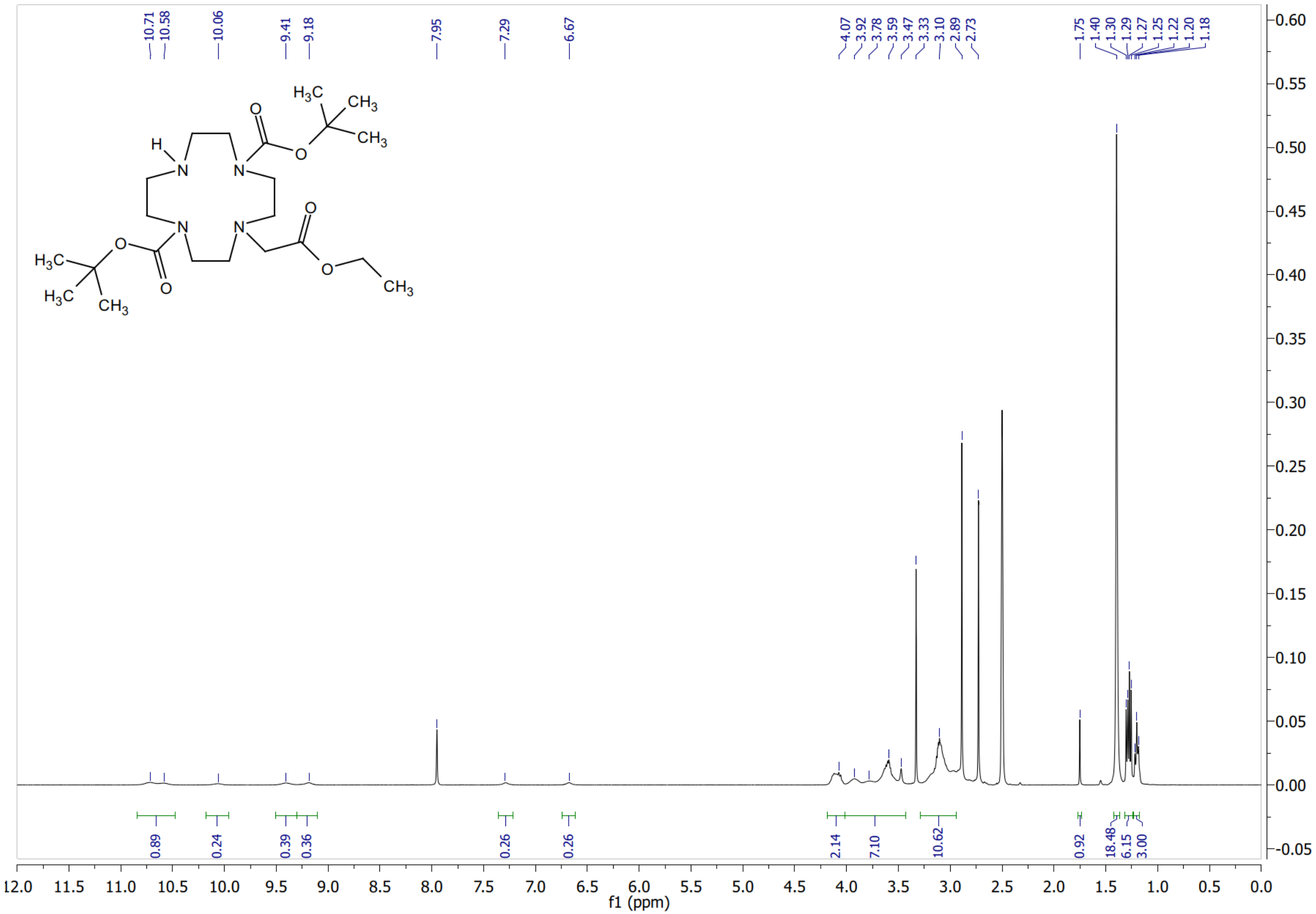

Figure S144. ^{1}H NMR spectrum of **S7** (400 MHz, DMSO-d_6).

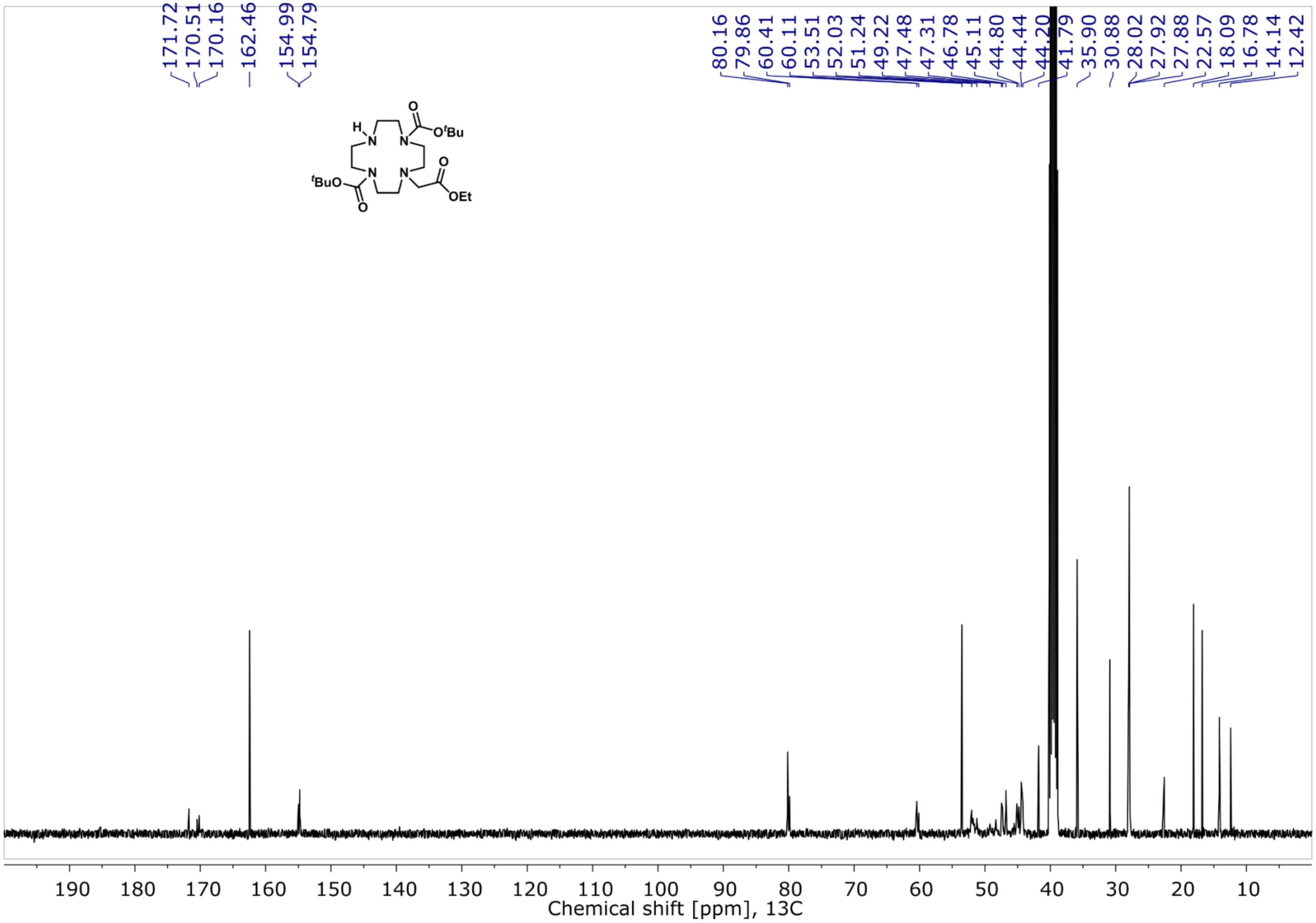

Figure S145. ^{13}C NMR spectrum of **S7** (101 MHz, DMSO-d_6).

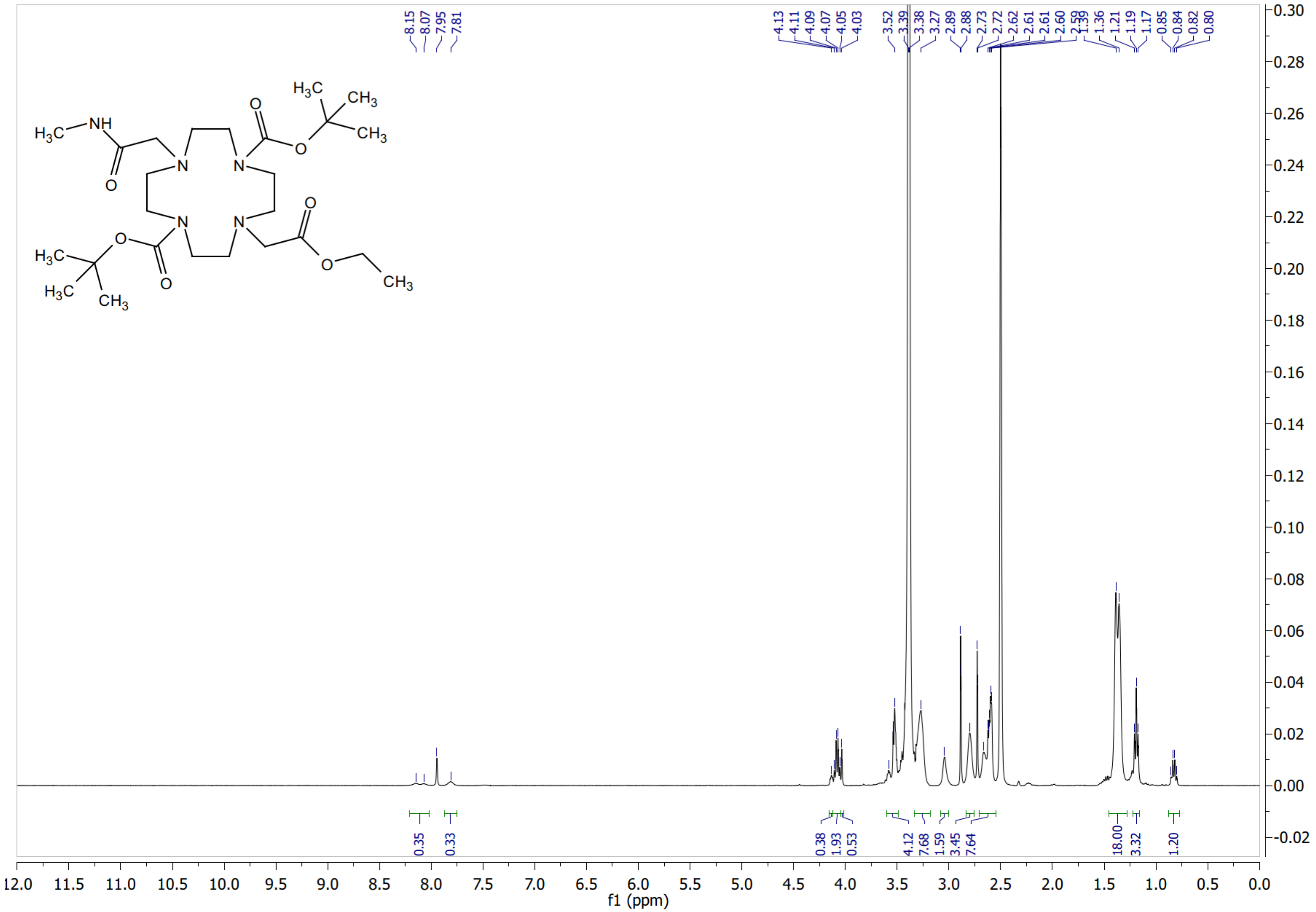

Figure S146. ^{1}H NMR spectrum of **S8** (400 MHz, DMSO-d_6).

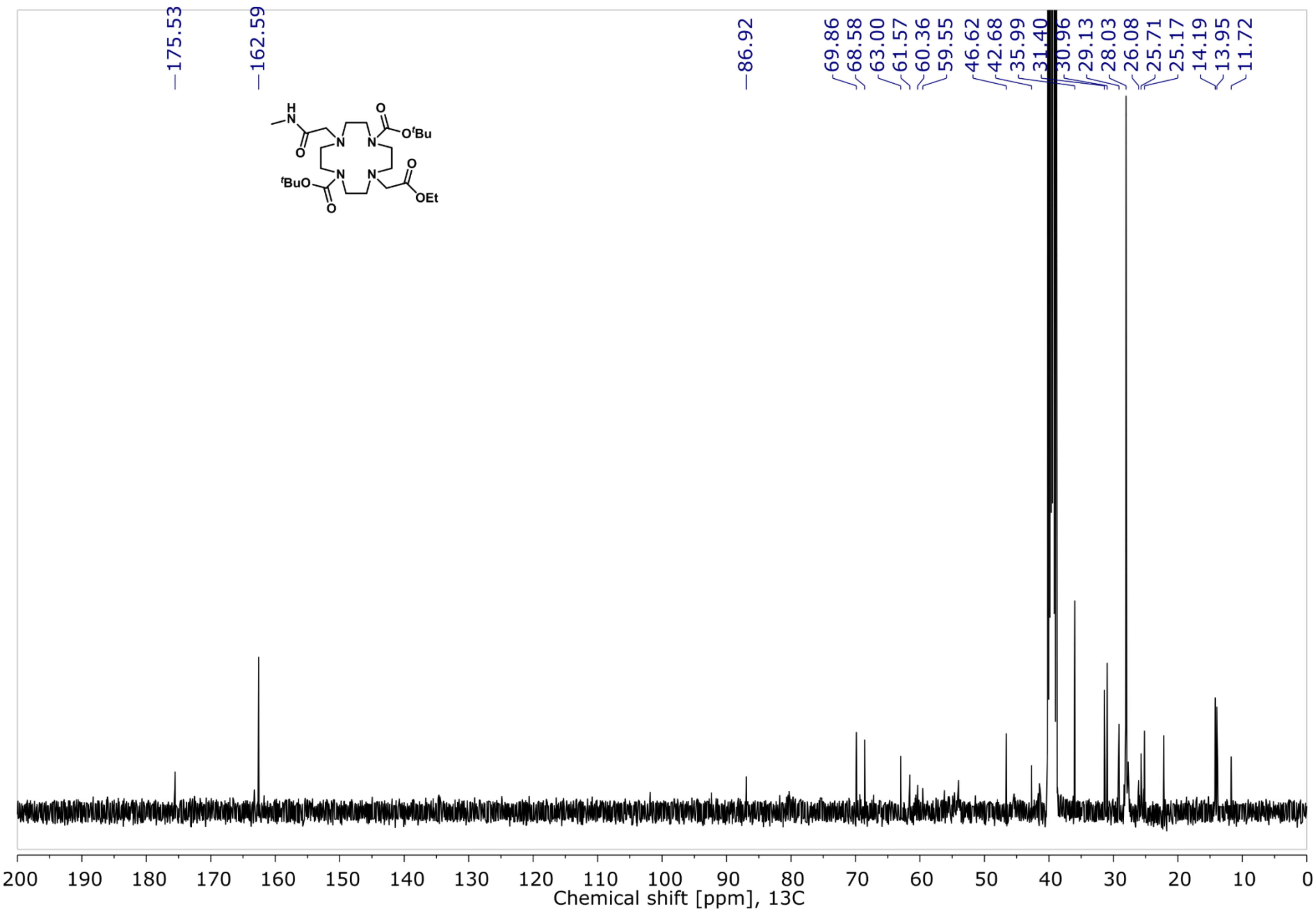

Figure S147. ^{13}C NMR spectrum of **S8** (101 MHz, DMSO-d_6).

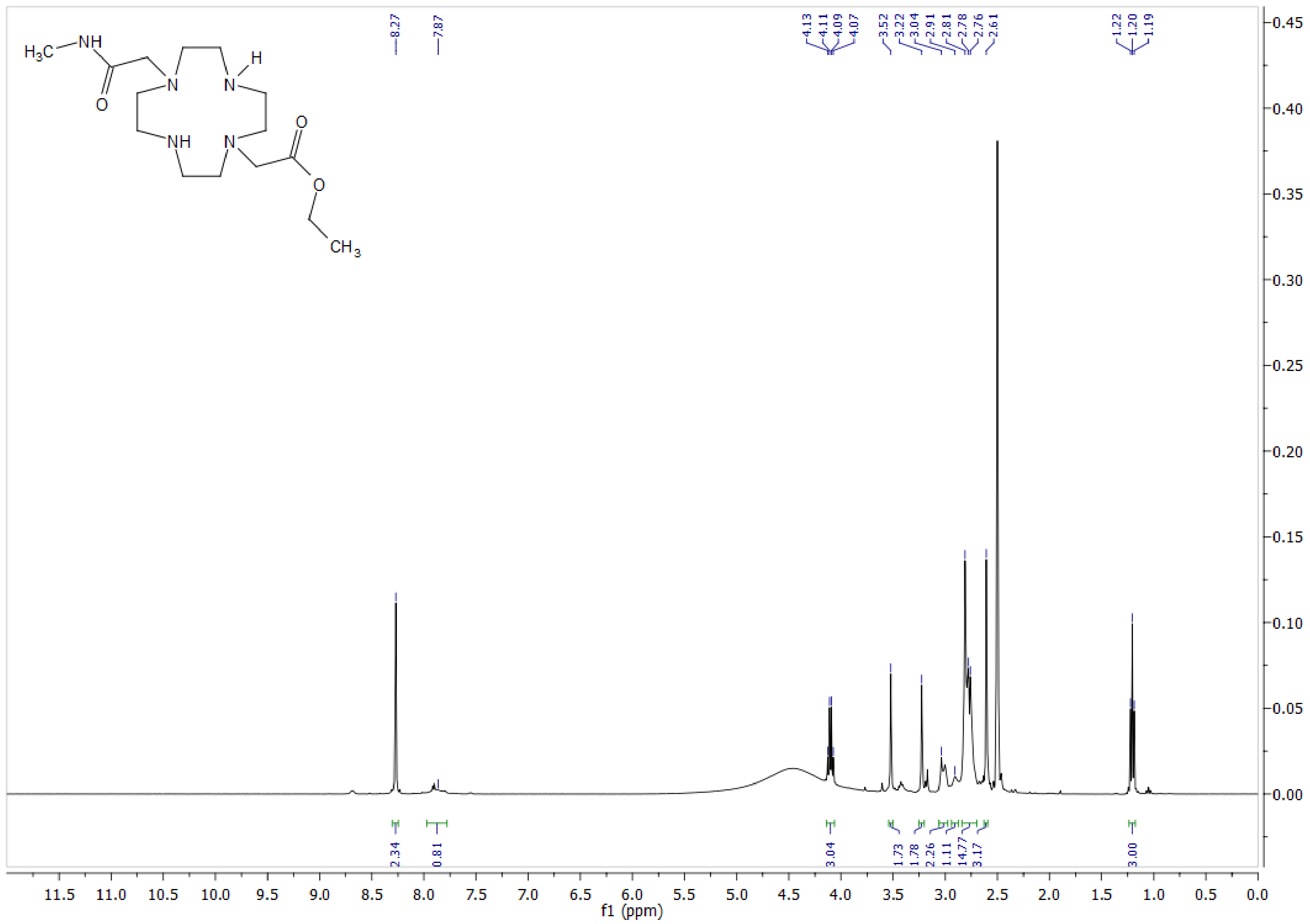

Figure S148. ^{1}H NMR spectrum of **S9** (400 MHz, DMSO-*d*$_6$).

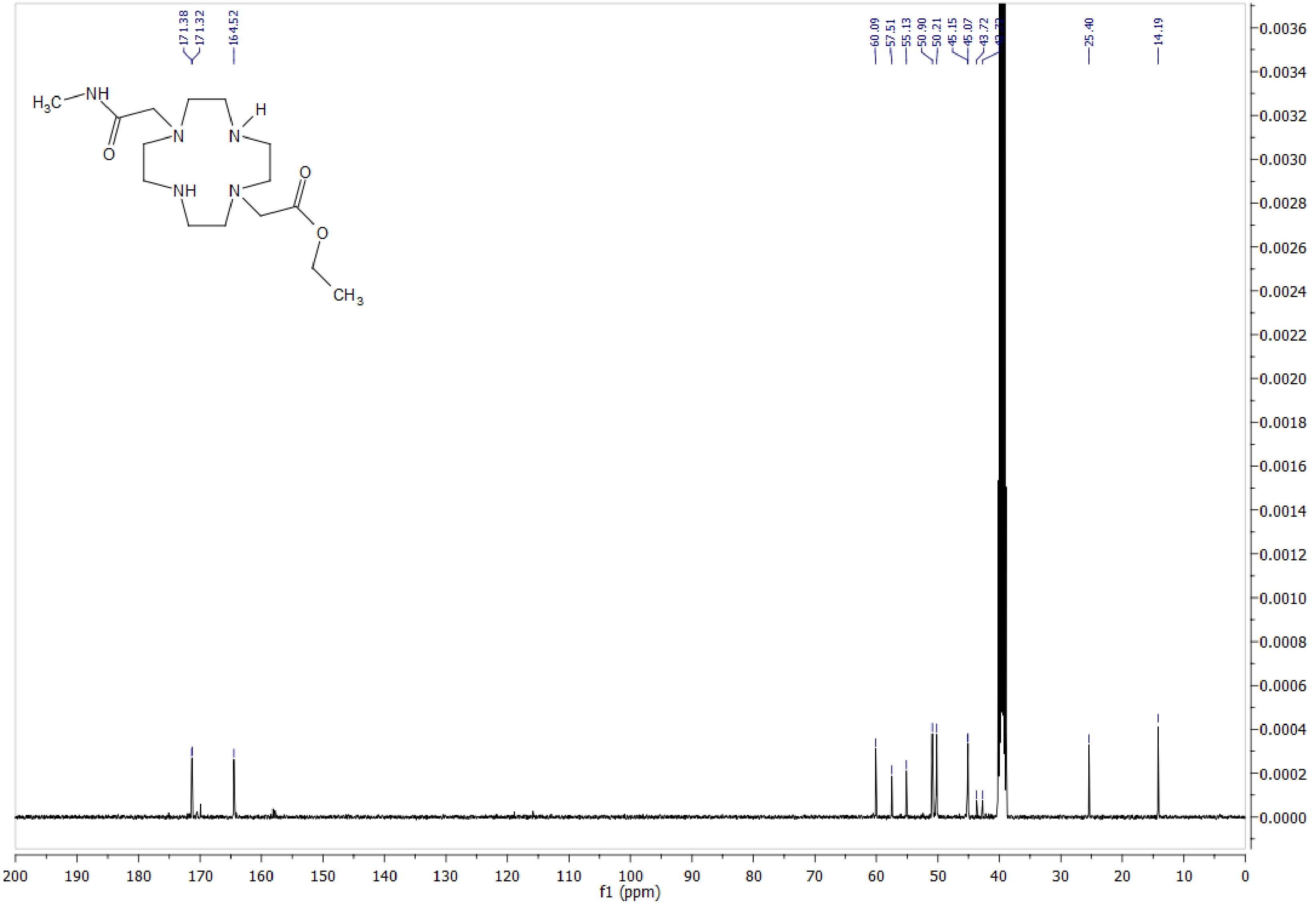

Figure S149. ^{13}C NMR spectrum of **S9** (101 MHz, DMSO-d_6).

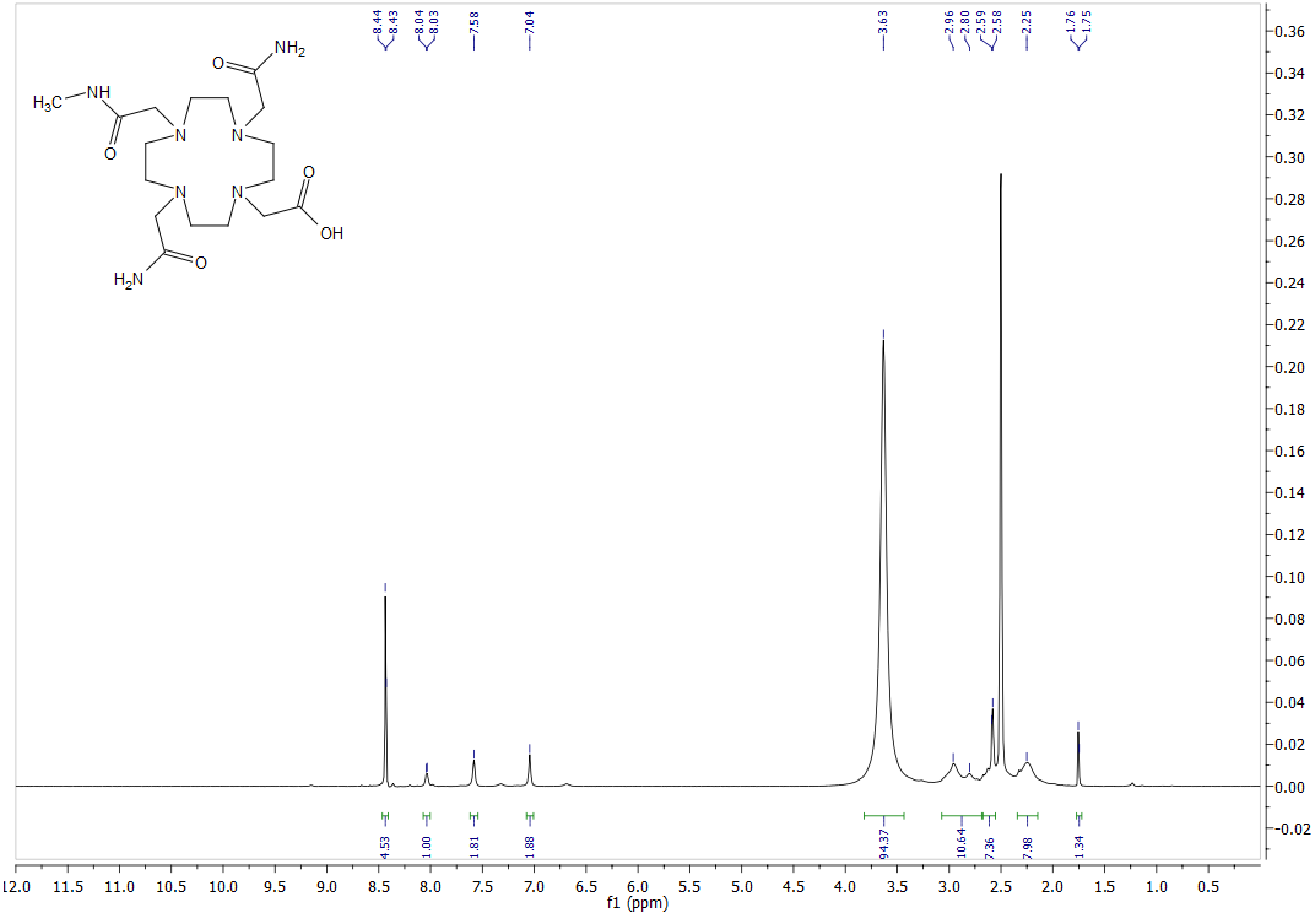

Figure S150. ^{1}H NMR spectrum of **L2^{m}** (400 MHz, DMSO-d_6).

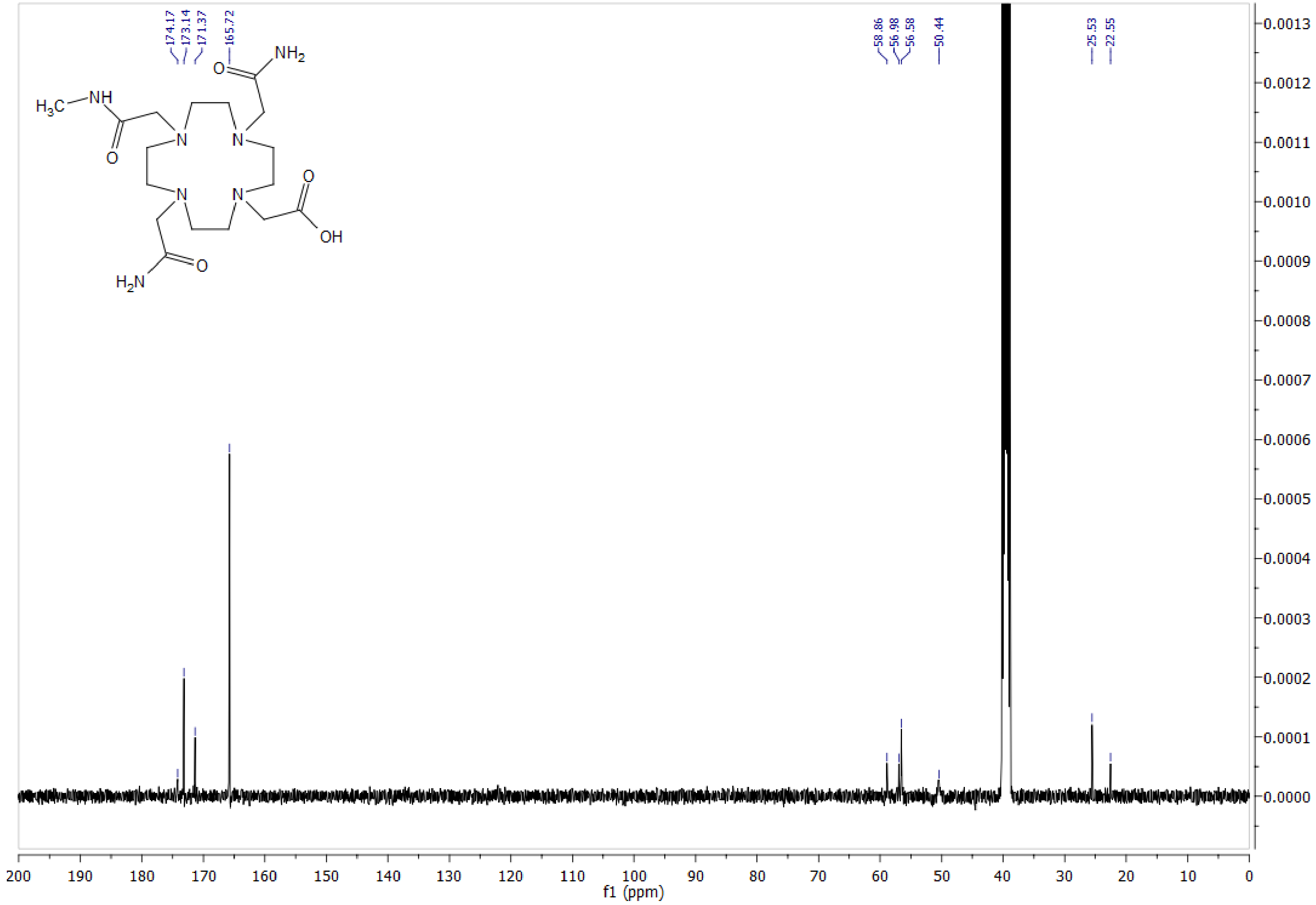

Figure S151. ^{13}C NMR spectrum of **L2^{m}** (101 MHz, DMSO-d_6).

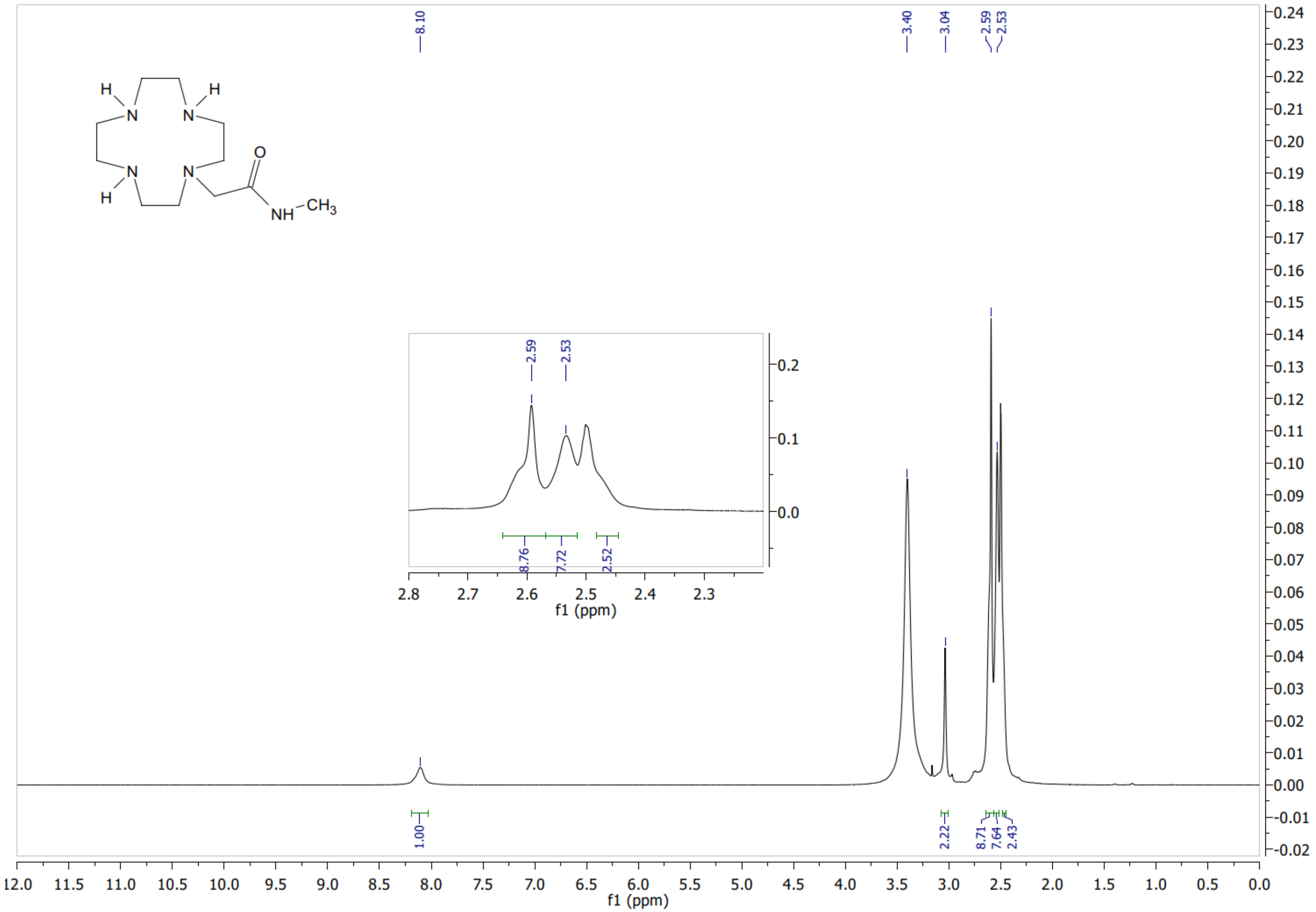

Figure S152. ^{1}H NMR spectrum of **S10** (400 MHz, DMSO-d_6).

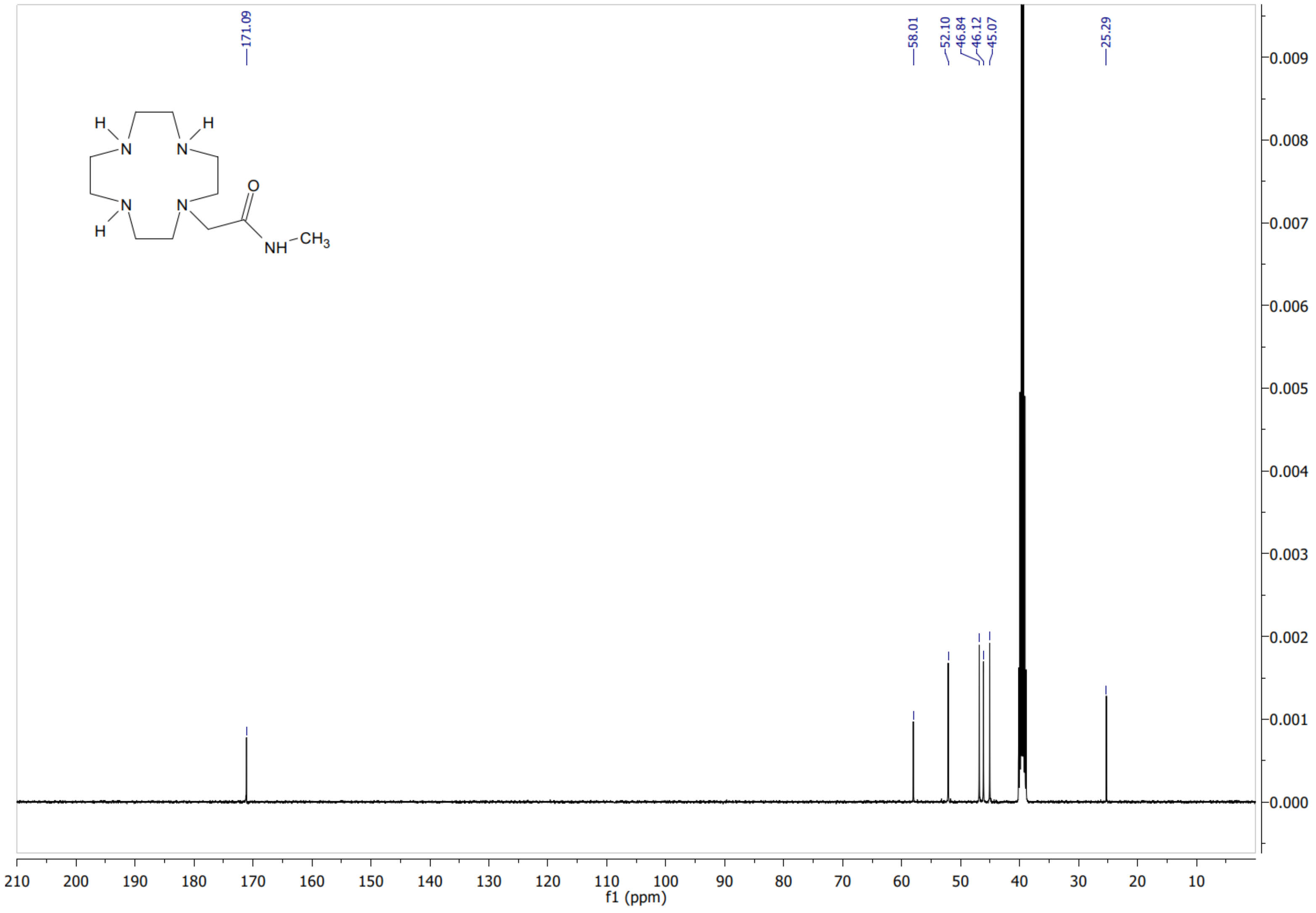

Figure S153. ^{13}C NMR spectrum of **S10** (101 MHz, DMSO-d_6).

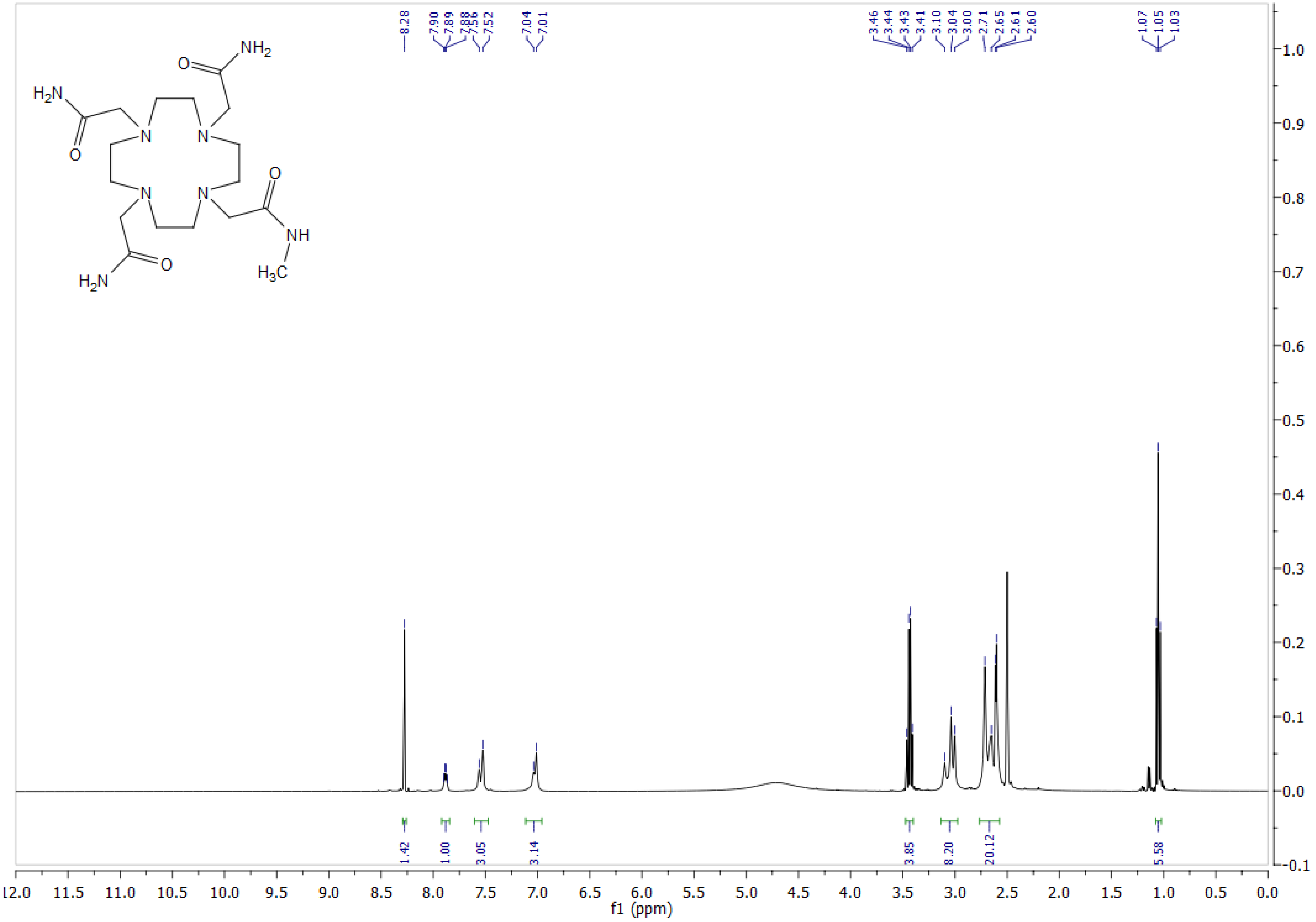

Figure S154. ^{1}H NMR spectrum of **L3[m]** (400 MHz, DMSO-d_6).

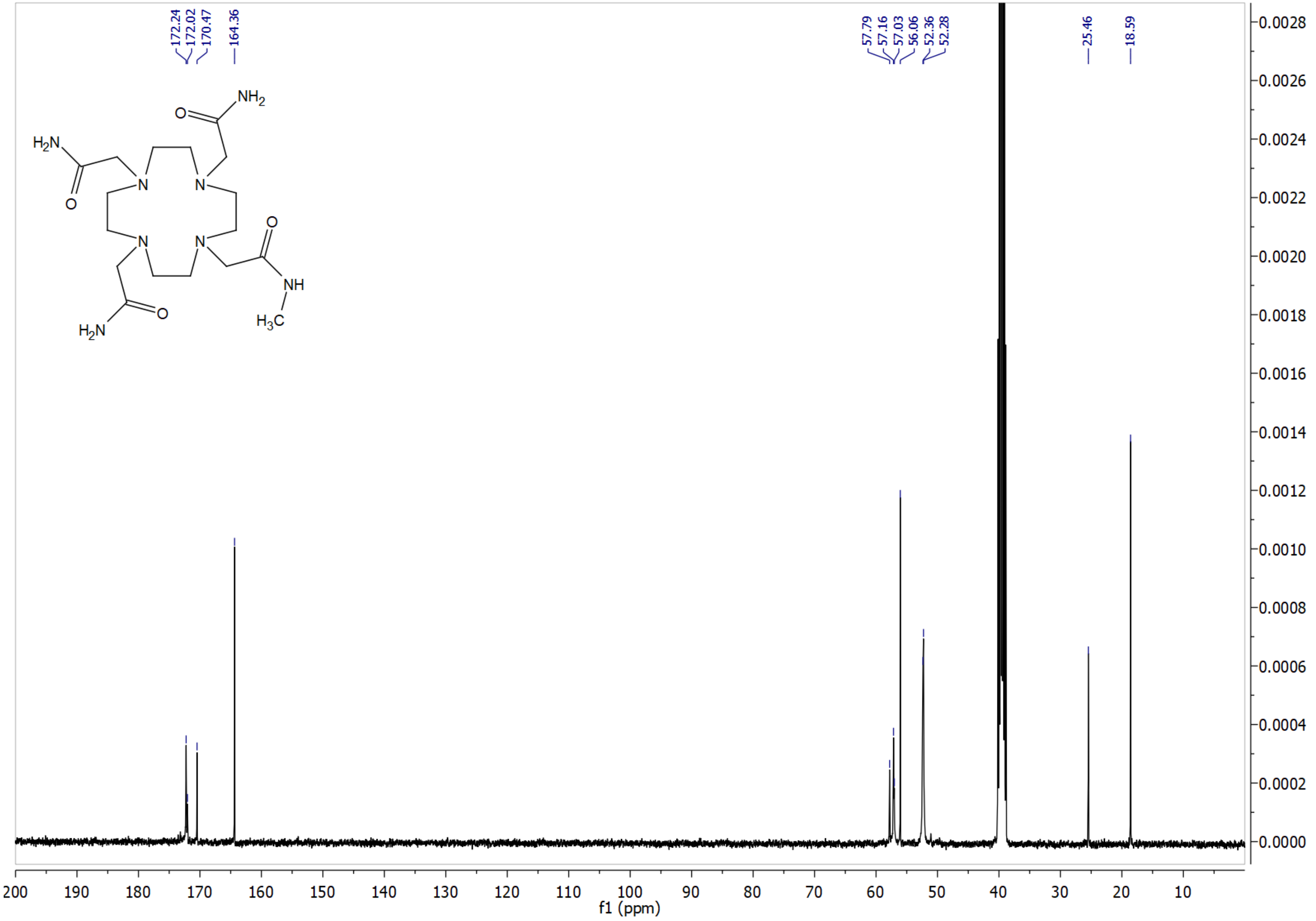

Figure S155. ^{13}C NMR spectrum of **L3ᵐ** (101 MHz, DMSO-d_6).

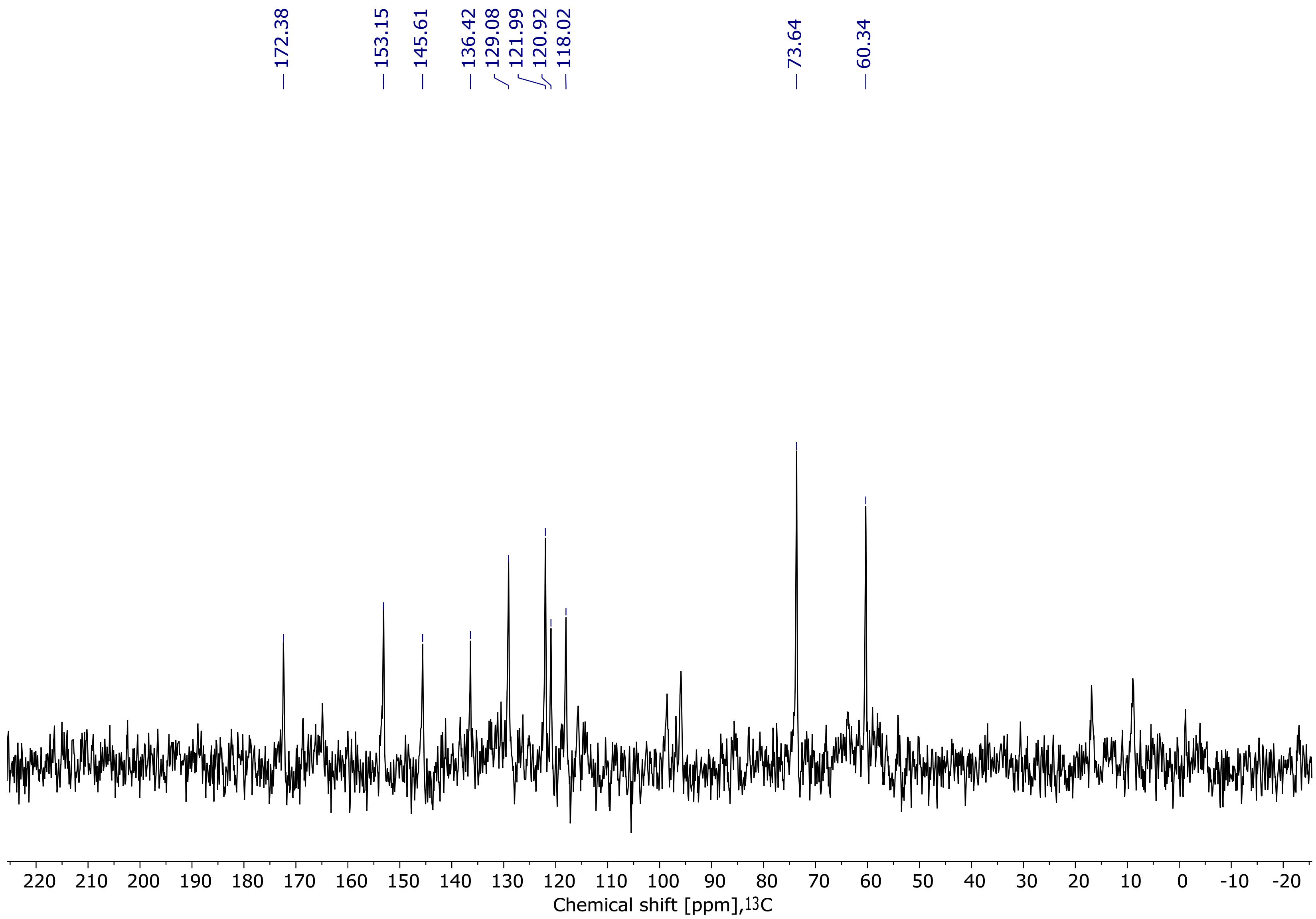

Figure S156. ^{13}C NMR spectrum of **YbL3a^{MOM}** (101 MHz, D_2O).

REFERENCES

1. Kovacs, D.; Kocsi, D.; Wells, J. A. L.; Kiraev, S. R.; Borbas, K. E., Electron transfer pathways in photoexcited lanthanide(iii) complexes of picolinate ligands. *Dalton Trans.* **2021,** *50*(12), 4244-4254.
2. Prasuhn, J. D. E.; Yeh, R. M.; Obenaus, A.; Manchester, M.; Finn, M. G., Viral MRI contrast agents: coordination of Gd by native virions and attachment of Gd complexes by azide–alkyne cycloaddition. *Chem. Commun.* **2007,** 1269-1271.
3. Hopper, L. E.; Allen, M. J., Rapid synthesis of 1,7-bis(t-butoxycarbonylmethyl)-1,4,7,10-tetraazacyclododecane (DO2A-t-Bu ester). *Tetrahedron Lett.* **2014,** *55*(40), 5560-5561.
4. Kovacs, D.; Mathieu, E.; Kiraev, S. R.; Wells, J. A. L.; Demeyere, E.; Sipos, A.; Borbas, K. E., Coordination Environment-Controlled Photoinduced Electron Transfer Quenching in Luminescent Europium Complexes. *J. Am. Chem. Soc.* **2020,** *142*(30), 13190-13200.
5. Suzuki, K.; Kobayashi, A.; Kaneko, S.; Takehira, K.; Yoshihara, T.; Ishida, H.; Shiina, Y.; Oishi, S.; Tobita, S., Reevaluation of absolute luminescence quantum yields of standard solutions using a spectrometer with an integrating sphere and a back-thinned CCD detector. *Phys. Chem. Chem. Phys.* **2009,** *11*(42), 9850-9860.
6. Glasoe, P. K.; Long, F. A., Use of glass electrodes to measure acidities in deuterium oxide. *J. Phys. Chem.* **1960,** *64*(1), 188-190.
7. Rößler, P.; Mathieu, D.; Gossert, A. D., Enabling NMR Studies of High Molecular Weight Systems Without the Need for Deuteration: The XL-ALSOFAST Experiment with Delayed Decoupling. *Angew. Chem. Int. Ed.* **2020,** *59*(43), 19329-19337.
8. Nielsen, L. G.; Sørensen, T. J., Including and Declaring Structural Fluctuations in the Study of Lanthanide(III) Coordination Chemistry in Solution. *Inorg. Chem.* **2020,** *59*(1), 94-105.
9. De León-Rodríguez, L. M.; Kovacs, Z.; Esqueda-Oliva, A. C.; Miranda-Olvera, A. D., Highly regioselective N-trans symmetrical diprotection of cyclen. *Tetrahedron Lett.* **2006,** *47*(39), 6937-6940.
10. Pulukkody, K. P.; Norman, T. J.; Parker, D.; Royle, L.; Broan, C. J., Synthesis of charged and uncharged complexes of gadolinium and yttrium with cyclic polyazaphosphinic acid ligands for in vivo applications. *J. Chem. Soc., Perkin Trans. 2* **1993,** 605-620.
11. Kovacs, D.; Lu, X.; Mészáros, L. S.; Ott, M.; Andres, J.; Borbas, K. E., Photophysics of Coumarin and Carbostyril-Sensitized Luminescent Lanthanide Complexes: Implications for Complex Design in Multiplex Detection. *J. Am. Chem. Soc.* **2017,** *139*(16), 5756-5767.
12. Nielsen, L. G.; Junker, A. K. R.; Sørensen, T. J., Composed in the f-block: solution structure and function of kinetically inert lanthanide(iii) complexes. *Dalton Trans.* **2018,** *47*(31), 10360-10376.
13. Tropiano, M.; Kilah, N. L.; Morten, M.; Rahman, H.; Davis, J. J.; Beer, P. D.; Faulkner, S., Reversible Luminescence Switching of a Redox-Active Ferrocene–Europium Dyad. *J. Am. Chem. Soc.* **2011,** *133*(31), 11847-11849.
14. Vitha, T.; Kubíček, V.; Kotek, J.; Hermann, P.; Vander Elst, L.; Muller, R. N.; Lukeš, I.; Peters, J. A., Gd(iii) complex of a monophosphinate-bis(phosphonate) DOTA analogue with a high relaxivity; Lanthanide(iii) complexes for imaging and radiotherapy of calcified tissues. *Dalton Trans.* **2009,** 3204-3214.
15. Harnden, A. C.; Suturina, E. A.; Batsanov, A. S.; Senanayake, P. K.; Fox, M. A.; Mason, K.; Vonci, M.; McInnes, E. J. L.; Chilton, N. F.; Parker, D., Unravelling the Complexities of Pseudocontact Shift Analysis in Lanthanide Coordination Complexes of Differing Symmetry. *Angew. Chem. Int. Ed.* **2019,** *58*(30), 10290-10294.
16. Mason, K.; Rogers, N. J.; Suturina, E. A.; Kuprov, I.; Aguilar, J. A.; Batsanov, A. S.; Yufit, D. S.; Parker, D., PARASHIFT Probes: Solution NMR and X-ray Structural Studies of Macrocyclic Ytterbium and Yttrium Complexes. *Inorg. Chem.* **2017,** *56*(7), 4028-4038.
17. Urbanovský, P.; Kotek, J.; Carniato, F.; Botta, M.; Hermann, P., Lanthanide Complexes of DO3A–(Dibenzylamino)methylphosphinate: Effect of Protonation of the Dibenzylamino Group on the Water-Exchange Rate and the Binding of Human Serum Albumin. *Inorg. Chem.* **2019,** *58*(8), 5196-5210.
18. Krchová, T.; Gálisová, A.; Jirák, D.; Hermann, P.; Kotek, J., Ln(iii)-complexes of a DOTA analogue with an ethylenediamine pendant arm as pH-responsive PARACEST contrast agents. *Dalton Trans.* **2016,** *45*(8), 3486-3496.

19. Aime, S.; Botta, M.; Fasano, M.; Marques, M. P. M.; Geraldes, C. F. G. C.; Pubanz, D.; Merbach, A. E., Conformational and Coordination Equilibria on DOTA Complexes of Lanthanide Metal Ions in Aqueous Solution Studied by ^{1}H-NMR Spectroscopy. *Inorg. Chem.* **1997,** *36*(10), 2059-2068.
20. Aime, S.; Botta, M.; Ermondi, G., NMR study of solution structures and dynamics of lanthanide(III) complexes of DOTA. *Inorg. Chem.* **1992,** *31*(21), 4291-4299.
21. Jenkins, B. G.; Lauffer, R. B., Solution structure and dynamics of lanthanide(III) complexes of diethylenetriaminepentaacetate: a two-dimensional NMR analysis. *Inorg. Chem.* **1988,** *27*(26), 4730-4738.
22. Jenkins, B. G.; Lauffer, R. B., 2D NMR studies of paramagnetic lanthanide(III)-diethylenetriaminepentaacetate complexes. *J. Magnetic Res.* **1988,** *80*(2), 328-336.
23. Weast, R. C., *Handbook of Chemistry and Physics. 59th Ed*. Blackwell Scientific Publications Ltd.: 1978.
24. Cramer, L. E.; Spears, K. G., Hydrogen bond strengths from solvent-dependent lifetimes of Rose Bengal dye. *J. Am. Chem. Soc.* **1978,** *100*(1), 221-227.
25. Dijk, J. M. F. v.; Schuurmans, M. F. H., On the nonradiative and radiative decay rates and a modified exponential energy gap law for 4f–4f transitions in rare-earth ions. *J. Chem. Phys.* **1983,** *78*(9), 5317-5323.
26. de Jong, M.; Seijo, L.; Meijerink, A.; Rabouw, F. T., Resolving the ambiguity in the relation between Stokes shift and Huang–Rhys parameter. *Phys. Chem. Chem. Phys.* **2015,** *17*(26), 16959-16969.
